# A Fluorescent Probe as a Lead Compound for a Selective α-Synuclein PET Tracer: Development of a Library of 2-Styrylbenzothiazoles and Biological Evaluation of [^18^F]PFSB and [^18^F]MFSB

**DOI:** 10.1021/acsomega.3c04292

**Published:** 2023-08-15

**Authors:** Adriana Di Nanni, Ran Sing Saw, Umberto M. Battisti, Gregory D. Bowden, Adam Boeckermann, Kaare Bjerregaard-Andersen, Bernd J. Pichler, Kristina Herfert, Matthias M. Herth, Andreas Maurer

**Affiliations:** † Werner Siemens Imaging Center, Department of Preclinical Imaging and Radiopharmacy, Eberhard Karls University Tübingen, Röntgenweg 13, 72076 Tübingen, Germany; ‡ Department of Drug Design and Pharmacology, Faculty of Health and Medicinal Sciences, University of Copenhagen, Jagtvej 160, 2100 Copenhagen, Denmark; § Cluster of Excellence iFIT (EXC 2180) “Image-Guided and Functionally Instructed Tumor Therapies”, Eberhard Karls University Tübingen, 72076 Tübingen, Germany; ∥ Department of Antibody Engineering and Biochemistry, H. Lundbeck A/S, Ottiliavej 9, 2500 Valby, Denmark; ⊥ Department of Clinical Physiology, Nuclear Medicine & PET, Rigshospitalet, Blegdamsvej 9, 2100 Copenhagen, Denmark

## Abstract

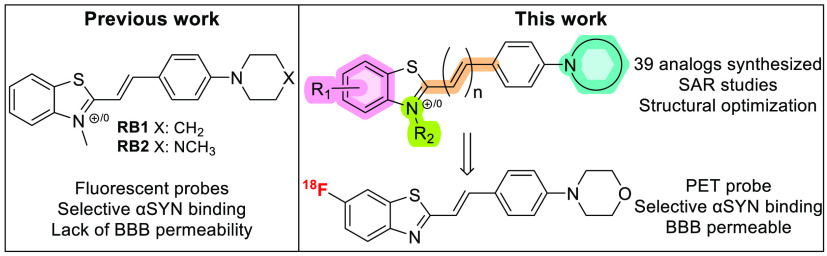

A method to detect and quantify aggregated α-synuclein (αSYN) fibrils *in vivo* would drastically impact the current understanding of multiple neurodegenerative diseases, revolutionizing their diagnosis and treatment. Several efforts have produced promising scaffolds, but a notable challenge has hampered the establishment of a clinically successful αSYN positron emission tomography (PET) tracer: the requirement of high selectivity over the other misfolded proteins amyloid β (Aβ) and tau. By designing and screening a library of 2-styrylbenzothiazoles based on the selective fluorescent probe **RB1**, this study aimed at developing a selective αSYN PET tracer. [^3^H]PiB competition binding assays identified **PFSB** (*K_i_* = 25.4 ± 2.3 nM) and its less lipophilic analogue **MFSB**, which exhibited enhanced affinity to αSYN (*K_i_* = 10.3 ± 4.7 nM) and preserved selectivity over Aβ. The two lead compounds were labeled with fluorine-18 and evaluated using *in vitro* autoradiography on human brain slices, where they demonstrated up to 4-fold increased specific binding in MSA cases compared to the corresponding control, reasonably reflecting selective binding to αSYN pathology. *In vivo* PET imaging showed [^18^F]**MFSB** successfully crosses the blood–brain barrier (BBB) and is taken up in the brain (SUV = 1.79 ± 0.02). Although its pharmacokinetic profile raises the need for additional structural optimization, [^18^F]**MFSB** represents a critical step forward in the development of a successful αSYN PET tracer by overcoming the major challenge of αSYN/Aβ selectivity.

## Introduction

Synucleinopathies include neurodegenerative diseases such as Parkinson’s disease (PD), multiple system atrophy (MSA), and dementia with Lewy bodies (DLB), which are all characterized by the accumulation of αSYN fibrils as a hallmark of pathogenesis.^1,2^ The establishment of a technique to quantitatively detect αSYN fibrils *in vivo* with the use of positron emission tomography (PET) would be crucial for the progress of the field, representing an invaluable tool for preclinical/clinical studies and early diagnosis, leading to an overall greater understanding of synucleinopathies.

Several studies have addressed this scientific need; however, to date, no αSYN PET tracer has reached clinical application. Multiple factors make targeting αSYN fibrils particularly challenging, specifically their low abundance (10- or more-fold lower than Aβ, hampering the achievement of high *B*_max_/*K_i_* ratios)^3,4^ intracellular location (in contrast to extracellular amyloid plaques), and coexistence with the structurally similar fibrils of the age-related misfolded proteins Aβ and tau.^5^ These factors highlight the import of high selectivity as a key feature of candidate αSYN tracers. An encouraging αSYN/Aβ selectivity was detected for a chalcone-based scaffold recently proposed by Kaide et al., but its pharmacokinetic profile was unsatisfactory.^6,7^ General criteria for the successful development of an αSYN PET tracer were outlined by the Michael J. Fox Foundation^8^ and are more comprehensively discussed by Korat et al., together with the most relevant scaffolds developed so far.^5^

Benzothiazoles represent a generally recognized pharmacophore for the target, as several studies have investigated αSYN ligands containing this moiety. However, despite their favorable affinity values, these compounds often lack selectivity over the other neurodegenerative aggregates.^9−11^ 2-(Pyridine-3-yl)benzothiazoles exhibited moderate αSYN/Aβ selectivity, but only qualitative results were presented.^12^ A 2-Styrylbenzoxazole produced by Verdurand et al. displayed high affinity to αSYN and moderate selectivity over Aβ (*K*_d-αSYN_ = 3.3 ± 2.8 nM, *K*_d-Aβ_ = 145.3 ± 114.5 nM) in fibril binding assays but showed no binding in human brain sections.^13^

**Figure 1:**
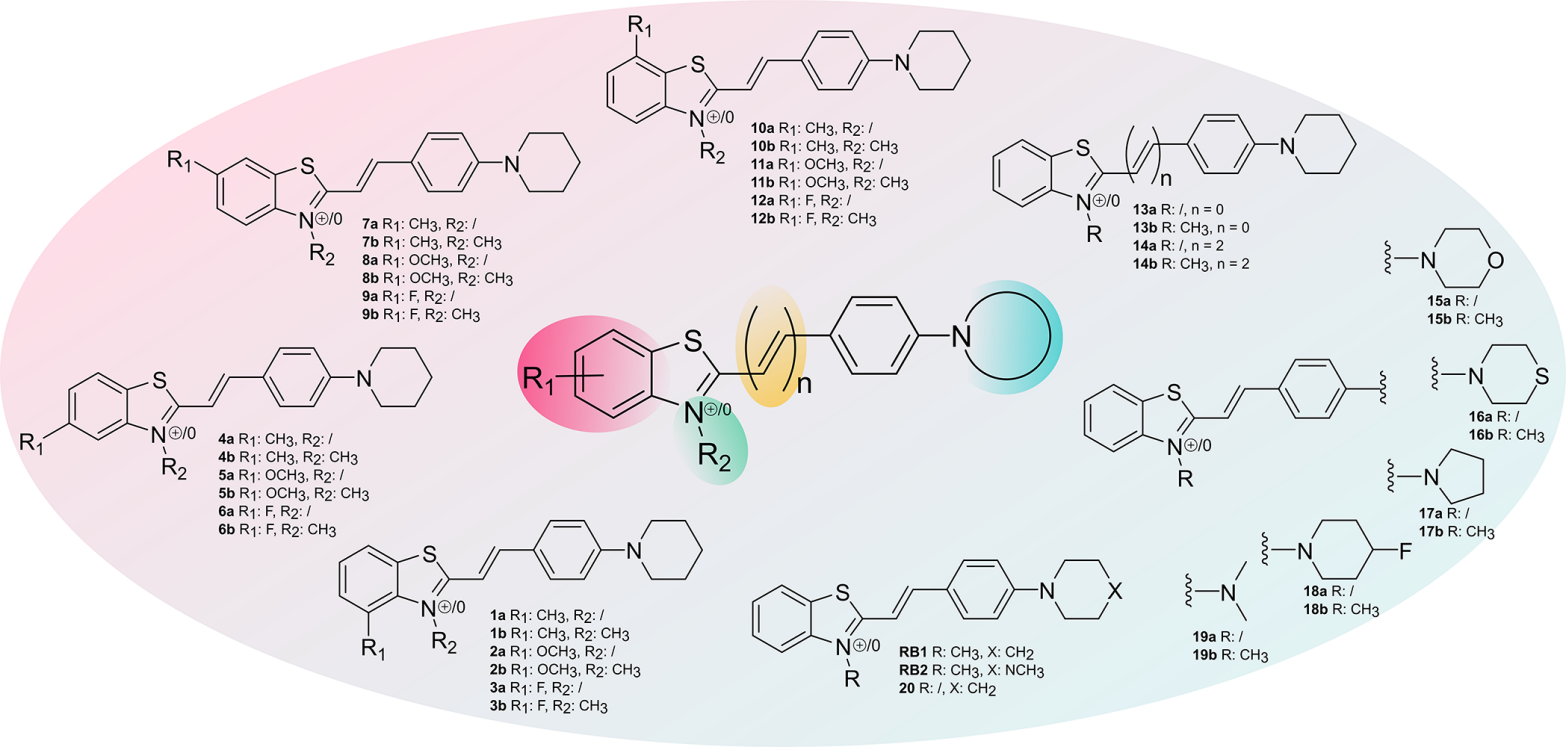
Development of a library of 2-styrylbenzothiazoles and structures of **RB1** and **RB2** fluorescent probes.

Gaur et al. developed the 2-styrylbenzothiazole-based fluorescent probes **RB1** and **RB2** (Figure 1) by modifying the structure of Thioflavin T (ThT) with the aim of improving both its fluorescence and binding properties. **RB1** exhibited selective imaging of αSYN fibrils in living cells as well as considerably higher affinity than its piperazine analogue **RB2** (*K*_d-**RB1**_ = 30 ± 10 nM, *K*_d-**RB2**_ = 4400 ± 500 nM).^14^

By adopting the 2-(4-amino-1yl)styrylbenzothiazole scaffold as a lead compound for the construction of a library, this work presents the development of a selective αSYN PET tracer.

## Results

### Development and *In Vitro* Evaluation of a 2-Styrylbenzothiazole-Based Library

A library of 2-styrylbenzothiazoles containing six subclasses of compounds was designed and developed to evaluate the effects of six different types of structural modifications on their affinity to αSYN fibrils (Figure 1). Methyl-, methoxy-, and fluoro-substitutions were applied at different positions on the benzothiazole ring (compounds **1a**–**12b**). The length of the conjugated system was modified by directly connecting the phenyl moiety with the benzothiazole or including two additional carbon atoms (compounds **13a**–**14b**). The impact of the nitrogen substitution patterns on binding affinity was also investigated (compounds **15a**–**19b**). A key feature of the parent compound **RB1** is the *N*-methylation of the benzothiazole ring, which results in a permanent positive charge, effectively hampering the crossing of the BBB. As brain uptake is an essential property for any central nervous system (CNS) tracer, nonmethylated analogues were synthesized for each structural modification to overcome this major drawback.

**Scheme 1:**
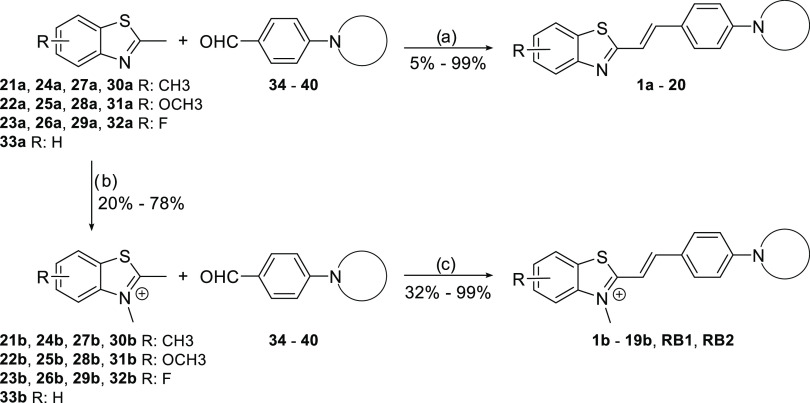
General Synthetic Pathway for *N*-Methylated and Nonmethylated 2-Styrylbenzothiazoles Reagents and conditions: (a) NaOH aq., dimethyl sulfoxide (DMSO), r.t., 2 to 24 h; (b) MeI or MeONs, MeCN, 80 °C, overnight; (c) EtOH, 80 °C, overnight.

**Scheme 2:**
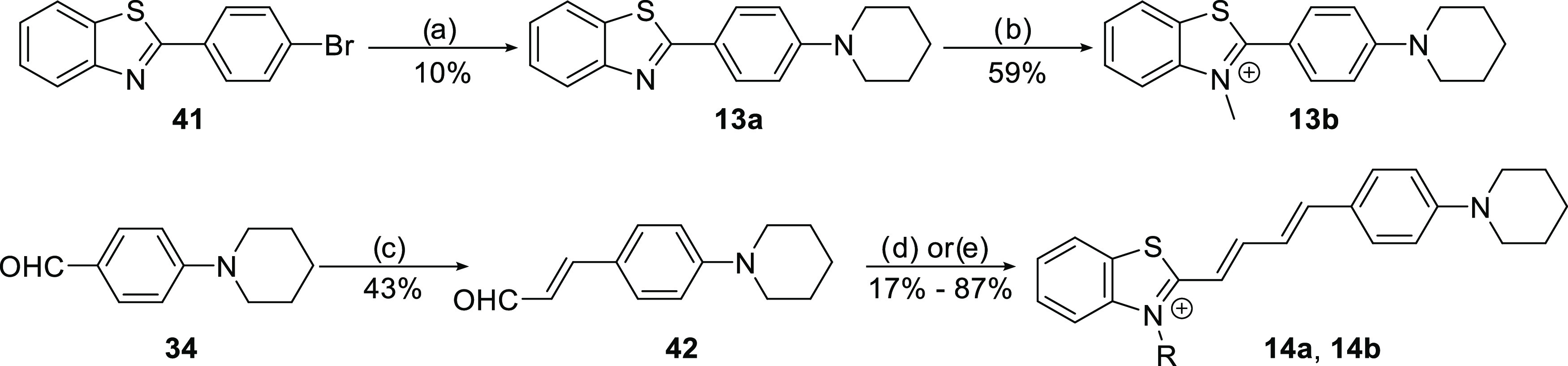
Synthetic Pathway for Analogues with Altered π-System Length Reagents and conditions: (a) Pd(PPh_3_)_4_, Cs_2_CO_3_, piperidine, toluene, 110 °C, 5 h; (b) MeONs, chlorobenzene, 80 °C, overnight; (c) H_2_SO_4_ conc., acetaldehyde, 0 °C, 1 h; (d) **33a**, DMSO, NaOH aq., r.t., 3 h; (e) **33b**, EtOH, 80 °C, overnight.

The diversely substituted benzothiazoles (compounds **21a**–**33a**) were produced via dedicated synthetic pathways depending on the availability of starting materials. All benzothiazoles were *N*-methylated by reacting them with methyl iodide or methyl nosylate, depending on their reactivity (compounds **21b**–**33b**, Scheme 1). Various 4-aminobenzaldehydes (compounds **34**–**40**) were synthesized by base-catalyzed substitution of 4-fluorobenzaldehyde with different amines. The benzothiazole and the benzaldehyde moieties were combined through condensation reactions to produce **RB1**, **RB2**, and 35 other analogues (Scheme 1). Pd(PPh_3_)_4_-catalyzed amination of 2-(4-bromophenyl)benzothiazole (**41**) and subsequent *N*-methylation afforded the ThT analogues **13a** and **13b** (Scheme 2). 4-Piperidinecinnamaldehyde (**42**) was synthesized from the corresponding benzaldehyde by reacting it with acetaldehyde in the presence of concentrated H_2_SO_4_, after which it was coupled with 2-methylbenzothiazoles to produce 1,3-butadiene derivatives **14a** and **14b** (Scheme 2).

The full library was screened for its binding affinity to αSYN fibrils via [^3^H]PiB competition assays (Tables 1 and 2) and showed substantial fluctuations depending on structural modification. Affinity was considerably improved in some analogues, up to *K_i_* = 9.6 nM, 19.7 nM for the 6-methoxy derivative **8b**.

**Table 1:**
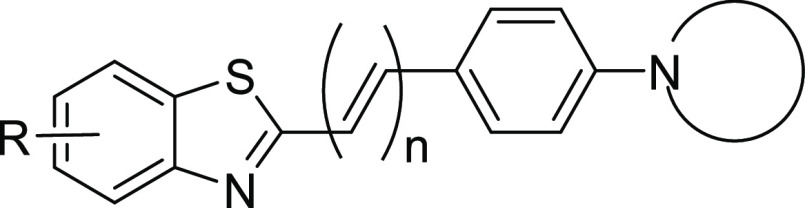
Binding Affinity of Nonmethylated Compounds Determined by [^3^H]PiB Competition Assays on αSYN Fibrils and Calculated Values for BBB Score and CNS MPO

#	R	n	*N*-substitution	*K_i_* (nM)	BBB score	CNS MPO
**1a**	4-CH3	1	*N*-piperidine	71.2; 198.3	4.79	3.0
**2a**	4-OCH3	1	*N*-piperidine	>400	4.90	3.3
**3a**	4-F	1	*N*-piperidine	186.6; 110.5	4.78	3.0
**4a**	5-CH3	1	*N*-piperidine	233.4; 88.1	4.79	3.0
**5a**	5-OCH3	1	*N*-piperidine	128.4; 187.8	4.90	3.3
**6a**	5-F	1	*N*-piperidine	169.1; 124.5	4.78	3.0
**7a**	6-CH3	1	*N*-piperidine	230.0; 217.8	4.79	3.0
**8a**	6-OCH3	1	*N*-piperidine	110.1; 102.9	4.90	3.3
**9a (PFSB)**	6-F	1	*N*-piperidine	25.4 ± 2.3^a^	4.78	3.0
**10a**	7-CH3	1	*N*-piperidine	>400	4.79	3.0
**11a**	7-OCH3	1	*N*-piperidine	>400	4.90	3.3
**12a**	7-F	1	*N*-piperidine	283.3^b^	4.78	3.0
**13a**	H	0	*N*-piperidine	134.5; 12.1	4.77	3.0
**14a**	H	2	*N*-piperidine	81.9 ± 15.6^a^	4.76	3.0
**15a**	H	1	*N*-morpholine	92.0 ± 30.3^a^	4.76	3.5
**16a**	H	1	*N*-thiomorpholine	219.1; 58.6	4.62	3.0
**17a**	H	1	*N*-pyrrolidine	73.4 ± 19.0^a^	4.81	3.0
**18a**	H	1	*N*-fluoropiperidine	99.8 ± 32.6^a^	4.68	3.0
**19a**	H	1	*N*-dimethylamine	>400	4.84	3.1
**20**	H	1	*N*-piperidine	170.5; 33.5	4.83	3.0

a Three data points available (mean *K_i_* ± SEM).

b Single data point available.

**Table 2:**
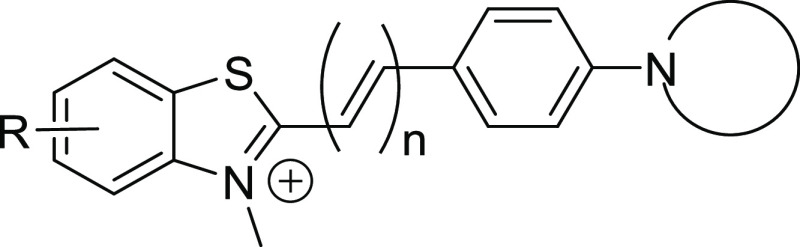
Binding Affinity of Methylated Compounds Determined by [^3^H]PiB Competition Assays on αSYN fibrils

#	R	n	*N*-substitution	*K_i_* (nM)
**RB1**	H	1	*N*-piperidine	>400
**RB2**	H	1	*N*-(*N*-methyl)piperazine	>400
**1b**	4-CH_3_	1	*N*-piperidine	36.1; 83.9
**2b**	4-OCH_3_	1	*N*-piperidine	70.8; 34.3
**3b**	4-F	1	*N*-piperidine	>400
**4b**	5-CH_3_	1	*N*-piperidine	84.6; 59.9
**5b**	5-OCH_3_	1	*N*-piperidine	27.5; 162.9
**6b**	5-F	1	*N*-piperidine	435.2; 268.2
**7b**	6-CH_3_	1	*N*-piperidine	16.8; 23.8
**8b**	6-OCH_3_	1	*N*-piperidine	19.7; 9.6
**9b**	6-F	1	*N*-piperidine	335.4; 43.6
**10b**	7-CH_3_	1	*N*-piperidine	231.3; 63.9
**11b**	7-OCH_3_	1	*N*-piperidine	233.7; 177.3
**12b**	7-F	1	*N*-piperidine	>400
**13b**	H	0	*N*-piperidine	>400
**14b**	H	2	*N*-piperidine	23.0; 16.8
**15b**	H	1	*N*-morpholine	>400
**16b**	H	1	*N*-thiomorpholine	>400
**17b**	H	1	*N*-pyrrolidine	144.7; 58.6
**18b**	H	1	*N*-fluoropiperidine	>400
**19b**	H	1	*N*-dimethylamine	>400

To cross-match the binding properties with the compounds’ predicted ability to reach the brain, calculations of BBB score and Central Nervous System Multiparameter Optimization (CNS MPO) were carried out according to the models proposed from the literature.^15,16^ The calculated values for all nonionic compounds were in the interval 4.62–4.90 for BBB score (range: 0–6) and 3.0–3.5 for CNS MPO (range: 0–5). As both parameters did not considerably differ among the analogues (Table 1), these predictions were not considered in the selection of a lead compound.

The most promising compounds were selected for further evaluation based on their affinity to αSYN, and their selectivity was assessed in [^3^H]PiB competition binding assays to Aβ fibrils. No appreciable competition with the tritium-labeled tracer was detected for any of the tested analogues, suggesting a high αSYN/Aβ selectivity characterizes the 2-styrylbenzothiazoles in this library (Figures 3b and S5).

### Structural Optimization

**Figure 2:**
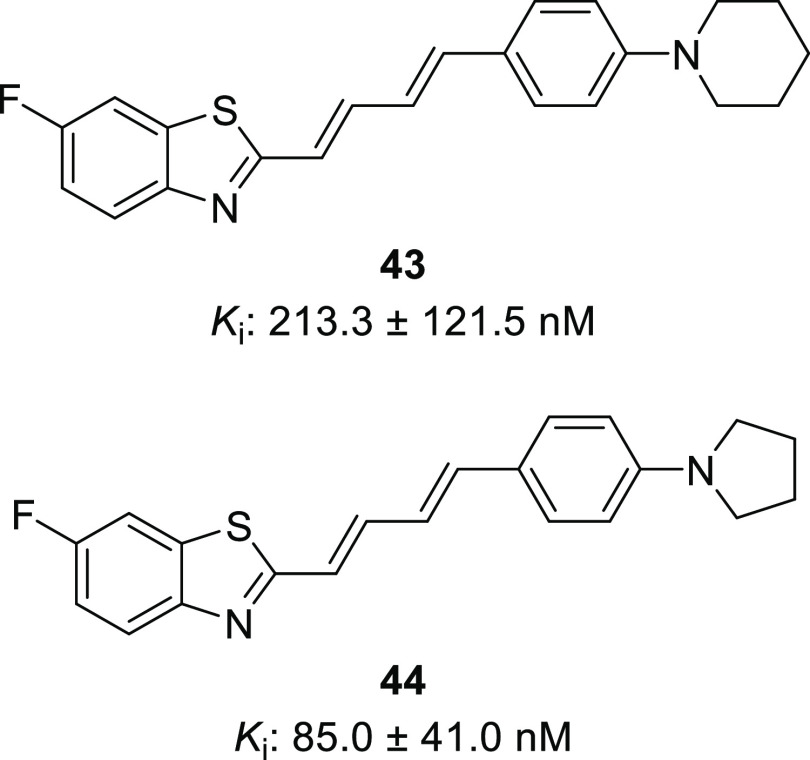
Structure of compounds combining 6-fluoro substitution, extended π-system, and *N*-piperidine/*N*-pyrrolidine and their αSYN binding affinity (mean *K_i_* ± SEM, *n* = 3) determined by [^3^H]PiB competition assays.

From these results, some structural features were recognized to favor αSYN fibril binding. With the purpose of further optimization, we combined these components to develop compounds **43** and **44**. The 6-fluorobenzothiazole moiety was selected as it was found to impart lower *K_i_* values and provided a site for the inclusion of ^18^F. Additionally, a longer π-system (*n* = 2) was adopted along with *N*-pyrrolidine substitution (Figure 2). Although binding to Aβ remained low (Figure S5), competition binding assays against [^3^H]PiB showed a decrease in affinity to αSYN fibrils (*K_i_*_-43_: 213.9 ± 121.5 nM, *K_i_*_-44_: 85.0 ± 41.0 nM).

**Figure 3:**
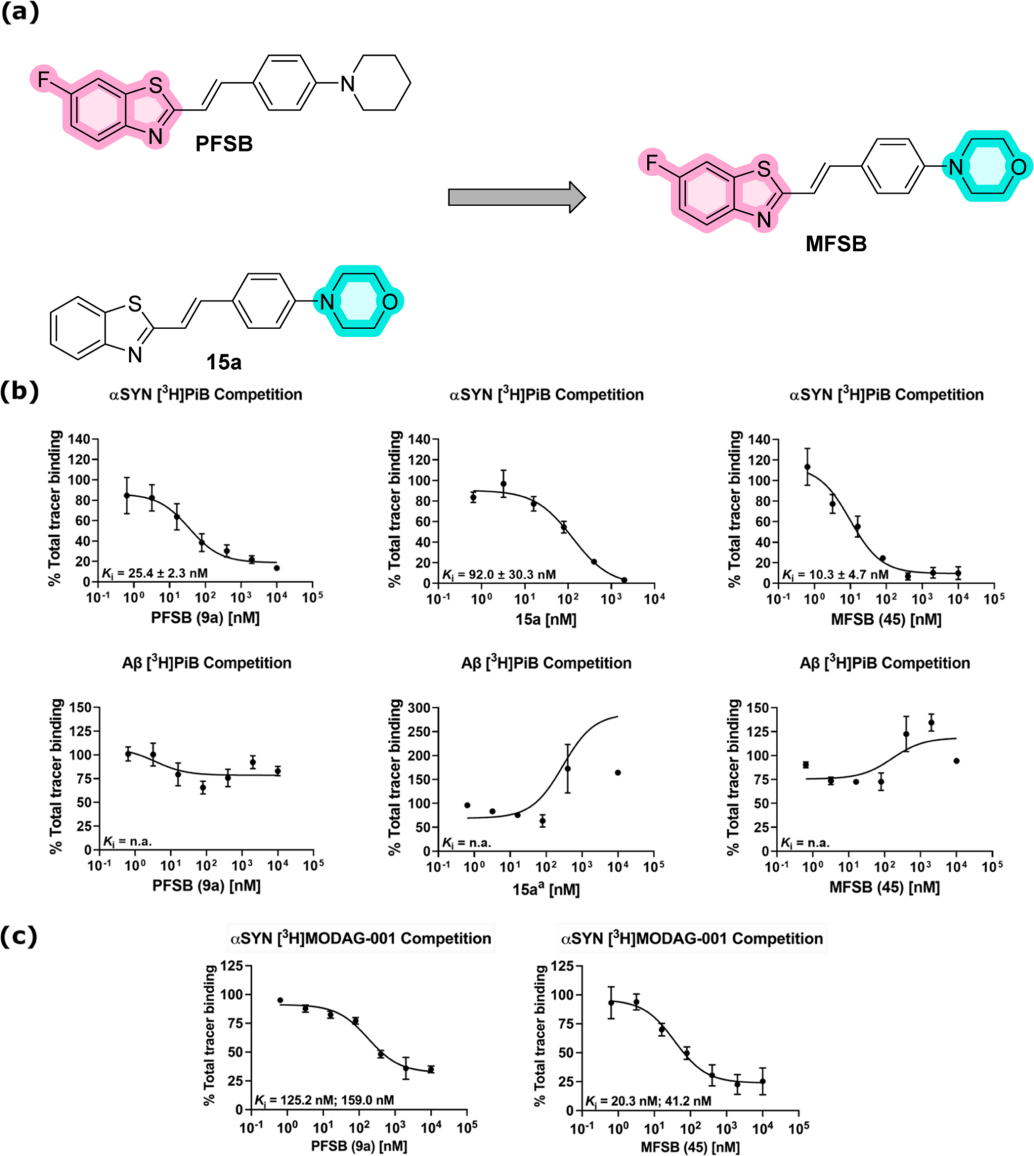
(a) Development of the less lipophilic analogue **MFSB**; (b) binding affinity of **PFSB**, **MFSB**, and **15a** to αSYN and Aβ fibrils determined by [^3^H]PiB competition assays (*n* = 3, ^a^: *n* = 2); (c) competition of **PFSB** and **MFSB** with [^3^H]MODAG-001 for αSYN binding (*n* = 2).

In addition to pursuing the enhancement of binding affinity, the selection of favorable moieties also aimed to improve pharmacokinetics. As the cLog *P* of most compounds within the library was ≥5.5 (Table S2), it raised a concern regarding lipophilicity and prompted us to design a more hydrophilic derivative. The *N*-morpholine compound **15a** had shown comparable affinity to its *N*-piperidine analogue **20**, as well as a substantially lower cLog *P* (4.57, Table S2) and the highest CNS MPO among the evaluated compounds (3.5, Table S2). As compound **9a** (**PFSB**) was selected from the first set of analogues for radiolabeling and further evaluation, the *N*-morpholine moiety was chosen to be combined with a 6-fluorobenzothiazole to produce **MFSB** (**45**, Figure 3a). [^3^H]PiB competition binding assays proved affinity was not only comparable to **PFSB** but improved by a 2.5 factor (*K_i_*_-MFSB_: 10.3 ± 4.7 nM; *K_i_*_-PFSB_: 25.4 ± 2.3 nM;). αSYN/Aβ selectivity remained unchanged (Figure 3b).

Due to scaffold similarity, all fibril binding assays adopted the benzothiazole-based [^3^H]PiB as a radiolabeled competitor, with the aim of evaluating the impact of structural modification across the library on the binding to αSYN. To assess their affinity in a less scaffold-relative manner, **PFSB** and **MFSB** were also evaluated in competition with [^3^H]MODAG-001, the current standard in αSYN preclinical imaging, due to its low *K*_d_ value of 0.6 ± 0.1 nM.^17^ Both compounds demonstrated moderate to good competition, with **MFSB** exhibiting a 4.6-fold lower *K_i_* than **PFSB** (*K_i_*_-PFSB_: 125.2 nM, 159.0 nM, *K_i_*_-MFSB_: 20.3 nM, 41.2 nM, Figure 3c).

### Radiolabeling of [^18^F]PFSB and [^18^F]MFSB

**Scheme 3:**
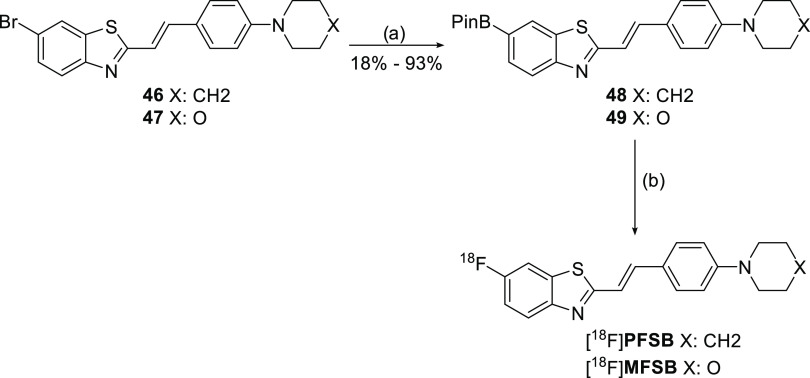
General Synthetic Pathway for the Synthesis of BPin Precursors and Fluorine-18 Labeling Reagents and conditions: (a) B_2_Pin_2_, KOAc, Pd(dppf)Cl_2_, *N*,*N*-dimethylformamide (DMF), 100 °C, 45 min; (b) Cu(OTf)_2_, pyridine, [^18^F]TBAF, *n-*BuOH 10% in DMA, 120 °C, 20 min.

From the first set of compounds, PFSB was selected for radiolabeling and further evaluation. A bromo-substituted analogue (**46**) was synthesized according to the general procedure (Scheme 1) and reacted in a Pd(dppf)Cl_2_-catalyzed borylation to generate a pinacol boronate precursor (**48**) (Scheme 3). A copper-mediated radiofluorination (CMRF) was established according to standard conditions. The amount of precursor and pyridine was optimized manually (Table S1), while Cu(OTf)_2_, *n-*BuOH, and *N*,*N*-dimethylacetamide (DMA) remained constant in all experiments. As precursor load did not seem to affect the reaction efficiency in the investigated range, a lower amount was chosen due to the compound’s poor solubility. Increased pyridine concentration negatively impacted radiochemical conversion (RCC).^18^ According to these results, entry (b) was selected as a starting point for automation (precursor 10 μmol, pyridine 60 μmol, Table S1). [^18^F]**PFSB** was produced with a radiochemical yield (RCY) of 5.8 ± 1.3% and a molar activity (*A*_m_) of 36.5 ± 8.5 GBq/μmol (*n* = 3).

The same general procedure was applied for precursor synthesis (**49**) and radiolabeling of the morpholine analogue [^18^F]**MFSB** (Scheme 3) to afford the tracer with an RCY of 11.6 ± 2.9% and an *A*_m_ of 41.2 ± 12.0 GBq/μmol (*n* = 3) so that the two compounds could be evaluated in parallel. Analytical results from both tracers are reported in the Supporting Information (Figures S1–S4).

### *In Vitro* Autoradiography and *In Vivo* PET Imaging

**Figure 4:**
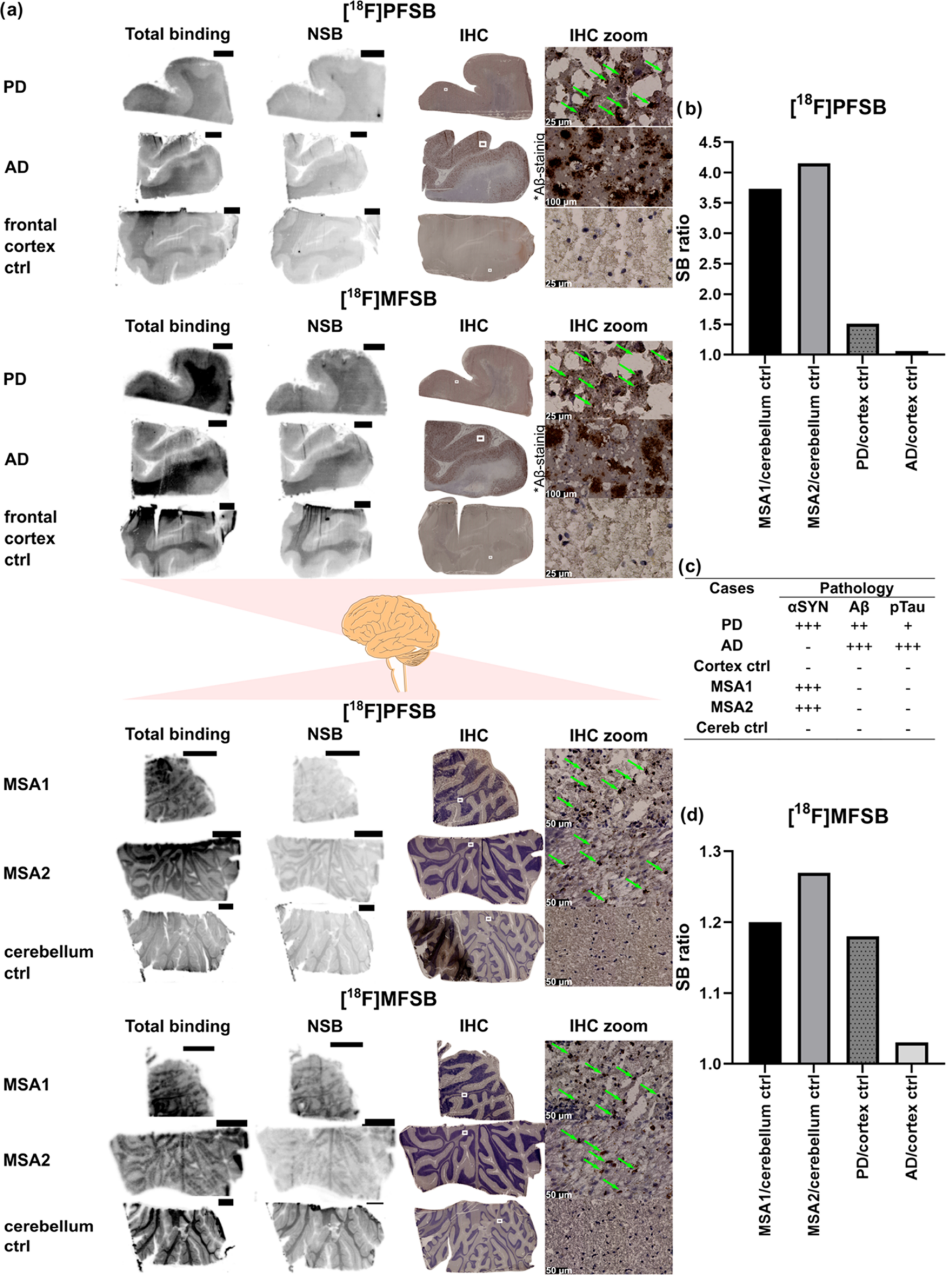
*In vitro* autoradiography of [^18^F]**PFSB** and [^18^F]**MFSB** on human brain slices: (a) total binding and nonspecific binding on consecutive slices for each case and corresponding IHC with αSYN-staining (MSA, PD, and Ctrl tissues) and Aβ-staining (AD tissue); pathological aggregates are highlighted by arrows; scale bar in autoradiography samples: 0.5 cm; (b) quantitative analysis (*n* = 1) of [^18^F]**PFSB** via SB_disease_/SB_ctrl_ ratio; (c) the extent of pathology in each subject case, indicated by the symbols “+” and “–“; the number of “+” symbols indicates increasing pathology from low (+), moderate (++) to high (+++); “–” symbolizes the absence of pathology; (d) quantitative analysis (*n* = 1) of [^18^F]**MFSB** via SB_disease_/SB_ctrl_ ratio.

*In vitro* autoradiography of [^18^F]**PFSB** on human brain slices was performed to validate our preliminary results from the fibril binding assays. The experiment corroborated the binding profile previously observed with the tracer showing affinity to αSYN pathology and selectivity over Aβ (Figure 4). Brain slices from MSA patients exhibited a particularly high specific binding (SB), with a signal intensity ratio of 3.7 and 4.2 compared to the control investigating the same brain area (Figure 4b, Table S3). Instead, the AD sample showed no increased binding in the regions where immunohistochemistry (IHC) detected the presence of Aβ plaques (AD/frontal cortex SB ratio: 1.06). However, nonspecific binding (NSB) to the white matter area proved to be an issue in all samples (Figure 4).

The comparison of [^18^F]**PFSB** with its less lipophilic morpholine analogue was expected to reduce NSB. However, SB ratios between patient slices (MSA and PD) and their corresponding controls (cerebellum and frontal cortex, respectively) appeared considerably reduced. While the autoradiography of [^18^F]**MFSB** displayed no successful decrease in NSB, it validated the αSYN selectivity of the 2-styrylbenzothiazoles in the library as the AD/frontal cortex SB ratio in the gray matter area remained favorable (Figure 4d).

**Figure 5:**
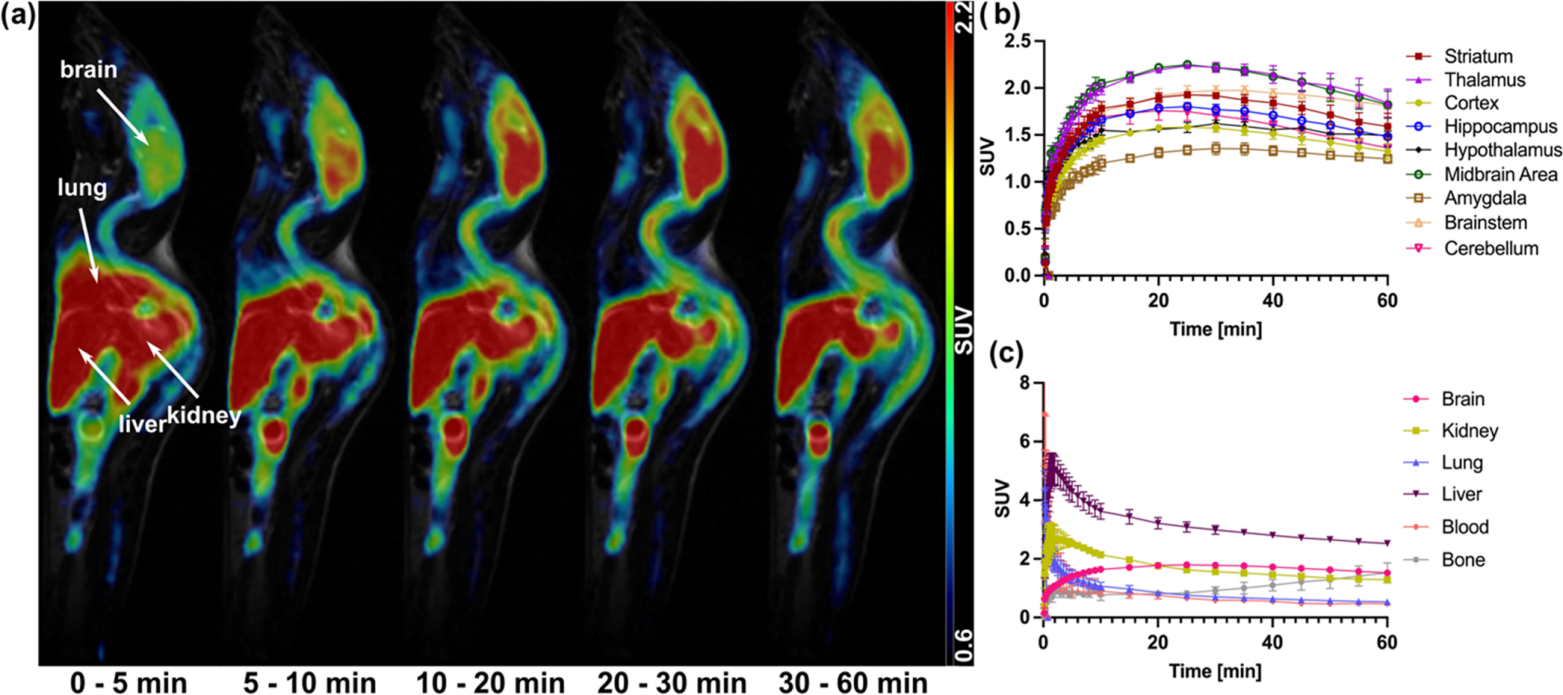
*In vivo* evaluation of [^18^F]**MFSB** pharmacokinetic profile: (a) whole-body PET/MR sagittal images at different time points; (b) time–activity curves in brain regions; (c) time–activity curves of whole brain, kidney, lung, liver, blood, and bone. Abbreviation: SUV, standardized uptake value.

To assess its pharmacokinetic profile, [^18^F]**MFSB** was injected into three healthy wild-type mice, and its distribution was evaluated over 60 min via dynamic PET imaging. Crucially, the experiment showed the compound successfully crossed the BBB (SUV = 1.79 ± 0.02, SUV_brain_/SUV_blood_ = 2.7), albeit with slow brain uptake and insufficient washout (Figure 5). Increased uptake in white-matter-rich regions such as the midbrain and brainstem is likely a reflection of high NSB. Outside the CNS, high uptake was detected in the lungs and liver in the first 2 min (SUV_lung_ = 4.43 ± 0.33, SUV_liver_ = 5.00 ± 0.35, Table S5), followed by fast to moderate clearance. Notable kidney uptake was also observed, showing renal excretion in the first few minutes (Figure 5, Table S5). Blood concentration overall remained low, indicating the tracer is unlikely to bind to plasma proteins. Bone uptake was detected only at later stages, with SUV reaching 1.52 ± 0.20 at 60 min (Table S5). As *in vivo* studies were solely conducted in wild-type mice, no blocking experiments were performed at this stage.

## Discussion

### Development and *In Vitro* Evaluation of a 2-Styrylbenzothiazole-Based Library

The results from [^3^H]PiB competition binding assays using **RB1** and **RB2** aligned with the literature. By titrating solutions of both probes with αSYN fibrils and detecting the resulting increase in fluorescence intensity, Gaur et al. demonstrated that **RB1** selectively binds to αSYN. At the same time, the binding was reduced by 2 orders of magnitude for its *N*-methylpiperazine analogue **RB2**.^14^ In our radioactivity-based experiments, the former probe exhibited only moderate-to-low affinity, but the **RB1**/**RB2** ratio remained somewhat consistent (*K_i_*_-**RB1**_: 480.8 nM, *K_i_*_-**RB2**_: 333.5 μM).

Based on the *in vitro* evaluation of the library of compounds, we could define some structure–activity relationships (SAR) for the interaction of 2-styrylbenzothiazoles with αSYN fibrils.

The impact of structural modification proved to be noncomparable between ionic and nonionic analogues, as different patterns were observed in affinity fluctuation. For *N*-methylated compounds, methyl and methoxy substitution afforded similar results within the same position, while the corresponding fluoro-substituted benzothiazoles exhibited higher *K_i_* values. The shortening of the π-system substantially decreased affinity (**13b**), while its extension with an additional double bond improved affinity to a *K_i_* < 20 nM (**14b**). A common trend shared by both methylated and nonmethylated derivatives highlighted 7-substituted benzothiazoles as the least favorable substitution position, with the 6-substitution affording the highest affinity. In nonionic analogues, alterations on the vinylaniline moiety (π-system length and *N*-substitution) produced *K_i_* values comparable to the original scaffold **20**, except for greater affinity loss for the *N*-dimethyl analogue **19a**. A similar modification of the *N*-substitution was applied to ThT derivatives in a recent study by Needham et al.^19^ Our results are in agreement both with the affinity trend they observed within nonionic compounds and with the considerable affinity decrease in their cationic analogues.

**Figure 6:**
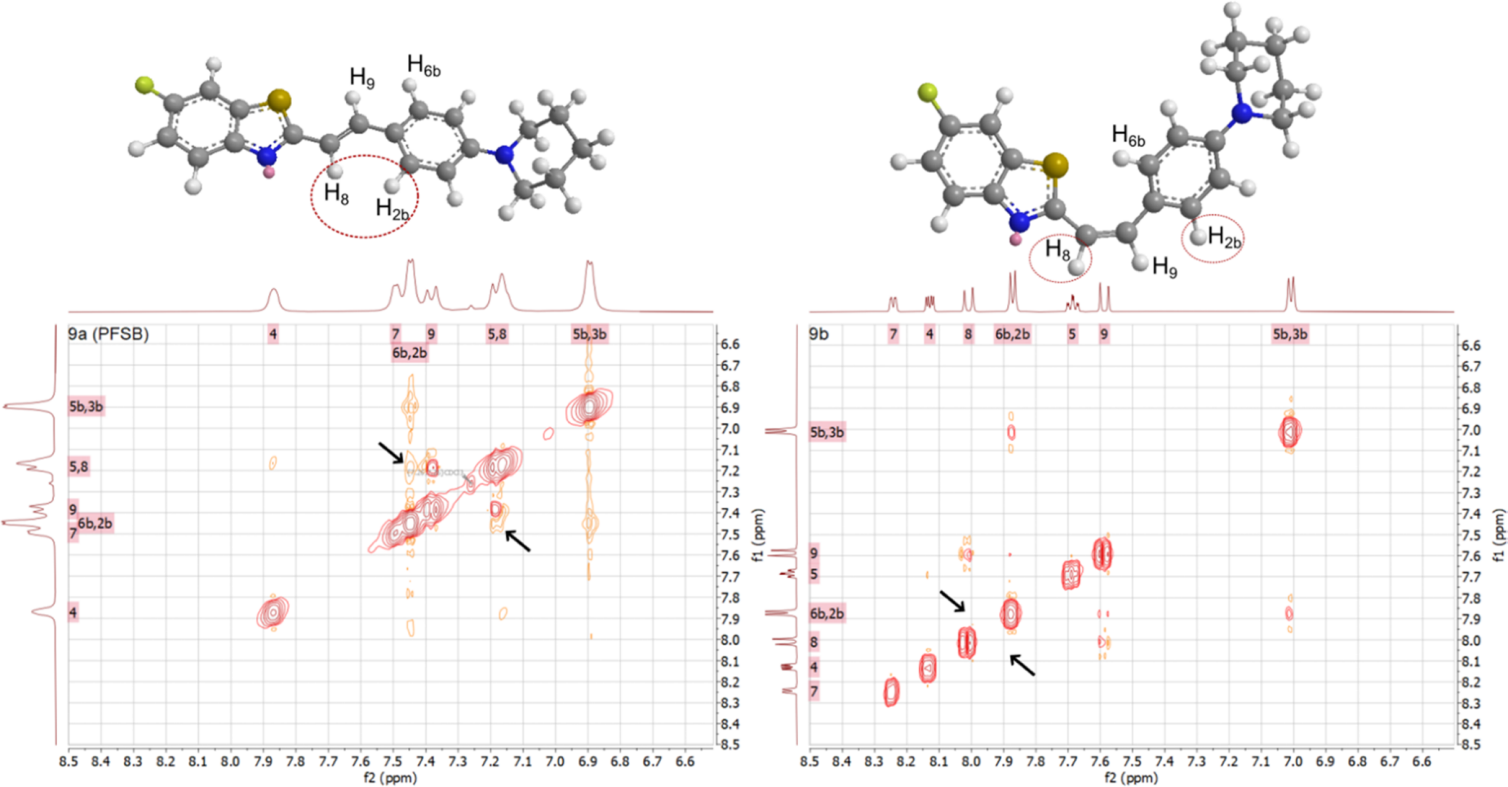
Three-dimensional predicted conformation of the (*E*)- and (*Z*)-isomers of the general structure of **9a** and **9b** (MM2 energy minimization calculated via Chem3D 20.1, PerkinElmer Informatics) and 2D ^1^H NOESY NMR spectra of **PFSB** (**9a**, left) and **9b** (right). In the first spectrum, a weak interaction between H_8_ and H_2b_H_6b_ shows that the sample is a mixture of (*E*)-**9a** and (*Z*)-**9a**. Such interaction is not detected in the second spectrum, highlighting (*Z*)-**9b** as the preferential configuration for the *N*-methylated compound.

We suggest that the different impact of structural alterations on the neutral and cationic compounds is related to the configuration of their double bond. Nonionic derivatives proved to be a mixture of (*E*)- and (*Z*)-isomers, while *N*-methylated analogues, synthesized via a different procedure, were preferentially in the *Z* configuration (illustrated by ^1^H NOESY NMR, Figure 6). Similar affinity alterations were observed in the *E*/*Z* stereoisomers of the indolinone derivatives developed by Chu et al.^20^

Following the SAR analysis, we were able to exclude the ionic derivatives from further evaluation: *N*-methylation interferes with the impact of other structural modifications but does not produce an affinity improvement *per se*. Therefore, permanently charged analogues were not pursued as the ionic state would preclude BBB penetration.^21,22^

According to BBB score and CNS MPO,^15,16^ all nonmethylated compounds were over a threshold of suitable properties and were expected to reach the brain. However, no structural modification substantially affected the estimated crossing of BBB: this prediction was not taken into consideration for the selection of a lead compound.

We identified analogues **9a**, **14a**, **15a**, and **17a** as the most promising αSYN ligands and screened them for their affinity to Aβ_1–42_ fibrils. None of the tested compounds appreciably competed against [^3^H]PiB (Figures 3b and S5). The reliability of the assay was confirmed by direct competition of nonradioactive PiB against the corresponding tritium-labeled tracer resulting in consistent binding curves (*K_i_* = 156.6, 165.9 nM, Figure S6). These results were indeed encouraging on the potential high selectivity of 2-styrylbenzothiazoles and prompted us to further validate them by direct interaction of radiolabeled compounds with human tissue.

### Structural Optimization

The combination of promising features into compounds **43** and **44** failed to further improve the binding properties and pointed out the nonlinearity of affinity enhancements following structural modifications on the 2-styrylbenzothiazole scaffold.

While the 6-fluoro substitution substantially decreased the *K_i_* of **PFSB** compared to its analogue **20**, it led to an ∼3-fold reduction of affinity in **43** compared to **14a**. A minor affinity decrease was also observed in the combination of 6-fluorobenzothiazole and the diene moiety with an *N*-pyrrolidine substitution as compound **44** showed a slightly higher *K_i_* than its parent compound **17a**. Instead, **44** was comparable with its nonfluorinated *N*-piperidine analogue **14a**. When focusing on the aromatic nitrogen substitution, these results aligned with both our previous findings (Tables 1 and 2) and the literature:^19^*N*-Pyrrolidine derivatives exhibit lower *K_i_* values than their corresponding analogues with diverse *N*-substitutions.

Multiple studies have shown the extension of the π-system by the addition of a double bond to often favor the binding to αSYN. Chu et al. observed consistently decreased *K_i_* values switching from indolinones to indolinone dienes, while Ono et al. found gradually enhanced affinities in their series of *n* = 1, 2, 3, 4 chalcone derivatives.^20,23^ Hsieh et al. pointed out the positive impact on the affinity of the increased intramolecular distance between hydrogen-bond acceptors,^24^ which may be a possible explanation for this trend. However, while the distance between nitrogen atoms clearly is one of the factors influencing binding affinity in our library, our findings show that it does not improve *K_i_* values *per se*. Overall, results on diene-enclosing compounds should be carefully interpreted due to potential photosensitivity.^25^

The simultaneous attempt to improve the pharmacokinetic properties of our potential radioligands via the synthesis of **MFSB** succeeded in the development of a second lead compound with slightly improved binding and considerably reduced lipophilicity.

The observed competition of both lead compounds, **PFSB** and **MFSB**, against the non-scaffold-related [^3^H]MODAG-001 validates 2-styrylbenzothiazoles as a scaffold with an encouraging affinity to αSYN, in addition to their promising αSYN/Aβ selectivity.

### *In Vitro* Autoradiography and *In Vivo* PET Imaging

*In vitro* autoradiography experiments confirmed the preliminary results afforded by the fibril binding assays both by displaying tracer binding in MSA and PD brain tissues with αSYN pathology and by highlighting the lack of binding in AD brain tissues which contain an abundance of Aβ plaques and tau pathology. Despite our attempt to produce a second tracer with decreased lipophilicity, NSB to the white matter remained high in both tracer candidates and hampered the accurate quantification of autoradiography results in the experiment evaluating [^18^F]**MFSB**, therefore producing a low SB ratio between pathological and healthy tissues.

Both tracers were produced as a mixture of the (*E*)- and (*Z*)-stereoisomers (Figures S1 and S3, Supporting Information). As we previously discussed the impact of the double bond configuration on the binding to the target, we speculate that SB could potentially be improved by the identification and isolation of the isomer with the highest affinity to αSYN fibrils.

The most relevant finding provided by the autoradiography experiment was the ability of both tracers to differentiate between misfolded proteins. Although some scaffolds successfully afforded a moderate αSYN/Aβ selectivity,^5^ the complete lack of binding to Aβ plaques has been displayed solely by the 4-nitrophenyl chalcone derivatives developed by Kaide et al. However, these compounds displayed insufficient clearance when injected in the healthy brain of ddY mice.^6,7^ To the best of our knowledge, 2-styrylbenzothiazoles are the only other scaffold exhibiting such selectivity but additionally offer the basis for extensive structural optimization in the perspective of lower lipophilicity.

While the pharmacokinetic profile requires some improvement to reduce NSB and allow for the detection of αSYN pathology, our *in vivo* PET results were indeed encouraging: [^18^F]**MFSB** crosses the BBB and exhibits moderate brain uptake, with an SUV peak up to 1.58 in the frontal cortex and up to 1.76 in the cerebellum. These findings lay the foundations for the development of enhanced MFSB derivatives and, potentially, the establishment of a clinical αSYN PET radioligand. Further evaluation may be needed to assess the feasibility of fluorescence-based assays with these novel 2-styrylbenzothiazole derivatives in comparison with the original scaffold.

## Conclusions

We developed a library of 2-styrylbenzothiazoles by modification of a fluorescent probe, identified a lead compound based on its binding affinity to αSYN fibrils, and established two fluorine-18 labeled radioligands.

Despite the need for further structural optimization in order to improve pharmacokinetic properties and NSB, [^18^F]**MFSB** shows promising potential to overcome the major challenge of αSYN/Aβ selectivity and exhibits BBB penetration *in vivo*. Therefore, we believe 2-styrylbenzothiazoles are an excellent starting point for the establishment of a successful scaffold and represent a crucial turning point in the development of αSYN PET tracers.

## Experimental Section

### Chemistry

All chemicals were purchased from Sigma-Aldrich (St. Louis, MO), abcr GmbH (Karlsruhe, Germany), or Carl Roth (Karlsruhe, Germany) and used without any further purification.

Reaction progress was monitored by thin-layer chromatography (TLC) on 0.20 mm Polygram SIL G/UV_254_ (silica gel 60) TLC plates (Macherey-Nagel, Düren, Germany) with the chosen eluent mixture and/or analytical HPLC-MS (quadrupole 6120 series ESI detector, Agilent, Santa Clara, CA) equipped with a Luna 5 μm C18 (2) 100 Å 50 mm × 2 mm column (Phenomenex, Torrance, CA) [gradient: 0–7.60 min (0–100% B), 7.60–8.80 (100% B), 8.80–9.30 min (100–0% B), 9.30–13.0 min (0% B); solvent A: 0.1% formic acid in H_2_O; solvent B: MeCN; 0.4 mL/min] or with a Zorbax Eclipse XBD-C18 50 mm × 4.6 mm column (Agilent, Santa Clara, CA) [gradient: 0–6 min (0–100% B); solvent A: H_2_O/MeCN/formic acid 95:5:0.1 v/v%; solvent B: 0.1% formic acid in MeCN; 1 mL/min].

Purification was performed through automated flash chromatography on an Isolera 4 system (Biotage, Uppsala, Sweden) or a CombiFlash NextGen 300+ (Teledyne ISCO, Lincoln, NE).

^1^H, ^13^C, and ^19^F NMR spectra were acquired on an Avance III AV 600 (^1^H: 600.13 MHz; ^13^C: 150.61 MHz) or an Avance II AV 400 (^1^H: 400 MHz; ^13^C: 101 MHz; ^19^F: 376 MHz) spectrometer (Bruker Biospin, Ettlingen, Germany). All chemical shifts (δ) are reported as parts per million (ppm) and referenced to residual solvent peaks (CDCl_3_: δ_H_ = 7.26, δ_C_ = 77.16; DMSO-*d*_6_: δ_H_ = 2.50, δ_C_ = 39.52). All compounds are >95% pure by HPLC analysis.

### General Procedure A

To a solution of the selected 2-methylbenzothiazole (6.70 mmol) and the selected 4-aminobenzaldehyde or 4-aminocinnamaldehyde (7.37 mmol) in DMSO (6.50 mL), NaOH aq. 18 M (6.50 mL) was slowly added. The mixture was stirred at room temperature for 2 to 24 h. A yellow precipitate was formed. The mixture was diluted with water, and complete precipitation was allowed. The precipitate was filtered under vacuum to afford a yellow solid which was recrystallized from EtOAc.

### General Procedure B

According to the literature procedure,^14^ a solution of the selected 2,3-dimethylbenzothiazolium salt (0.86 mmol) and the selected 4-aminobenzaldehyde or 4-aminocinnamaldehyde (1.83 mmol) in EtOH (7.00 mL) was refluxed overnight. The color turned to red/purple. The precipitate was filtered under vacuum and washed with EtOAc to afford a dark solid, which was recrystallized from EtOH and/or Et_2_O.

#### 4-Methyl-2-(4-(piperidin-1-yl)styryl)benzo[*d*]thiazole (**1a**)

The synthesis was carried out according to general procedure A (46 mg, 60%). *R_f_*: 0.48 (Hept/EtOAc 3:1). ^1^H NMR (600 MHz, CDCl_3_) δ 7.69–7.63 (m, 1H), 7.47 (d, *J =* 8.8 Hz, 2H), 7.39 (d, *J =* 16.1 Hz, 1H), 7.30 (d, *J =* 16.1 Hz, 1H), 7.25–7.20 (m, 2H), 6.91 (d, *J =* 8.7 Hz, 2H), 3.28 (t, *J =* 5.6 Hz, 4H), 2.75 (s, 3H), 1.70 (p, *J =* 5.6 Hz, 4H), 1.66–1.60 (m, 2H). ^13^C NMR (151 MHz, CDCl_3_) δ 167.1, 153.5, 152.6, 137.9, 134.1, 132.7, 128.8, 126.8, 125.6, 125.0, 119.0, 118.9, 115.5, 49.6, 25.7, 24.5, 18.7. HPLC-MS (ESI): *m*/*z* calcd for C_21_H_22_N_2_S 334.15; [M + H]^+^ found 335.25.

#### 3,4-Dimethyl-2-(4-(piperidin-1-yl)styryl)benzo[*d*]thiazol-3-ium 4-nitrobenzenesulfonate (**1b**)

The synthesis was carried out according to general procedure B (119 mg, 59%). *R_f_*: 0.19 (DCM/MeOH 5%). ^1^H NMR (400 MHz, DMSO-*d*_6_) δ 8.19 (d, *J =* 8.3 Hz, 2H), 8.12 (br s, 1H), 8.01 (d, *J =* 15.3 Hz, 1H), 7.89 (d, *J =* 8.5 Hz, 2H), 7.83 (d, *J =* 8.2 Hz, 2H), 7.66 (d, *J =* 15.3 Hz, 1H), 7.54 (s, 2H), 7.04 (d, *J =* 8.7 Hz, 2H), 4.43 (s, 3H), 3.49 (s, 4H), 2.91 (s, 3H), 1.61 (s, 6H). ^13^C NMR (101 MHz, DMSO-*d*_6_) δ 171.5, 154.4, 153.5, 149.6, 147.2, 140.9, 132.8, 132.7, 127.7, 127.7, 127.3, 126.9, 123.3, 122.3, 121.7, 113.5, 107.0, 47.6, 40.0, 25.0, 23.9, 20.9. HPLC-MS (ESI): *m*/*z* calcd for C_22_H_25_N_2_S^+^ 349.17; [M]^+^ found 349.16.

#### 4-Methoxy-2-(4-(piperidin-1-yl)styryl)benzo[*d*]thiazole (**2a**)

The synthesis was carried out according to general procedure A (10 mg, 5%). *R_f_*: 0.18 (Hept/EtOAc 3:1). ^1^H NMR (600 MHz, CDCl_3_) δ 7.45 (dt, *J =* 8.7, 2.9 Hz, 2H), 7.41 (dd, *J =* 8.0, 0.9 Hz, 1H), 7.38 (d, *J =* 16.1 Hz, 1H), 7.31 (d, *J =* 16.1 Hz, 1H), 7.28 (t, *J =* 8.0 Hz, 1H), 6.91 (dd, *J =* 8.8, 2.7 Hz, 2H), 6.88 (dd, *J =* 8.0, 0.9 Hz, 1H), 4.05 (s, 3H), 3.27 (t, *J =* 5.6 Hz, 4H), 1.70 (p, *J =* 5.6 Hz, 4H), 1.65–1.60 (m, 2H). ^13^C NMR (151 MHz, CDCl_3_) δ 166.9, 153.3, 152.6, 144.2, 137.7, 135.8, 128.8, 126.0, 125.6, 119.0, 115.5, 113.6, 106.7, 56.0, 49.6, 25.7, 24.5. HPLC-MS (ESI): *m*/*z* calcd for C_21_H_22_N_2_OS 350.15; [M + H]^+^ found 351.13.

#### 4-Methoxy-3-methyl-2-(4-(piperidin-1-yl)styryl)benzo[*d*]thiazol-3-ium Iodide (**2b**)

The synthesis was carried out according to general procedure B (46 mg, quant.). *R_f_*: 0.24 (DCM/MeOH 5%). ^1^H NMR (600 MHz, DMSO-*d*_6_) δ 7.97 (d, *J =* 15.3 Hz, 1H), 7.87 (d, *J =* 8.6 Hz, 2H), 7.83 (d, *J =* 8.1 Hz, 1H), 7.66–7.52 (m, 2H), 7.37 (d, *J =* 8.2 Hz, 1H), 7.04 (d, *J =* 8.6 Hz, 2H), 4.43 (s, 3H), 4.04 (s, 3H), 3.49 (t, *J =* 5.3 Hz, 4H), 1.77–1.51 (m, 6H). ^13^C NMR (151 MHz, DMSO-*d*_6_) δ 170.7, 153.5, 149.7, 149.3, 132.7, 131.1, 128.7, 128.6, 122.3, 115.5, 113.5, 111.7, 106.9, 56.9, 47.5, 39.3, 25.1, 23.9. HPLC-MS (ESI): *m*/*z* calcd for C_22_H_25_N_2_OS^+^ 365.17; [M]^+^ found 365.25.

#### 4-Fluoro-2-(4-(piperidin-1-yl)styryl)benzo[*d*]thiazole (**3a**)

The synthesis was carried out according to general procedure A (187 mg, 56%). *R_f_*: 0.43 (Hept/EtOAc 3:1). ^1^H NMR (400 MHz, CDCl_3_) δ 7.58 (dd, *J =* 8.0, 1.0 Hz, 1H), 7.51–7.40 (m, 3H), 7.30–7.24 (m, 2H), 7.14 (ddd, *J =* 10.5, 8.2, 1.0 Hz, 1H), 6.91 (dt, *J =* 8.8, 8.8, 2.7 Hz, 1H), 3.34–3.22 (m, 4H), 1.76–1.66 (m, 4H), 1.66–1.59 (m, 2H). ^13^C NMR (101 MHz, CDCl_3_) δ 168.7, 155.6 (d, *J*_C-F_*=* 255.6 Hz), 152.7, 143.0 (d, *J*_C-F_*=* 13.4 Hz), 139.1, 136.9 (d, *J*_C-F_*=* 3.6 Hz), 129.0, 125.6 (d, *J*_C-F_*=* 7.0 Hz), 125.1, 118.1, 117.2 (d, *J*_C-F_*=* 4.3 Hz), 115.3, 112.0 (d, *J*_C-F_*=* 18.0 Hz), 49.4, 25.6, 24.5. ^19^F NMR (376 MHz, CDCl_3_) δ −122.8. HPLC-MS (ESI): *m*/*z* calcd for C_20_H_19_FN_2_S 338.13; [M + H]^+^ found 339.10.

#### 4-Fluoro-3-methyl-2-(4-(piperidin-1-yl)styryl)benzo[*d*]thiazol-3-ium 4-nitrobenzenesulfonate (**3b**)

The synthesis was carried out according to general procedure B (165 mg, 76%). *R_f_*: 0.12 (DCM/MeOH 5%). ^1^H NMR (400 MHz, DMSO-*d*_6_) δ 8.19 (d, *J =* 8.3 Hz, 2H), 8.14–8.09 (m, 1H), 8.06 (d, *J =* 15.3 Hz, 1H), 7.91 (d, *J =* 8.6 Hz, 2H), 7.83 (d, *J =* 8.4 Hz, 2H), 7.73–7.57 (m, 3H), 7.04 (d, *J =* 8.7 Hz, 2H), 4.33 (d, *J =* 2.3 Hz, 3H), 3.52 (t, *J =* 5.1 Hz, 4H), 1.79–1.49 (m, 6H). ^13^C NMR (101 MHz, DMSO-*d*_6_) δ 172.1, 154.4, 153.7, 151.0, 150.5 (d, *J*_C-F_*=* 252.0 Hz), 147.2, 133.3, 130.5 (d, *J*_C-F_*=* 9.8 Hz), 129.3, 128.3 (d, *J*_C-F_*=* 8.0 Hz), 126.9, 123.3, 122.2, 120.1 (d, *J*_C-F_*=* 4.0 Hz), 115.9 (d, *J*_C-F_*=* 20.2 Hz), 113.4, 106.0, 47.5, 38.3 (d, *J*_C-F_*=* 11.1 Hz), 25.1, 23.9. ^19^F NMR (376 MHz, DMSO-*d*_6_) δ −125.2. HPLC-MS (ESI): *m*/*z* calcd for C_21_H_22_FN_2_S^+^ 353.15; [M]^+^ found 353.13.

#### 5-Methyl-2-(4-(piperidin-1-yl)styryl)benzo[*d*]thiazole (**4a**)

The synthesis was carried out according to general procedure A (100 mg, 43%). *R_f_*: 0.44 (Hept/EtOAc 3:1). ^1^H NMR (400 MHz, CDCl_3_) δ 7.75 (s, 1H), 7.69 (d, *J =* 8.1 Hz, 1H), 7.46 (d, *J =* 8.6 Hz, 2H), 7.42 (d, *J =* 16.1 Hz, 1H), 7.21 (d, *J =* 16.1 Hz, 1H), 7.16 (dd, *J =* 8.1, 1.6 Hz, 1H), 6.91 (d, *J =* 8.6 Hz, 2H), 3.28 (t, *J =* 5.5 Hz, 4H), 2.49 (s, 3H), 1.70 (p, *J =* 5.5 Hz, 4H), 1.64 (d, *J =* 5.8 Hz, 4H). ^13^C NMR (101 MHz, CDCl_3_) δ 168.3, 154.5, 152.6, 137.8, 136.3, 131.2, 128.9, 126.6, 125.5, 122.8, 121.0, 118.6, 115.5, 49.6, 25.7, 24.5, 21.6. HPLC-MS (ESI): *m*/*z* calcd for C_21_H_22_N_2_S 334.15; [M + H]^+^ found 335.10.

#### 3,5-Dimethyl-2-(4-(piperidin-1-yl)styryl)benzo[*d*]thiazol-3-ium Iodide (**4b**)

The synthesis was carried out according to general procedure B (278 mg, 99%). *R_f_*: 0.21 (DCM/MeOH 5%). ^1^H NMR (400 MHz, DMSO-*d*_6_) δ 8.17 (d, *J =* 8.3 Hz, 1H), 8.01 (d, *J =* 15.4 Hz, 1H), 7.96 (br s, 1H), 7.88 (d, *J =* 8.8 Hz, 2H), 7.63 (d, *J =* 15.4 Hz, 1H), 7.53 (dd, *J =* 8.3, 1.5 Hz, 1H), 7.04 (d, *J =* 8.8 Hz, 2H), 4.21 (s, 3H), 3.49 (t, *J =* 5.2 Hz, 4H), 2.54 (s, 3H), 1.71–1.50 (m, 6H). ^13^C NMR (101 MHz, DMSO-*d*_6_) δ 171.3, 153.5, 149.2, 142.2, 139.3, 132.7, 128.8, 123.9, 123.3, 122.3, 115.9, 113.4, 107.0, 47.5, 35.6, 25.1, 23.9, 21.1. HPLC-MS (ESI): *m*/*z* calcd for C_22_H_25_N_2_S^+^ 349.17; [M]^+^ found 349.15.

#### 5-Methoxy-2-(4-(piperidin-1-yl)styryl)benzo[*d*]thiazole (**5a**)

The synthesis was carried out according to general procedure A (170 mg, 58%). *R_f_*: 0.29 (Hept/EtOAc 3:1). ^1^H NMR (400 MHz, CDCl_3_) δ 7.67 (d, *J =* 8.7 Hz, 1H), 7.51–7.40 (m, 4H), 7.18 (d, *J =* 16.1 Hz, 1H), 6.98 (dd, *J =* 8.7, 2.5 Hz, 1H), 6.91 (dt, *J =* 8.8, 2.9 Hz, 2H), 3.89 (s, 3H), 3.35–3.21 (m, 4H), 1.77–1.65 (m, 4H), 1.62 (ddd, *J =* 7.7, 5.9, 3.4 Hz, 2H). ^13^C NMR (101 MHz, CDCl_3_) δ 169.3, 159.2, 155.4, 152.6, 137.6, 128.9, 126.2, 125.5, 121.7, 118.3, 115.4, 115.0, 105.3, 55.7, 49.5, 25.7, 24.5. HPLC-MS (ESI): *m*/*z* calcd for C_21_H_22_N_2_OS 350.15; [M + H]^+^ found 351.30.

#### 5-Methoxy-3-methyl-2-(4-(piperidin-1-yl)styryl)benzo[*d*]thiazol-3-ium Iodide (**5b**)

The synthesis was carried out according to general procedure B (222 mg, 97%). *R_f_*: 0.19 (DCM/MeOH 5%). ^1^H NMR (400 MHz, DMSO-*d*_6_) δ 8.18 (d, *J =* 8.9 Hz, 1H), 7.99 (d, *J =* 15.4 Hz, 1H), 7.87 (d, *J =* 8.8 Hz, 2H), 7.63 (d, *J =* 2.3 Hz, 1H), 7.62 (d, *J =* 15.4 Hz, 1H), 7.32 (dd, *J =* 8.9, 2.3 Hz, 1H), 7.04 (d, *J =* 8.8 Hz, 2H), 4.22 (s, 3H), 3.94 (s, 3H), 3.48 (t, *J =* 5.2 Hz, 4H), 1.73–1.51 (m, 6H). ^13^C NMR (101 MHz, DMSO-*d*_6_) δ 171.9, 160.5, 153.4, 148.6, 143.4, 132.6, 124.5, 122.3, 118.5, 116.6, 113.5, 107.2, 99.9, 56.3, 47.5, 35.7, 25.0, 23.9. HPLC-MS (ESI): *m*/*z* calcd for C_22_H_25_N_2_OS^+^ 365.17; [M]^+^ found 365.40.

#### 5-Fluoro-2-(4-(piperidin-1-yl)styryl)benzo[*d*]thiazole (**6a**)

The synthesis was carried out according to general procedure A (402 mg, 99%). *R_f_*: 0.45 (Hept/EtOAc 3:1). ^1^H NMR (400 MHz, CDCl_3_) δ 7.73 (dd, *J =* 8.8, 5.1 Hz, 1H), 7.62 (dd, *J =* 9.6, 2.5 Hz, 1H), 7.51–7.41 (m, 3H), 7.19 (d, *J =* 16.1 Hz, 1H), 7.09 (td, *J =* 8.8, 2.5 Hz, 1H), 6.91 (dt, *J =* 8.9, 3.0 Hz, 2H), 3.35–3.23 (m, 4H), 1.78–1.66 (m, 4H), 1.66–1.58 (m, 2H). ^13^C NMR (101 MHz, CDCl_3_) δ 170.6, 162.1 (d, *J*_C-F_*=* 242.8 Hz), 155.1 (d, *J*_C-F_*=* 12.1 Hz), 152.7, 138.6, 129.7 (d, *J*_C-F_*=* 1.9 Hz), 129.1, 125.1, 122.1 (d, *J*_C-F_*=* 9.9 Hz), 118.0, 115.3, 113.4 (d, *J*_C-F_*=* 25.0 Hz), 108.8 (d, *J*_C-F_*=* 23.6 Hz), 49.4, 25.7, 24.5. ^19^F NMR (376 MHz, CDCl_3_) δ −116.3. HPLC-MS (ESI): *m*/*z* calcd for C_20_H_19_FN_2_S 338.13; [M + H]^+^ found 339.12.

#### 5-Fluoro-3-methyl-2-(4-(piperidin-1-yl)styryl)benzo[*d*]thiazol-3-ium Iodide (**6b**)

The synthesis was carried out according to general procedure B (175 mg, quant.). *R_f_*: 0.19 (DCM/MeOH 5%). ^1^H NMR (400 MHz, DMSO-*d*_6_) δ 8.34 (dd, *J =* 9.0, 5.1 Hz, 1H), 8.12 (dd, *J =* 9.7, 2.4 Hz, 1H), 8.06 (d, *J =* 15.3 Hz, 1H), 7.89 (d, *J =* 8.9 Hz, 2H), 7.64–7.55 (m, 2H), 7.04 (d, *J =* 8.9 Hz, 2H), 4.19 (s, 3H), 3.51 (t, *J =* 5.2 Hz, 4H), 1.71–1.54 (m, 6H). ^13^C NMR (101 MHz, DMSO-*d*_6_) δ 173.7, 162.8 (d, *J*_C-F_*=* 245.4 Hz), 154.2, 150.5, 143.7 (d, *J*_C-F_*=* 12.6 Hz), 133.6, 126.1 (d, *J*_C-F_*=* 10.0 Hz), 123.1 (d, *J*_C-F_*=* 2.1 Hz), 122.7, 116.1 (d, *J*_C-F_*=* 24.6 Hz), 113.9, 107.2, 104.0 (d, *J*_C-F_*=* 28.9 Hz), 48.0, 36.3, 25.6, 24.4. ^19^F NMR (376 MHz, DMSO-*d*_6_) δ −111.1. HPLC-MS (ESI): *m*/*z* calcd for C_21_H_22_FN_2_S^+^ 353.15; [M]^+^ found 353.14.

#### 6-Methyl-2-(4-(piperidin-1-yl)styryl)benzo[*d*]thiazole (**7a**)

The synthesis was carried out according to general procedure A (132 mg, 26%). *R_f_*: 0.31 (Hept/EtOAc 3:1). ^1^H NMR (400 MHz, CDCl_3_) δ 7.89 (d, *J =* 8.3 Hz, 1H), 7.69 (d, *J =* 1.7 Hz, 1H), 7.53 (dt, *J =* 8.8, 2.6 Hz, 1H), 7.47 (d, *J =* 16.1 Hz, 1H), 7.35–7.28 (m, 2H), 6.99 (d, *J =* 8.4 Hz, 2H), 3.47–3.23 (m, 4H), 2.55 (s, 3H), 1.78 (p, *J =* 5.7 Hz, 4H), 1.73–1.62 (m, 3H). ^13^C NMR (151 MHz, CDCl_3_) δ 167.1, 152.6, 152.2, 137.6, 135.2, 134.5, 128.8, 127.8, 125.6, 122.2, 121.3, 118.6, 115.5, 49.6, 25.7, 24.5, 21.7. HPLC-MS (ESI): *m*/*z* calcd for C_21_H_22_N_2_S 334.15; [M + H]^+^ found 335.10.

#### 3,6-Dimethyl-2-(4-(piperidin-1-yl)styryl)benzo[*d*]thiazol-3-ium Iodide (**7b**)

The synthesis was carried out according to general procedure B (273 mg, 97%). *R_f_*: 0.27 (DCM/MeOH 5%). ^1^H NMR (600 MHz, DMSO-*d*_6_) δ 8.10 (s, 1H), 8.01 (d, *J =* 15.3 Hz, 2H), 8.00 (d, *J =* 8.6 Hz, 1H), 7.87 (d, *J =* 8.8 Hz, 2H), 7.62 (d, *J =* 15.3 Hz, 1H), 7.61 (dd, *J =* 8.6, 1.6 Hz, 1H), 7.04 (d, *J =* 8.8 Hz, 2H), 4.22 (s, 3H), 3.49 (t, *J =* 5.2 Hz, 4H), 2.51 (s, 1H), 1.70–1.62 (m, 2H), 1.62–1.56 (m, 4H). ^13^C NMR (151 MHz, DMSO-*d*_6_) δ 170.5, 153.5, 149.1, 140.1, 137.9, 132.7, 130.1, 126.9, 123.3, 122.3, 115.7, 113.5, 107.0, 47.5, 35.6, 25.1, 23.9, 20.9. HPLC-MS (ESI): *m*/*z* calcd for C_22_H_25_N_2_S^+^ 349.17; [M]^+^ found 349.16.

#### 6-Methoxy-2-(4-(piperidin-1-yl)styryl)benzo[*d*]thiazole (**8a**)

The synthesis was carried out according to general procedure A (95 mg, 19%). *R_f_*: 0.22 (Hept/EtOAc 3:1). ^1^H NMR (400 MHz, CDCl_3_) δ 7.83 (d, *J* = 8.9 Hz, 1H), 7.45 (d, *J =* 8.4 Hz, 2H), 7.34 (d, *J =* 16.1 Hz, 1H), 7.29 (d, *J =* 2.6 Hz, 1H), 7.19 (d, *J =* 16.1 Hz, 1H), 7.04 (dd, *J =* 8.9, 2.6 Hz, 1H), 6.91 (d, *J =* 8.4 Hz, 2H), 3.88 (s, 3H), 3.27 (t, *J =* 5.4 Hz, 4H), 1.70 (p, *J =* 5.6 Hz, 4H), 1.62 (q, *J =* 6.5 Hz, 2H). ^13^C NMR (151 MHz, CDCl_3_) δ 165.8, 157.8, 152.5, 148.7, 137.1, 135.7, 128.7, 125.6, 123.2, 118.6, 115.5, 115.4, 104.4, 56.0, 49.6, 25.7, 24.5. HPLC-MS (ESI): *m*/*z* calcd for C_21_H_22_N_2_OS 350.15; [M + H]^+^ found 351.15.

#### 6-Methoxy-3-methyl-2-(4-(piperidin-1-yl)styryl)benzo[*d*]thiazol-3-ium Iodide (**8b**)

The synthesis was carried out according to general procedure B (89 mg, 32%). *R_f_*: 0.18 (DCM/MeOH 5%). ^1^H NMR (400 MHz, DMSO-*d*_6_) δ 8.02 (d, *J =* 9.2 Hz, 1H), 7.99–7.91 (m, 2H), 7.86 (d, *J =* 8.5 Hz, 2H), 7.60 (d, *J =* 15.4 Hz, 1H), 7.38 (d, *J =* 9.2 Hz, 1H), 7.03 (d, *J =* 8.6 Hz, 2H), 4.21 (s, 3H), 3.90 (s, 3H), 3.57–3.44 (m, 4H), 1.82–1.47 (m, 6H). ^13^C NMR (101 MHz, DMSO-*d*_6_) δ 169.2, 158.9, 153.4, 148.3, 136.1, 132.4, 128.7, 122.4, 117.6, 117.0, 113.5, 107.3, 106.7, 56.2, 47.5, 35.8, 25.0, 23.9. HPLC-MS (ESI): *m*/*z* calcd for C_22_H_25_N_2_OS^+^ 365.17; [M]^+^ found 365.14.

#### 6-Fluoro-2-(4-(piperidin-1-yl)styryl)benzo[*d*]thiazole (**9a**–PFSB)

The synthesis was carried out according to general procedure A (34 mg, 24%). *R_f_*: 0.38 (Hept/EtOAc 3:1). ^1^H NMR (400 MHz, CDCl_3_) δ 7.87 (dd, *J =* 8.9, 4.8 Hz, 1H), 7.50 (dd, *J =* 8.2, 2.6 Hz, 1H), 7.46 (d, *J =* 8.4 Hz, 2H), 7.39 (d, *J =* 16.1 Hz, 1H), 7.23–7.12 (m, 2H), 6.91 (d, *J =* 8.6 Hz, 2H), 3.28 (t, *J =* 5.3 Hz, 4H), 1.86–1.66 (m, 4H), 1.67–1.57 (m, 2H). ^13^C NMR (101 MHz, CDCl_3_) δ 167.9 (d, *J*_C-F_*=* 3.3 Hz), 160.5 (d, *J*_C-F_*=* 245.1 Hz), 152.7, 150.8 (d, *J*_C-F_*=* 1.7 Hz), 138.3, 135.3 (d, *J*_C-F_*=* 11.0 Hz), 128.9, 125.2, 123.4 (d, *J*_C-F_*=* 9.4 Hz), 118.1, 115.4, 114.7 (d, *J*_C-F_*=* 24.6 Hz), 107.8 (d, *J*_C-F_*=* 26.8 Hz), 49.5, 25.7, 24.5. ^19^F NMR (376 MHz, CDCl_3_) δ −116.4. HPLC-MS (ESI): *m*/*z* calcd for C_20_H_19_FN_2_S 338.13; [M + H]^+^ found 339.15.

#### 6-Fluoro-3-methyl-2-(4-(piperidin-1-yl)styryl)benzo[*d*]thiazol-3-ium Iodide (**9b**)

The synthesis was carried out according to general procedure B (96 mg, 88%). *R_f_*: 0.16 (DCM/MeOH 5%). ^1^H NMR (400 MHz, DMSO-*d*_6_) δ 8.25 (dd, *J =* 8.3, 2.7 Hz, 1H), 8.15 (dd, *J =* 9.1, 4.3 Hz, 1H), 8.04 (d, *J =* 15.3 Hz, 1H), 7.89 (d, *J =* 8.8 Hz, 2H), 7.70 (td, *J =* 9.1, 2.7 Hz, 1H), 7.62 (d, *J =* 15.3 Hz, 1H), 7.04 (d, *J =* 8.8 Hz, 2H), 4.22 (s, 3H), 3.50 (t, *J =* 5.2 Hz, 4H), 1.70–1.51 (m, 6H). ^13^C NMR (101 MHz, DMSO-*d*_6_) δ 172.1 (d, *J*_C-F_*=* 1.6 Hz), 161.0 (d, *J*_C-F_*=* 246.6 Hz), 154.1, 150.4, 139.3, 133.4, 129.0 (d, *J*_C-F_*=* 12.0 Hz), 122.7, 118.2 (d, *J*_C-F_*=* 9.5 Hz), 117.6 (d, *J*_C-F_*=* 25.6 Hz), 113.9, 111.1 (d, *J*_C-F_*=* 28.8 Hz), 107.3, 48.0, 36.4, 25.6, 24.4. ^19^F NMR (376 MHz, DMSO-*d*_6_) δ −112.6. HPLC-MS (ESI): *m*/*z* calcd for C_21_H_22_FN_2_S^+^ 353.15; [M]^+^ found 353.13.

#### 7-Methyl-2-(4-(piperidin-1-yl)styryl)benzo[*d*]thiazole (**10a**)

The synthesis was carried out according to general procedure A (28 mg, 43%). *R_f_*: 0.40 (Hept/EtOAc 3:1). ^1^H NMR (400 MHz, CDCl_3_) δ 7.79 (d, *J =* 8.1 Hz, 1H), 7.54–7.40 (m, 3H), 7.36 (t, *J =* 7.7 Hz, 1H), 7.23 (d, *J =* 16.1 Hz, 1H), 7.13 (d, *J =* 7.3 Hz, 1H), 6.91 (d, *J =* 8.4 Hz, 2H), 3.28 (t, *J =* 5.3 Hz, 4H), 2.56 (s, 3H), 1.70 (p, *J =* 5.4 Hz, 4H), 1.65–1.62 (m, 2H). ^13^C NMR (101 MHz, CDCl_3_) δ 167.7, 154.0, 152.5, 138.0, 134.7, 131.6, 128.9, 126.4, 125.6, 125.3, 120.1, 118.6, 115.6, 49.7, 25.6, 24.5, 21.6. HPLC-MS (ESI): *m*/*z* calcd for C_21_H_22_N_2_S 334.15; [M + H]^+^ found 335.15.

#### 3,7-Dimethyl-2-(4-(piperidin-1-yl)styryl)benzo[*d*]thiazol-3-ium Iodide (**10b**)

The synthesis was carried out according to general procedure B (11 mg, 64%). *R_f_*: 0.19 (DCM/MeOH 5%). ^1^H NMR (600 MHz, DMSO-*d*_6_) δ 8.13 (d, *J =* 15.3 Hz, 1H), 7.95 (d, *J =* 8.4 Hz, 1H), 7.88 (d, *J =* 8.8 Hz, 2H), 7.72 (dd, *J =* 8.4, 7.4 Hz, 1H), 7.66 (d, *J =* 15.3 Hz, 1H), 7.54 (d, *J =* 7.4 Hz, 1H), 7.05 (d, *J =* 8.8 Hz, 2H), 4.24 (s, 3H), 3.51 (t, *J =* 5.4 Hz, 4H), 2.60 (s, 3H), 1.70–1.62 (m, 2H), 1.62–1.55 (m, 4H). ^13^C NMR (151 MHz, DMSO-*d*_6_) δ 170.7, 153.6, 150.1, 141.8, 132.9, 132.7, 129.3, 127.9, 126.6, 122.3, 113.7, 113.5, 106.8, 47.5, 35.9, 25.1, 23.9, 19.3. HPLC-MS (ESI): *m*/*z* calcd for C_22_H_25_N_2_S^+^ 349.17; [M]^+^ found 349.20.

#### 7-Methoxy-2-(4-(piperidin-1-yl)styryl)benzo[*d*]thiazole (**11a**)

The synthesis was carried out according to general procedure A and purified by flash chromatography (PE/EtOAc 1–20% B) to afford the product as a yellow solid (25 mg, 13%). *R_f_*: 0.41 (PE/EtOAc 4:1). ^1^H NMR (600 MHz, CDCl_3_) δ 7.60 (d, *J =* 8.1 Hz, 1H), 7.52–7.45 (m, 3H), 7.39 (t, *J =* 8.0 Hz, 1H), 7.24 (d, *J =* 15.8 Hz, 1H), 7.03 (s, 2H), 6.80 (d, *J =* 8.0 Hz, 1H), 3.99 (s, 3H), 3.30 (t, *J =* 5.5 Hz, 4H), 1.89–1.70 (m, 4H), 1.68–1.62 (m, 2H). ^13^C NMR (151 MHz, CDCl_3_) δ 168.4, 155.6, 154.4, 154.3, 137.6, 129.0, 127.2, 126.8, 122.8, 119.2, 116.0, 115.5, 105.3, 56.1, 50.0, 25.3, 24.0. HPLC-MS (ESI): *m*/*z* calcd for C_21_H_22_N_2_OS 350.15; [M + H]^+^ found 351.15.

#### 7-Methoxy-3-methyl-2-(4-(piperidin-1-yl)styryl)benzo[*d*]thiazol-3-ium Iodide (**11b**)

The synthesis was carried out according to general procedure B (21 mg, 46%). *R_f_*: 0.21 (DCM/MeOH 5%). ^1^H NMR (600 MHz, DMSO-*d*_6_) δ 8.12 (d, *J =* 15.3 Hz, 1H), 7.87 (d, *J =* 8.6 Hz, 2H), 7.75 (t, *J =* 8.2 Hz, 1H), 7.69 (d, *J =* 8.5 Hz, 1H), 7.63 (d, *J =* 15.3 Hz, 1H), 7.31 (d, *J =* 8.1 Hz, 1H), 7.05 (d, *J =* 8.6 Hz, 2H), 4.21 (s, 3H), 4.06 (s, 3H), 3.46–3.42 (m, 4H), 1.69–1.54 (m, 6H). ^13^C NMR (151 MHz, DMSO-*d*_6_) δ 171.4, 153.6, 153.6, 150.2, 143.2, 133.0, 130.8, 122.2, 114.7, 113.4, 108.7, 108.4, 106.7, 56.9, 47.5, 36.0, 25.1, 23.9. HPLC-MS (ESI): *m*/*z* calcd C_22_H_25_N_2_OS^+^ 365.17; [M]^+^ found 365.25.

#### 7-Fluoro-2-(4-(piperidin-1-yl)styryl)benzo[*d*]thiazole (**12a**)

The synthesis was carried out according to general procedure A and purified by flash chromatography (PE/EtOAc 1–20% B) to afford the product as a yellow solid (119 mg, 72%). *R_f_*: 0.49 (PE/EtOAc 4:1). ^1^H NMR (600 MHz, CDCl_3_) δ 7.75 (d, *J =* 8.1 Hz, 1H), 7.50–7.45 (m, 3H), 7.39 (td, *J =* 8.1, 5.4 Hz, 1H), 7.21 (d, *J =* 16.1 Hz, 1H), 7.05 (t, *J =* 8.6 Hz, 1H), 6.98–6.90 (m, 2H), 3.29 (t, *J =* 5.5 Hz, 4H), 1.71 (br s, 4H), 1.65–1.60 (m, 2H). ^13^C NMR (151 MHz, CDCl_3_) δ 169.0, 157.1 (d, *J*_C-F_*=* 248.7 Hz), 157.0 (d, *J*_C-F_*=* 2.7 Hz), 152.5, 138.9, 129.1, 127.1 (d, *J*_C-F_*=* 7.3 Hz), 125.3, 121.3 (d, *J*_C-F_*=* 16.6 Hz), 118.4 (d, *J*_C-F_*=* 3.5 Hz), 117.8, 115.6, 110.5 (d, *J*_C-F_*=* 18.9 Hz), 49.7, 25.5, 24.3. HPLC-MS (ESI): *m*/*z* calcd for C_20_H_19_FN_2_S 338.13; [M + H]^+^ found 339.15.

#### 7-Fluoro-3-methyl-2-(4-(piperidin-1-yl)styryl)benzo[*d*]thiazol-3-ium Iodide (**12b**)

The synthesis was carried out according to general procedure B (42 mg, 69%). *R_f_*: 0.19 (DCM/MeOH 5%). ^1^H NMR (600 MHz, DMSO-*d*_6_) δ 8.18 (d, *J =* 15.1 Hz, 1H), 7.96 (d, *J =* 8.4 Hz, 1H), 7.90 (d, *J =* 8.6 Hz, 2H), 7.83 (q, *J =* 8.0 Hz, 1H), 7.69–7.57 (m, 2H), 7.06 (d, *J =* 8.6 Hz, 2H), 4.22 (s, 3H), 3.54 (t, *J =* 5.4 Hz, 4H), 1.65 (q, *J =* 5.6 Hz, 2H), 1.63–1.53 (m, 4H). ^13^C NMR (151 MHz, DMSO-*d*_6_) δ 172.2, 156.3 (d, *J*_C-F_*=* 247.7 Hz), 154.4, 151.8, 144.9 (d, *J*_C-F_*=* 15.5 Hz), 134.1, 131.4 (d, *J*_C-F_*=* 7.5 Hz), 122.6, 114.4 (d, *J*_C-F_*=* 23.1 Hz), 113.9, 113.0, 109.5, 106.5, 48.0, 36.7, 25.7, 24.4. HPLC-MS (ESI): *m*/*z* calcd for C_21_H_22_FN_2_S^+^ 353.15; [M]^+^ found 353.10.

#### 2-(4-(Piperidin-1-yl)phenyl)benzo[*d*]thiazole (**13a**)

To a solution of **41** (140 mg, 0.48 mmol) in toluene (4.00 mL), Pd(PPh_3_)_4_ (28.0 mg, 0.02 mmol) and Cs_2_CO_3_ (140 mg, 0.72 mmol) were added under argon atmosphere. Piperidine (0.12 mL, 1.21 mmol) was diluted with toluene (1.00 mL) and slowly added. The mixture was refluxed for 5 h. It was diluted with water and extracted with EtOAc. The organic phase was dried over MgSO_4_, evaporated under reduced pressure, and purified by flash chromatography (PE/EtOAc 1–10% B) to afford the product (14.0 mg, 10%). *R_f_*: 0.50 (PE/EtOAc 5:1). ^1^H NMR (600 MHz, CDCl_3_) δ 8.01 (d, *J =* 8.1 Hz, 1H), 7.98 (d, *J =* 8.5 Hz, 2H), 7.85 (d, *J =* 7.9 Hz, 1H), 7.47–7.42 (m, 1H), 7.35–7.29 (m, 1H), 7.02 (br s, 2H), 3.34 (t, *J =* 5.5 Hz, 4H), 1.77–1.74 (m, 4H), 1.65 (p, *J =* 5.7 Hz, 2H). ^13^C NMR (151 MHz, CDCl_3_) δ 166.2, 154.2, 151.7, 134.7, 129.0, 128.3, 126.3, 124.7, 122.7, 121.7, 121.6, 52.7, 29.8, 25.3. HPLC-MS (ESI): *m*/*z* calcd for C_18_H_18_N_2_S 294.12; [M + H]^+^ found 295.05.

#### 3-Methyl-2-(4-(piperidin-1-yl)phenyl)benzo[*d*]thiazol-3-ium (**13b**)

To a solution of **13a** (16.0 mg, 0.05 mmol) in chlorobenzene (1.50 mL) was added methyl nosylate (14.0 mg, 0.06 mmol). The mixture was stirred overnight at 80 °C. The precipitate was filtered under vacuum and triturated in Et_2_O. The yellow solid was further purified by semipreparative HPLC (0.1% TFA in H_2_O/MeCN 20–60% B in 15 min) to isolate the product from the aniline *N*-methylated byproduct (10.0 mg, 59%). *R_f_*: 0.13 (DCM/MeOH 5%). ^1^H NMR (600 MHz, DMSO-*d*_6_) δ 8.40 (dd, *J =* 8.1, 1.2 Hz, 1H), 8.25 (d, *J =* 8.4 Hz, 1H), 7.89 (ddd, *J =* 8.5, 7.2, 1.2 Hz, 1H), 7.81 (dt, *J =* 9.1, 3.2 Hz, 2H), 7.78 (ddd, *J =* 8.2, 7.2, 1.0 Hz, 1H), 7.19 (dt, *J =* 9.1, 3.2 Hz, 2H), 4.25 (s, 3H), 3.55–3.53 (m, 4H), 1.72–1.64 (m, 2H), 1.64–1.57 (m, 4H). ^13^C NMR (151 MHz, DMSO-*d*_6_) δ 173.4, 153.8, 142.7, 132.5, 129.3, 128.1, 127.7, 123.9, 116.9, 113.5, 111.6, 47.4, 38.1, 24.9, 23.8. HPLC-MS (ESI): *m*/*z* calcd for C_19_H_21_N_2_S^+^ 309.14; [M]^+^ found 309.15.

#### 2-(4-(4-(Piperidin-1-yl)phenyl)buta-1,3-dien-1-yl)benzo[*d*]thiazole (**14a**)

The synthesis was carried out according to general procedure A (32 mg, 17%). *R_f_*: 0.48 (PE/EtOAc 4:1). ^1^H NMR (600 MHz, CDCl_3_) δ 7.90 (d, *J =* 8.1 Hz, 1H), 7.78 (d, *J =* 8.0 Hz, 1H), 7.39 (t, *J =* 7.7 Hz, 1H), 7.33 (d, *J =* 8.3 Hz, 2H), 7.29 (t, *J =* 7.5 Hz, 2H), 7.23 (d, *J =* 16.1 Hz, 1H), 6.84 (d, *J =* 7.7 Hz, 2H), 6.83–6.66 (m, 3H), 3.20 (t, *J =* 5.4 Hz, 4H), 1.64 (q, *J =* 5.8 Hz, 4H), 1.59–1.53 (m, 2H). ^13^C NMR (151 MHz, CDCl_3_) δ 167.6, 154.1, 152.2, 139.3, 138.3, 134.4, 128.4, 126.8, 126.3, 125.1, 124.1, 123.4, 122.7, 121.5, 115.6, 49.7, 25.7, 24.4. HPLC-MS (ESI): *m*/*z* calcd for C_22_H_22_N_2_S 346.15; [M + H]^+^ found 347.10.

#### 3-Methyl-2-(4-(4-(piperidin-1-yl)phenyl)buta-1,3-dien-1-yl)benzo[*d*]thiazol-3-ium Iodide (**14b**)

The synthesis was carried out according to general procedure B (109 mg, 87%). *R_f_*: 0.16 (DCM/MeOH 5%). ^1^H NMR (600 MHz, DMSO-*d*_6_) δ 8.33 (d, *J =* 8.1 Hz, 1H), 8.15 (d, *J =* 8.4 Hz, 1H), 7.99 (dd, *J =* 14.6, 10.9 Hz, 1H), 7.81 (ddd, *J =* 8.5, 7.2, 1.2 Hz, 1H), 7.71 (ddd, *J =* 8.1, 7.3, 1.0 Hz, 1H), 7.53 (d, *J =* 8.8 Hz, 2H), 7.43 (d, *J =* 15.1 Hz, 1H), 7.35 (d, *J =* 14.6 Hz, 1H), 7.21 (dd, *J =* 15.1, 10.9 Hz, 1H), 7.00 (d, *J =* 8.8 Hz, 2H), 4.16 (s, 3H), 3.39 (t, *J =* 5.1 Hz, 4H), 1.63–1.56 (m, 6H). ^13^C NMR (151 MHz, DMSO-*d*_6_) δ 170.6, 154.5, 152.4, 150.9, 147.4, 141.9, 130.4, 129.1, 127.8, 127.3, 124.0, 122.9, 116.3, 114.3, 112.9, 48.0, 35.7, 25.0, 24.0. HPLC-MS (ESI): *m*/*z* calcd for C_23_H_25_N_2_S^+^ 361.17; [M]^+^ found 361.20.

#### 4-(4-(2-(Benzo[*d*]thiazol-2-yl)vinyl)phenyl)morpholine (**15a**)

The synthesis was carried out according to general procedure A (47 mg, 59%). *R_f_*: 0.21 (Hept/EtOAc 3:1). ^1^H NMR (600 MHz, CDCl_3_) δ 7.96 (d, *J =* 8.1 Hz, 1H), 7.84 (d, *J =* 8.0 Hz, 1H), 7.51 (d, *J =* 8.5 Hz, 2H), 7.46 (d, *J =* 16.1 Hz, 1H), 7.45 (t, *J =* 7.4 Hz, 1H), 7.35 (t, *J =* 7.6 Hz, 1H), 7.26 (d, *J =* 16.1 Hz, 1H), 6.91 (d, *J =* 8.5 Hz, 2H), 3.87 (t, *J =* 4.9 Hz, 4H), 3.25 (t, *J =* 4.8 Hz, 4H).^13^C NMR (151 MHz, CDCl_3_) δ 167.8, 154.1, 152.1, 137.7, 134.4, 128.9, 126.8, 126.3, 125.1, 122.8, 121.6, 119.2, 115.2, 66.9, 48.5. HPLC-MS (ESI): *m*/*z* calcd for C_19_H_18_N_2_OS 322.11; [M + H]^+^ found 323.12.

#### 3-Methyl-2-(4-morpholinostyryl)benzo[*d*]thiazol-3-ium Iodide (**15b**)

The synthesis was carried out according to general procedure B (52 mg, 69%). *R_f_*: 0.13 (DCM/MeOH 5%). ^1^H NMR (600 MHz, DMSO-*d*_6_) δ 8.34 (dd, *J =* 8.2, 1.2 Hz, 1H), 8.14 (d, *J =* 8.3 Hz, 1H), 8.10 (d, *J =* 15.4 Hz, 1H), 7.95 (dt, *J =* 9.0, 2.9 Hz, 2H), 7.81 (ddd, *J =* 8.5, 7.2, 1.2 Hz, 1H), 7.74 (d, *J =* 15.4 Hz, 1H), 7.72 (ddd, *J =* 8.2, 7.2, 1.0 Hz, 1H), 7.09 (dt, *J =* 9.0, 3.1 Hz, 2H), 4.27 (s, 3H), 3.80–3.70 (m, 4H), 3.42 (dd, *J =* 5.7, 4.1 Hz, 4H). ^13^C NMR (151 MHz, DMSO-*d*_6_) δ 171.7, 153.8, 149.5, 142.0, 132.4, 129.0, 127.7, 127.1, 123.9, 123.5, 116.2, 113.6, 108.2, 65.8, 46.5, 35.8. HPLC-MS (ESI): *m*/*z* calcd for C_20_H_21_N_2_OS^+^ 337.14; [M]^+^ found 337.14.

#### 2-(4-Thiomorpholinostyryl)benzo[*d*]thiazole (**16a**)

The synthesis was carried out according to general procedure A (6 mg, 11%). *R_f_*: 0.39 (Hept/EtOAc 3:1). ^1^H NMR (600 MHz, CDCl_3_) δ 7.96 (d, *J =* 8.1 Hz, 1H), 7.84 (d, *J =* 7.9 Hz, 1H), 7.49 (d, *J =* 8.5 Hz, 2H), 7.47–7.42 (m, 2H), 7.35 (td, *J =* 8.0, 1.1 Hz, 1H), 7.25 (d, *J =* 16.1 Hz, 1H), 6.88 (d, *J =* 8.3 Hz, 2H), 3.77–3.67 (m, 4H), 2.74 (t, *J =* 5.1 Hz, 4H). ^13^C NMR (151 MHz, CDCl_3_) δ 167.9, 154.0, 151.2, 137.8, 134.3, 129.1, 126.4, 126.1, 125.1, 122.7, 121.6, 119.0, 116.0, 51.2, 26.3. HPLC-MS (ESI): *m*/*z* calcd for C_19_H_18_N_2_S_2_ 338.09; [M + H]^+^ found 339.07.

#### 3-Methyl-2-(4-thiomorpholinostyryl)benzo[*d*]thiazol-3-ium Iodide (**16b**)

The synthesis was carried out according to general procedure B (33 mg, 65%). *R_f_*: 0.13 (DCM/MeOH 5%). ^1^H NMR (600 MHz, DMSO-*d*_6_) δ 8.33 (dd, *J =* 8.1, 1.2 Hz, 1H), 8.13 (d, *J =* 8.4 Hz, 1H), 8.08 (d, *J =* 15.4 Hz, 1H), 7.97–7.89 (m, 2H), 7.81 (ddd, *J =* 8.5, 7.2, 1.2 Hz, 1H), 7.74–7.66 (m, 2H), 7.07 (d, *J =* 9.0 Hz, 2H), 4.26 (s, 3H), 3.94–3.84 (m, 4H), 2.72–2.63 (m, 4H). ^13^C NMR (151 MHz, DMSO-*d*_6_) δ 171.6, 152.3, 149.5, 142.0, 132.8, 128.9, 127.6, 127.0, 123.9, 122.7, 116.1, 113.9, 107.6, 49.5, 35.7, 25.2. HPLC-MS (ESI): *m*/*z* calcd for C_20_H_21_N_2_S_2_^+^ 353.11; [M]^+^ found 353.10.

#### 2-(4-(Pyrrolidin-1-yl)styryl)benzo[*d*]thiazole (**17a**)

The synthesis was carried out according to general procedure A (15 mg, 15%). *R_f_*: 0.50 (Hept/EtOAc 3:1). ^1^H NMR (600 MHz, CDCl_3_) δ 7.93 (d, *J =* 8.1 Hz, 1H), 7.82 (d, *J =* 7.9 Hz, 1H), 7.55–7.38 (m, 4H), 7.31 (t, *J =* 7.5 Hz, 1H), 7.18 (d, *J =* 16.1 Hz, 1H), 6.57 (d, *J =* 8.3 Hz, 2H), 3.35 (t, *J =* 6.1 Hz, 4H), 2.03 (hept, *J =* 3.4 Hz, 4H). ^13^C NMR (151 MHz, CDCl_3_) δ 168.6, 154.2, 148.9, 138.8, 134.2, 129.2, 126.2, 124.8, 122.8, 122.5, 121.5, 116.7, 112.0, 47.7, 25.6. HPLC-MS (ESI): *m*/*z* calcd for C_19_H_18_N_2_S 306.12; [M + H]^+^ found 307.05.

#### 3-Methyl-2-(4-(pyrrolidin-1-yl)styryl)benzo[*d*]thiazol-3-ium Iodide (**17b**)

The synthesis was carried out according to general procedure B (118 mg, 96%). *R_f_*: 0.16 (DCM/MeOH 5%). ^1^H NMR (400 MHz, DMSO-*d*_6_) δ 8.28 (d, *J =* 8.0 Hz, 1H), 8.12–7.99 (m, 2H), 7.90 (d, *J =* 8.6 Hz, 2H), 7.77 (t, *J =* 7.6 Hz, 1H), 7.67 (t, *J =* 7.6 Hz, 1H), 7.58 (d, *J =* 15.2 Hz, 1H), 6.69 (d, *J =* 8.6 Hz, 2H), 4.21 (s, 3H), 3.43–3.35 (m, 4H), 2.00 (p, *J =* 3.2 Hz, 4H). ^13^C NMR (101 MHz, DMSO-*d*_6_) δ 171.1, 151.0, 150.3, 141.9, 133.1, 128.8, 127.3, 126.7, 123.7, 121.3, 115.8, 112.4, 105.6, 47.6, 35.4, 24.8. HPLC-MS (ESI): *m*/*z* calcd for C_20_H_21_N_2_S^+^ 321.14; [M]^+^ found 321.14.

#### 2-(4-(4-Fluoropiperidin-1-yl)styryl)benzo[*d*]thiazole (**18a**)

The synthesis was carried out according to general procedure A (84 mg, 57%). *R_f_*: 0.23 (Hept/EtOAc 3:1). ^1^H NMR (400 MHz, DMSO-*d*_6_) δ 8.04 (dd, *J =* 7.9, 1.1 Hz, 1H), 7.91 (d, *J =* 8.1 Hz, 1H), 7.61 (d, *J =* 8.6 Hz, 2H), 7.54 (d, *J =* 16.1 Hz, 1H), 7.48 (td, *J =* 7.2, 1.0 Hz, 1H), 7.39 (td, *J =* 8.2, 0.9 Hz, 1H), 7.36 (d, *J =* 16.1 Hz, 1H), 7.00 (d, *J =* 8.4 Hz, 2H), 4.87 (dtt, *J =* 49.0, 7.3, 3.6 Hz, 1H), 3.58–3.42 (m, 2H), 3.27 (ddd, *J =* 12.6, 7.7, 3.8 Hz, 2H), 2.05–1.87 (m, 2H), 1.83–1.69 (m, 2H). ^13^C NMR (101 MHz, DMSO-*d*_6_) δ 167.7, 154.1, 151.5, 138.3, 134.2, 129.6, 126.8, 125.5, 125.3, 122.6, 122.5, 118.1, 115.3, 89.0 (d, *J*_C-F_*=* 169.4 Hz), 44.5 (d, *J*_C-F_*=* 6.9 Hz), 30.9 (d, *J*_C-F_*=* 19.1 Hz). HPLC-MS (ESI): *m*/*z* calcd for C_20_H_19_FN_2_S 338.13; [M + H]^+^ found 339.15.

#### 2-(4-(4-Fluoropiperidin-1-yl)styryl)-3-methylbenzo[*d*]thiazol-3-ium Iodide (**18b**)

The synthesis was carried out according to general procedure B (143 mg, 87%). *R_f_*: 0.19 (DCM/MeOH 5%). ^1^H NMR (400 MHz, DMSO-*d*_6_) δ 8.33 (dd, *J =* 8.1, 1.2 Hz, 1H), 8.13 (d, *J =* 8.4 Hz, 1H), 8.07 (d, *J =* 15.5 Hz, 1H), 7.92 (d, *J =* 8.7 Hz, 2H), 7.80 (td, *J =* 8.1, 7.2, 1.3 Hz, 1H), 7.75–7.64 (m, 2H), 7.11 (d, *J =* 8.7 Hz, 2H), 4.93 (dtt, *J =* 48.9, 7.1, 3.5 Hz, 1H), 4.26 (s, 3H), 3.76–3.58 (m, 2H), 3.55–3.44 (m, 2H), 1.98 (dddd, *J =* 24.8, 16.1, 7.5, 3.6 Hz, 2H), 1.78 (dtt, *J =* 14.1, 7.2, 3.8 Hz, 2H). ^13^C NMR (101 MHz, DMSO-*d*_6_) δ 172.1, 153.5, 150.0, 142.4, 133.2, 129.4, 128.1, 127.5, 124.4, 123.3, 116.6, 114.3, 108.1, 88.7 (d, *J*_C-F_*=* 169.5 Hz), 43.6 (d, *J*_C-F_*=* 6.6 Hz), 36.2, 31.0 (d, *J*_C-F_*=* 19.4 Hz). ^19^F NMR (376 MHz, DMSO-*d*_6_) δ -177.5. HPLC-MS (ESI): *m*/*z* calcd for C_21_H_22_FN_2_S^+^ 353.15; [M]^+^ found 353.14.

#### 4-(2-(Benzo[*d*]thiazol-2-yl)vinyl)-*N*,*N*-dimethylaniline (**19a**)

The synthesis was carried out according to general procedure A (58 mg, 34%). *R_f_*: 0.40 (Hept/EtOAc 3:1). ^1^H NMR (600 MHz, CDCl_3_) δ 7.94 (dt, *J =* 8.0, 0.9 Hz, 1H), 7.82 (dt, *J =* 7.7, 0.9 Hz, 1H), 7.48 (dt, *J =* 8.8, 2.8 Hz, 2H), 7.45 (d, *J =* 16.1 Hz, 1H), 7.43 (ddd, *J =* 8.3, 7.2, 1.2 Hz, 1H), 7.32 (ddd, *J =* 8.2, 7.2, 1.2 Hz, 1H), 7.21 (d, *J =* 16.1 Hz, 1H), 6.72 (dt, *J =* 8.9, 2.9 Hz, 2H), 3.03 (s, 6H). ^13^C NMR (151 MHz, CDCl_3_) δ 168.4, 154.2, 151.4, 138.5, 134.3, 129.1, 126.2, 124.9, 123.5, 122.6, 121.5, 117.4, 112.2, 40.4. HPLC-MS (ESI): *m*/*z* calcd for C_17_H_16_N_2_S 280.10; [M + H]^+^ found 281.11.

#### 2-(4-(Dimethylamino)styryl)-3-methylbenzo[*d*]thiazol-3-ium Iodide (**19b**)

The synthesis was carried out according to general procedure B (125 mg, 90%). *R_f_*: 0.14 (DCM/MeOH 5%). ^1^H NMR (400 MHz, DMSO-*d*_6_) δ 8.30 (dd, *J =* 8.1, 1.2 Hz, 1H), 8.15–7.99 (m, 2H), 7.91 (d, *J =* 8.9 Hz, 2H), 7.78 (ddd, *J =* 8.5, 7.2, 1.2 Hz, 1H), 7.72–7.65 (m, 1H), 7.62 (d, *J =* 15.3 Hz, 1H), 6.84 (d, *J =* 8.9 Hz, 2H), 4.23 (s, 3H), 3.10 (s, 6H). ^13^C NMR (101 MHz, DMSO-*d*_6_) δ 171.3, 153.5, 150.1, 141.9, 132.8, 128.8, 127.4, 126.8, 123.8, 121.4, 115.9, 111.9, 106.2, 39.8, 35.5. HPLC-MS (ESI): *m*/*z* calcd for C_18_H_19_N_2_S^+^ 295.13; [M]^+^ found 295.11.

#### 2-(4-(Piperidin-1-yl)styryl)benzo[*d*]thiazole (**20**)

The synthesis was carried out according to general procedure A (1.14 g, 53%). *R_f_*: 0.38 (Hept/EtOAc 3:1). ^1^H NMR (400 MHz, CDCl_3_) δ 7.95 (d, *J =* 8.1 Hz, 1H), 7.83 (d, *J =* 7.9 Hz, 1H), 7.53–7.39 (m, 4H), 7.33 (t, *J =* 7.6 Hz, 1H), 7.23 (d, *J =* 16.2 Hz, 1H), 6.91 (d, *J =* 8.5 Hz, 2H), 3.28 (t, *J =* 5.6 Hz, 4H), 1.70 (p, *J =* 5.6 Hz, 4H), 1.63 (q, *J =* 6.5, 5.6 Hz, 2H). ^13^C NMR (101 MHz, CDCl_3_) δ 168.1, 154.2, 152.6, 138.1, 134.3, 128.9, 126.3, 125.4, 125.0, 122.7, 121.5, 118.4, 115.4, 49.5, 25.7, 24.5. HPLC-MS (ESI): *m*/*z* calcd for C_20_H_20_N_2_S 320.13; [M + H]^+^ found 321.13.

#### 3-Methyl-2-(4-(piperidin-1-yl)styryl)benzo[*d*]thiazol-3-ium Iodide (**RB1**)

The synthesis was carried out according to general procedure B (322 mg, 81%). *R_f_*: 0.17 (DCM/MeOH 5%). ^1^H NMR (400 MHz, DMSO-*d*_6_) δ 8.31 (dd, *J =* 8.2, 1.2 Hz, 1H), 8.11 (d, *J =* 8.4 Hz, 1H), 8.05 (d, *J =* 16.1 Hz, 1H), 7.90 (d, *J =* 9.0 Hz, 2H), 7.79 (ddd, *J =* 8.5, 7.3, 1.3 Hz, 1H), 7.73–7.62 (m, 2H), 7.05 (d, *J =* 8.7 Hz, 2H), 4.24 (s, 3H), 3.50 (t, *J =* 5.1 Hz, 4H), 1.69–1.51 (m, 6H). ^13^C NMR (101 MHz, DMSO-*d*_6_) δ 171.9, 154.1, 150.2, 142.4, 133.4, 129.4, 128.0, 127.4, 124.3, 122.7, 116.5, 113.9, 107.4, 48.0, 36.1, 25.6, 24.4. HPLC-MS (ESI): *m*/*z* calcd for C_21_H_23_N_2_S^+^ 335.16; [M]^+^ found 335.17.

#### 3-Methyl-2-(4-(4-methylpiperazin-1-yl)styryl)benzo[*d*]thiazol-3-ium Iodide (**RB2**)

The synthesis was carried out according to general procedure B (159 mg, 97%). *R_f_*: 0.19 (DCM/MeOH 5%). ^1^H NMR (400 MHz, DMSO-*d*_6_) δ 8.35 (d, *J =* 7.9 Hz, 1H), 8.15 (d, *J =* 8.4 Hz, 1H), 8.10 (d, *J =* 15.5 Hz, 1H), 7.95 (d, *J =* 8.5 Hz, 2H), 7.82 (t, *J =* 7.9 Hz, 1H), 7.75 (d, *J =* 15.5 Hz, 1H), 7.72 (t, *J =* 7.7 Hz, 1H), 7.11 (d, *J =* 8.5 Hz, 2H), 4.28 (s, 3H), 3.56 (br s, 4H), 2.85 (br s, 4H), 2.53 (s, 3H). ^13^C NMR (101 MHz, DMSO-*d*_6_) δ 171.7, 153.0, 149.3, 141.9, 132.4, 129.0, 127.7, 127.1, 123.9, 123.7, 116.2, 114.0, 108.4, 53.3, 45.2, 44.0, 35.9. HPLC-MS (ESI): *m*/*z* calcd for C_21_H_24_N_3_S^+^ 350.17; [M + H]^+^ found 350.15.

#### 6-Fluoro-2-(4-(4-(piperidin-1-yl)phenyl)buta-1,3-dien-1-yl)benzo[*d*]thiazole (**43**)

The synthesis was carried out according to general procedure A (37 mg, 28%). *R_f_*: 0.47 (PE/EtOAc 4:1). ^1^H NMR (600 MHz, CDCl_3_) δ 7.90 (dd, *J =* 8.9, 4.8 Hz, 1H), 7.52 (dd, *J =* 7.1, 1.6 Hz, 1H), 7.40 (d, *J =* 8.3 Hz, 2H), 7.33–7.27 (m, 1H), 7.19 (t, *J =* 8.7 Hz, 1H), 6.92 (d, *J =* 8.3 Hz, 2H), 6.88–6.80 (m, 3H), 3.27 (t, *J =* 5.4 Hz, 4H), 1.81–1.68 (m, 4H), 1.64 (p, *J =* 5.7 Hz, 2H). ^13^C NMR (151 MHz, CDCl_3_) δ 167.3, 160.5 (d, *J*_C-F_*=* 245.6 Hz), 152.1, 150.8 (d, *J*_C-F_*=* 1.5 Hz), 139.3, 138.4, 135.4 (d, *J*_C-F_*=* 11.2 Hz), 128.4, 126.9, 124.0, 123.5 (d, *J*_C-F_*=* 9.3 Hz), 123.1, 115.7, 114.8 (d, *J*_C-F_*=* 24.8 Hz), 107.8 (d, *J*_C-F_*=* 27.0 Hz), 49.8, 25.6, 24.4. HPLC-MS (ESI): *m*/*z* calcd for C_22_H_21_FN_2_S 364.14; [M + H]^+^ found 365.10.

#### 6-Fluoro-2-(4-(4-(pyrrolidin-1-yl)phenyl)buta-1,3-dien-1-yl)benzo[*d*]thiazole (**44**)

The synthesis was carried out according to general procedure A (24 mg, 36%). *R_f_*: 0.51 (PE/EtOAc 4:1). ^1^H NMR (600 MHz, CDCl_3_) δ 7.92–7.77 (m, 1H), 7.46 (d, *J =* 8.3 Hz, 1H), 7.34 (d, *J =* 8.1 Hz, 2H), 7.25–7.20 (m, 1H), 7.13 (t, *J =* 9.1 Hz, 1H), 6.93–6.68 (m, 3H), 6.52 (d, *J =* 8.8 Hz, 2H), 3.31 (s, 4H), 2.00 (s, 4H). ^13^C NMR (151 MHz, CDCl_3_) δ 167.7, 162.1 (d, *J*_C-F_*=* 239.1 Hz), 150.5, 148.3, 140.1, 139.4, 137.2, 128.8, 127.8, 123.3 (d, *J*_C-F_*=* 8.4 Hz), 122.4, 121.9, 114.8 (d, *J*_C-F_*=* 24.0 Hz), 112.1, 107.8 (d, *J*_C-F_*=* 26.8 Hz), 47.9, 25.6. HPLC-MS (ESI): *m*/*z* calcd for C_21_H_19_FN_2_S 350.13; [M + H]^+^ found 351.15.

#### 4-(4-(2-(6-Fluorobenzo[*d*]thiazol-2-yl)vinyl)phenyl)morpholine (**45**–MFSB)

The synthesis was carried out according to general procedure A (21 mg, 69%). *R_f_*: 0.18 (PE/EtOAc 4:1). ^1^H NMR (600 MHz, CDCl_3_) δ 7.90 (dd, *J =* 9.0, 4.8 Hz, 1H), 7.58–7.47 (m, 3H), 7.43 (d, *J =* 16.0 Hz, 1H), 7.24 (d, *J =* 16.0 Hz, 1H), 7.19 (td, *J =* 8.9, 2.6 Hz, 1H), 6.96 (d, *J =* 8.2 Hz, 2H), 3.90 (t, *J =* 4.6 Hz, 4H), 3.27 (t, *J =* 4.2 Hz, 4H). ^13^C NMR (151 MHz, CDCl_3_) δ 167.6, 160.6 (d, *J*_C-F_*=* 245.5 Hz), 151.8, 150.4, 138.0, 135.2, 129.0, 127.0, 123.5 (d, *J*_C-F_*=* 9.0 Hz), 118.9, 115.4, 115.0 (d, *J*_C-F_*=* 25.1 Hz), 107.9 (d, *J*_C-F_*=* 27.1 Hz), 66.7, 48.7. HPLC-MS (ESI): *m*/*z* calcd for C_19_H_17_FN_2_OS 340.10; [M + H]^+^ found 341.10.

#### 6-Bromo-2-(4-(piperidin-1-yl)styryl)benzo[*d*]thiazole (**46**)

The synthesis was carried out according to general procedure A (2.80 g, 80%). *R_f_*: 0.51 (PE/EtOAc 4:1). ^1^H NMR (600 MHz, CDCl_3_) δ 7.94 (s, 1H), 7.78 (d, *J =* 8.6 Hz, 1H), 7.52 (d, *J =* 8.6 Hz, 1H), 7.46 (d, *J =* 8.3 Hz, 2H), 7.43 (d, *J =* 16.0 Hz, 1H), 7.18 (d, *J =* 16.0 Hz, 1H), 6.92 (br s, 2H), 3.29 (t, *J =* 5.4 Hz, 4H), 1.70 (br s, 4H), 1.63 (q, *J =* 5.7 Hz, 2H). ^13^C NMR (151 MHz, CDCl_3_) δ 168.6, 153.0, 152.5, 138.7, 136.0, 129.7, 129.0, 125.3, 124.0, 123.7, 118.4, 117.9, 115.5, 49.6, 25.5, 24.4. HPLC-MS (ESI): *m*/*z* calcd for C_20_H_19_BrN_2_S 398.05; [M + H]^+^ found 399.00, 400.95.

#### 4-(4-(2-(6-Bromobenzo[*d*]thiazol-2-yl)vinyl)phenyl)morpholine (**47**)

The synthesis was carried out according to general procedure A (639 mg, 73%). *R_f_*: 0.15 (PE/EtOAc 4:1). ^1^H NMR (600 MHz, CDCl_3_) δ 7.97 (d, *J =* 1.9 Hz, 1H), 7.82 (d, *J =* 8.6 Hz, 1H), 7.55 (dd, *J =* 8.6, 1.9 Hz, 1H), 7.53 (dt, *J =* 8.7, 2.0 Hz, 2H), 7.48 (d, *J =* 16.1 Hz, 1H), 7.25 (d, *J =* 16.1 Hz, 1H), 7.00 (d, *J =* 8.4 Hz, 2H), 3.92 (t, *J =* 4.8 Hz, 4H), 3.30–3.26 (m, 4H). ^13^C NMR (151 MHz, CDCl_3_) δ 168.3, 151.9, 151.0, 138.7, 135.6, 130.1, 129.3, 126.2, 124.2, 123.6, 118.9, 118.7, 116.0, 66.4, 49.2. HPLC-MS (ESI): *m*/*z* calcd for C_19_H_17_BrN_2_OS 400.02; [M + H]^+^ found 400.95, 403.05.

#### 2-(4-(Piperidin-1-yl)styryl)-6-(4,4,5,5-tetramethyl-1,3,2-dioxaborolan-2-yl)benzo[*d*]thiazole (**48**)

Pd(dppf)Cl_2_ (303 mg, 0.40 mmol) was added to a solution of **46** (1.60 g, 4.01 mmol), potassium acetate (786 mg, 8.01 mmol), and bis(pinacolato)diboron (1.53 g, 6.01 mmol) in dry DMF (34.0 mL). The mixture was heated at 100 °C for 45 min. It was poured into water and extracted with EtOAc. The organic phase was dried over MgSO_4_, evaporated under reduced pressure, and purified by flash chromatography (PE/EtOAc 5–20% B) to afford the product as a yellow solid (1.66 g, 93%). *R_f_*: 0.35 (PE/EtOAc 6:1). ^1^H NMR (600 MHz, CDCl_3_) δ 8.31 (d, *J =* 1.1 Hz, 1H), 7.93 (d, *J =* 8.1 Hz, 1H), 7.87 (dd, *J =* 8.1, 1.1 Hz, 1H), 7.49–7.44 (m, 3H), 7.23 (d, *J =* 16.1 Hz, 1H), 6.90 (d, *J =* 8.4 Hz, 2H), 3.29–3.23 (m, 4H), 1.68 (p, *J =* 5.5 Hz, 4H), 1.60 (q, *J =* 7.0, 6.4 Hz, 2H), 1.37 (s, 12H). ^13^C NMR (151 MHz, CDCl_3_) δ 169.6, 156.1, 152.4, 138.5, 133.8, 132.3, 129.0, 129.0, 128.4, 125.4, 121.8, 118.4, 115.4, 84.1, 49.5, 25.5, 25.0, 24.3. HPLC-MS (ESI): *m*/*z* calcd for C_26_H_31_BN_2_O_2_S 446.22; [M + H]^+^ found 447.30.

#### 4-(4-(2-(6-(4,4,5,5-Tetramethyl-1,3,2-dioxaborolan-2-yl)benzo[*d*]thiazol-2-yl)vinyl)phenyl)morpholine (**49**)

The synthesis was carried out following the same procedure as **48** (124 mg, 18%). *R_f_*: 0.17 (PE/EtOAc 3:1). ^1^H NMR (600 MHz, CDCl_3_) δ 8.31 (d, *J =* 1.1 Hz, 1H), 7.94 (d, *J =* 8.1 Hz, 1H), 7.87 (dd, *J =* 8.1, 1.1 Hz, 1H), 7.49 (d, *J =* 8.7 Hz, 2H), 7.48 (d, *J =* 16.1 Hz, 1H), 7.26 (d, *J =* 16.1 Hz, 1H), 6.89 (dt, *J =* 8.8, 2.9 Hz, 2H), 3.87–3.84 (m, 4H), 3.25–3.21 (m, 4H), 1.36 (s, 12H). ^13^C NMR (151 MHz, CDCl_3_) δ 169.5, 155.6, 152.0, 138.5, 133.6, 132.4, 130.7, 129.0, 128.5, 126.6, 121.8, 118.8, 115.1, 84.2, 66.7, 48.3, 25.0. HPLC-MS (ESI): *m*/*z* calcd for C_25_H_29_BN_2_O_3_S 448.20; [M + H]^+^ found 448.95.

### Radiochemistry

#### Manual Synthesis of [^18^F]PFSB

[^18^F]Fluoride was produced using a PETtrace 890 cyclotron (GE Healthcare, Uppsala, Sweden) and delivered as a target wash in H_2_O. A Sep-Pak Plus Light QMA carb cartridge was conditioned with a sequence of: 10 mL of KOTf aq (90 mg/mL), 10 mL of air, 10 mL of H_2_O, 10 mL of air. [^18^F]Fluoride was trapped onto the QMA cartridge, dried with argon (10 mL passed through the cartridge), and eluted with a solution of TBAOTf in MeOH (10 mg/1 mL) to afford [^18^F]TBAF. The resulting solution was aliquoted in 4 reaction vials, and MeOH was evaporated at 90 °C.

A stock solution of Cu(OTf)_2_ in DMA (100 mg/mL) was prepared. To prepare each reaction mixture, 25.5 μL of the stock solution was diluted in the chosen amount of DMA (510 μL for a and b, 499 μL for c and d) and *n*-BuOH (60.0 μL) and pyridine (4.90 μL, 60 μmol or 16.0 μL, 200 μmol; Table S1) were added. The solution was added to the precursor **48** (9.00 mg, 20.2 μmol or 4.50 mg, 10.1 μmol; Table S1). The mixture was sonicated, added to the corresponding reaction vial, and heated at 120 °C for 20 min. The reaction was quenched with 1 mL of 0.1 M HCl and neutralized with 1 mL of 0.1 M NaOH. 500 μL of MeCN was added to each mixture to avoid product precipitation. Reaction performance was evaluated by radioTLC (PE/EtOAc 2:1) and radioHPLC. Analytical HPLC conditions and chromatograms are reported in the Supporting Information.

#### Automated Synthesis of [^18^F]PFSB and [^18^F]MFSB

A Sep-Pak Plus Light QMA carb cartridge was conditioned with a sequence of: 10 mL of KOTf aq (90 mg/mL), 10 mL of air, 10 mL of H_2_O, 10 mL of air. A Sep-Pak Plus Light Alum N cartridge was prepared with 5 mL of H_2_O. A Sep-Pak Plus tC18 and a Sep-Pak Light C18 were conditioned with 10 mL of EtOH and 10 mL of H_2_O each. To prepare the reaction mixture, Cu(OTf)_2_ (2.40 mg, 6.72 μmol) was dissolved in 535.7 μL of DMA. *n*-BuOH (60.0 μL) and pyridine (4.30 μL, 53.8 μmol) were added. The solution was added to the **PFSB** precursor **48** (4.00 mg, 8.96 μmol) or the **MFSB** precursor **49** (4.00 mg, 8.92 μmol) and sonicated.

[^18^F]Fluoride was produced by a PETtrace 890 cyclotron (GE Healthcare, Uppsala, Sweden) and delivered into an FX N Pro module (GE Healthcare, Münster, Germany). It was trapped onto the QMA cartridge, eluted into the reactor with a solution of TBAOTf in MeOH (10 mg/1 mL), and the solvent evaporated at 90 °C. The reaction mixture was added, and the reactor was heated to 120 °C for 20 min. The resulting mixture was diluted with 10 mL of MeCN/ammonium formate buffer (25 mM, pH 8) 1:1 v/v and trapped on a stack of Alox and tC18 cartridges. The product was eluted with MeCN (3.4 mL for [^18^F]PFSB; 2.8 mL for [^18^F]MFSB) into tube 2, which was equipped with 25 mM ammonium formate (1.6 mL for [^18^F]PFSB; 2.2 mL for [^18^F]MFSB). The mixture was injected into the HPLC loop. Semipreparative HPLC conditions for purification: Luna 5 μm C8 (2) 100 Å 250 mm × 10 mm; 68% MeCN in 25 mM ammonium formate at pH 8 (retention time ≈ 17 min) for [^18^F]PFSB; 55% MeCN in 25 mM ammonium formate at pH 8 (retention time ≈ 12 min) for [^18^F]MFSB; 6 mL/min.

The product peak was cut, diluted with water (55 mL), and trapped onto a C18 cartridge. It was washed with water (5 mL), eluted with EtOH (0.5 mL), formulated with phosphate-buffered saline (PBS) (4.5 mL), and transferred into the product vial. Quality control (QC) was performed (analytical HPLC conditions and chromatograms are reported in the Supporting Information).

#### Tritium-Labeling of [^3^H]PiB and [^3^H]MODAG-001

PiB and MODAG-001 were tritiated by RC Tritec AG (Teufen, Switzerland), dissolved in EtOH, and stored at −80 °C until use. Radiochemical purities of >99% were achieved for both radioligands, and the molar activities (*A*_m_) of [^3^H]PiB and [^3^H]MODAG-001 were 21.7 and 78.9 Ci/mmol, respectively.

### Biological Evaluation

#### Calculation of BBB Score and CNS MPO

All required properties (cLog *P*, cLog *D*, TPSA, molecular weight, p*K*_a_) were calculated via Chemicalize (ChemAxon, Budapest, Hungary) and entered into the calculation Excel tables provided in the literature.^15,16^ All values are reported in the Supporting Information (Table S2).

#### Preparation of αSYN and Aβ_1-42_ Fibrils

Following the cloning of the DNA construct encoding full-length αSYN into the pET-22b vector and its transformation into BL21 cells, the expression of human αSYN from a prokaryotic host was induced overnight in *Escherichia coli* at 20 °C with 0.5 mM IPTG. The cells were harvested by centrifugation, resuspended in 20 mM Tris-HCl pH 7.6, 25 mM NaCl and 1× complete protease inhibitor (Roche, Basel, Switzerland), and lysed by sonication. Additionally, they were boiled for 15 min and centrifuged at 12 000*g* for 30 min. Anion exchange chromatography (HiTrap Q, GE Healthcare, Chicago, Illinois) was used to trap the produced αSYN, which was eluted with NaCl 0.1 M over 20 column volumes. Fractions containing αSYN were concentrated and further purified through a Superdex 75 SEC column equilibrated with PBS. Additional purification was conducted via a high-capacity endotoxin removal spin column (Pierce, Waltham, Massachusetts), affording an endotoxin level below 1.0 EU/mg. The monomeric state and monodispersity of pure αSYN were confirmed by dynamic light scattering and fibrillation was performed according to the protocol from Makky et al.^26^ A 4 mg/mL solution of αSYN in 20 mM K_3_PO_4_ pH 9.1 was placed in an orbital shaker at 37 °C, 1000 rpm for 5 days to prepare P91s. The assemblies were either sonicated with cycles of 20 s on and 10 s off at 50% amplitude (Q800R3, Qsonica) for a total of 10 min or directly aliquoted in volumes of 10 μL each stored at −80 °C until use. The method for Aβ_1-42_ fibrils generation was adapted from Bagchi et al. and described in Kuebler et al.^17,27^ Synthetic lyophilized human Aβ_1-42_ peptide (5 mg) with >90% purity (EMC Microcollections, Tuebingen, Germany) was dissolved in DMSO (221.5 μL), followed by the addition of deionized water (4.1 mL) and 1 M Tris-HCl (111 μL, pH 7.6) to reach a final monomeric concentration of 250 μM. Aggregation was induced by incubation in an Eppendorf Thermomixer at 37 °C with shaking at 800 rpm for 72 h. The resulting fibrils were sonicated for 3 min in a water bath (Elmasonic S 60 H, Elma Schmidbauer GmbH, Singen, Germany). The final products were aliquoted, frozen on dry ice, and stored at −80 °C until use. The fibril batch underwent quality control via ThT fluorescence and binding assays with nonradioactive PiB competing with [^3^H]PiB and MODAG-001 (Figure S6).

#### Fibril Binding Assays

The *K*_d_ values of [^3^H]PiB and [^3^H]MODAG-001 were determined via saturation binding assays on human recombinant αSYN (180 nM for [^3^H]PiB, 50 nM for [^3^H]MODAG-001) and synthetic human Aβ_1-42_ fibrils (2 μM for [^3^H]PiB, 1 μM for [^3^H]MODAG-001) diluted in phosphate-buffered saline (PBS; Gibco DPBS, no calcium, no magnesium, Thermo Fisher Scientific, Waltham, MA). The fibrils were incubated in 96-well micro test low-binding plates (Ratiolab GmbH, Dreieich, Germany) with increasing concentrations of [^3^H]PiB (up to 56 nM) and [^3^H]MODAG-001 (up to 36 nM) in 30 mM Tris-HCl, 0.1% bovine serum albumin, 0.05% Tween20 in a total volume of 200 μL/well. For blocking, the tracers were incubated with the corresponding nonradioactive compound (1.5 μM PiB or 0.5 μM MODAG-001). The stock solutions of PiB and MODAG-001 were prepared by dissolving the compounds in DMSO to 1 mM, which yielded a DMSO concentration of ≤0.15% in the final assay.

The binding affinity (*K_i_* values) of the newly developed 2-styrylbenzothiazoles was determined via competition binding assays against [^3^H]PiB and [^3^H]MODAG-001. The test compounds were dissolved in DMSO to a stock concentration of 1 mM, which resulted in ≤1% DMSO concentration in the final assay. Increasing concentrations of the test compounds (0.6 nM–10 μM) competed against 6 nM [^3^H]PiB and 1 nM [^3^H]MODAG-001. Concentrations of αSYN and Aβ_1-42_ fibrils were as described above.

Plates were incubated on a shaker (MaxQ 6000, Thermo Fisher Scientific, Inc., Marietta, OH) at 50 rpm for 2 h at 37 °C, covered by removable sealing tapes (PerkinElmer, Waltham, MA). Vacuum filtration and read-out were performed as previously reported.^17^ Radioactivity was plotted against increasing concentrations of [^3^H]PiB, [^3^H]MODAG-001, or the nonradioactive test compounds. Data points were fitted using nonlinear regression analysis in GraphPad Prism (GraphPad Software, Inc., Version 8.4.0, La Jolla).

#### Autoradiography and Immunohistochemistry

*Post-mortem* human brain slices (10 μm thickness) were obtained from the subject cases and were analyzed by the Neurobiobank München (NBM, Munich, Germany), where tissues were collected on the basis of written informed consent according to the guidelines of the ethics committee of the Ludwig Maximilians University of Munich, Germany (# 345-13). The use of brain tissue samples in this study was approved by the ethics committee of the Faculty of Medicine at the University of Tuebingen (Ethics approval number: 813/2018BO2). Table S4 (Supporting Information) summarizes the information of the subjects from which samples were obtained and used in the experiment.

For autoradiography, frozen brain slices were thawed for 1 h before preincubation in bovine serum albumin (BSA) buffer for 25 min at room temperature. To determine total binding (TB), brain slices were incubated with the tracer (10 nM [^18^F]PFSB, 10 nM [^18^F]MFSB). Incubation of consecutive slices with the corresponding nonradioactive compounds (10 μM PFSB, 10 μM MFSB) was performed to determine nonspecific binding (NSB). The nonradioactive compounds were prepared by dissolving in DMSO to 10 mM, which yielded a DMSO concentration of ≤0.1% in the final solution. Incubation was carried out for 1 hour at room temperature with subsequent washing in cold BSA buffer (3 × 10 min) followed by three dippings in cold deionized water. After drying under an IR lamp, brain slices were exposed to a storage phosphor screen (Molecular Dynamics, Caesarea, Israel) for 18 h, which was then scanned in a phosphor imager (STORM 840, Molecular Dynamics, Sunnyvale, CA).

Quantitative data analysis was performed by drawing four regions of interest (ROIs) in relevant areas of the slice and one ROI next to it for background subtraction (Figures S7 and S8; ImageJ 1.8.0_172, National Institute of Health, Bethesda, MD).^28^ Specific binding (SB) is obtained by subtracting NSB from TB. The SB disease/SB control ratio was calculated by dividing by the SB in diseased tissues with the respective SB in control tissues.

After autoradiography procedures, brain slices were stored at −20 °C. IHC was performed on the same tissue slices which were used for TB in autoradiography. After thawing, the slices were post-fixed in 4.5% paraformaldehyde (PFA, SAV Liquid Production GmbH, Flintsbach am Inn, Germany) for 20 min at room temperature. Following washing steps in PBS (2 × 5 min), antigen retrieval was performed. For αSYN pSer129 staining, sodium citrate buffer (10 mM, pH 6, Sigma-Aldrich Chemie GmbH, Darmstadt, Germany) was boiled and the brain slices were incubated at room temperature in the boiled buffer for 30 min; brain sections to be stained for Aβ were incubated in 97% formic acid for 10 min at room temperature. After washing, quenching was performed for 20 min (1 mL quenching solution = 890 μL tris-buffered saline (TBS), 100 μL MeOH, and 10 μL 30% H_2_O_2_). Brain slices were subsequently washed and equilibrated in TBS (2 × 5 min) and TBS supplemented with 0.1% Triton X-100 and 1% BSA (in the following referred to as TBS-X) (1 × 5 min). Blocking in TBS supplemented with 0.3% Triton X-100 and 10% normal goat serum for 60 min at room temperature was performed. Incubation with primary antibody was carried out overnight at 4 °C with either mouse anti-phosphorylated αSYN pSer129 monoclonal antibody (1:5000 in TBS-X, clone pSyn#64; 015-25191, FUJIFILM Wako Chemicals Europe GmbH, Neuss, Germany) or mouse anti-β-amyloid 17–24 antibody (1:6000 in TBS-X, clone 4G8, 800708, BioLegend, Amsterdam, The Netherlands).

On the second day, the brain slices were washed with TBS-X (3 × 10 min) and incubated with secondary antibody (EnVision+/HRP Dual Link Rabbit/Mouse, K406189-2, Agilent, Waldbronn, Germany) for 30 min at room temperature. After washing in TBS-X (2 × 10 min) and TBS (1 × 10 min), the samples were incubated with 3,3′-diaminobenzidine (1:50, Agilent, Waldbronn, Germany) for 10 min. After washing with deionized water (2 × 5 min), the brain slices were incubated in hematoxylin (Merck KGaA, Darmstadt, Germany) for 45 s and then rinsed with running tap water for 10 min. To dehydrate and clear the tissues, the samples were washed in 70% EtOH (1 min), 95% EtOH (2 × 1 min), 100% EtOH (2 × 1 min), and xylene (2 × 2 min). The slides were mounted with Eukitt quick-hardening mounting medium (Fluka Analytical, Munich, Germany). The stained tissues were scanned with NanoZoomer 2.0 HT (Hamamatsu Photonics K.K., Hamamatsu, Japan) at 40× magnification.

#### *In Vivo* PET/MR Imaging

The animal experiments were performed in compliance with the European directives on the protection and use of laboratory animals (Council Directive 2010/63/UE) and the German animal welfare act with approval from the local authorities (Regierungspräsidium Tuebingen, R3/19G). Healthy wild-type female C57BL/6J mice (20.3 ± 0.9 g; 9 weeks old) purchased from Charles River Laboratories (Sulzbach, Germany) were maintained in our vivarium on a 12:12 hour light–dark cycle, and were kept at a temperature of 22 °C with 40–60% humidity and free access to a standard diet and tap water.

The mice (*n* = 3) were anesthetized with 1.5% isoflurane evaporated in 100% oxygen at a flow rate of 0.8 L/min. A body temperature of 37 °C was maintained using a feedback temperature control unit. PET imaging studies were performed on Inveon dedicated microPET system (Inveon D-PET, Siemens, Knoxville, TN). Five seconds after the start of PET acquisition, the mice were injected intravenously with 9.9 ± 0.6 MBq of [^18^F]MFSB (*A*_m_ = 46.2 ± 2.5 GBq/μmol at the time of injection). The 1 h dynamic acquisitions were divided into 39 time frames (12 × 5 s, 6 × 10 s, 6 × 30 s, 5 × 60 s, and 10 × 300 s). A 13-min transmission measurement with a cobalt-57 point source was performed for attenuation correction. Subsequently, an anatomical MR scan with a 7 Tesla MR scanner (ClinScan, Bruker BioSpin MRI GmbH, Ettlingen, Germany) using a rat whole-body volume coil and the Paravision software (v6.0.1, Bruker, Ettlingen, Germany) using a T2-weighted Turbo-RARE MRI sequence.

The PET image was reconstructed via the OSEM3D/SP-MAP reconstruction algorithm and coregistered to the whole-body MRI scan. Volumes of interest (VOIs) were hand-drawn for the relevant organs based on the MR anatomy in PMOD (PMOD Technologies, Faellanden, Switzerland, Version 4.2) and VOIs at different regions in the mouse brain were extracted using the atlas provided by PMOD to calculate the respective time–activity curves (TACs). Standardized uptake values (SUVs) were calculated as a ratio of the detected activity with injected activity and weight (SUV = (TAC (kBq/cc)/inj. activity (kBq)) × weight (g)).

## Supporting Information Available

Synthetic procedures for all intermediates, radioTLC and radioHPLC chromatograms, additional information on the biological evaluation, HPLC-MS chromatograms, and ^1^H NMR spectra of the tested compounds and radiolabeling precursors (PDF)

## Abbreviations

Aβ: amyloid β
AD: Alzheimer’s disease
*A*_m_: molar activity
αSYN: α-synuclein
BBB: blood–brain barrier
CMRF: copper-mediated radiofluorination
CNS MPO: central nervous system multiparameter optimization
DLB: dementia with Lewy bodies
IHC: immunohistochemistry
MSA: multiple system atrophy
NSB: nonspecific binding
PD: Parkinson’s disease
PET: positron emission tomography
RCC: radiochemical conversion
RCY: radiochemical yield
*R_f_*: retention factor
ROI: region of interest
SAR: structure–activity relationship
SB: specific binding
SEM: standard error of the mean
SUV: standardized uptake value
TAC: time–activity curve
ThT: thioflavin T
TPSA: topological polar surface area
VOI: volume of interest
