# Supplementary material for: A Fluorescent Probe as a Lead Compound for a Selective α-Synuclein PET Tracer: Development of a Library of 2-Styrylbenzothiazoles and Biological Evaluation of [18F]PFSB and [18F]MFSB
Source: ACS Omega. 2023 Aug 15;8(34):31450–67. doi: 10.1021/acsomega.3c04292 (PMC10468942; doi:10.1021/acsomega.3c04292)
Supplement: Supplementary file 1 — ao3c04292_si_001.pdf [file ao3c04292_si_001.pdf]

Supporting information

A fluorescent probe as a lead compound for a selective α-synuclein PET tracer: development of a library of 2-styrylbenzothiazoles and biological evaluation of [^{18}F]PFSB and [^{18}F]MFSB

Adriana Di Nanni[§]; Ran Sing Saw[§]; Umberto M. Battisti[#]; Gregory D. Bowden[§ ‖]; Adam Boeckermann[§]; Kaare Bjerregaard-Andersen[†]; Bernd J. Pichler[§ ‖]; Kristina Herfert[§]; Matthias M. Herth[# ‡]; Andreas Maurer[§ ‖ *]

§ Werner Siemens Imaging Center, Department of Preclinical Imaging and Radiopharmacy, Eberhard Karls University Tübingen, Röntgenweg 13, 72076 Tübingen, Germany

Department of Drug Design and Pharmacology, Faculty of Health and Medicinal Sciences, University of Copenhagen, Jagtvej 160, 2100 Copenhagen, Denmark

‖ Cluster of Excellence iFIT (EXC 2180) "Image-Guided and Functionally Instructed Tumor Therapies", Eberhard Karls University Tübingen, Germany

† Department of Antibody Engineering and Biochemistry, H. Lundbeck A/S, Ottiliavej 9, 2500 Valby, Denmark

‡ Department of Clinical Physiology, Nuclear Medicine & PET, Rigshospitalet, Blegdamsvej 9, 2100 Copenhagen, Denmark

* Corresponding author

Table of Contents

1. Synthesis of intermediates S2
2. Radiochemistry S15
3. Biological evaluation S19
4. HPLC-MS chromatograms of the tested compounds S24
5. HPLC-MS chromatograms of radiolabeling precursors 48 and 49 ....S45
6. ^{1}H NMR spectra of the tested compounds S46
7. ^{1}H NMR spectra of radiolabeling precursors 48 and 49 ....S88

1. Synthesis of intermediates

General procedure S1

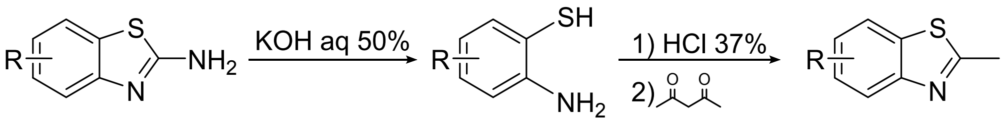

A suspension of the selected 2-aminobenzothiazole in KOH aq 50% was refluxed for 18 h to 42 h. The mixture was acidified by addition of HCl 37%. Color changed and precipitate formed. 2,4-pentanedione was added and the reaction was stirred at room temperature for 90 min. The mixture was extracted with EtOAc. The organic phase was dried over $MgSO_4$, evaporated under reduced pressure, and purified by flash chromatography (Hept/EtOAc).

General procedure S2

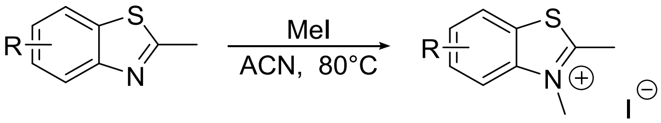

To a solution of the selected 2-methylbenzothiazole in MeCN was added methyl iodide. The mixture was stirred overnight at 80 °C. The precipitate was filtered under vacuum, washed with EtOAc and/or triturated in Et_2O.

General procedure S3

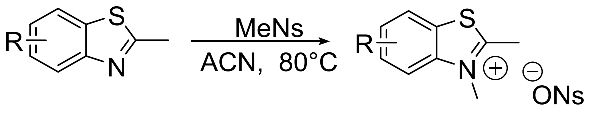

To a solution of the selected 2-methylbenzothiazole in MeCN was added methyl nosylate. The mixture was stirred overnight at 80 °C. The precipitate was filtered under vacuum, washed with EtOAc and/or triturated in Et_2O.

General procedure S4

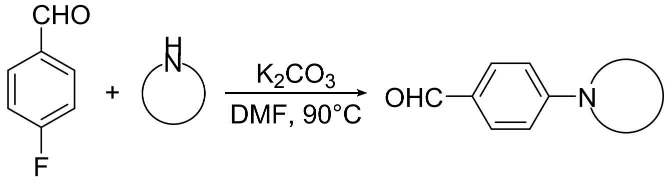

A mixture of 4-fluorobenzaldehyde and the selected secondary amine was diluted with DMF. K_2CO_3 was added and the reaction was stirred overnight at 90 °C. The mixture was diluted with water and extracted with EtOAc. The organic phase was dried over $MgSO_4$, evaporated under reduced pressure, and purified by flash chromatography (Hept/EtOAc).

2,4-dimethylbenzo[d]thiazole (21a)

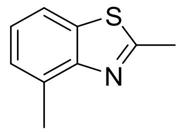

The synthesis was carried out according to general procedure S1 (2-amino-4-methylbenzothiazole: 1.21 g, 7.36 mmol; KOH aq: 28.0 mL; 2,4-pentanedione: 1.11 g, 11.0 mmol), additionally adding ethylen glycol (3.5 mL) to the mixture (393 mg, 33%). R_f: 0.41 (Hept/EtOAc 4:1). ^{1}H NMR (400 MHz, DMSO-d_6) δ 7.82 (pd, *J* = 3.6, 0.6 Hz, 1H), 7.28 (s, 1H), 7.27 – 7.25 (m, 1H), 2.80 (s, 3H), 2.63 (s, 3H). ^{13}C NMR (101 MHz, DMSO-d_6) δ 165.6, 152.2, 134.9, 131.4, 126.4, 124.6, 119.2, 19.8, 18.1. HPLC-MS (ESI): m/z calcd for C_9H_9NS 163.05; $[M+H]^+$ found 164.06.

2,3,4-trimethylbenzo[d]thiazol-3-ium 4-nitrobenzenesulfonate (21b)

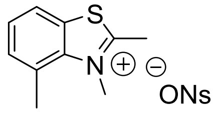

The synthesis was carried out according to general procedure S3 (**21a**: 158 mg, 0.97 mmol; MeONs: 420 mg, 1.94 mmol; MeCN: 3.00 mL, product: 248 mg, 67%). R_f: 0.29 (DCM/MeOH 9:1). ^{1}H NMR (400 MHz, DMSO-d_6) δ 8.30 – 8.22 (m, 1H), 8.19 (d, *J* = 8.4 Hz, 2H), 7.82 (d, *J* = 8.3 Hz, 2H), 7.64 (d, *J* = 2.1 Hz, 1H), 7.62 (s, 1H), 4.36 (s, 3H), 3.13 (s, 3H), 2.93 (s, 3H). ^{13}C NMR (151 MHz, DMSO-d_6) δ 176.6, 154.2, 147.3, 140.6, 132.8, 129.5, 128.3, 127.7, 126.9, 123.4, 122.4, 39.9, 20.4, 17.6. HPLC-MS (ESI): m/z calcd for $C_{10}H_{12}NS^+$ 178.07; $[M]^+$ found 178.07.

4-methoxy-2-methylbenzo[d]thiazole (22a)

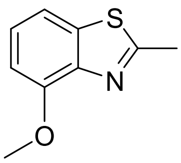

The synthesis was carried out according to general procedure S1 (2-amino-4-methoxybenzothiazole: 1.14 g; KOH aq: 22.0 mL; 2,4-pentanedione: 950 mg, 9.49 mmol; product: 223 mg, 20%). R_f: 0.09 (Hept/EtOAc 4:1). ^{1}H NMR (400 MHz, DMSO-d_6) δ 7.55 (dd, *J* = 8.0, 1.0 Hz, 1H), 7.32 (t, *J* = 8.0 Hz, 1H), 7.01 (dd, *J* = 8.0, 1.0 Hz, 1H), 3.93 (s, 3H), 2.77 (s, 3H). ^{13}C NMR (101 MHz, DMSO-d_6) δ 164.7, 152.6, 142.8, 136.7, 125.7, 113.6, 107.4, 55.7, 19.7. HPLC-MS (ESI): m/z calcd for C_9H_9NOS 179.04; $[M+H]^+$ found 180.00.

4-methoxy-2,3-dimethylbenzo[d]thiazol-3-ium iodide (22b)

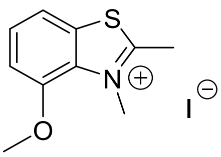

The synthesis was carried out according to general procedure S2 (**22a**: 95.0 mg, 0.53 mmol; MeI: 66.0 µL, 1.06 mmol; MeCN: 2.00 mL; product: 35.0 mg, 20%). R_f: 0.34 (DCM/MeOH 9:1). ^{1}H NMR (600 MHz, DMSO-d_6) δ 7.93 (dd, *J* = 8.2, 0.9 Hz, 1H), 7.70 (t, *J* = 8.2 Hz, 1H), 7.45 (dd, *J* = 8.2, 0.9 Hz, 1H), 4.35 (s, 3H), 4.05 (s, 3H), 3.09 (s, 3H). ^{13}C NMR (151 MHz, DMSO-d_6) δ 176.4, 150.4, 131.5, 131.1, 129.5, 116.4, 112.1, 57.5, 40.6, 17.6. HPLC-MS (ESI): m/z calcd for $C_{10}H_{12}NOS^+$ 194.06; $[M]^+$ found 194.10.

4-fluoro-2-methylbenzo[d]thiazole (23a)

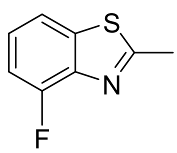

The synthesis was carried out according to general procedure S1 (2-amino-4-fluorobenzothiazole: 1.20 g, 7.13 mmol; KOH aq: 25.0 mL; 2,4-pentanedione; 1.10 mL, 10.7 mmol; product: 431 mg, 36%). R_f: 0.35 (Hept/EtOAc 4:1). ^{1}H NMR (400 MHz, DMSO-d_6) δ 7.86 (dd, *J* = 8.1, 1.1 Hz, 1H), 7.41 (td, *J* = 8.1, 4.9 Hz, 1H), 7.31 (ddd, *J* = 11.0, 8.1, 1.1 Hz, 1H), 2.82 (s, 3H). ^{13}C NMR (101 MHz, DMSO-d_6) δ 168.2, 154.4 (d, $J_{C\text{-}F}$ = 252.9 Hz), 141.2 (d, $J_{C\text{-}F}$ = 13.3 Hz), 138.1 (d, $J_{C\text{-}F}$ = 3.8 Hz), 125.7 (d, $J_{C\text{-}F}$ = 7.1 Hz), 118.1 (d, $J_{C\text{-}F}$ = 4.1 Hz), 111.7 (d, $J_{C\text{-}F}$ = 17.9 Hz), 19.7. ^{19}F NMR (376 MHz, DMSO-d_6) δ -123.34. HPLC-MS (ESI): m/z calcd for C_8H_6FNS 167.02; $[M+2H]^+$ found 169.12.

4-fluoro-2,3-dimethylbenzo[d]thiazol-3-ium 4-nitrobenzenesulfonate (23b)

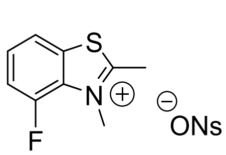

The synthesis was carried out according to general procedure S3 (**23a**: 105 mg, 0.63 mmol; MeONs: 273 mg, 1.26 mmol; MeCN: 2.00 mL; product: 188 mg, 78%). R_f: 0.32 (DCM/MeOH 9:1). ^{1}H NMR (400 MHz, DMSO-d_6) δ 8.28 – 8.20 (m, 1H), 8.18 (d, *J* = 8.4 Hz, 2H), 7.86 – 7.75 (m, 4H), 4.29 (d, *J* = 2.7 Hz, 3H), 3.16 (s, 3H). ^{13}C NMR (101 MHz, DMSO-d_6) δ 178.6, 154.3, 150.8 (d, J_{C-F} = 253.2 Hz), 147.2, 131.3, 130.3 (d, J_{C-F} = 10.7 Hz), 129.1 (d, J_{C-F} = 7.5 Hz), 126.9, 123.3, 120.8 (d, J_{C-F} = 4.6 Hz), 116.0 (d, J_{C-F} = 19.1 Hz), 39.2, 17.0. ^{19}F NMR (376 MHz, DMSO-d_6) δ -125.33. HPLC-MS (ESI): m/z calcd for $C_9H_9FNS^+$ 182.04; $[M]^+$ found 182.06.

2,3,5-trimethylbenzo[d]thiazol-3-ium iodide (24b)

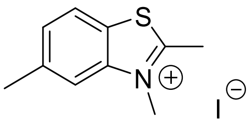

The synthesis was carried out according to general procedure S2 from the commercially available **24a** (**24a**: 400 mg, 2.45 mmol; MeI: 305 µL, 4.90 mmol; MeCN: 8.50 mL; product: 460 mg, 61%). R_f: 0.32 (DCM/MeOH 9:1). ^{1}H NMR (400 MHz, DMSO-d_6) δ 8.29 (d, *J* = 8.4 Hz, 1H), 8.14 (d, *J* = 1.5 Hz, 1H), 7.64 (dd, *J* = 8.4, 1.5 Hz, 1H), 4.17 (s, 3H), 3.15 (s, 3H), 2.57 (s, 3H). ^{13}C NMR (101 MHz, DMSO-d_6) δ 176.8, 141.8, 139.8, 129.5, 125.8, 123.9, 116.4, 36.0, 21.1, 17.0. HPLC-MS (ESI): m/z calcd for $C_{10}H_{12}NS^+$ 178.07; $[M]^+$ found 178.08.

5-methoxy-2-methylbenzo[d]thiazole (25a)

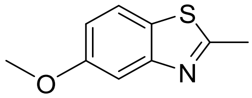

To a solution of 2-methyl-5-benzothiazolol (500 mg, 3.03 mmol) in DMF (8.50 mL) was added K_2CO_3 (836 mg, 6.05 mmol). The mixture was stirred at room temperature for 10 min. Methyl iodide (283 µL, 4.54 mmol) was added dropwise and the reaction was stirred at room temperature for 1 h. The mixture was diluted with water and extracted with EtOAc. The organic phase was dried over $MgSO_4$, evaporated under reduced pressure and used without any further purification (477 mg, 88%). R_f: 0.16 (Hept/EtOAc 4:1). ^{1}H NMR (400 MHz, DMSO-d_6) δ 7.87 (d, *J* = 8.8 Hz, 1H), 7.45 (d, *J* = 2.5 Hz, 1H), 7.02 (dd, *J* = 8.8, 2.5 Hz, 1H), 3.83 (s, 3H), 2.76 (s, 3H). ^{13}C NMR (101 MHz, DMSO-d_6) δ 168.0, 158.4, 154.3, 126.8, 122.2, 114.1, 105.1, 55.4, 19.8. HPLC-MS (ESI): m/z calcd for C_9H_9NOS 179.04; $[M+H]^+$ found 180.05.

5-methoxy-2,3-dimethylbenzo[d]thiazol-3-ium iodide (25b)

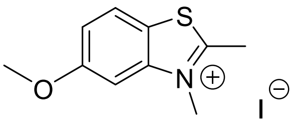

The synthesis was carried out according to general procedure S2 (**25a**: 210 mg, 1.17 mmol; MeI: 146 µL, 2.34 mmol; MeCN: 4.00 mL; product: 204 mg, 54%). R_f: 0.40 (DCM/MeOH 9:1). ^{1}H NMR (400 MHz, DMSO-d_6) δ 8.29 (d, *J* = 9.0 Hz, 1H), 7.78 (d, *J* = 2.4 Hz, 1H), 7.42 (dd, *J* = 9.0, 2.4 Hz, 1H), 4.17 (s, 3H),

3.97 (s, 3H), 3.13 (s, 3H). ^{13}C NMR (101 MHz, DMSO-d_6) δ 177.0, 160.6, 143.1, 125.1, 120.4, 117.7, 100.0, 56.4, 36.2, 17.1. HPLC-MS (ESI): m/z calcd for $C_{10}H_{12}NOS^+$ 194.06; $[M]^+$ found 194.07.

5-fluoro-2,3-dimethylbenzo[d]thiazol-3-ium iodide (26b)

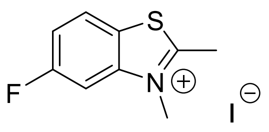

The synthesis was carried out according to general procedure S2 from commercially available **26a** (**26a**: 400 mg, 2.38 mmol; MeI: 298 µL, 4.78 mmol; MeCN: 8.00 mL; product: 168 mg, 23%). R_f: 0.32 (DCM/MeOH 9:1). ^{1}H NMR (400 MHz, DMSO-d_6) δ 8.48 (dd, *J* = 9.1, 5.1 Hz, 1H), 8.34 (dd, *J* = 9.5, 2.5 Hz, 1H), 7.74 (td, *J* = 9.0, 2.4 Hz, 1H), 4.17 (s, 3H), 3.17 (s, 3H). ^{13}C NMR (101 MHz, DMSO-d_6) δ 179.5, 162.3 (d, J_{C-F} = 246.7 Hz), 142.7 (d, J_{C-F} = 12.8 Hz), 126.5 (d, J_{C-F} = 10.1 Hz), 124.7 (d, J_{C-F} = 2.1 Hz), 116.8 (d, J_{C-F} = 25.0 Hz), 104.2 (d, J_{C-F} = 28.8 Hz), 36.4, 17.3. ^{19}F NMR (376 MHz, DMSO-d_6) δ -110.17. HPLC-MS (ESI): m/z calcd for $C_9H_9FNS^+$ 182.04; $[M]^+$ found 182.05.

2,6-dimethylbenzo[d]thiazole (27a)

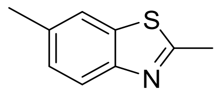

The synthesis was carried out according to general procedure S1 (2-amino-6-methylbenzothiazole: 2.50 g, 15.2 mmol; KOH aq: 53.0 mL; 2,4-pentanedione: 2.34 mL, 22.8 mmol; product: 1.50 g, 60%). R_f: 0.37 (Hept/EtOAc 3:1). ^{1}H NMR (400 MHz, DMSO-d_6) δ 7.80 (p, *J* = 0.8 Hz, 1H), 7.77 (d, *J* = 8.3 Hz, 1H), 7.27 (dd, *J* = 8.2, 1.6 Hz, 1H), 2.76 (s, 3H), 2.42 (s, 3H). ^{13}C NMR (101 MHz, DMSO-d_6) δ 165.6, 151.1, 135.3, 134.2, 127.3, 121.5, 121.4, 20.9, 19.6. HPLC-MS (ESI): m/z calcd for C_9H_9NS 163.05; $[M+H]^+$ found 164.06

2,3,6-trimethylbenzo[d]thiazol-3-ium iodide (27b)

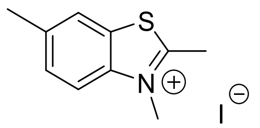

The synthesis was carried out according to general procedure S2 (**27a**: 400 mg, 2.45 mmol; MeI: 305 µL, 4.90 mmol; MeCN: 9.00; product: 378 mg, 50%). R_f: 0.28 (DCM/MeOH 9:1). ^{1}H NMR (400 MHz, DMSO-d_6) δ 8.21 (t, *J* = 1.2 Hz, 1H), 8.18 (d, *J* = 8.7 Hz, 1H), 7.72 (dd, *J* = 8.7, 1.7 Hz, 1H), 4.17 (s, 3H), 3.14 (s, 3H), 2.53 (s, 3H). ^{13}C NMR (101 MHz, DMSO-d_6) δ 176.0, 139.8, 138.4, 130.5, 128.7, 123.7, 116.3, 36.1, 21.0, 17.0. HPLC-MS (ESI): m/z calcd for $C_{10}H_{12}NS^+$ 178.07; $[M]^+$ found 178.07.

6-methoxy-2-methylbenzo[d]thiazole (28a)

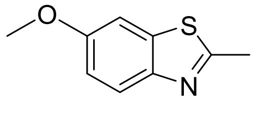

The synthesis was carried out according to general procedure S1 (2-amino-6-methoxybenzothiazole: 2.20 g, 12.2 mmol; KOH aq: 43.0 mL; 2,4-pentanedione: 1.88 mL, 18.3 mmol; product: 1.34 g, 61%). R*f*: 0.29 (Hept/EtOAc 3:1). ^{1}H NMR (400 MHz, DMSO-*d*$_6$) δ 7.78 (d, *J* = 8.9 Hz, 1H), 7.59 (d, *J* = 2.6 Hz, 1H), 7.05 (dd, *J* = 8.9, 2.6 Hz, 1H), 3.81 (s, 3H), 2.73 (s, 3H). ^{13}C NMR (101 MHz, DMSO-*d*$_6$) δ 164.0, 156.9, 147.3, 136.5, 122.3, 114.9, 104.7, 55.6, 19.5. HPLC-MS (ESI): m/z calcd for C_9H_9NOS 179.04; $[M+H]^+$ found 180.04.

6-methoxy-2,3-dimethylbenzo[d]thiazol-3-ium iodide (28b)

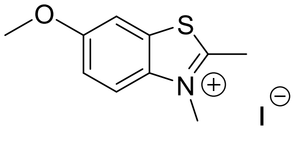

The synthesis was carried out according to general procedure S2 (**28a**: 400 mg, 2.23 mmol; MeI: 278 µL, 4.46 mmol; MeCN: 8.00 mL; product: 410 mg, 57%). R*f*: 0.15 (DCM/MeOH 9:1). ^{1}H NMR (400 MHz, DMSO-*d*$_6$) δ 8.18 (d, *J* = 9.3 Hz, 1H), 8.00 (d, *J* = 2.6 Hz, 1H), 7.47 (dd, *J* = 9.3, 2.6 Hz, 1H), 4.16 (s, 3H), 3.90 (s, 3H), 3.11 (s, 3H). ^{13}C NMR (101 MHz, DMSO-*d*$_6$) δ 174.3, 159.0, 135.8, 130.4, 118.4, 117.6, 106.6, 56.2, 36.2, 16.9. HPLC-MS (ESI): m/z calcd for $C_{10}H_{12}NOS^+$ 194.06; $[M]^+$ found 194.06.

6-fluoro-2-methylbenzo[d]thiazole (29a)

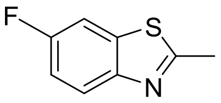

2-amino-5-fluorobenzenethiol (300 mg, 2.09 mmol) and 2,4-pentanedione (323 µL, 3.14 mmol) were dissolved in MeCN (1 mL) and p-toluenesulfonic acid (18.0 mg, 0.10 mmol) was added. The mixture was stirred overnight at room temperature. It was diluted with water and extracted with EtOAc. The organic phase was dried over $MgSO_4$, evaporated under reduced pressure, and purified by flash chromatography (Hept/EtOAc 0% to 20% B) to afford the product (257 mg, 73%). R*f*: 0.29 (Hept/EtOAc 5:1). ^{1}H NMR (400 MHz, DMSO-*d*$_6$) δ 7.94 (dd, *J* = 8.2, 2.1 Hz, 1H), 7.91 (dd, *J* = 8.3, 4.3 Hz, 1H), 7.33 (td, *J* = 9.1, 2.7 Hz, 1H), 2.78 (s, 3H). ^{13}C NMR (101 MHz, DMSO-*d*$_6$) δ 167.1 (d, J_{C-F} = 3.2 Hz), 159.4 (d, J_{C-F} = 241.7 Hz), 149.7, 136.4 (d, J_{C-F} = 11.7 Hz), 123.0 (d, J_{C-F} = 9.5 Hz), 114.3 (d, J_{C-F} = 24.8 Hz), 108.3 (d, J_{C-F} = 27.1 Hz), 19.7. ^{19}F NMR (376 MHz, DMSO-*d*$_6$) δ -117.24. HPLC-MS (ESI): m/z calcd for C_8H_6FNS 167.02; $[M+H]^+$ found 168.04.

6-fluoro-2,3-dimethylbenzo[d]thiazol-3-ium iodide (29b)

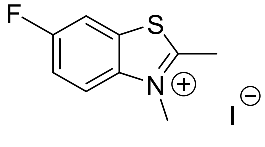

The synthesis was carried out according to general procedure S2 (**29a**: 165 mg, 0.99 mmol; MeI: 123 µL, 1.97 mmol; MeCN: 3.50 mL; product: 73.6 mg, 24%). R*f*: 0.17 (DCM/MeOH 9:1). ^{1}H NMR (600 MHz, DMSO-*d*$_6$) δ 8.36 (dd, *J* = 9.1, 4.3 Hz, 1H), 8.32 (dd, *J* = 8.3, 2.7 Hz, 1H), 7.84 (td, *J* = 9.1, 2.7 Hz, 1H), 4.19 (s, 3H), 3.15 (s, 3H). ^{13}C NMR (151 MHz, DMSO-*d*$_6$) δ 177.8 (d, J_{C-F} = 2.5 Hz), 160.7 (d, J_{C-F} = 247.2 Hz),

138.5, 130.3 (d, J_{C-F} = 12.2 Hz), 118.8 (d, J_{C-F} = 9.6 Hz), 117.9 (d, J_{C-F} = 25.7 Hz), 110.9 (d, J_{C-F} = 28.6 Hz), 36.5, 17.2. ^{19}F NMR (376 MHz, DMSO-d_6) δ -111.66. HPLC-MS (ESI): m/z calcd for $C_9H_9FNS^+$ 182.04; $[M]^+$ found 182.06.

1-methyl-3-nitro-2-thiocyanatobenzene (30.1)

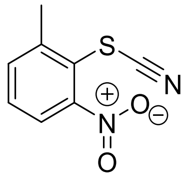

To a suspension of 2-methyl-6-nitroaniline (1.20 g, 7.89 mmol) in water (27.0 mL) cooled to 0 °C, HCl 37% (12.0 mL) was added. Sodium nitrite (653 mg, 9.46 mmol) was dissolved in water (5.00 mL) and added dropwise. After stirring for 5 min at 0 °C, a solution of potassium thiocyanate (2.30 g, 23.7 mmol) and iron (III) chloride (640 mg, 3.94 mmol) in water (9.00 mL) was added dropwise. The mixture was stirred at room temperature for 5 h. It was diluted with water and extracted with EtOAc. The organic phase was dried over $MgSO_4$, evaporated and used without any further purification (quant.). R_f: 0.54 (Hept/EtOAc 1:1). ^{1}H NMR (400 MHz, DMSO-d_6) δ 7.96 (d, J = 7.0 Hz, 1H), 7.82 (d, J = 6.9 Hz, 1H), 7.73 (t, J = 7.3 Hz, 1H), 2.66 (s, 3H). ^{13}C NMR (101 MHz, DMSO-d_6) δ 152.9, 144.5, 135.4, 132.1, 123.1, 117.0, 110.5, 21.1.

2,7-dimethylbenzo[d]thiazole (30a)

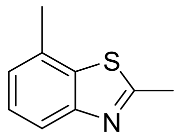

To a solution of **30.1** (2.55 g, 13.1 mmol) in EtOH/H_2O 1:1 v/v (45 mL, 45 mL), Na_2S (2.05 g, 26.3 mmol) was added in portions under argon atmosphere. The mixture was heated at 65 °C for 3 h and then extracted with EtOAc. The organic phase was dried over $MgSO_4$, evaporated under reduced pressure, and purified by flash chromatography (DCM/MeOH 0% to 2% B). The collected residue was dissolved in MeCN (1 mL) and 2,4-pentanedione (0.06 mL, 0.60 mmol) and p-toluenesulfonic acid (3.00 mg, 0.02 mmol) were added. The mixture was stirred at room temperature for 3 h. It was diluted with water and extracted with EtOAc. The organic phase was dried over $MgSO_4$, evaporated, and purified by flash chromatography (PE/EtOAc 0% to 20% B) to afford the product (17.0 mg, 1%). R_f: 0.26 (PE/EtOAc 4:1). ^{1}H NMR (400 MHz, $CDCl_3$) δ 7.79 (d, J = 8.1 Hz, 1H), 7.36 (t, J = 7.7 Hz, 1H), 7.15 (dt, J = 7.4, 1.0 Hz, 1H), 2.85 (s, 3H), 2.54 (s, 3H). ^{13}C NMR (151 MHz, $CDCl_3$) δ 166.7, 153.2, 136.2, 131.7, 126.3, 125.1, 119.9, 21.6, 20.3. HPLC-MS (ESI): m/z calcd for C_9H_9NS 163.05; $[M+H]^+$ found 164.10.

2,3,7-trimethylbenzo[d]thiazol-3-ium iodide (30b)

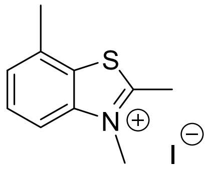

The synthesis was carried out according to general procedure S2 (**30a**: 16.0 mg, 0.10 mmol; MeI: 18.3 µL, 0.29 mmol; MeCN: 0.50 mL; product: 12.0 mg, 40%). R_f: 0.35 (DCM/MeOH 9:1). ^{1}H NMR (600 MHz, DMSO-d_6) δ 8.15 (d, *J* = 8.5 Hz, 1H), 7.83 (dd, *J* = 8.5, 7.5 Hz, 1H), 7.66 (dt, *J* = 7.5, 1.0 Hz, 1H), 4.21 (s, 3H), 3.20 (s, 3H), 2.66 (s, 3H). ^{13}C NMR (151 MHz, DMSO-d_6) δ 176.2, 147.0, 136.7, 133.5, 129.7, 128.5, 114.5, 36.6, 19.3, 17.2. HPLC-MS (ESI): m/z calcd for $C_{10}H_{12}NS^+$ 178.07; $[M]^+$ found 178.05.

2-methoxy-6-nitroaniline (31.1)

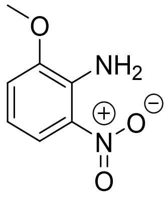

To a solution of 2-amino-3-nitrophenol (4.18 g, 27.1 mmol) in MeCN (47.0 mL) was added K_2CO_3 (3.75 g, 27.1 mmol). Methyl iodide (1.86 mL, 29.8 mmol) was added dropwise and the mixture was stirred at room temperature for 22 h. It was diluted with water and extracted with EtOAc. The organic phase was dried over $MgSO_4$, evaporated under reduced pressure, and purified by flash chromatography (Hept/EtOAc 0% to 20% B) to afford the product as an orange solid (2.63 g, 58%). R_f: 0.39 (Hept/EtOAc 2:1). ^{1}H NMR (400 MHz, $CDCl_3$) δ 7.73 (dd, *J* = 8.9, 1.3 Hz, 1H), 6.88 (dd, *J* = 7.8, 1.3 Hz, 1H), 6.61 (dd, *J* = 8.9, 7.8 Hz, 1H), 6.42 (s, 2H), 3.92 (s, 3H). ^{13}C NMR (101 MHz, $CDCl_3$) δ 148.3, 137.2, 131.8, 117.5, 114.7, 113.5, 56.4. HPLC-MS (ESI): m/z calcd for $C_7H_8N_2O_3$ 168.05; $[M+H]^+$ found 169.06.

1-methoxy-3-nitro-2-thiocyanatobenzene (31.2)

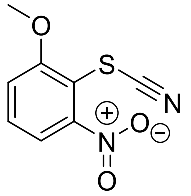

The synthesis was carried out following the same procedure as **30.1** (**31.1**: 2.56 g, 15.2 mmol; H_2O: 53.0 mL; HCl 37%: 25.0 mL; $NaNO_2$: 1.26 g, 18.3 mmol; KSCN: 4.44 g, 45.7 mmol; $FeCl_3$: 1.23 g, 7.61 mmol; quant.). R_f: 0.21 (PE/EtOAc 3:1). ^{1}H NMR (600 MHz, DMSO-d_6) δ 7.82 – 7.70 (m, 2H), 7.64 (dd, *J* = 8.2, 4.2 Hz, 1H), 4.07 (s, 3H). ^{13}C NMR (151 MHz, DMSO-d_6) δ 158.8, 132.9, 124.5, 117.7, 117.3, 109.9, 106.6, 57.5.

7-methoxy-2-methylbenzo[d]thiazole (31a)

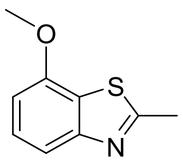

The synthesis was carried out following the same procedure as **30a** (**31.2**: 3.00 g, 14.3 mmol; EtOH: 48.o mL; H_2O: 48.0 mL; Na_2S: 2.23 g, 28.5 mmol; MeCN: 4.00 mL; 2,4-pentanedione: 531 µL, 5.17 mmol; p-toluenesulfonic acid: 29.7, 0.17 mmol; product: 49.0 mg, 2%). R_f: 0.27 (PE/EtOAc 4:1). ^{1}H NMR (600

MHz, DMSO-d_6) δ 6.79 (t, *J* = 8.0 Hz, 1H), 6.26 (dd, *J* = 8.3, 0.9 Hz, 1H), 6.21 (dd, *J* = 7.8, 0.9 Hz, 1H), 3.70 (s, 3H), 2.11 (s, 3H). ^{13}C NMR (151 MHz, DMSO-d_6) δ 206.2, 153.5, 147.4, 126.2, 111.1, 102.8, 101.5, 55.3, 30.9. HPLC-MS (ESI): m/z calcd for C_9H_9NOS 179.04; $[M+H]^+$ found 180.00.

7-methoxy-2,3-dimethylbenzo[d]thiazol-3-ium iodide (31b)

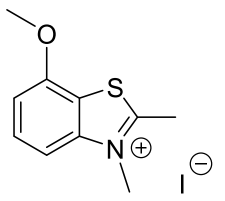

The synthesis was carried out according to general procedure S2 (**31a**: 42.0 mg, 0.23 mmol; MeI: 29.2 µL, 0.47 mmol; MeCN: 1.00 mL; product: 39.0 mg, 52%). R_f: 38 (DCM/MeOH 9:1). ^{1}H NMR (600 MHz, DMSO-d_6) δ 7.88 (s, 2H), 7.41 (s, 1H), 4.20 (s, 3H), 4.07 (s, 3H), 3.20 (s, 3H). ^{13}C NMR (151 MHz, DMSO-d_6) δ 176.9, 153.8, 142.9, 131.4, 117.0, 109.1, 109.1, 57.0, 36.9, 17.6. HPLC-MS (ESI): m/z calcd for $C_{10}H_{12}NOS^+$ 194.06; $[M]^+$ found 194.10.

N-(2-bromo-3-fluorophenyl)acetamide (32.1)

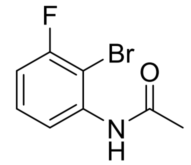

To a solution of 2-bromo-3-fluoroaniline (1.55 g, 8.16 mmol) in acetic anhydride (38.5 mL) was added pyridine (6.5 mL, 81.6 mmol). The mixture was stirred at room temperature for 3 h. It was poured into water and extracted with EtOAc. The organic phase was dried over $MgSO_4$, evaporated and used without any further purification (1.75 g, 93%). R_f: 0.18 (PE/EtOAc 4:1). ^{1}H NMR (600 MHz, DMSO-d_6) δ 9.58 (s, 1H), 7.47 (dt, *J* = 8.1, 1.3 Hz, 1H), 7.39 (td, *J* = 8.2, 6.2 Hz, 1H), 7.18 (td, *J* = 8.5, 1.5 Hz, 1H), 2.10 (s, 3H). ^{13}C NMR (151 MHz, DMSO-d_6) δ 168.7, 158.8 (d, $J_{C\text{-}F}$ = 243.7 Hz), 138.5, 128.7 (d, $J_{C\text{-}F}$ = 9.2 Hz), 122.4, 112.7 (d, $J_{C\text{-}F}$ = 22.2 Hz), 105.0 (d, $J_{C\text{-}F}$ = 24.1 Hz), 23.3. HPLC-MS (ESI): m/z calcd for C_8H_7BrFNO 230.97; $[M+H]^+$ found 232.05, 234.00.

N-(2-bromo-3-fluorophenyl)ethanethioamide (32.2)

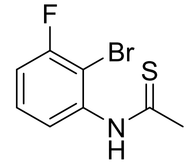

To a solution of **32.1** (1.40 g, 6.03 mmol) in THF (12 mL), Lawesson's reagent (1.71 g, 4.22 mmol) was added under argon atmosphere. The mixture was stirred overnight at room temperature and the precipitate was filtered. The filtrate was collected and further purified by flash chromatography (PE/EtOAc 5% to 80% B) to afford the product as an orange oil (1.04 g, 69%). R_f: 0.72 (PE/EtOAc 1:1). ^{1}H NMR (600 MHz, DMSO-d_6) δ 11.54 (s, 1H), 7.47 (td, *J* = 8.2, 6.1 Hz, 1H), 7.35 (td, *J* = 8.5, 1.4 Hz, 1H), 7.25 (dt, *J* = 8.0, 1.4 Hz, 1H), 2.62 (s, 3H). ^{13}C NMR (151 MHz, DMSO-d_6) δ 202.4, 159.1 (d, $J_{C\text{-}F}$ = 244.9

Hz), 140.5 (d, J_{C-F} = 1.9 Hz), 129.1 (d, J_{C-F} = 9.3 Hz), 125.7 (d, J_{C-F} = 3.1 Hz), 115.3 (d, J_{C-F} = 22.4 Hz), 108.5 (d, J_{C-F} = 21.4 Hz), 33.2. HPLC-MS (ESI): m/z calcd for C_8H_7BrFNS 246.95; $[M+H]^+$ found 247.95, 249.90.

7-fluoro-2-methylbenzo[d]thiazole (32a)

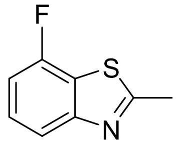

To a solution of **32.2** (810 mg, 3.26 mmol) in 1,4-dioxane (12 mL), Cs_2CO_3 (1.60 g, 4.90 mmol) and $Pd(PPh_3)_4$ (189 mg, 0.16 mmol) were added under argon atmosphere. The mixture was stirred overnight at 80 °C. It was diluted with water and extracted with EtOAc. The organic phase was dried over $MgSO_4$, evaporated under reduced pressure, and purified by flash chromatography (PE/EtOAc 1% to 15% B) to afford the product (314 mg, 58%). R_f: 0.53 (PE/EtOAc 3:1). ^1H NMR (600 MHz, DMSO-d_6) δ 7.78 (d, *J* = 8.1 Hz, 1H), 7.51 (td, *J* = 8.1, 5.7 Hz, 1H), 7.29 (ddd, *J* = 9.4, 8.2, 1.1 Hz, 1H), 2.83 (s, 3H). ^{13}C NMR (151 MHz, DMSO-d_6) δ 168.0, 156.2 (d, J_{C-F} = 246.6 Hz), 155.9 (d, J_{C-F} = 2.6 Hz), 127.4 (d, J_{C-F} = 7.6 Hz), 121.9 (d, J_{C-F} = 16.7 Hz), 118.3 (d, J_{C-F} = 3.4 Hz), 110.4 (d, J_{C-F} = 18.7 Hz), 19.7. HPLC-MS (ESI): m/z calcd for C_8H_6FNS 167.02; $[M+H]^+$ found 168.00.

7-fluoro-2,3-dimethylbenzo[d]thiazol-3-ium iodide (32b)

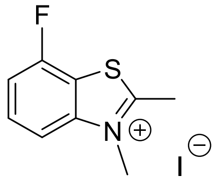

The synthesis was carried out according to general procedure S2 (**32a**: 103 mg, 0.62 mmol; MeI: 76.7 µL, 1.23 mmol; MeCN: 1.50 mL; product: 47.0 mg, 25%). R_f: 0.19 (DCM/MeOH 9:1). ^1H NMR (600 MHz, DMSO-d_6) δ 8.21 (d, *J* = 8.5 Hz, 1H), 7.97 (q, *J* = 7.5 Hz, 1H), 7.78 (t, *J* = 8.9 Hz, 1H), 4.24 (s, 3H), 3.23 (s, 3H). ^{13}C NMR (151 MHz, DMSO-d_6) δ 178.4, 155.8 (d, J_{C-F} = 250.7 Hz), 143.9 (d, J_{C-F} = 5.6 Hz), 131.5 (d, J_{C-F} = 8.1 Hz), 116.4 (d, J_{C-F} = 23.4 Hz), 114.1 (d, J_{C-F} = 17.8 Hz), 113.6 (d, J_{C-F} = 3.5 Hz), 37.1, 17.6. HPLC-MS (ESI): m/z calcd for $C_9H_9FNS^+$ 182.04; $[M]^+$ found 182.10.

2-methylbenzo[d]thiazole (33a)

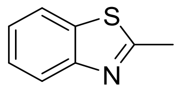

p-Toluenesulfonic acid (402 mg, 2.34 mmol) was added to a mixture of 2-aminobenzenethiol (5.00 mL, 46.7 mmol) and 2,4-pentanedione (7.20 mL, 70.1 mmol) and stirred overnight. The mixture was diluted with water and extracted with EtOAc. The organic phase was dried over $MgSO_4$, evaporated under reduced pressure, and purified by flash chromatography (Hept/EtOAc 0% to 20% B) to afford the product as an orange oil (5.74 g, 82%). R_f: 0.23 (Hept/EtOAc 5:1). ^1H NMR (400 MHz, DMSO-d_6) δ 8.02 (dt, *J* = 8.0, 1.0 Hz, 1H), 7.91 (dt, *J* = 8.2, 0.9 Hz, 1H), 7.47 (ddd, *J* = 8.2, 7.2, 1.4 Hz, 1H), 7.38 (ddd, *J* =

8.3, 7.3, 1.3 Hz, 1H), 2.79 (s, 3H). ^{13}C NMR (101 MHz, DMSO-d_6) δ 166.8, 153.0, 135.2, 125.9, 124.7, 121.9, 19.7. HPLC-MS (ESI): m/z calcd for C_8H_7NS 149.03; $[M+H]^+$ found 150.06.

2,3-dimethylbenzo[d]thiazol-3-ium iodide (33b)

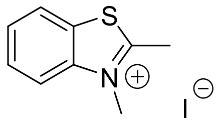

The synthesis was carried out according to general procedure S2 to afford a pink solid (**33a**: 1.23 g, 8.24 mmol; MeI: 1.03 mL, 16.49 mmol; MeCN: 25.0 mL; product: 1.21 g, 50%). R_f: 0.18 (DCM/MeOH 9:1). ^{1}H NMR (400 MHz, DMSO-d_6) δ 8.44 (dd, *J* = 8.1, 1.2 Hz, 1H), 8.29 (d, *J* = 8.4 Hz, 1H), 7.90 (ddd, *J* = 8.5, 7.3, 1.3 Hz, 1H), 7.81 (ddd, *J* = 8.3, 7.2, 1.1 Hz, 1H), 4.21 (s, 3H), 3.18 (s, 3H). ^{13}C NMR (101 MHz, DMSO-d_6) δ 177.2, 141.6, 129.2, 128.7, 128.0, 124.5, 116.7, 36.2, 17.1. HPLC-MS (ESI): m/z calcd for $C_9H_{10}NS^+$ 164.05; $[M]^+$ found 164.35.

4-(piperidin-1-yl)benzaldehyde (34)

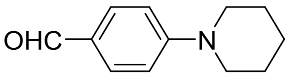

The synthesis was carried out according to general procedure S4 to afford a white solid (4-fluorobenzaldehyde: 5.00 mL, 46.6 mmol; piperidine: 3.91 mL, 39.6 mmol; K_2CO_3: 8.05 g, 58.3 mmol; DMF: 25.3 mL; product: 6.45 g, 86%). R_f: 0.18 (Hept/EtOAc 5:1). ^{1}H NMR (400 MHz, DMSO-d_6) δ 9.68 (s, 1H), 7.67 (dt, *J* = 9.8, 2.9 Hz, 2H), 7.00 (dt, *J* = 9.0, 2.7 Hz, 2H), 3.41 (dd, *J* = 6.6, 3.9 Hz, 4H), 1.64 – 1.52 (m, 6H). ^{13}C NMR (101 MHz, DMSO-d_6) δ 189.9, 154.6, 131.6, 125.4, 113.0, 47.6, 24.8, 23.9. HPLC-MS (ESI): m/z calcd for $C_{12}H_{15}NO$ 189.12; $[M+H]^+$ found 190.13.

4-morpholinobenzaldehyde (35)

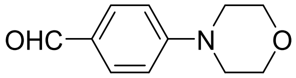

The synthesis was carried out according to general procedure S4 (4-fluorobenzaldehyde: 400 µL, 3.73 mmol; morpholine: 273 µL, 3.17 mmol; K_2CO_3: 773 mg, 5.59 mmol; DMF: 2.00 mL; product: 1.77 g, 58%). R_f: 0.19 (Hept/EtOAc 3:1). ^{1}H NMR (600 MHz, DMSO-d_6) δ 9.74 (s, 1H), 7.73 (dt, *J* = 9.0, 2.8 Hz, 2H), 7.06 (dt, *J* = 8.9, 2.6 Hz, 2H), 3.78 – 3.69 (m, 4H), 3.38 – 3.32 (m, 4H). ^{13}C NMR (151 MHz, DMSO-d_6) δ 190.4, 154.9, 131.4, 126.7, 113.2, 65.8, 46.6. HPLC-MS (ESI): m/z calcd for $C_{11}H_{13}NO_2$ 191.09; $[M+H]^+$ found 192.10.

4-thiomorpholinobenzaldehyde (36)

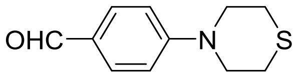

The synthesis was carried out according to general procedure S4 (4-fluorbenzaldehyde: 400 µL, 3.73 mmol; thiomorpholine: 318 µL, 3.17 mmol; K_2CO_3: 773 mg, 5.59 mmol; DMF: 2.00 mL; product: 73.5

mg, 11%). R_f: 0.22 (Hept/EtOAc 5:1). ^{1}H NMR (600 MHz, DMSO-d_6) δ 9.70 (s, 1H), 7.71 (dd, J = 9.0, 2.8 Hz, 2H), 7.13 – 6.89 (m, 2H), 3.93 – 3.69 (m, 4H), 2.75 – 2.55 (m, 4H). ^{13}C NMR (151 MHz, DMSO-d_6) δ 190.1, 153.3, 131.7, 125.8, 113.4, 49.4, 24.8. HPLC-MS (ESI): m/z calcd for $C_{11}H_{13}NOS$ 207.07; $[M+H]^+$ found 208.09.

4-(pyrrolidin-1-yl)benzaldehyde (37)

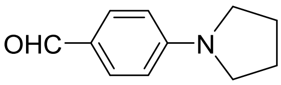

The synthesis was carried out according to general procedure S4 (4-fluorobenzaldehyde: 600 µL, 5.59 mmol; pyrrolidine: 390 µL, 4.75 mmol; K_2CO_3: 1.16 g, 8.39 mmol; DMF: 3.00 mL; product: 381 mg, 46%). R_f: 0.37 (Hept/EtOAc 3:1). ^{1}H NMR (400 MHz, DMSO-d_6) δ 9.64 (s, 1H), 7.67 (dt, J = 8.8, 2.7 Hz, 2H), 6.63 (dt, J = 8.8, 2.6 Hz, 2H), 3.38 – 3.30 (m, 4H), 1.98 – 1.93 (m, 4H).^{13}C 1NMR (101 MHz, DMSO-d_6) δ 189.6, 151.6, 131.7, 124.2, 111.2, 47.3, 24.9. HPLC-MS (ESI): m/z calcd for $C_{11}H_{13}NO$ 175.10; $[M+H]^+$ found 176.11.

4-(4-fluoropiperidin-1-yl)benzaldehyde (38)

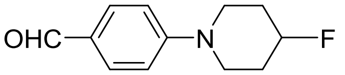

The synthesis was carried out according to general procedure S4 (4-fluorobenzaldehyde: 300 µL, 2.80 mmol; 4-fluoropiperidine: 332 mg, 2.38 mmol; K_2CO_3: 1.16 g, 8.40 mmol; DMF: 2.00 mL; product: 293 mg, 59%). R_f: 0.21 (Hept/EtOAc 3:1). ^{1}H NMR (400 MHz, DMSO-d_6) δ 9.71 (s, 1H), 7.70 (dt, J = 8.9, 2.5 Hz, 2H), 7.07 (dt, J = 8.9, 2.5 Hz, 2H), 4.90 (dtt, J = 48.9, 7.1, 3.6 Hz, 1H), 3.71 – 3.55 (m, 2H), 3.41 (ddd, J = 13.5, 7.4, 3.9 Hz, 2H), 1.94 (dddd, J = 24.9, 12.0, 8.1, 3.8 Hz, 2H), 1.84 – 1.62 (m, 2H). ^{13}C NMR (101 MHz, DMSO-d6) δ 190.6, 154.5, 132.1, 126.5, 113.8, 88.8 (d, $J_{C\text{-}F}$ = 169.5 Hz), 43.7 (d, $J_{C\text{-}F}$ = 6.6 Hz), 30.8 (d, $J_{C\text{-}F}$ = 19.3 Hz). ^{19}F NMR (376 MHz, DMSO-d_6) δ -177.3. HPLC-MS (ESI): m/z calcd for $C_{12}H_{14}FNO$ 207.11; $[M+H]^+$ found 208.11.

4-(dimethylamino)benzaldehyde (39)

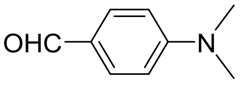

The synthesis was carried out according to general procedure S4 (4-fluorobenzaldehyde: 600 µL, 5.59 mmol; dimethylamine: 3.08 mL, 6.15 mmol; K_2CO_3: 1.16 g, 8.39 mmol; DMF: 1.00 mL; product: 346 mg, 41%). R_f: 0.33 (Hept/EtOAc 3:1). ^{1}H NMR (400 MHz, DMSO-d_6) δ 9.67 (s, 1H), 7.68 (dt, J = 9.1, 2.7 Hz, 2H), 6.79 (dt, J = 8.9, 2.8 Hz, 2H), 3.04 (s, 6H). ^{13}C NMR (101 MHz, DMSO-d_6) δ 190.3, 154.7, 132.0, 125.0, 111.5, 40.1. HPLC-MS (ESI): m/z calcd for $C_9H_{11}NO$ 149.08; $[M+H]^+$ found 150.09.

4-(4-methylpiperazin-1-yl)benzaldehyde (40)

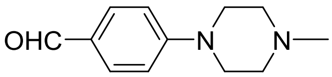

The synthesis was carried out according to general procedure S4 (4-fluorobenzaldehyde: 500 µL, 4.66 mmol; *N*-methylpiperazine: 439 µL, 3.96 mmol; K_2CO_3: 773 mg, 5.59 mmol; DMF: 2.50 mL; product: 408 mg, 50%). R_f: 0.08 (DCM/MeOH 2%). ^{1}H NMR (400 MHz, DMSO-d_6) δ 9.71 (s, 1H), 7.70 (dt, *J* = 9.0, 2.8 Hz, 2H), 7.04 (dt, *J* = 9.0, 2.5 Hz, 2H), 3.37 (dd, *J* = 6.4, 3.8 Hz, 4H), 2.42 (dd, *J* = 6.6, 3.6 Hz, 4H), 2.21 (s, 3H). ^{13}C NMR (101 MHz, DMSO-d_6) δ 190.2, 154.7, 131.4, 126.2, 113.3, 54.2, 46.3, 45.7. HPLC-MS (ESI): m/z calcd for $C_{12}H_{16}N_2O$ 204.13; $[M+H]^+$ found 205.14.

2-(4-bromophenyl)benzo[d]thiazole (41)

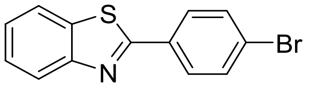

A solution of 2-aminobenzenethiol (750 mg, 5.99 mmol) and 4-bromobenzaldehyde (1.11 g, 5.99 mmol) in DMSO (6.00 mL) was heated at 140 °C for 75 min. The mixture was diluted with water and extracted with EtOAc. The organic phase was dried over $MgSO_4$, evaporated under reduced pressure, and recrystallized from Et_2O to afford the product as a white solid (569 mg, 33%). R_f: 0.70 (PE/EtOAc 5:1). ^{1}H NMR (600 MHz, DMSO-d_6) δ 8.16 (dt, *J* = 8.2, 0.8 Hz, 1H), 8.07 (dt, *J* = 8.0, 0.8 Hz, 1H), 8.03 (dt, *J* = 8.5, 2.5 Hz, 2H), 7.77 (dt, *J* = 8.5, 2.5 Hz, 2H), 7.56 (ddd, *J* = 8.2, 7.2, 1.3 Hz, 1H), 7.48 (ddd, *J* = 8.2, 7.2, 1.2 Hz, 1H). ^{13}C NMR (151 MHz, DMSO-d_6) δ 166.1, 153.5, 134.5, 132.4, 132.0, 129.0, 126.8, 125.8, 124.9, 123.0, 122.4. HPLC-MS (ESI): m/z calcd for $C_{13}H_8BrNS$ 288.96; $[M+H]^+$ found 289.95, 291.90.

3-(4-(piperidin-1-yl)phenyl)acrylaldehyde (42)

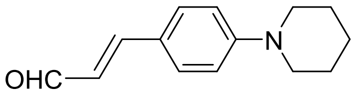

A solution of **34** (800 mg, 4.23 mmol) in H_2SO_4 96% (3 mL) was cooled to 0 °C and acetaldehyde (0.71 mL, 12.7 mmol) was added dropwise. The mixture was stirred for 1 h at 0 °C. It was poured into water, neutralized with NaOH aq 18 M and extracted with EtOAc. The organic phase was dried over $MgSO_4$, evaporated under reduced pressure, and purified by flash chromatography (PE/EtOAc 5% to 10% B) to afford the product (390 mg, 43%). R_f: 0.51 (PE/EtOAc 3:1). ^{1}H NMR (600 MHz, DMSO-d_6) δ 9.54 (d, *J* = 7.9 Hz, 1H), 7.59 – 7.52 (m, 3H), 6.95 (dt, *J* = 8.9, 3.1 Hz, 2H), 6.60 (dd, *J* = 15.6, 7.9 Hz, 1H), 3.34 (d, *J* = 5.4 Hz, 4H), 1.60 – 1.56 (m, 6H). ^{13}C NMR (151 MHz, DMSO-d6) δ 193.7, 153.9, 152.9, 130.6, 124.0, 122.7, 114.1, 47.9, 24.9, 24.0. HPLC-MS (ESI): m/z calcd for $C_{14}H_{17}NO$ 215.13; $[M+H]^+$ found 216.15.

3-(4-(pyrrolidin-1-yl)phenyl)acrylaldehyde (50)

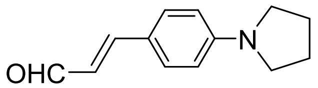

The synthesis was carried out following the same procedure as **42** (**37**: 700 mg, 3.99 mmol; H_2SO_4 96%: 3.00 mL; acetaldehyde: 223 µL, 3.99 mmol; product: 513 mg, 64%). R_f: 0.32 (PE/EtOAc 4:1). ^{1}H NMR (600 MHz, DMSO-d_6) δ 9.51 (d, *J* = 8.0 Hz, 1H), 7.58 – 7.51 (m, 3H), 6.59 – 6.53 (m, 3H), 3.32 – 3.28 (m, 4H), 2.00 – 1.92 (m, 4H). ^{13}C NMR (151 MHz, DMSO-d_6) δ 193.3, 154.5, 149.7, 130.8, 122.7, 120.8, 111.7, 47.3, 24.9. HPLC-MS (ESI): m/z calcd for $C_{13}H_{15}NO$ 201.12; $[M+H]^+$ found 202.20.

2. Radiochemistry

Table S1. Optimization of CMRF of **[^{18}F]PFSB**: variation of the amount of precursor and pyridine and corresponding RCC. The amount of $Cu(OTf)_2$, *n*-BuOH and DMA remained constant in all experiments.

#	Precursor **48** (µmol)	Pyridine (µmol)	RCC (%)
a	20	60	7.6
b	10	60	7.0
c	20	200	2.4
d	10	200	4.0

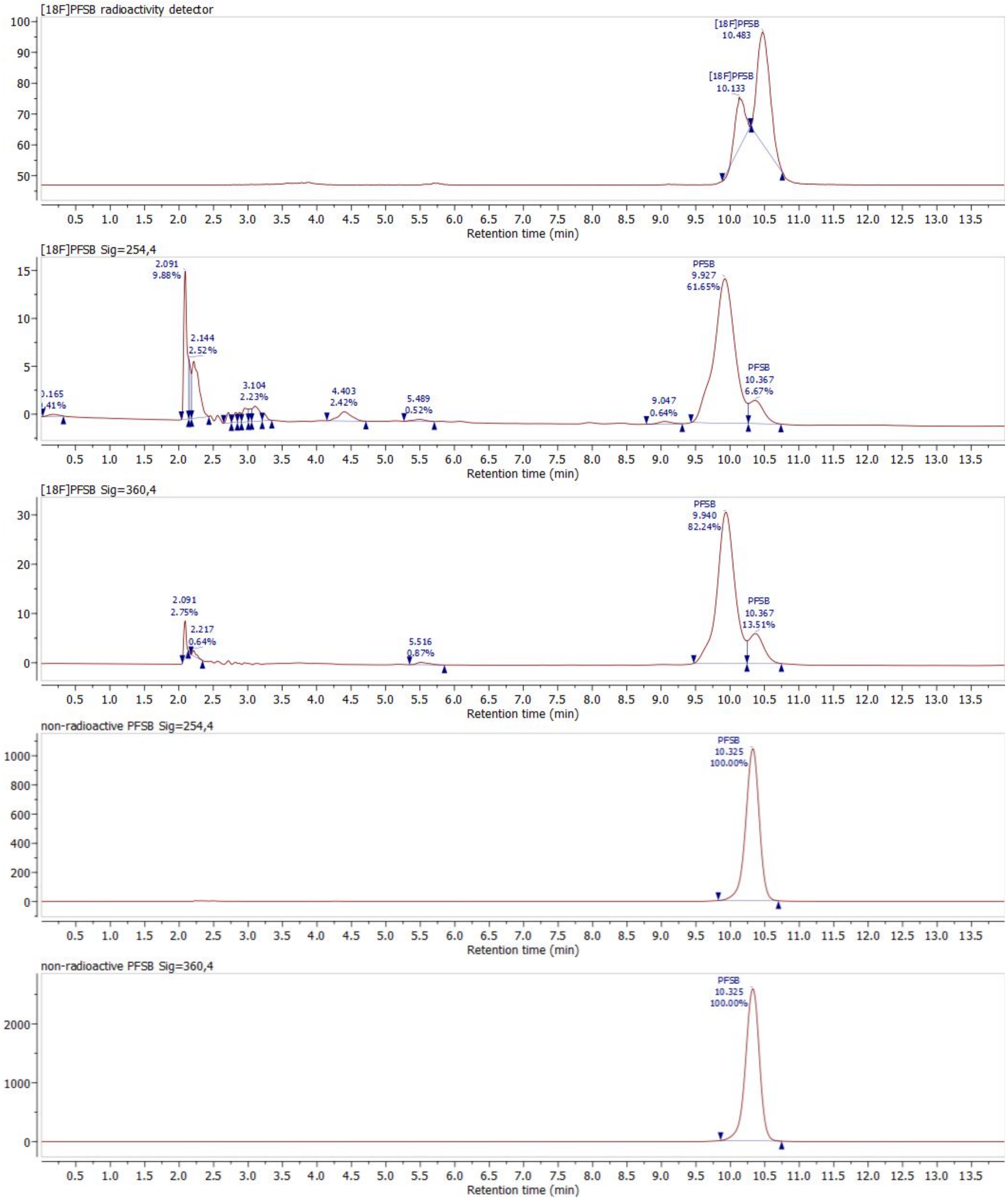

Figure S1. Example of analytical QC HPLC of [^{18}F]PFSB and its non-radioactive reference compound using a Luna column 5 µm C18 (2) 100 Å 250 x 4.6 mm, isocratic method: 88% MeCN in 25 mM ammonium formate at pH 8, 1 mL/min. The ratio between the two peaks (retention time ≈ 10.1 and 10.5 min) is dependent on the E/Z isomer ratio.

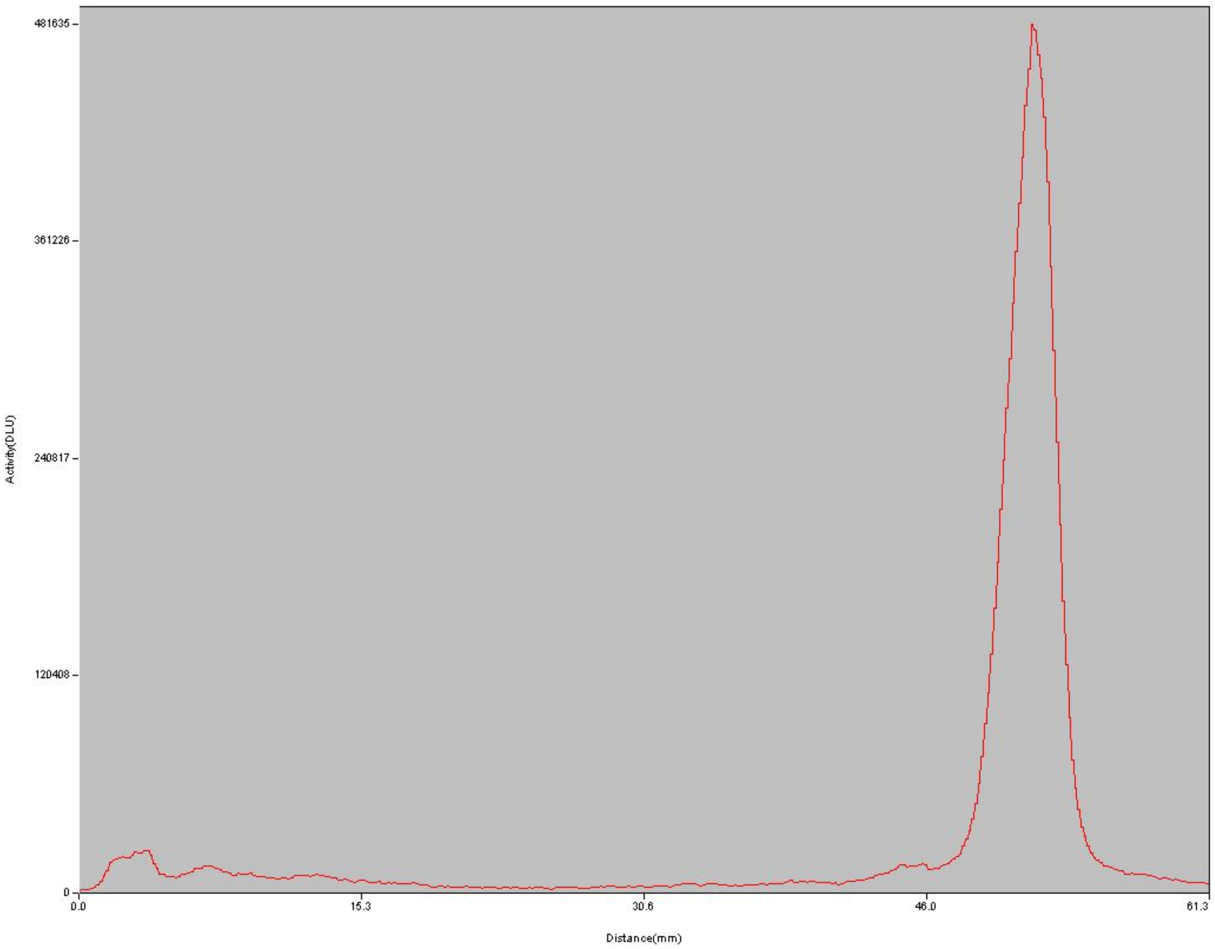

Figure S2. Example of radioTLC of [^{18}F]PFSB, eluent: PE/EtOAc 2:1.

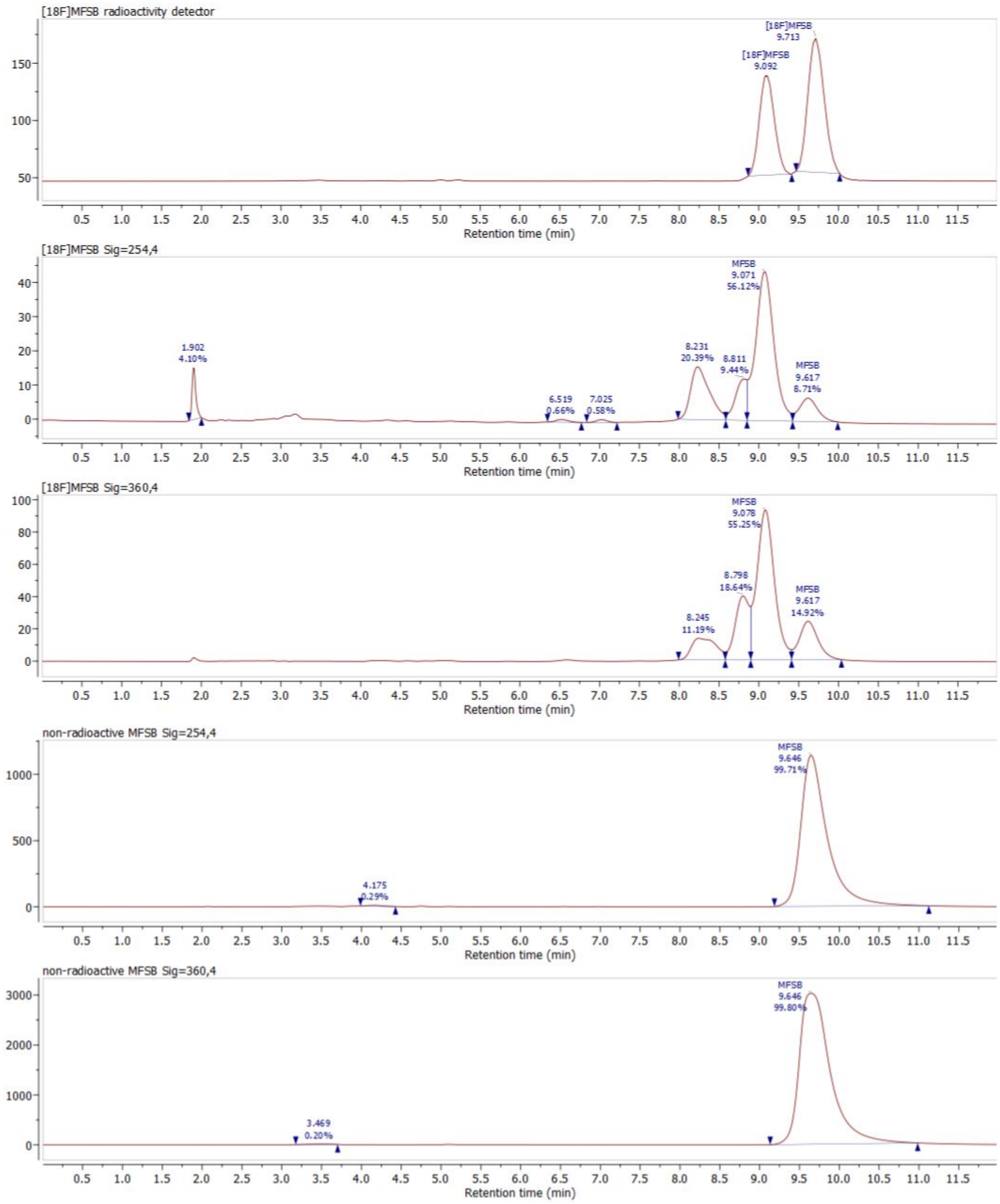

Figure S3. Example of analytical QC HPLC of [^{18}F]MFSB and its non-radioactive reference compound using a Luna column 5 µm C18 (2) 100 Å 250 x 4.6 mm, isocratic method: 72% MeCN in 25 mM ammonium formate at pH 8, 1 mL/min. The ratio between the two peaks (retention time ≈ 9.1 and 9.7 min) is dependent on the E/Z isomer ratio.

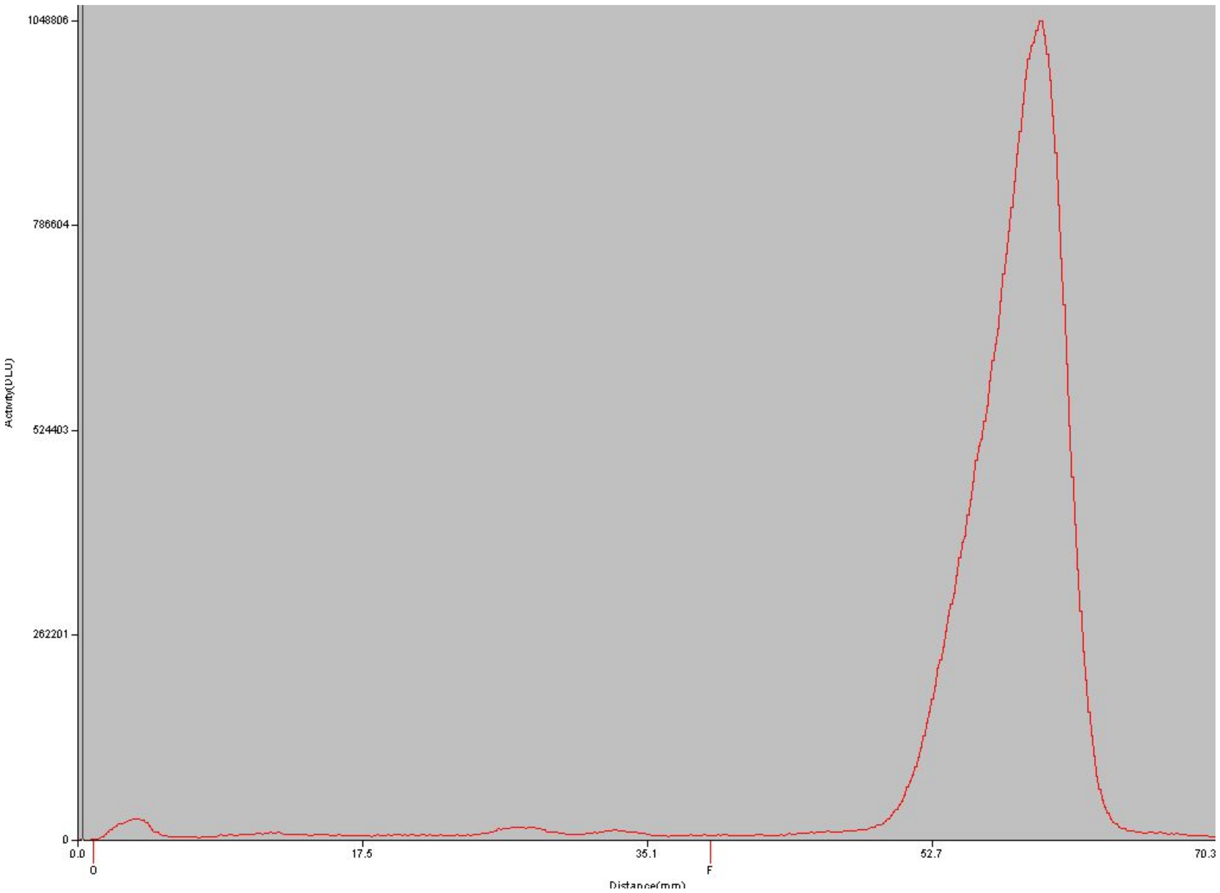

Figure S4. Example of radioTLC of [^{18}F]MFSB, eluent: PE/EtOAc 1:1.

3. Biological evaluation

Table S2. Chemical properties required for the calculation of BBB score and CNS MPO for all non-ionic compounds.

#	cLogP	TPSA (Å²)	MW (g/mol)	pKa	aromatic rings	heavy atoms	Hydrogen bond acceptors	Hydrogen bond donors	BBB score	CNS MPO
**1a**	6.15	16.13	334.48	5.30	3	24	2	0	4.79	3.0
**2a**	5.48	25.36	350.48	5.30	3	25	3	0	4.90	3.3
**3a**	5.78	16.13	338.44	5.30	3	24	2	0	4.78	3.0
**4a**	6.15	16.13	334.48	5.31	3	24	2	0	4.79	3.0
**5a**	5.48	25.36	350.48	5.31	3	25	3	0	4.90	3.3
**6a**	5.78	16.13	338.44	5.30	3	24	2	0	4.78	3.0
**7a**	6.15	16.13	334.48	5.30	3	24	2	0	4.79	3.0
**8a**	5.48	25.36	350.48	5.30	3	25	3	0	4.90	3.3
**9a**	5.78	16.13	338.44	5.30	3	24	2	0	4.78	3.0
**10a**	6.15	16.13	334.48	5.31	3	24	2	0	4.79	3.0
**11a**	5.48	25.36	350.48	5.31	3	25	3	0	4.90	3.3
**12a**	5.78	16.13	338.44	5.30	3	24	2	0	4.78	3.0
**13a**	5.10	16.13	294.42	4.36	3	21	2	0	4.77	3.0
**14a**	6.16	16.13	346.49	5.37	3	25	2	0	4.76	3.0
**15a**	4.57	25.36	322.42	2.20	3	23	3	0	4.76	3.5
**16a**	5.15	16.13	338.49	3.50	3	23	2	0	4.62	3.0
**17a**	5.19	16.13	306.43	4.89	3	22	2	0	4.81	3.0
**18a**	5.00	16.13	338.44	4.44	3	24	2	0	4.68	3.0
**19a**	4.79	16.13	280.39	4.75	3	20	2	0	4.84	3.1
**20**	5.64	16.13	320.45	5.30	3	23	2	0	4.83	3.0
**43**	6.30	16.13	364.48	5.37	3	26	2	0	4.71	3.0
**44**	5.86	16.13	350.46	4.96	3	25	2	0	4.70	3.0
**45**	4.71	25.36	340.42	2.26	3	24	3	0	4.73	3.4

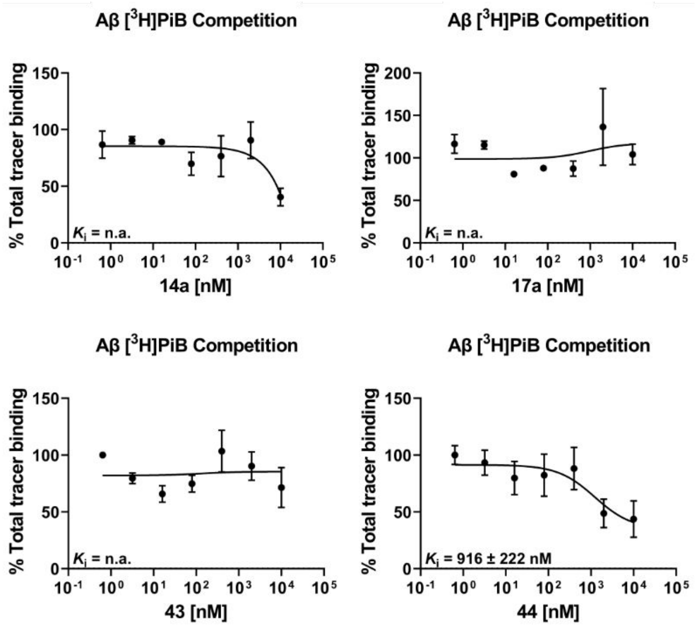

Figure S5. [^{3}H]PiB competition binding assays on Aβ fibrils for compounds **14a**, **17a**, **43**, **44**.

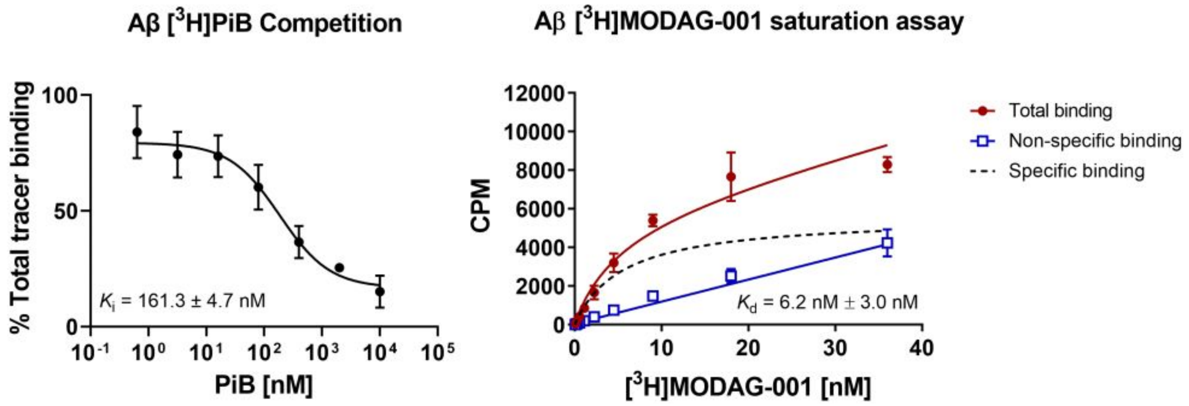

Figure S6. Binding curves for non-radioactive PiB/[^{3}H]PiB competition assay and MODAG-001 saturation assay on in-house produced A$\beta_{1\text{-}42}$ fibrils (K_d value consistent with Kuebler et al., 2020).

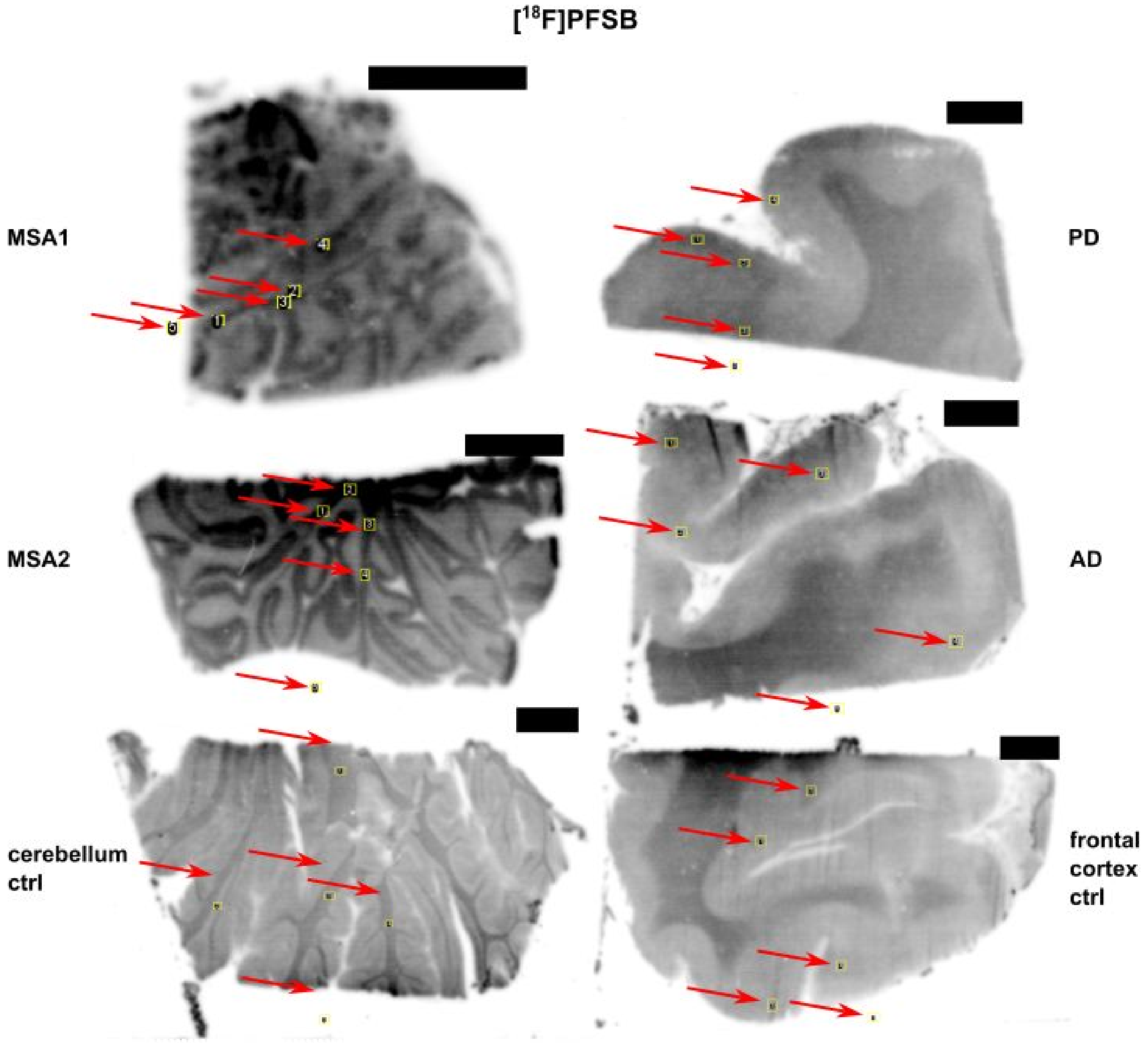

Figure S7. The regions of interest (ROIs) drawn for the quantitative analysis of the [^{18}F]**PFSB** autoradiography. The slices used for the detection of total binding are illustrated in the figure, ROIs were analogously located on the consecutive slice for each case to quantify NSB.

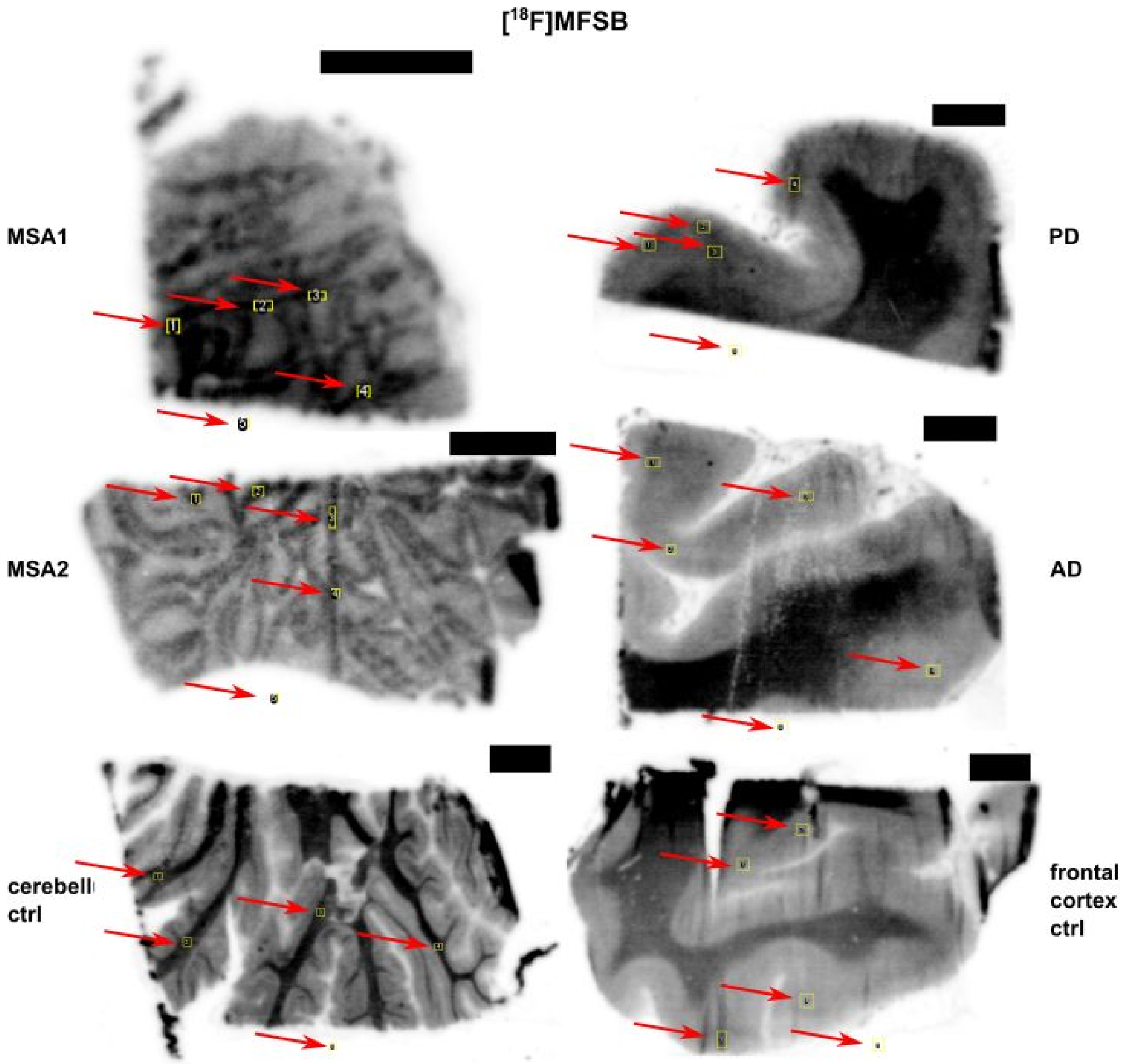

Figure S8. The ROIs drawn for the quantitative analysis of the [^{18}F]**MFSB** autoradiography. The slices used for the detection of total binding are illustrated in the figure, ROIs were analogously located on the consecutive slice for each case to quantify NSB.

Table S3. Quantitative analysis of *in vitro* autoradiography of [^{18}F]PFSB and [^{18}F]MFSB. The accurate quantification of the disease/control SB ratio for MSA sections in the experiment with [^{18}F]MFSB was hampered by high NSB.

**SB disease/SB control**	**[^{18}F]PFSB**	**[^{18}F]MFSB**
MSA1/cerebellum ctrl	3.73	1.20
MSA2/cerebellum ctrl	4.15	1.27
PD/frontal cortex ctrl	1.51	1.18
AD/frontal cortex ctrl	1.06	1.03

Table S4. Overview of the human brain tissues used in the autoradiography and immunohistochemistry experiments.

**Cases**	**Region**	**Age at death (years)**	**Gender**	**PMI (hours)**	**LBD (Braak)**	**AD (Braak & Braak)**	**AD (Thal)**	**Pathology**		
								**αSYN**	**Aβ**	**pTau**
**MSA1**	CB	64	F	52	0	I	1	+++	-	-
**MSA2**	CB	54	M	71	0	0-I	0	+++	-	-
**Cerebellum ctrl**	CB	66	F	27-39	0	I	3	-	-	-
**PD**	FC	79	F	26	6	IV	3	+++	++	+
**AD**	FC	70	M	24	0	VI	5	-	+++	+++
**Frontal cortex ctrl**	FC	64	F	39	0	0	0	-	-	-

The extent of pathology in each subject case was analyzed by Neurobiobank München and is indicated by the symbols “+” and “-”. The number of “+” symbols indicates increasing pathology from low (+), moderate (++) to high (+++); “-” symbolizes the absence of pathology. Abbreviations: Aβ, β-amyloid; αSYN, α-synuclein; AD, Alzheimer’s disease; CB, cerebellum; Ctrl, control; F, female; FC, frontal cortex; LBD, Lewy body dementia; M, male; MSA, multiple system atrophy; PD, Parkinson’s disease; PMI, postmortem interval; pTau, phospho-Tau.

Table S5. Summary of the time-activity curves (TACs) resulting the analysis of the *in vivo* PET data investigating the pharmacokinetics of [^{18}F]MFSB.

Investigated organ	TAC peak value	Time to peak	AUC
Brain	1.79 ± 0.02	25 min	98.13 ± 1.30
Kidney	2.70 ± 0.27	110 s	102.27 ± 1.33
Lung	4.43 ± 0.33	15 s	51.22 ± 3.94
Liver	5.00 ± 0.35	90 s	188.40 ± 6.15
Blood	6.94 ± 0.71	0.25 s	46.52 ± 4.44
Bone	1.52 ± 0.20	60 min	60.79 ± 4.93

4. HPLC-MS chromatograms of the tested compounds

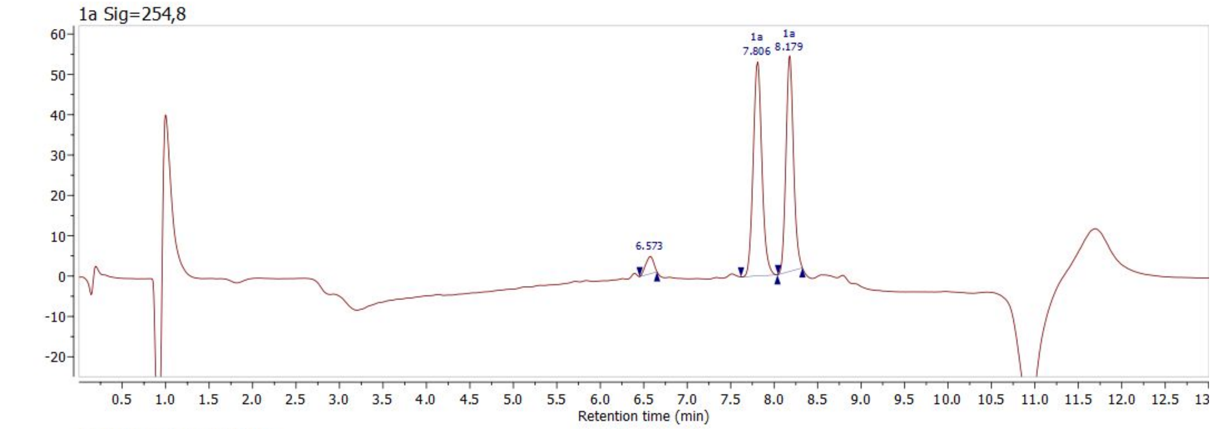

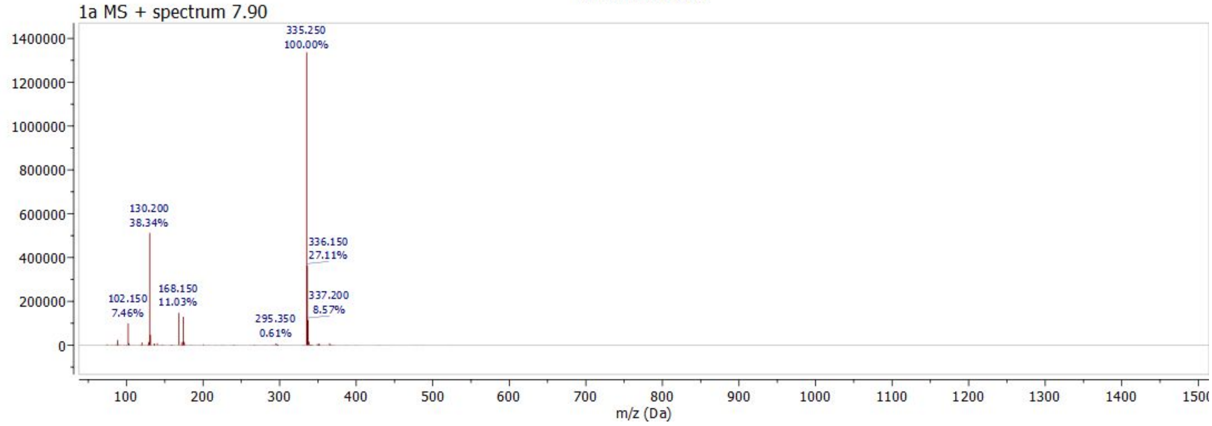

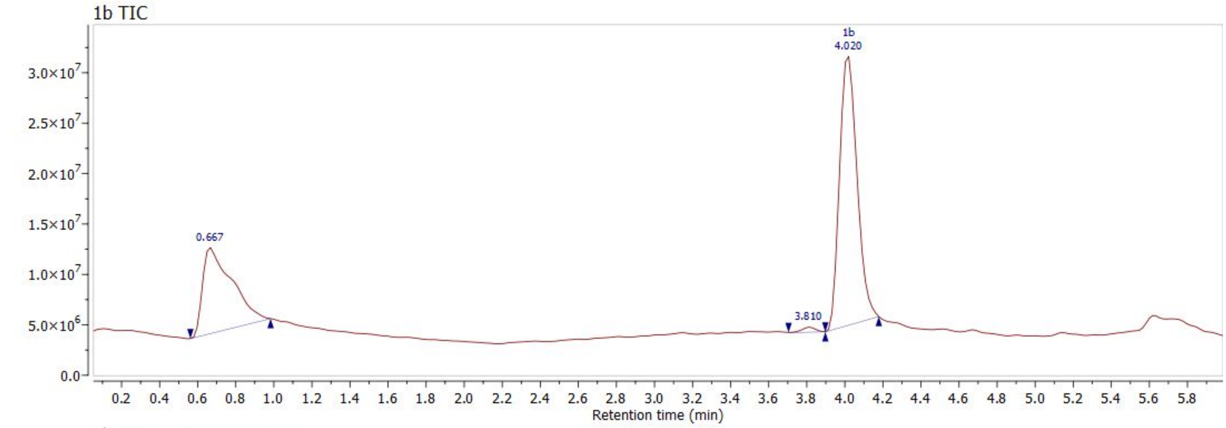

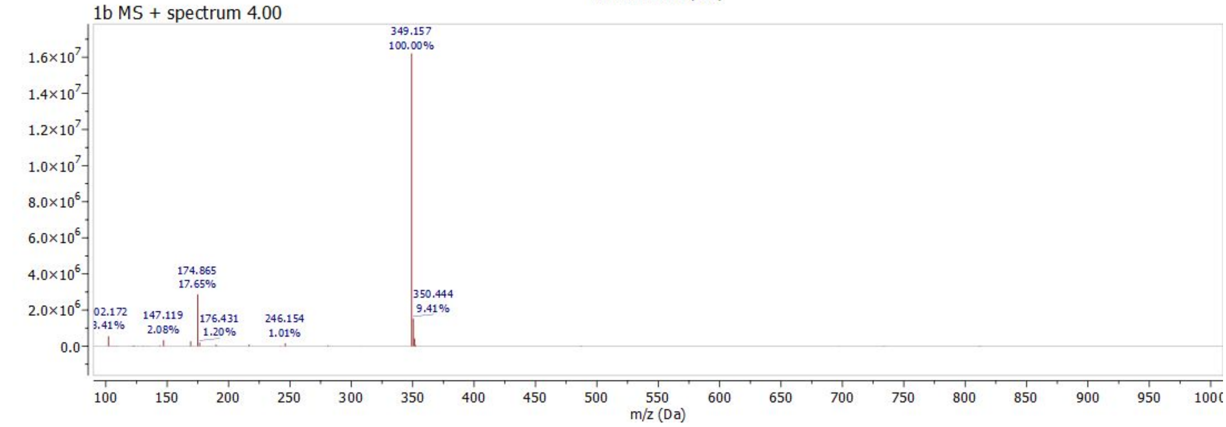

2a TIC

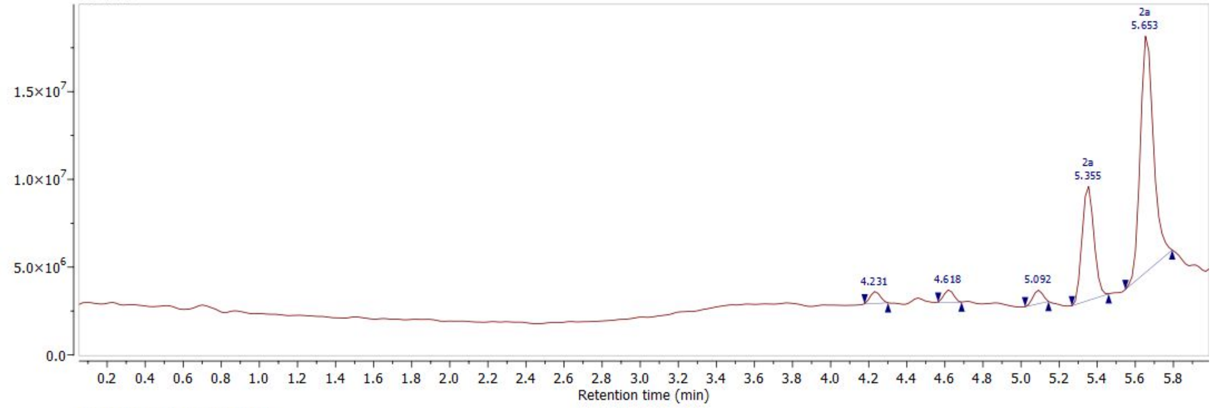

2a MS + spectrum 5.65

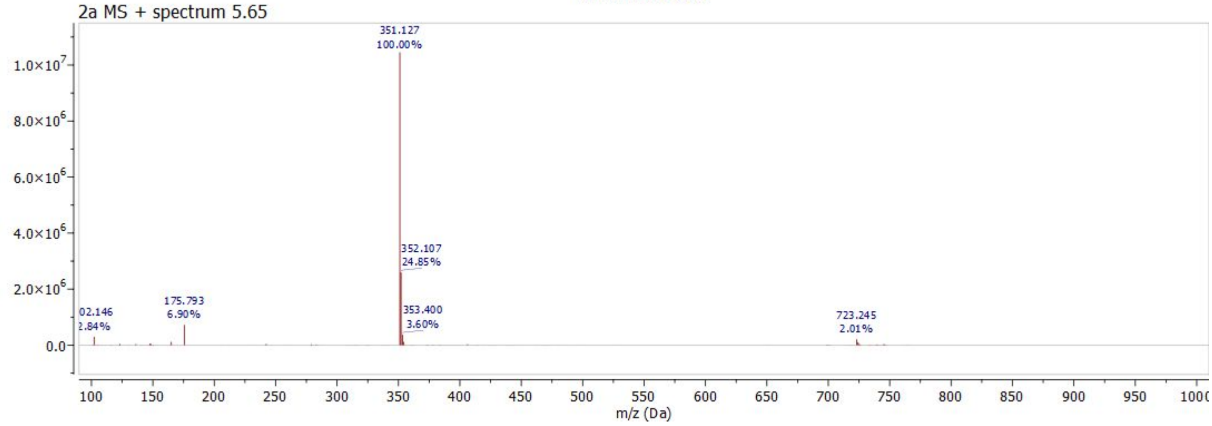

2b Sig=254,8

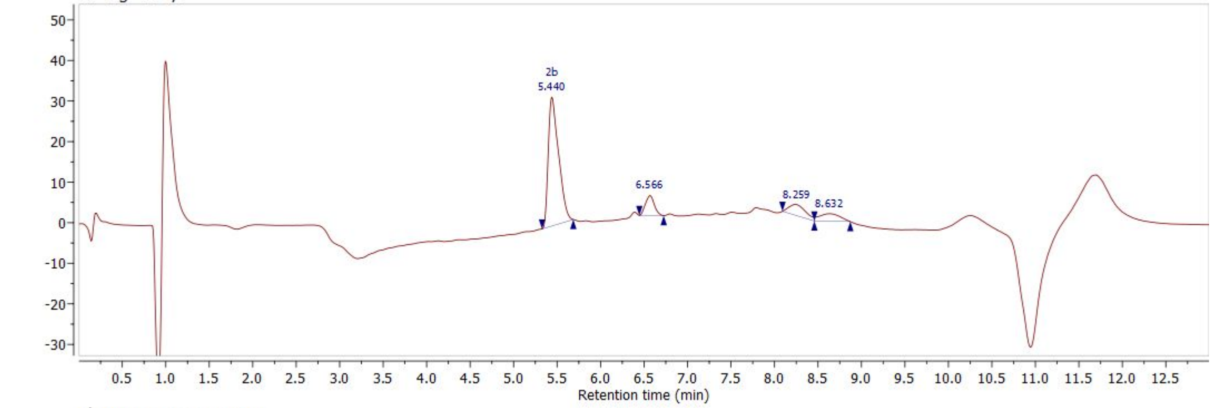

2b MS + spectrum 5.51

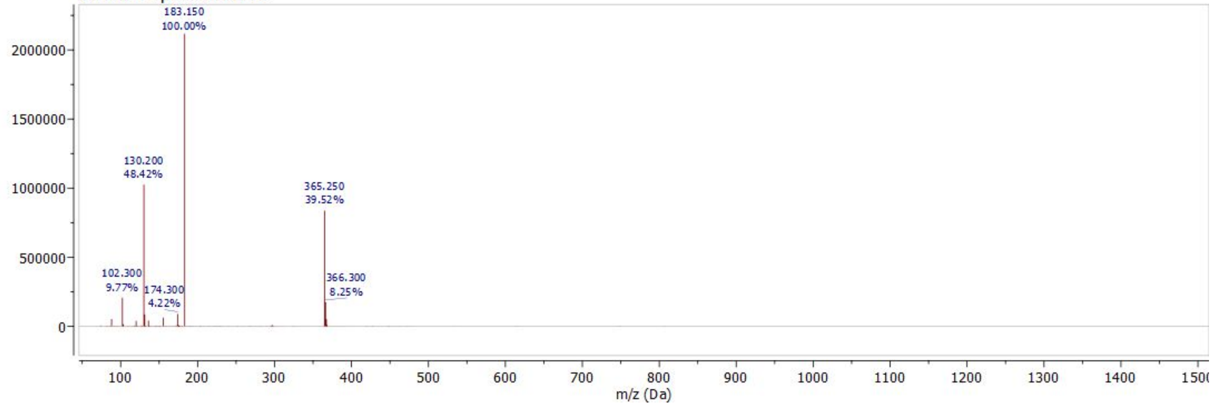

3a TIC

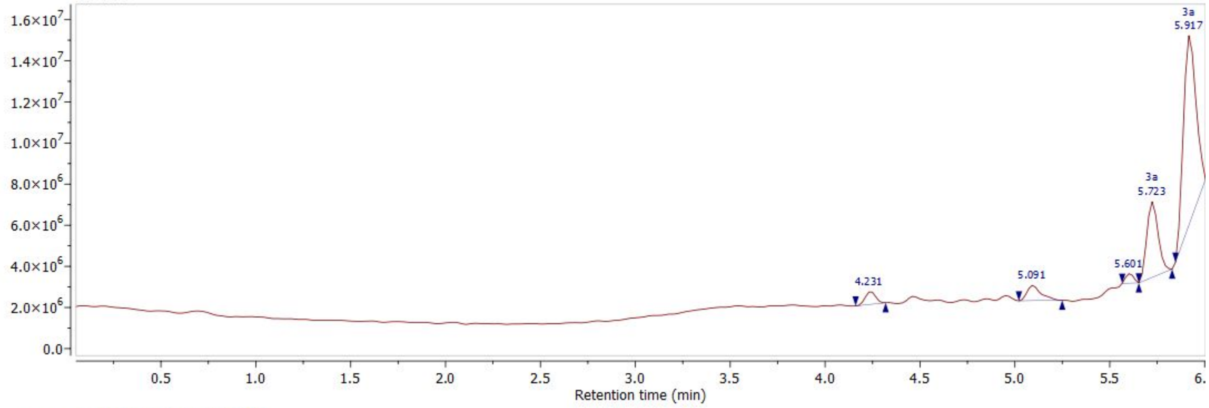

3a MS + spectrum 5.92

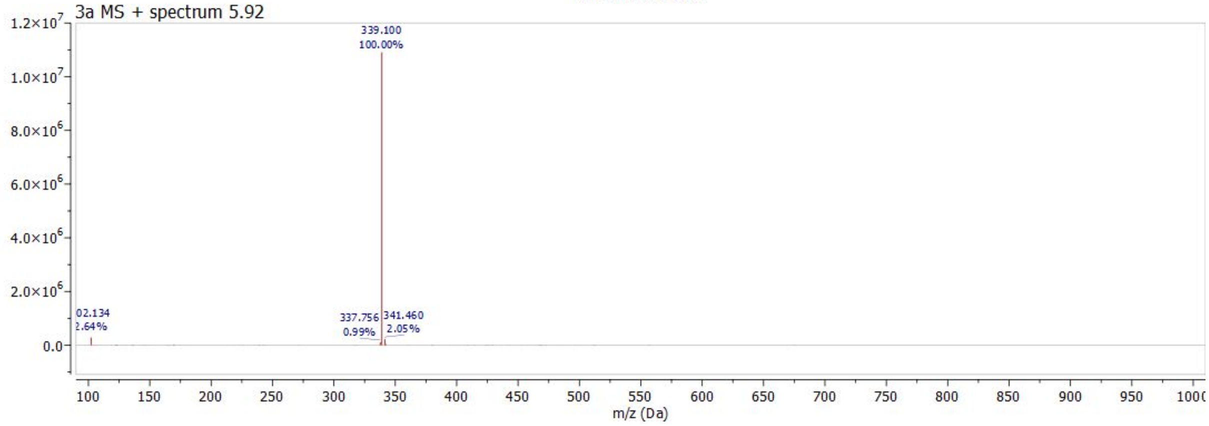

3b TIC

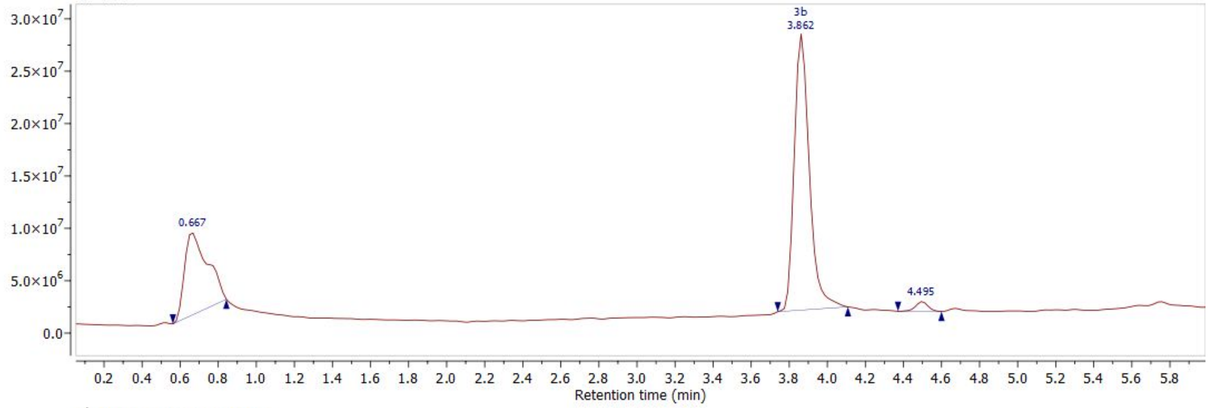

3b MS + spectrum 3.86

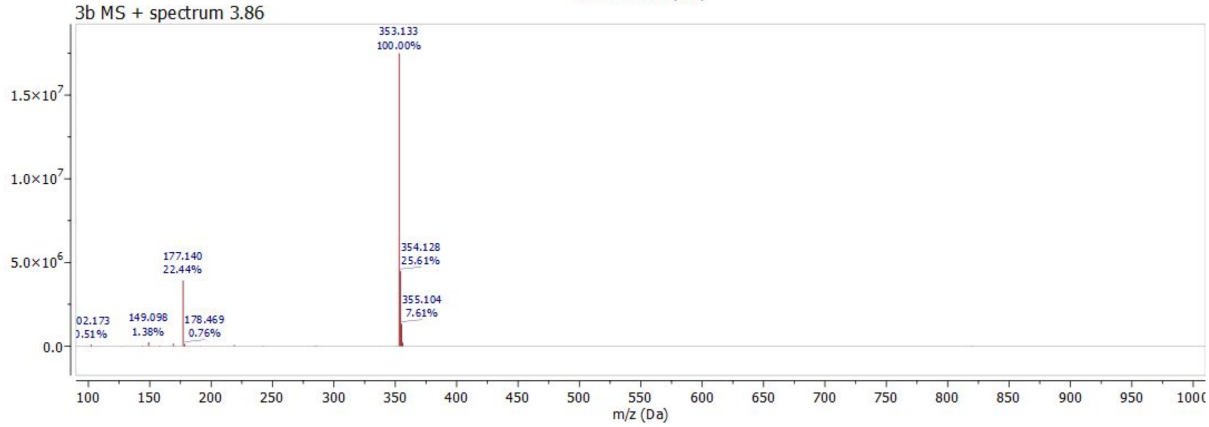

4a Sig=254,8

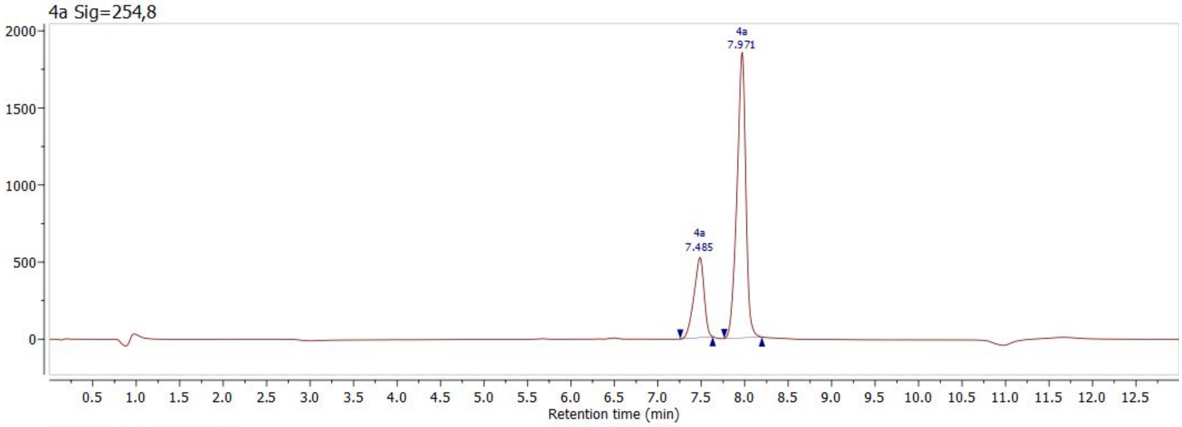

4a MS + spectrum 7.50

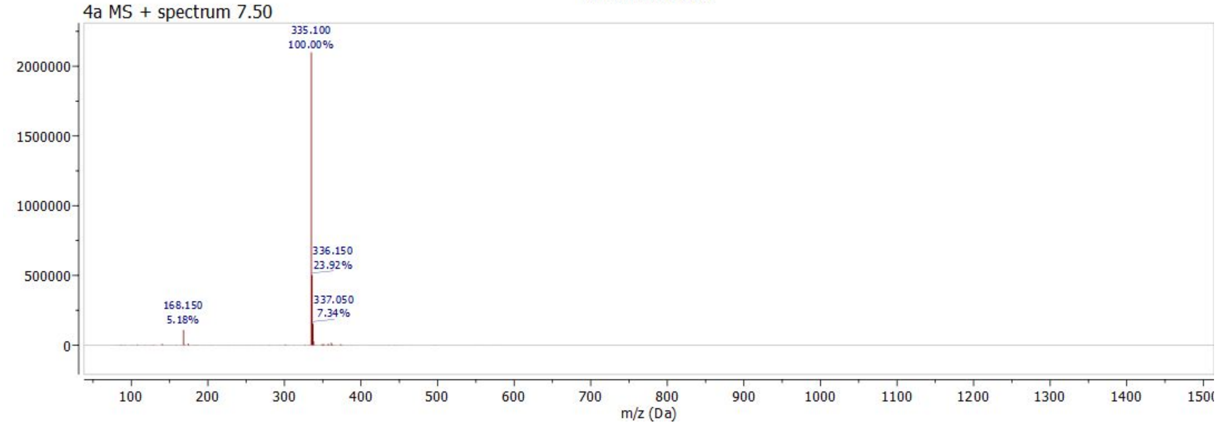

4b TIC

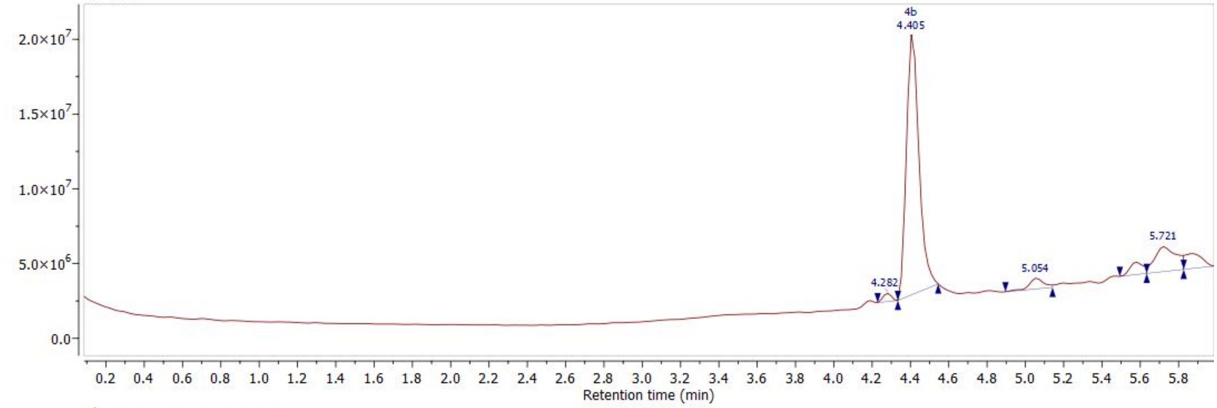

4b MS + spectrum 4.40

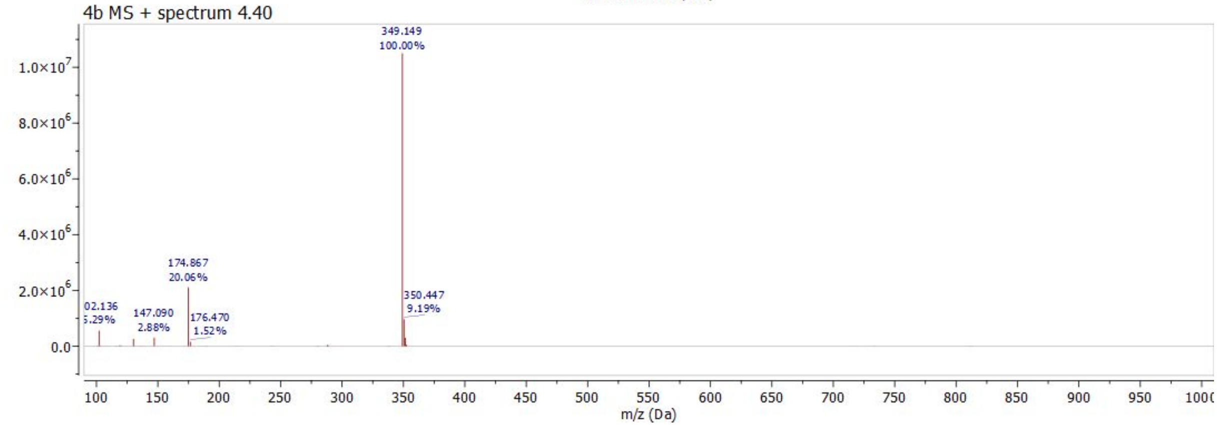

5a Sig=254,8

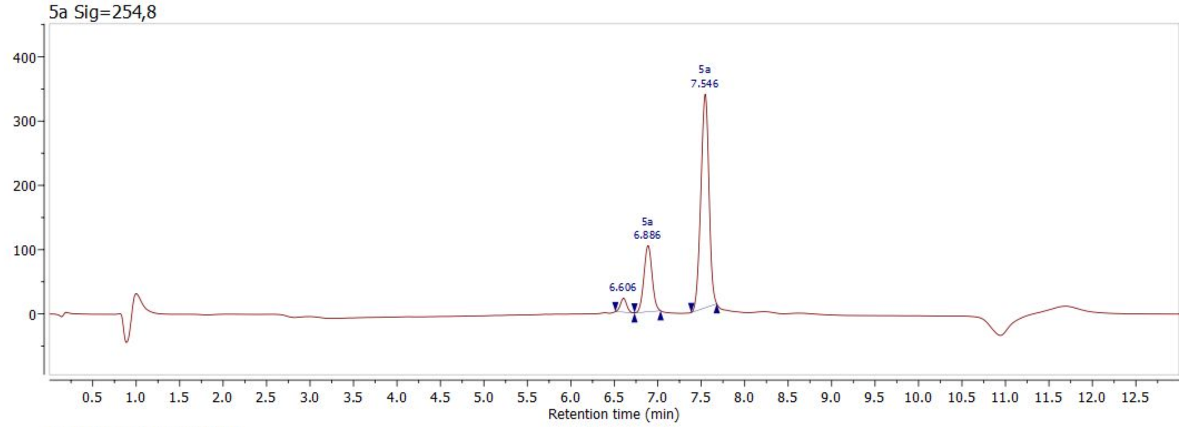

5a MS + spectrum 7.62

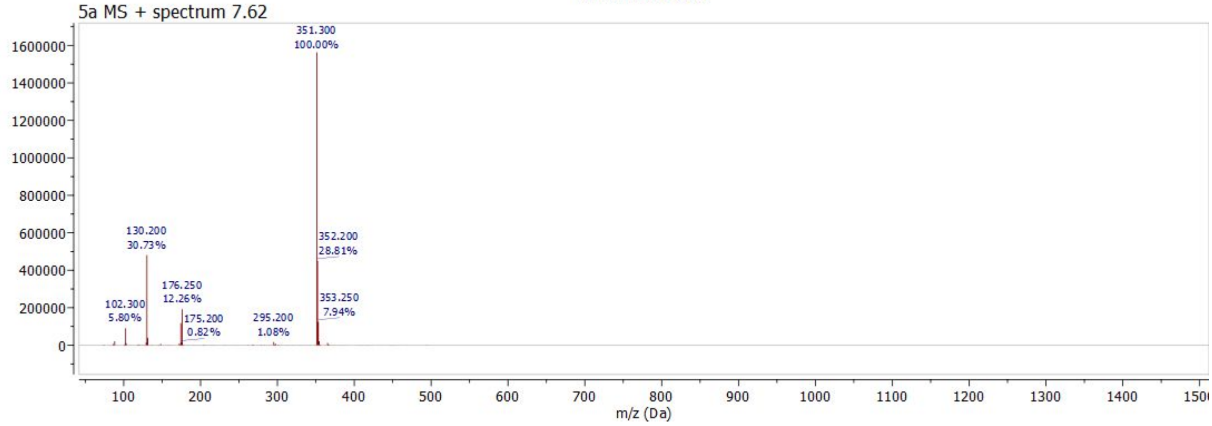

5b Sig=254,8

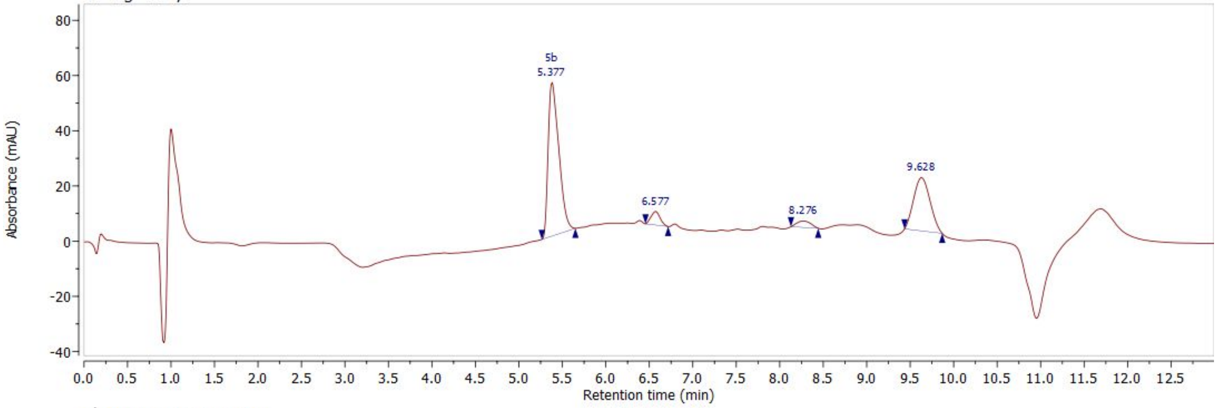

5b MS + spectrum 5.51

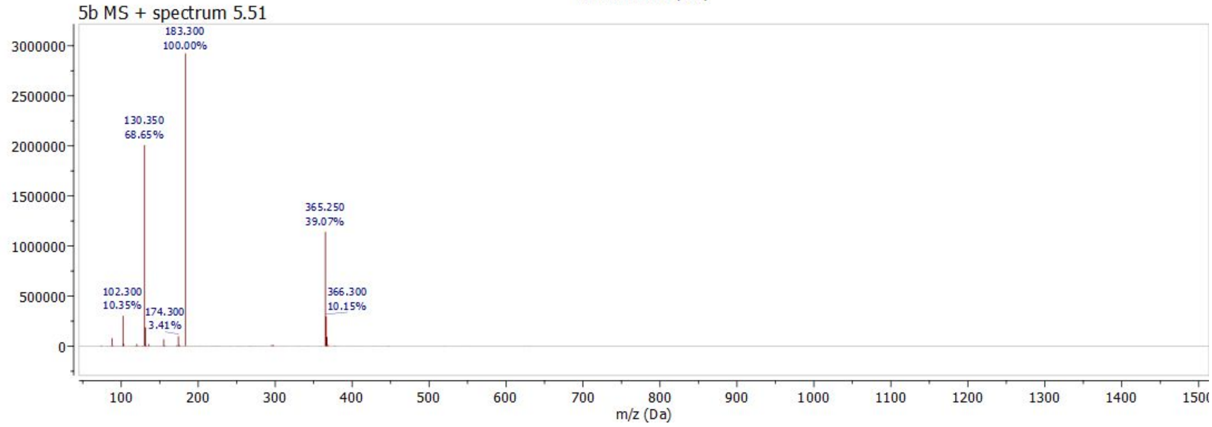

6a TIC

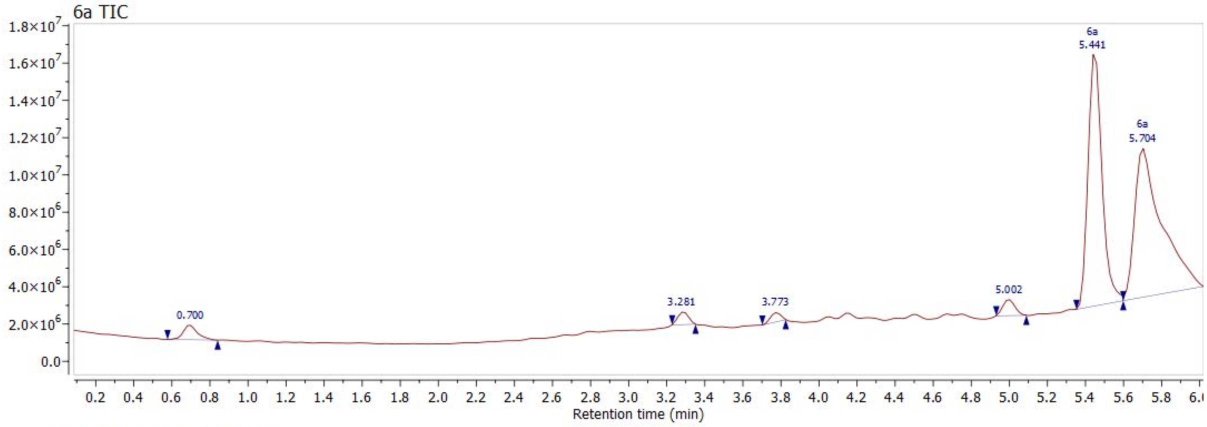

6a MS + spectrum 5.44

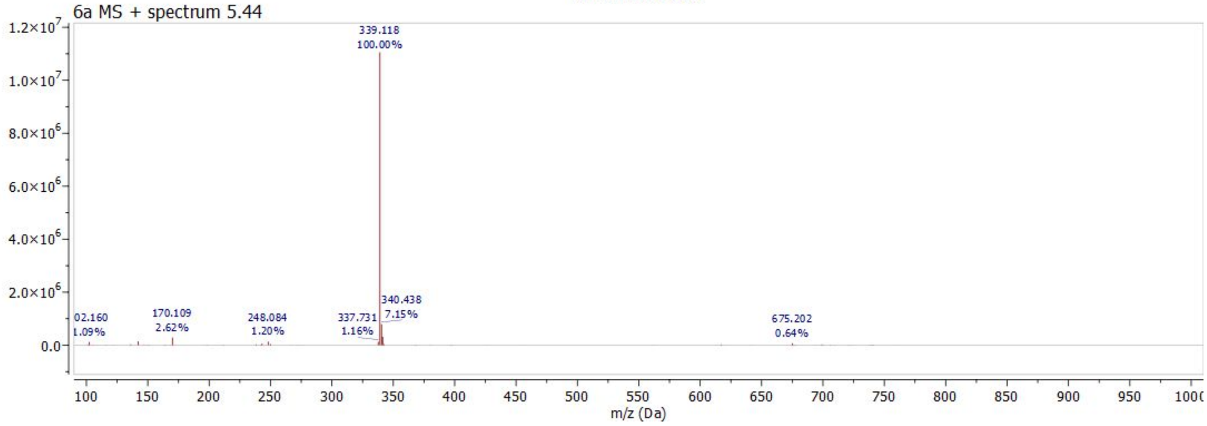

6b TIC

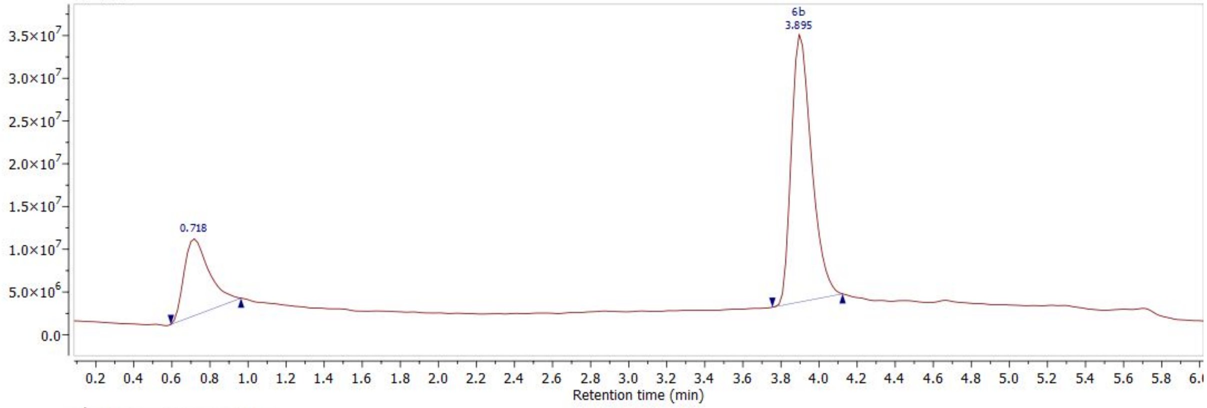

6b MS + spectrum 6.90

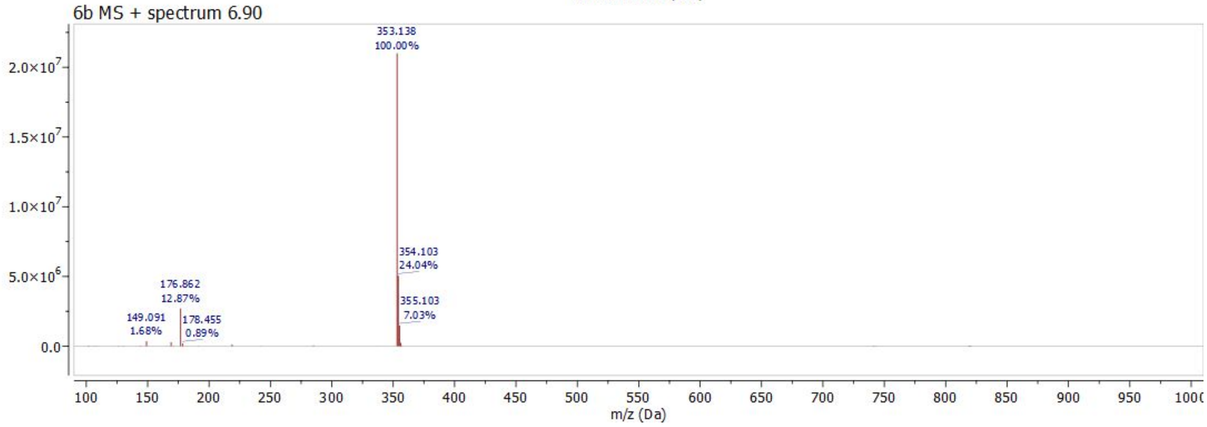

7a Sig=254,8

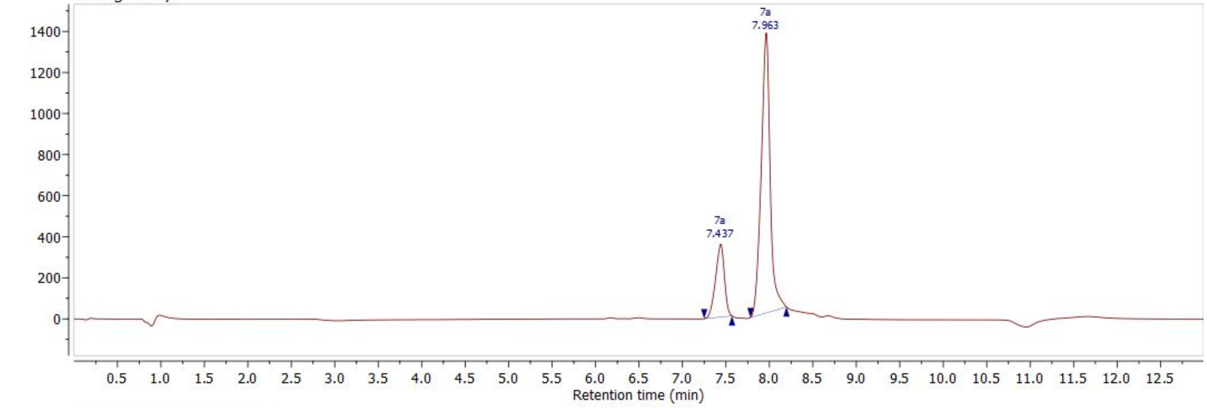

7a MS + spectrum 7.54

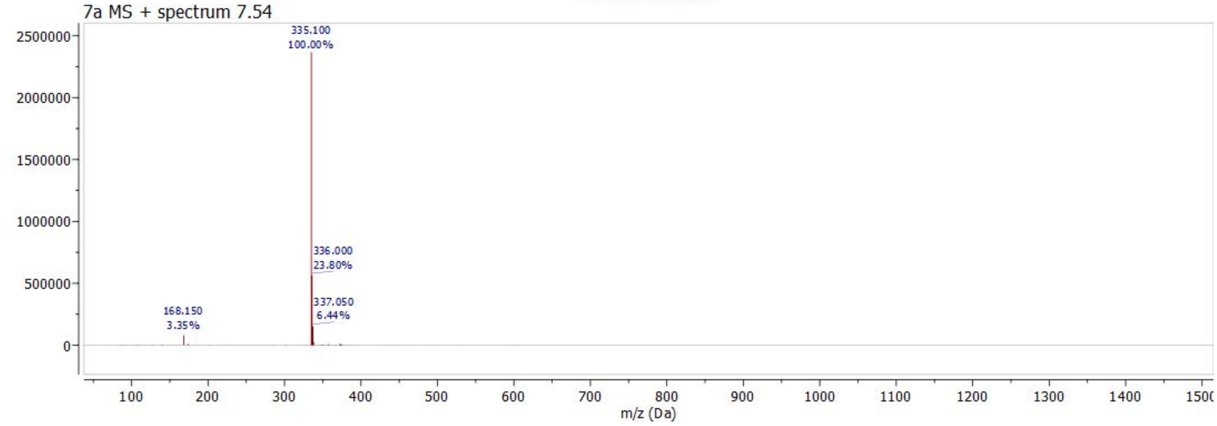

7b TIC

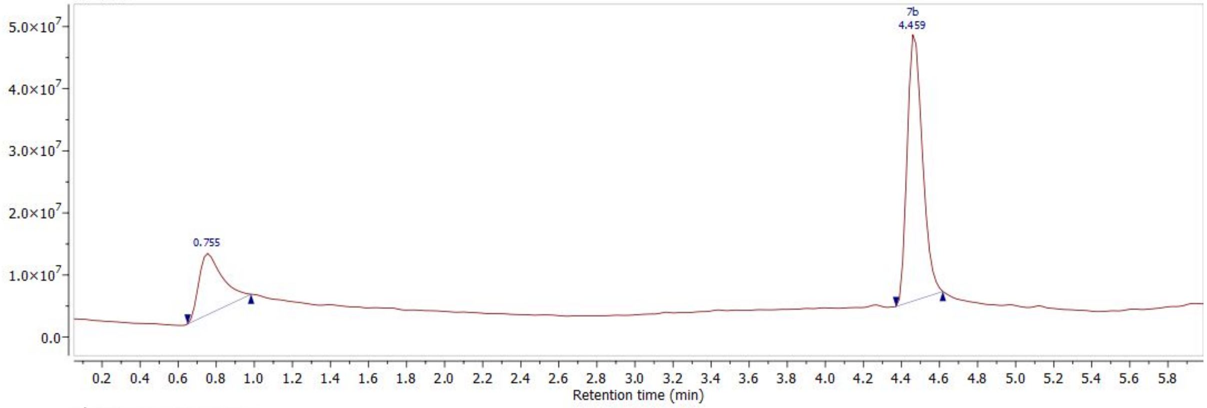

7b MS+ spectrum 4.46

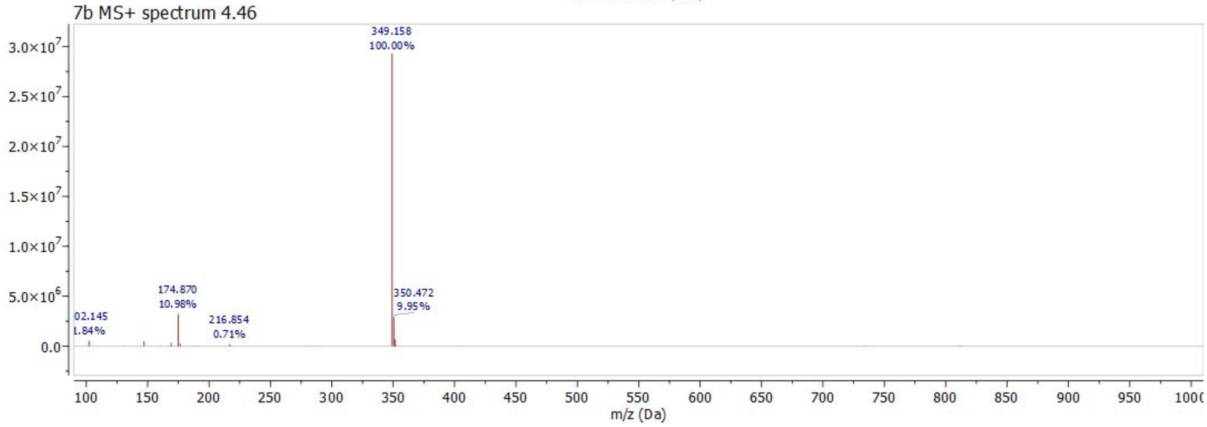

8a Sig=254,8

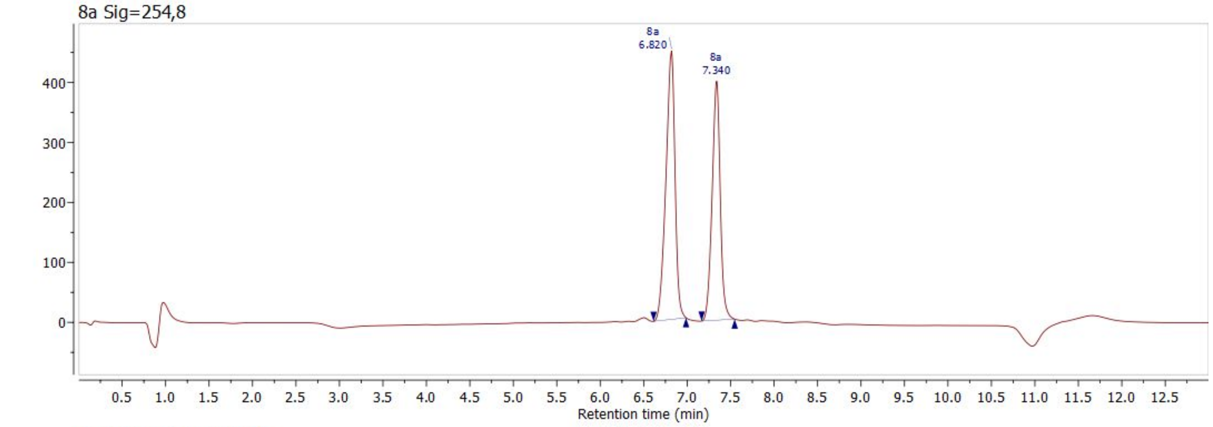

8a MS + spectrum 6.93

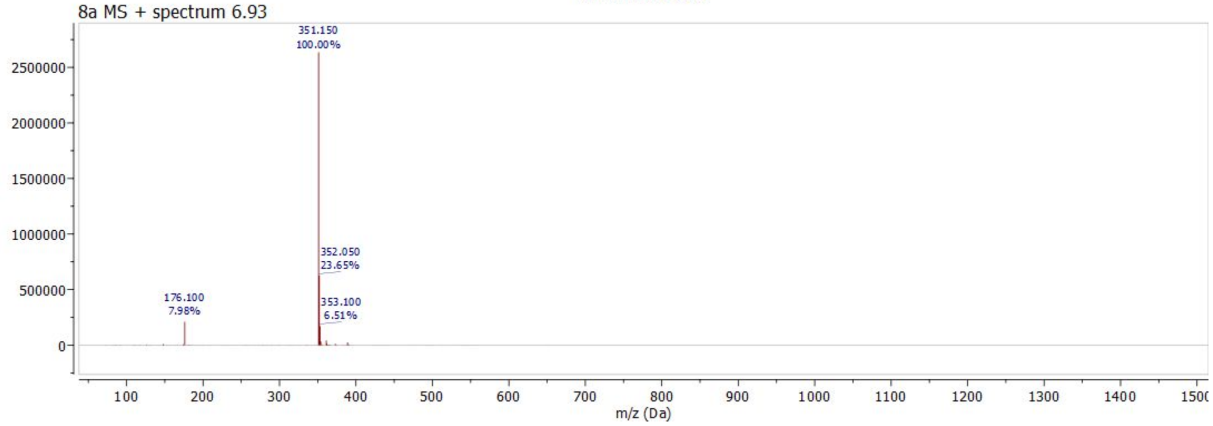

8b TIC

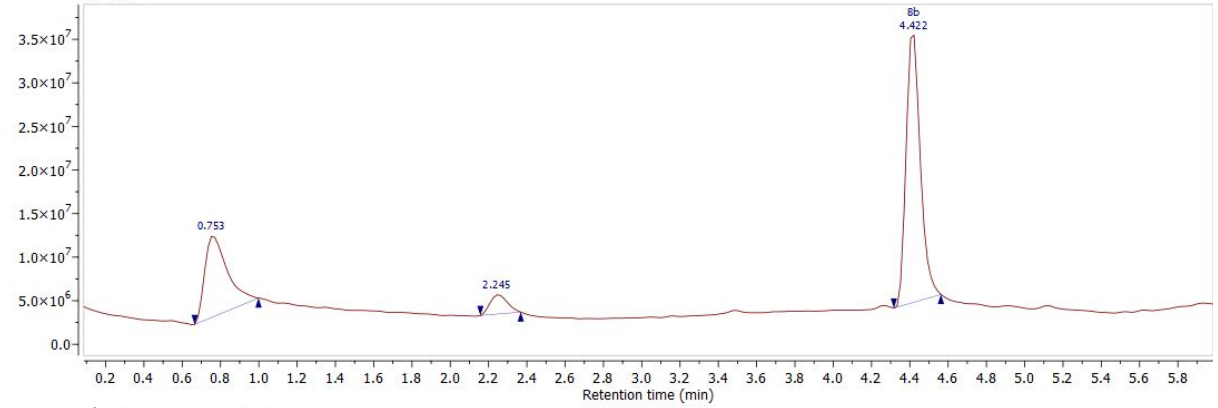

8b MS + spectrum 4.42

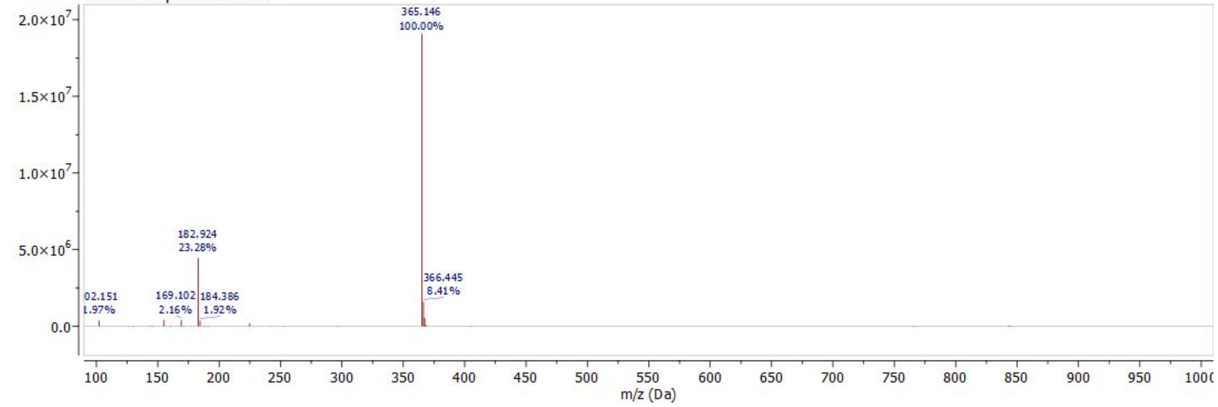

9a (PFSB) Sig=254,8

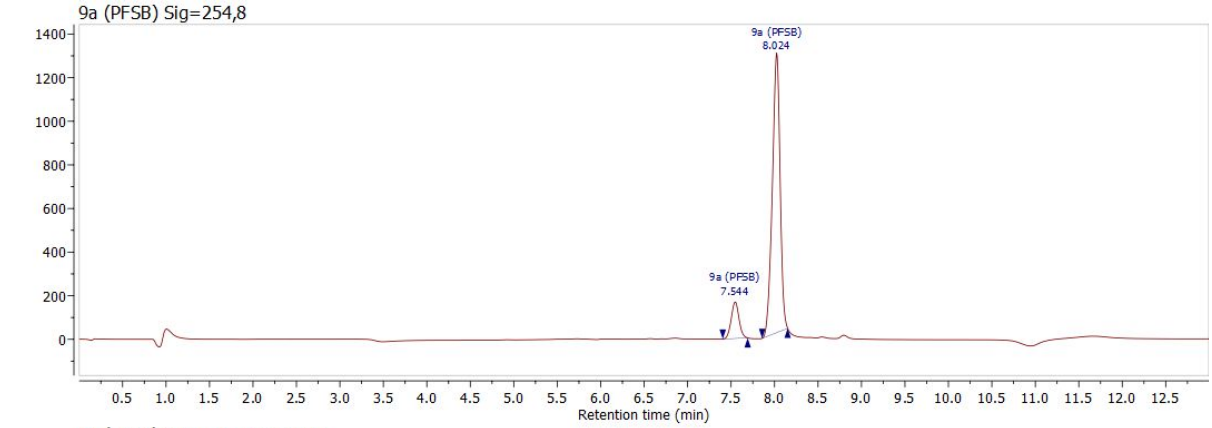

9a (PFSB) MS + spectrum 7.66

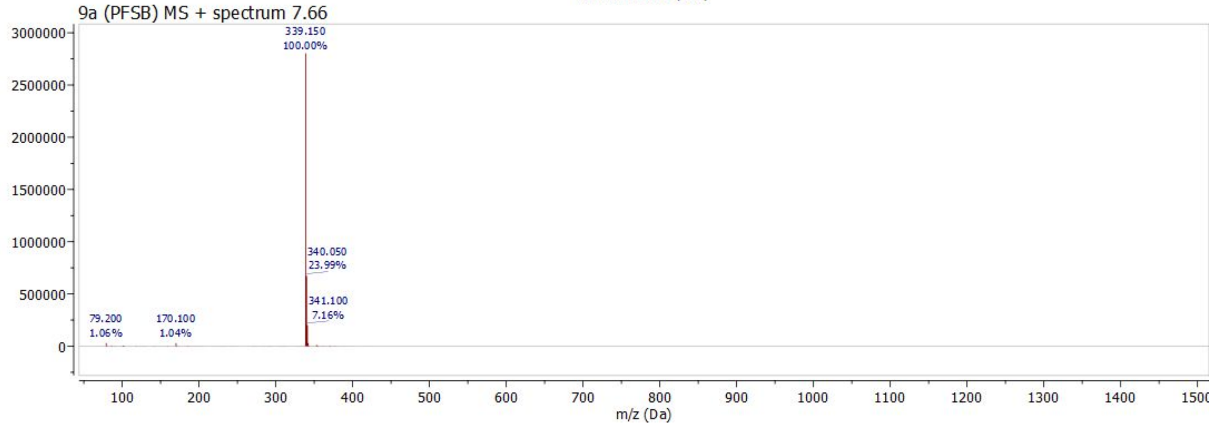

9b TIC

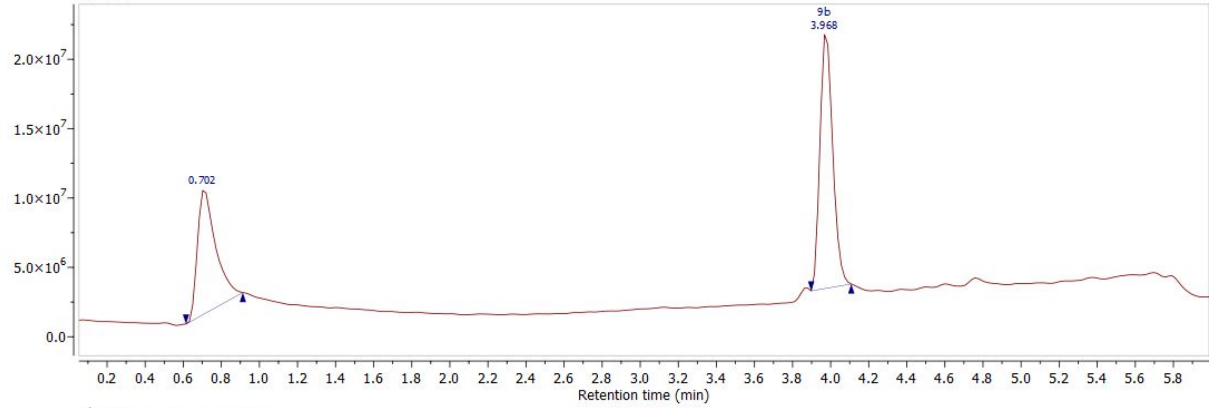

9b MS + spectrum 3.99

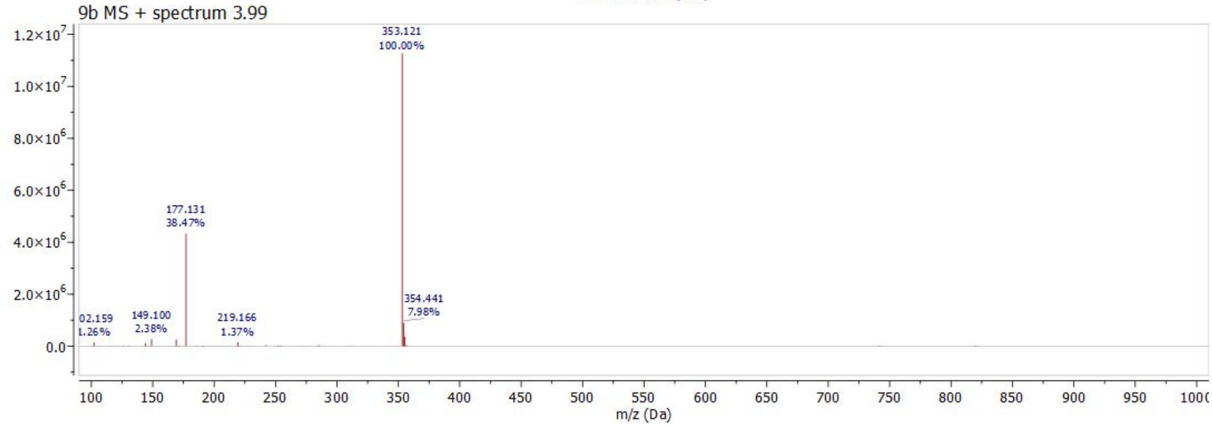

10a TIC

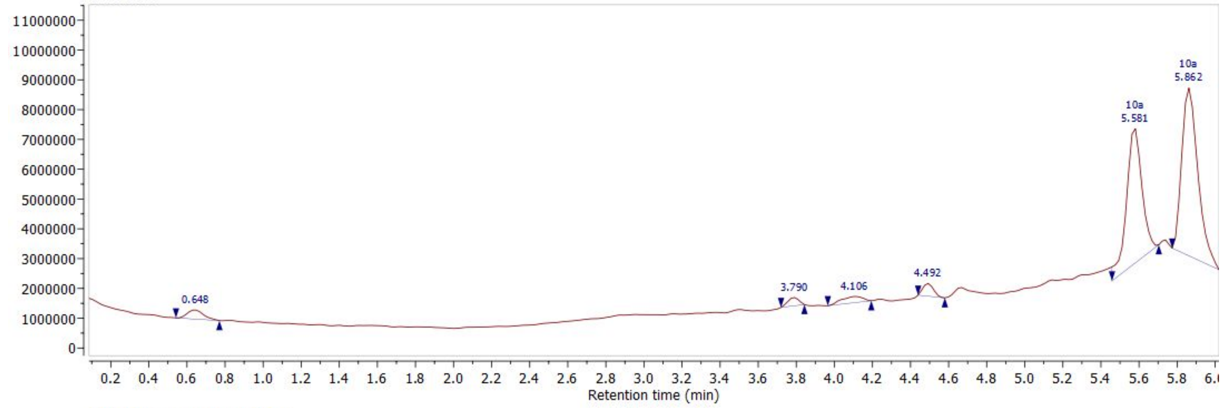

10a MS + spectrum 5.86

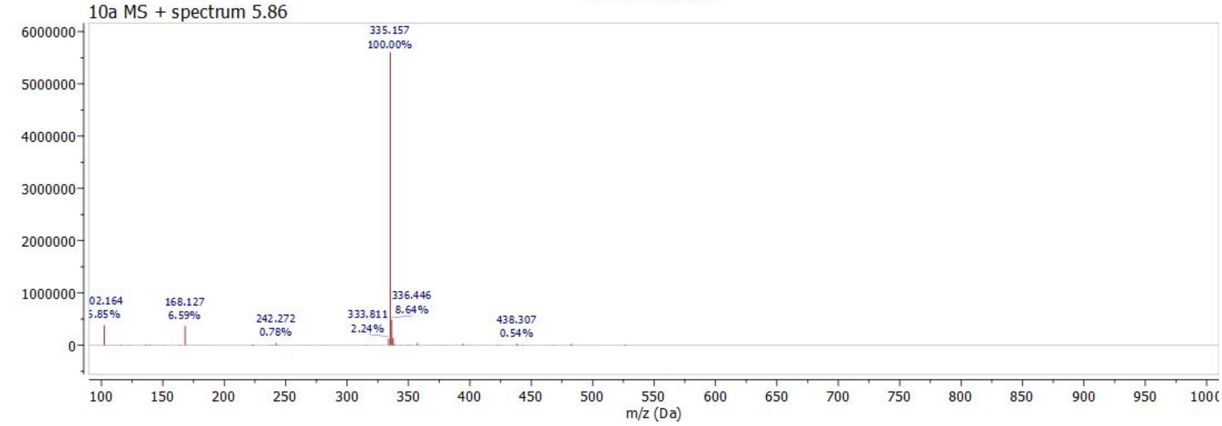

10b Sig=254,8

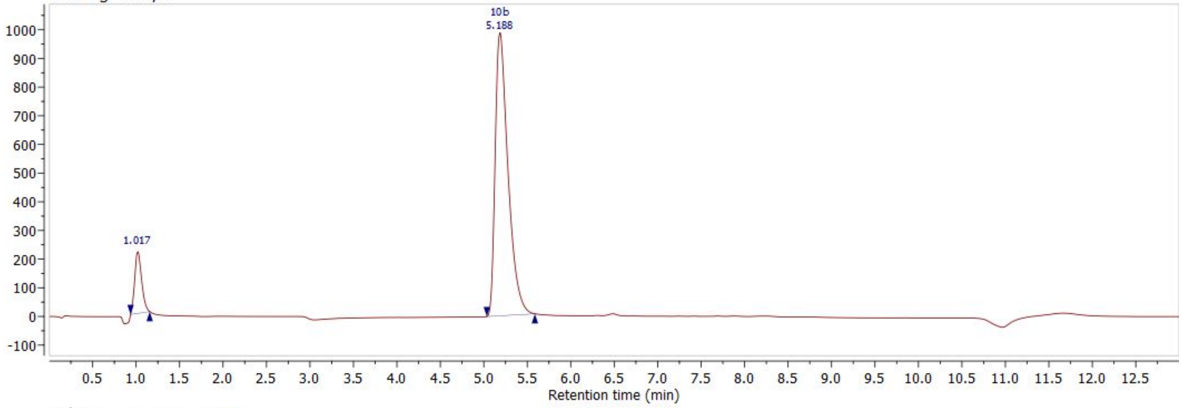

10b MS + spectrum 5.27

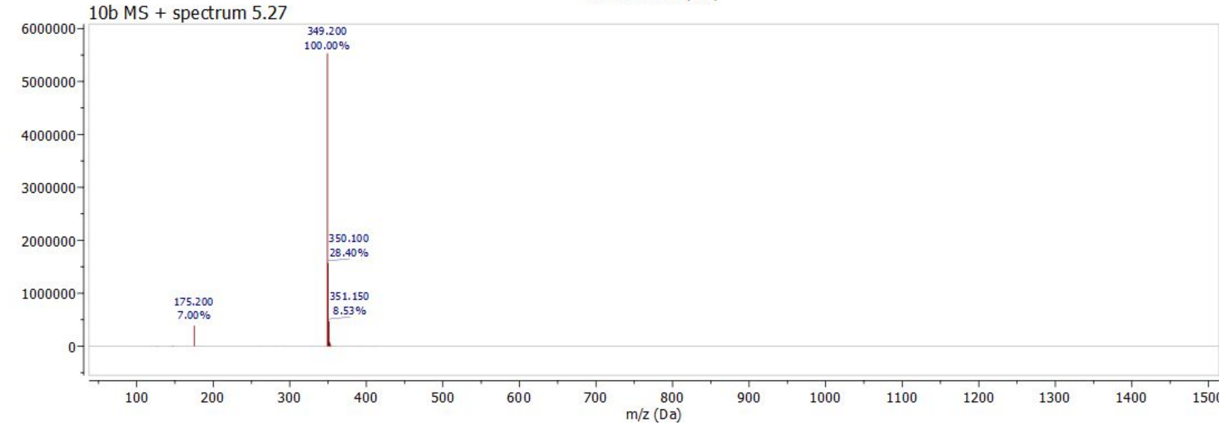

11a Sig=254,8

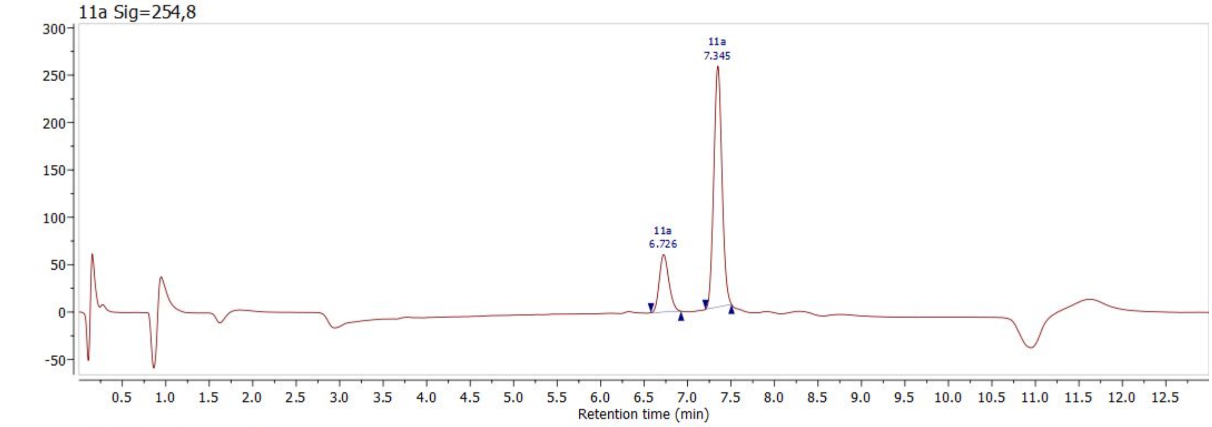

11a MS + spectrum 6.85

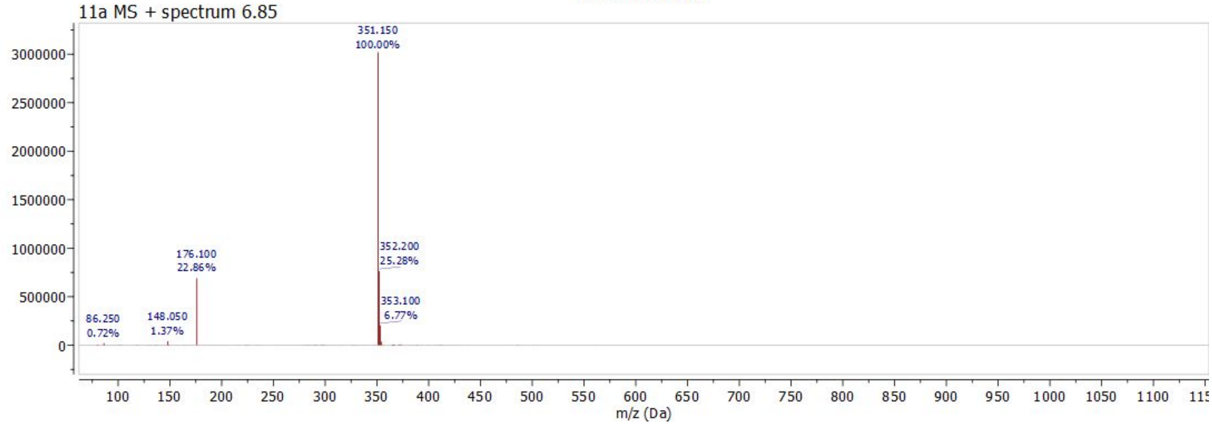

11b TIC

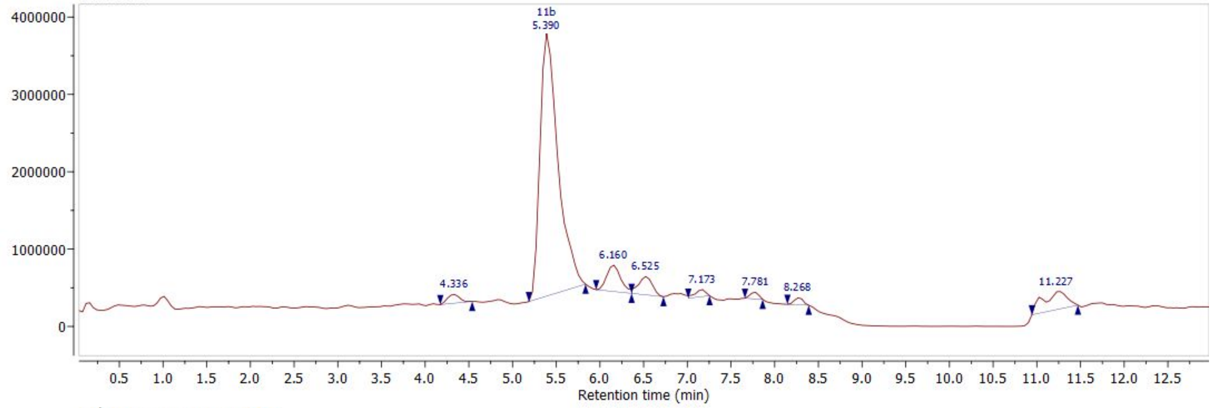

11b MS + spectrum 5.27

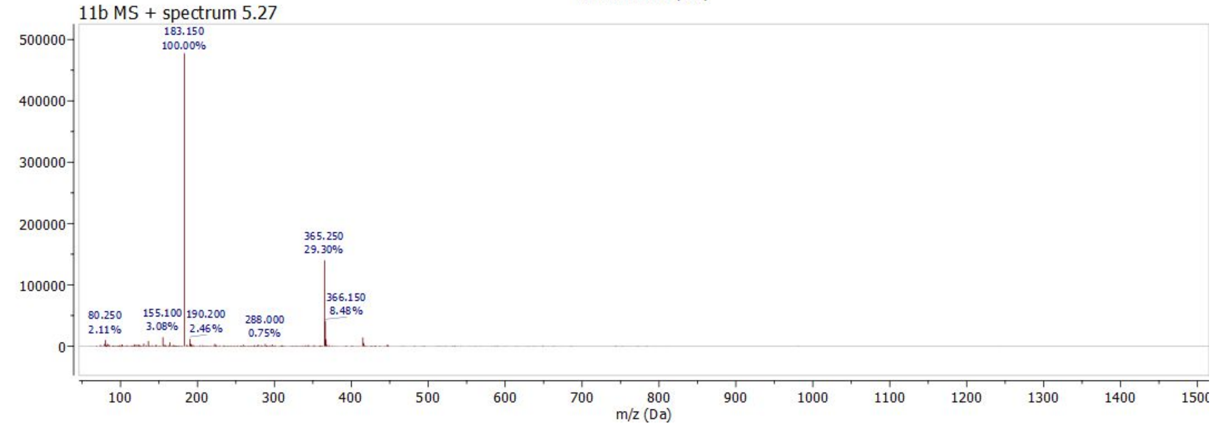

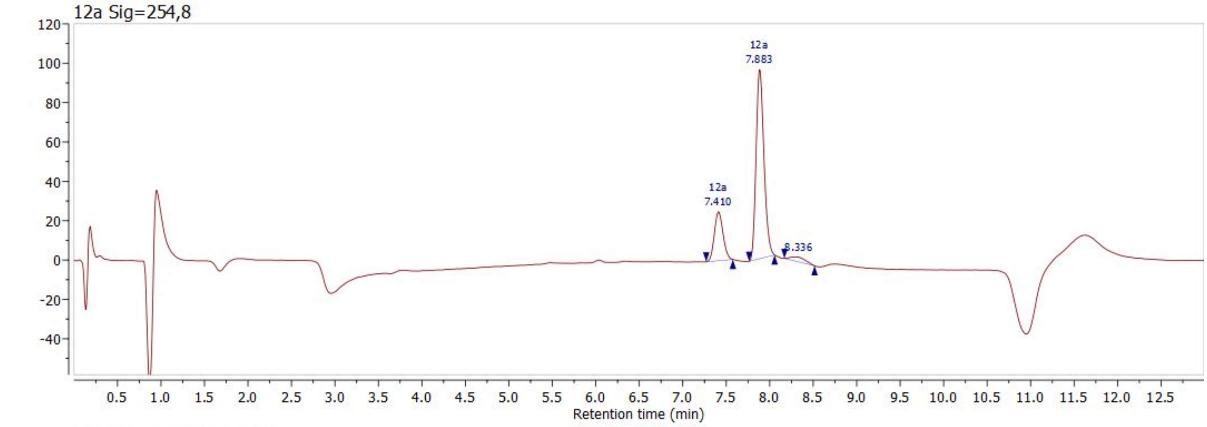

12a Sig=254,8
12a
7.883
12a
7.410
8.336
Retention time (min)

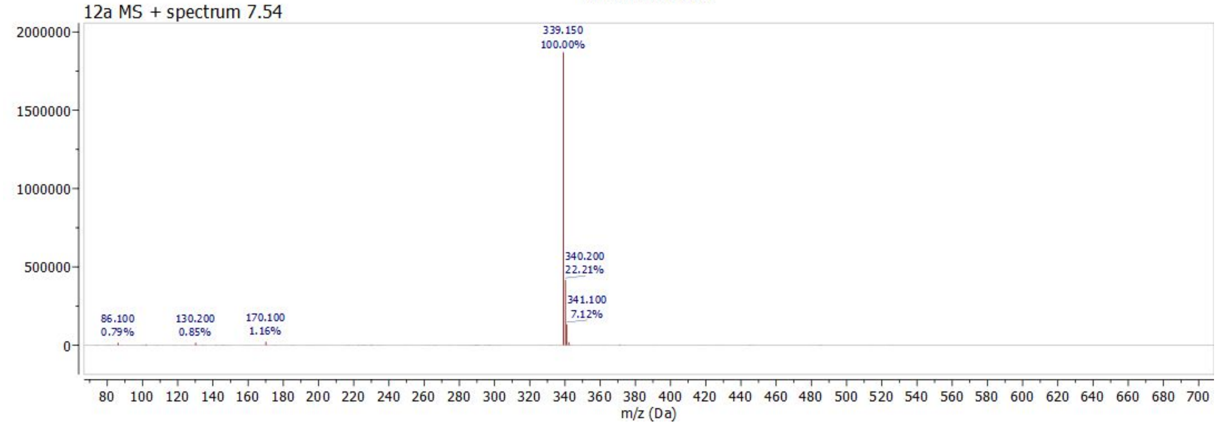

12a MS + spectrum 7.54
339.150
100.00%
340.200
22.21%
341.100
7.12%
86.100
0.79%
130.200
0.85%
170.100
1.16%
m/z (Da)

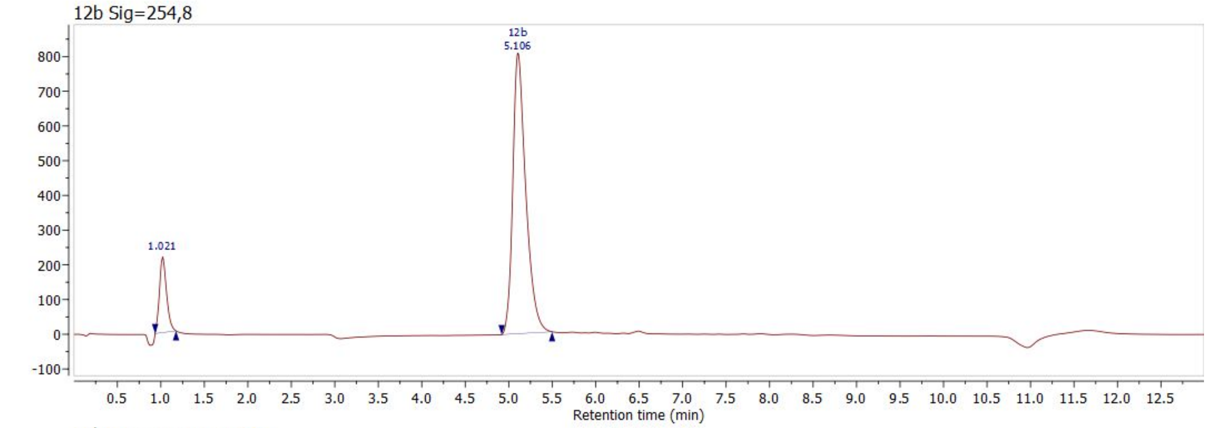

12b Sig=254,8
12b
5.106
1.021
Retention time (min)

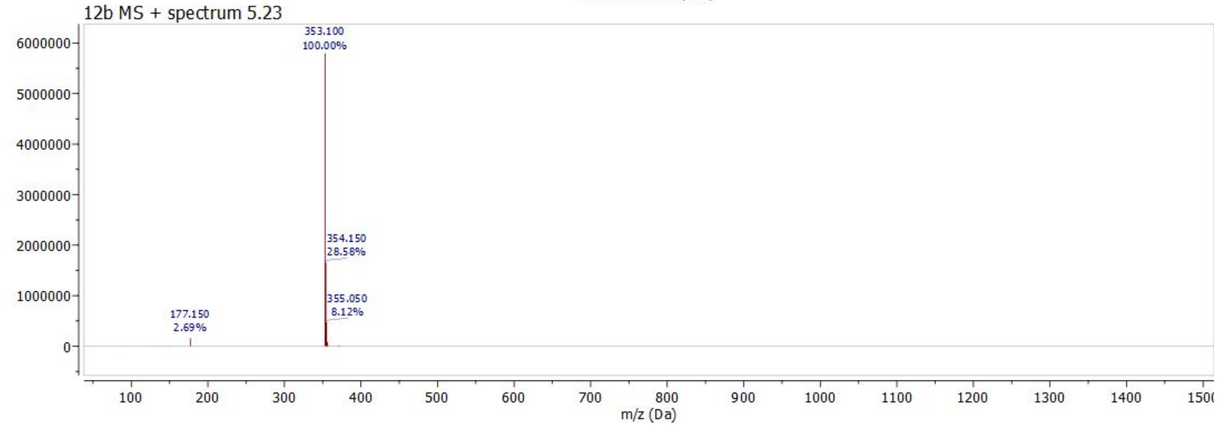

12b MS + spectrum 5.23
353.100
100.00%
354.150
28.58%
355.050
8.12%
177.150
2.69%
m/z (Da)

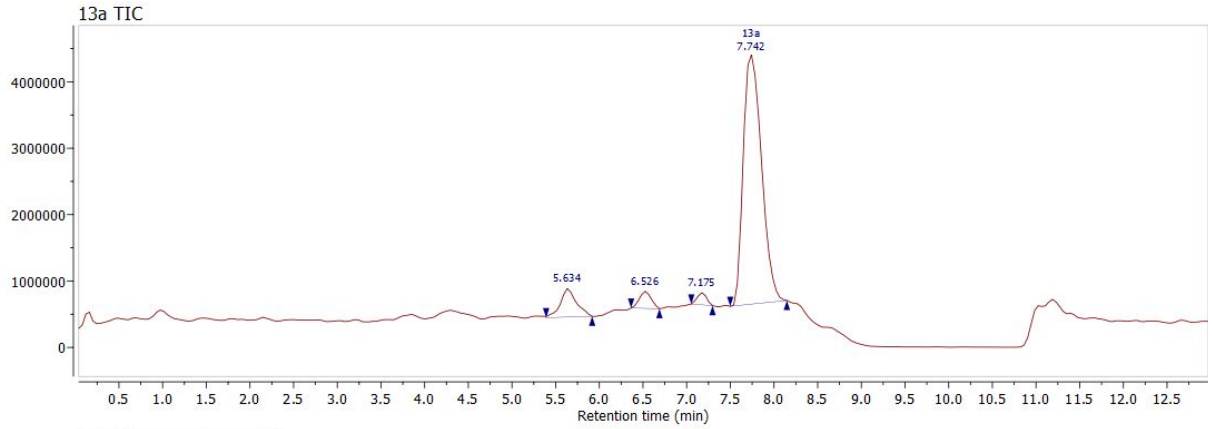
13a TIC
13a
7.742
5.634
6.526
7.175
Retention time (min)

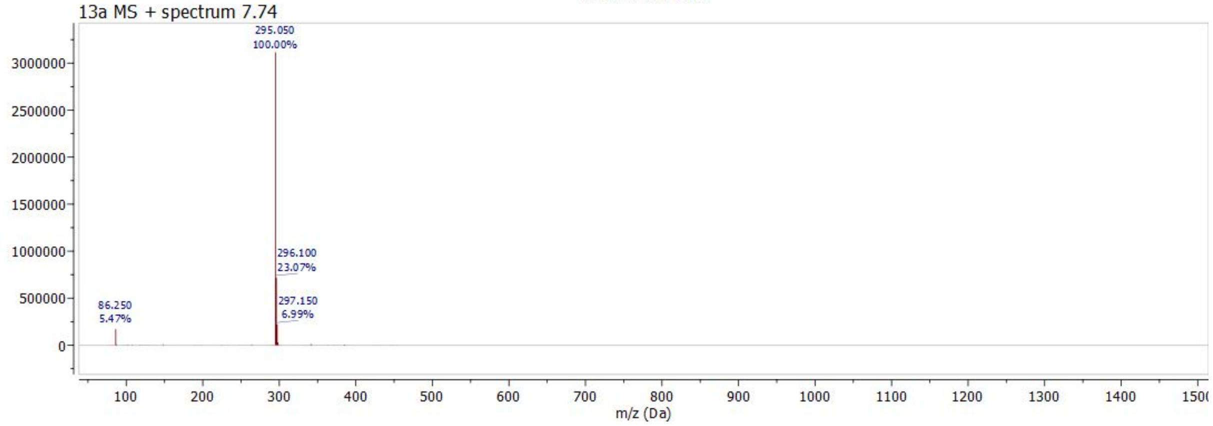
13a MS + spectrum 7.74
295.050
100.00%
296.100
23.07%
297.150
6.99%
86.250
5.47%
m/z (Da)

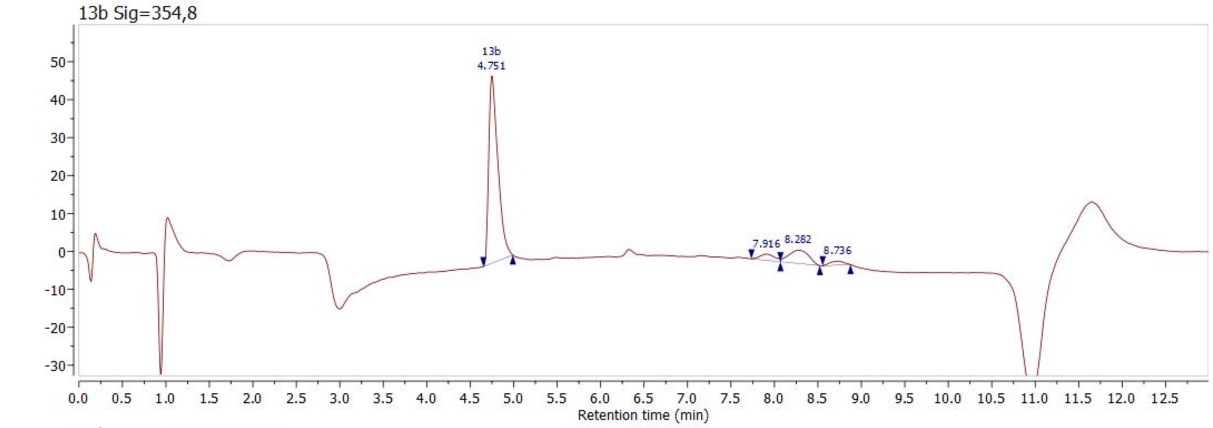
13b Sig=354,8
13b
4.751
7.916
8.282
8.736
Retention time (min)

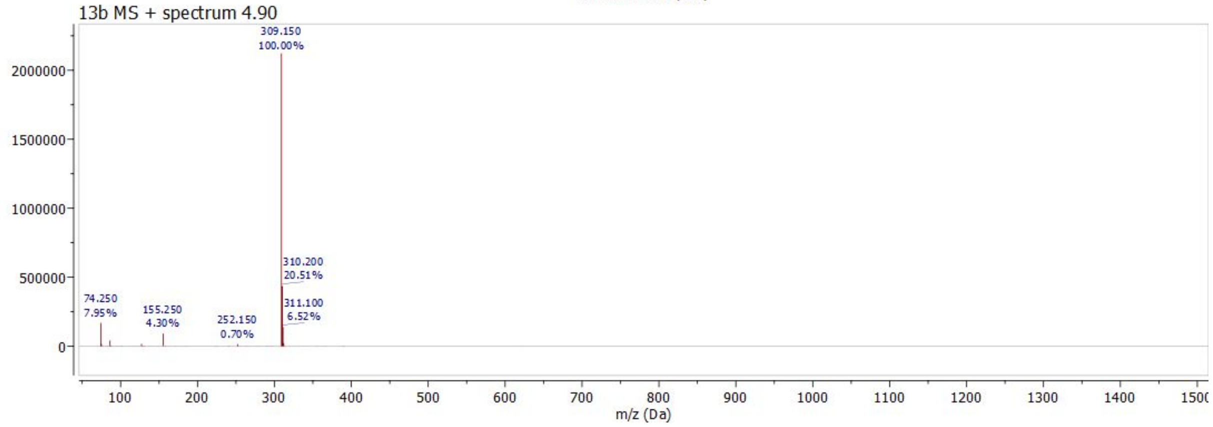
13b MS + spectrum 4.90
309.150
100.00%
310.200
20.51%
311.100
6.52%
74.250
7.95%
155.250
4.30%
252.150
0.70%
m/z (Da)

14a Sig=254,8

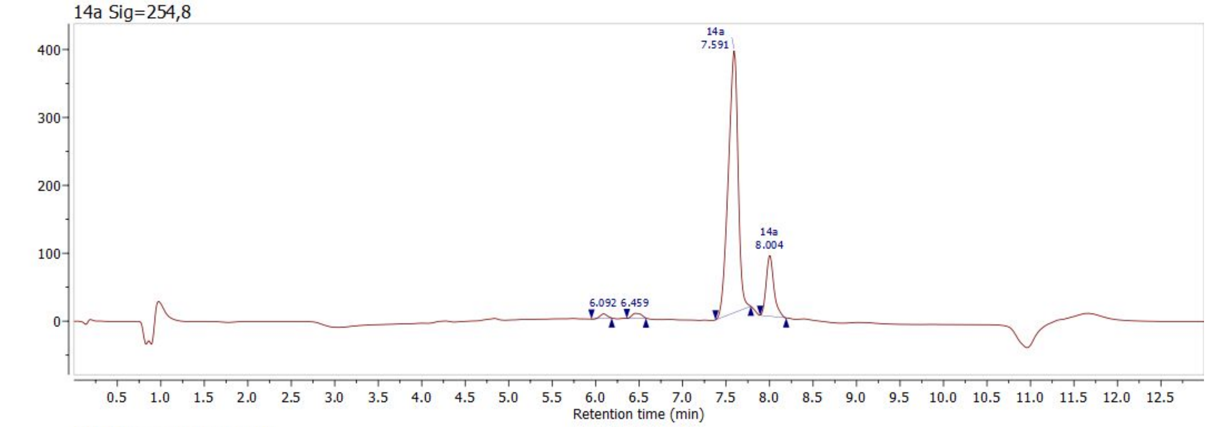

14a MS + spectrum 7.62

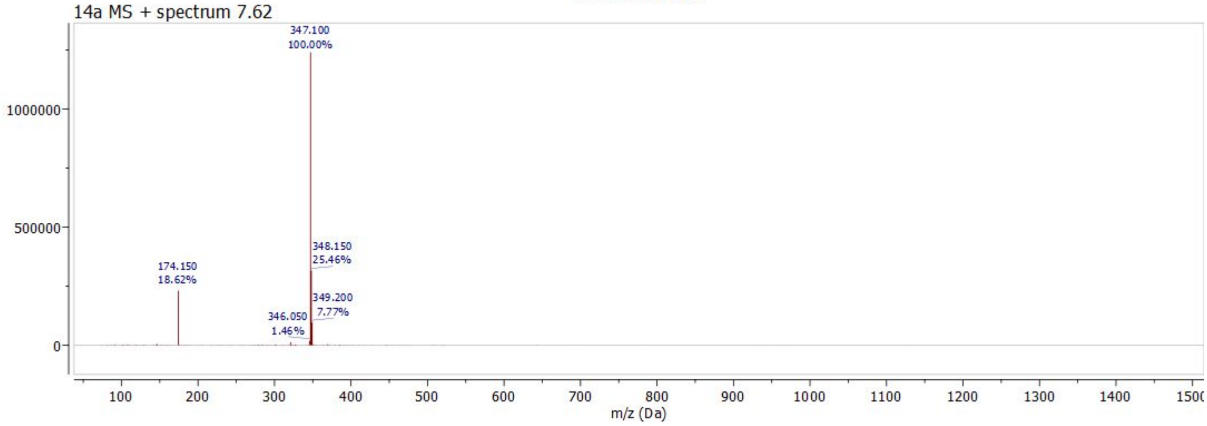

14b Sig=254,8

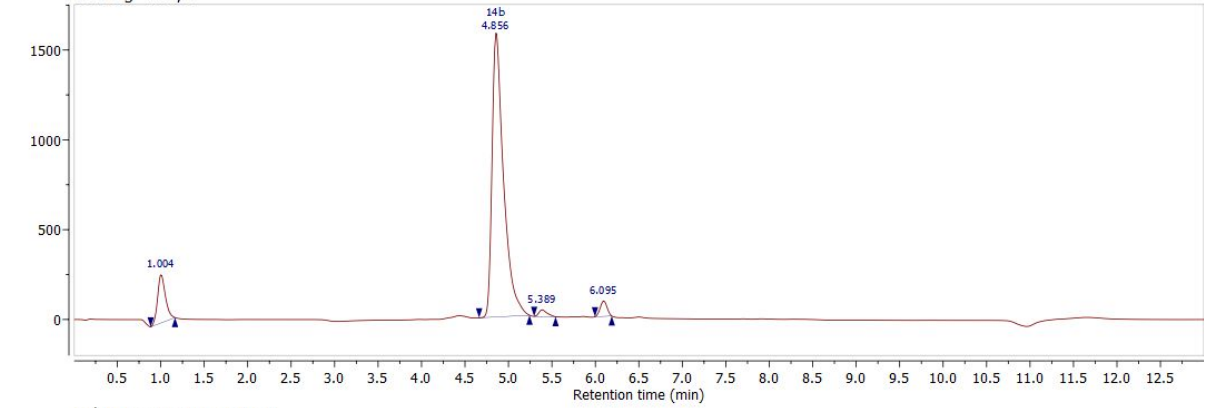

14b MS + spectrum 4.98

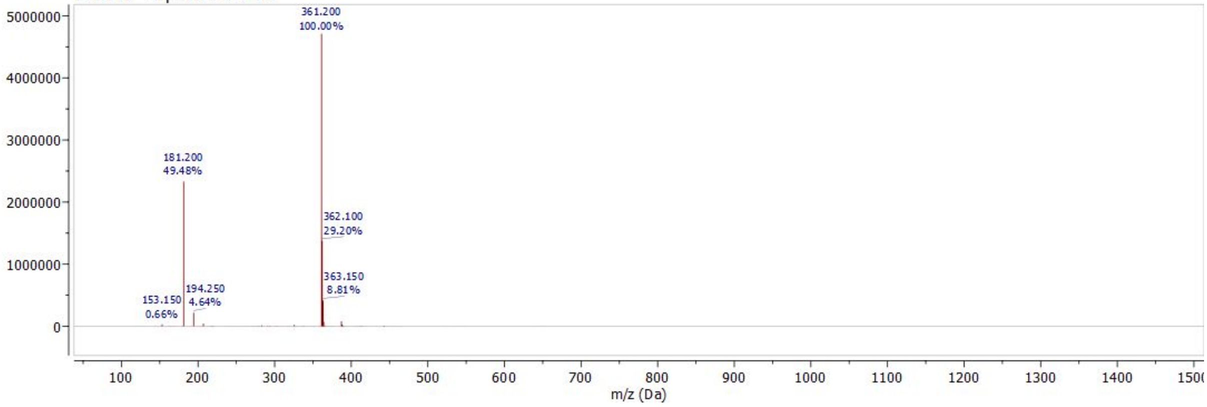

15a TIC

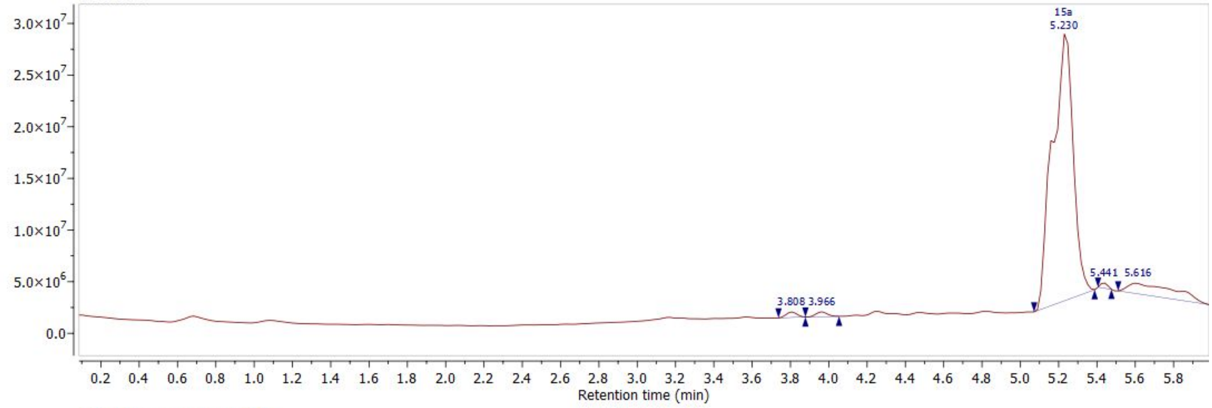

15a MS + spectrum 5.23

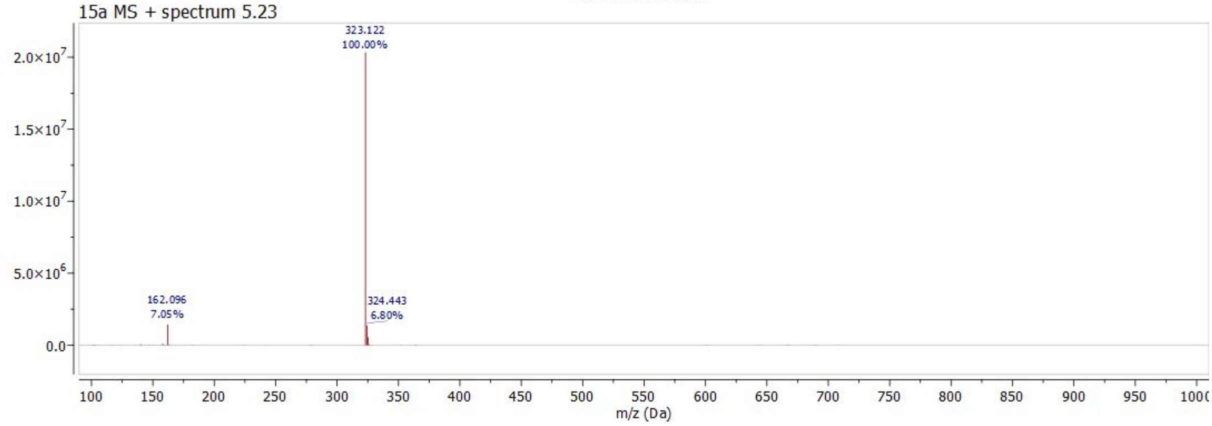

15b TIC

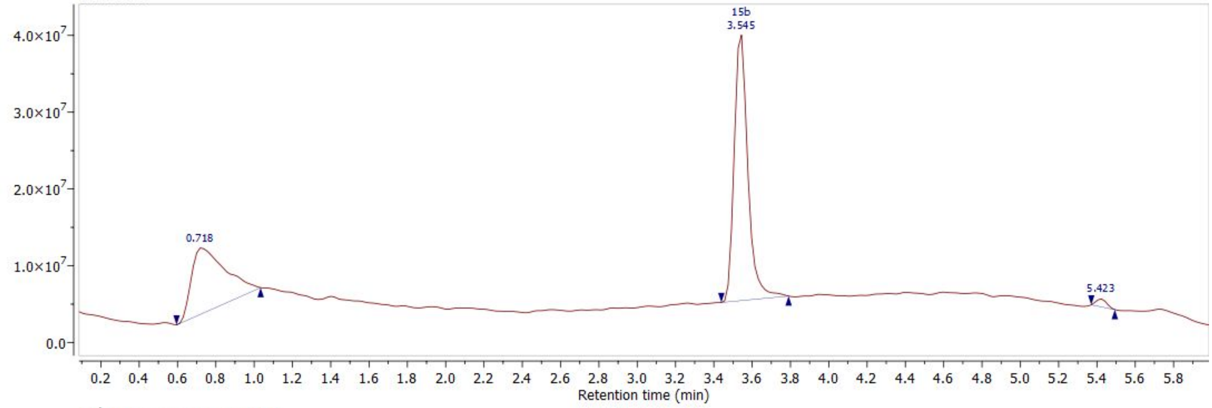

15b MS + spectrum 3.54

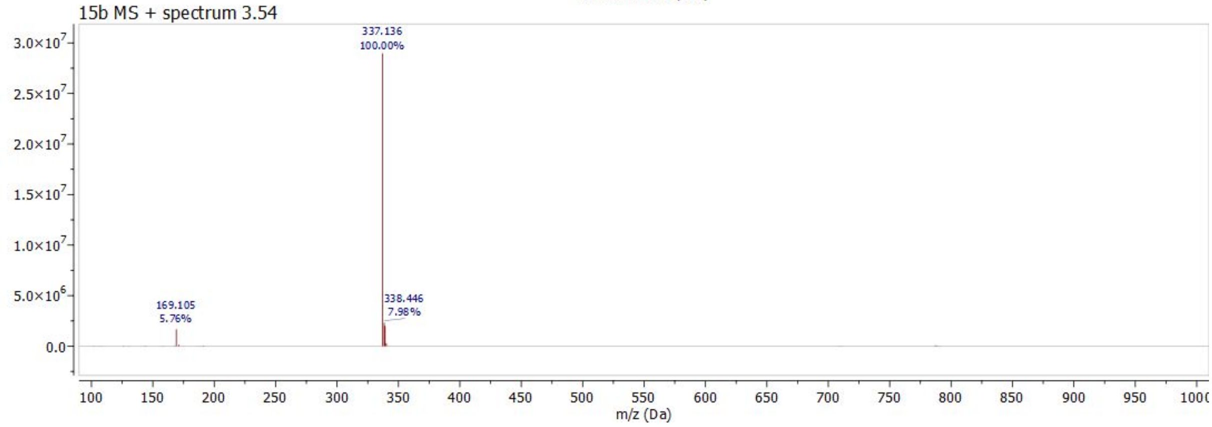

16a TIC

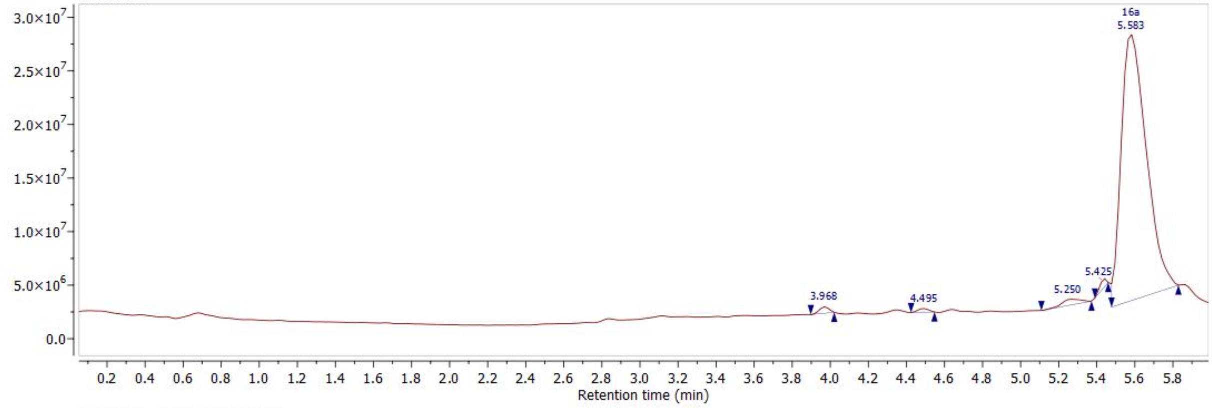

16a MS + spectrum 5.58

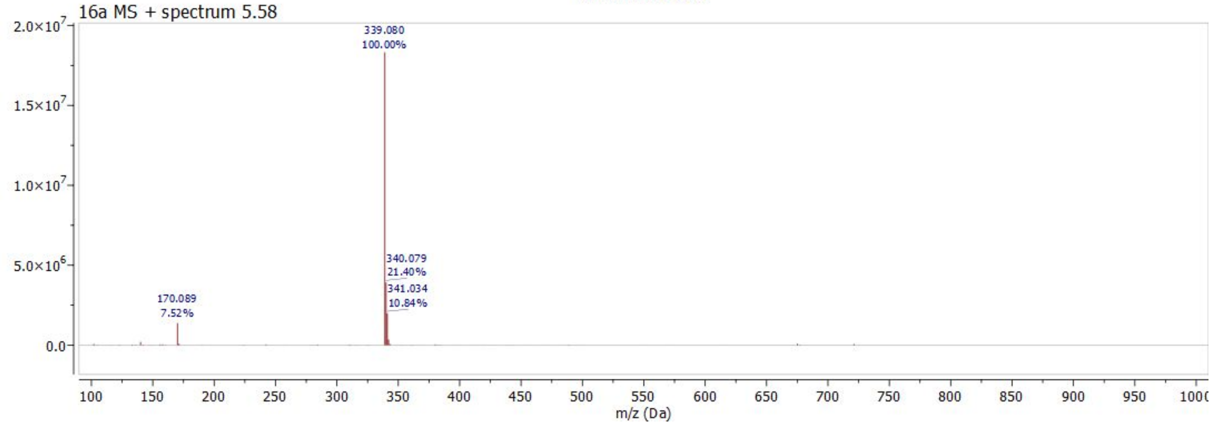

16b Sig=254,8

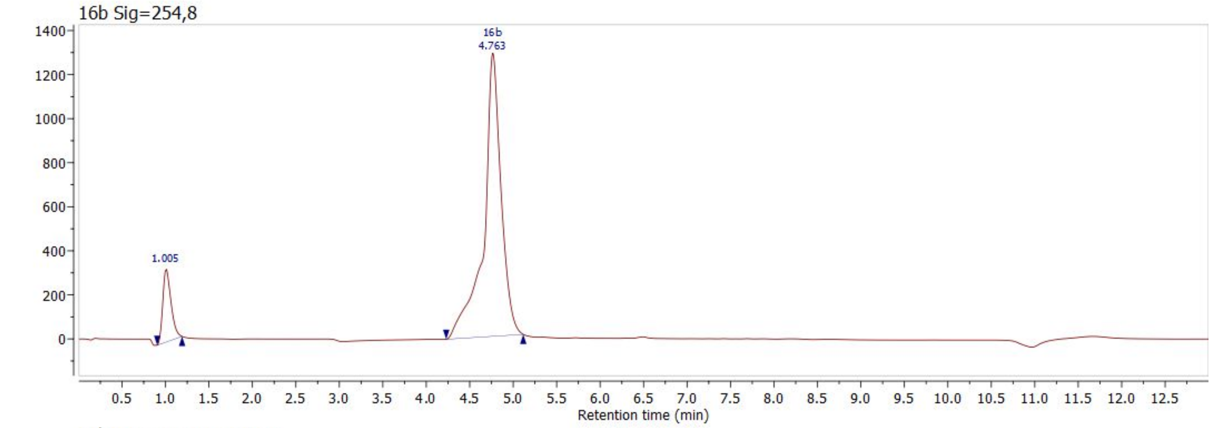

16b MS + spectrum 4.90

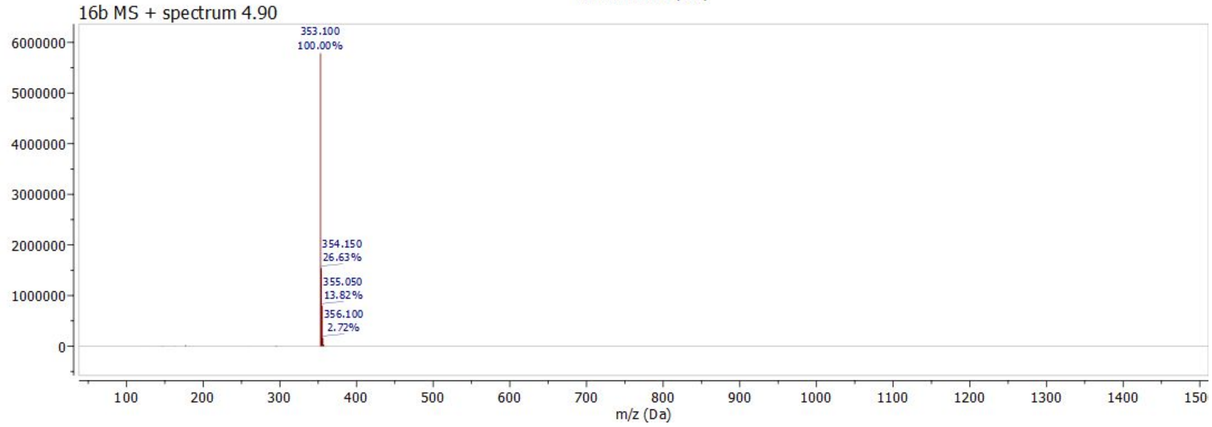

17a Sig=254,8

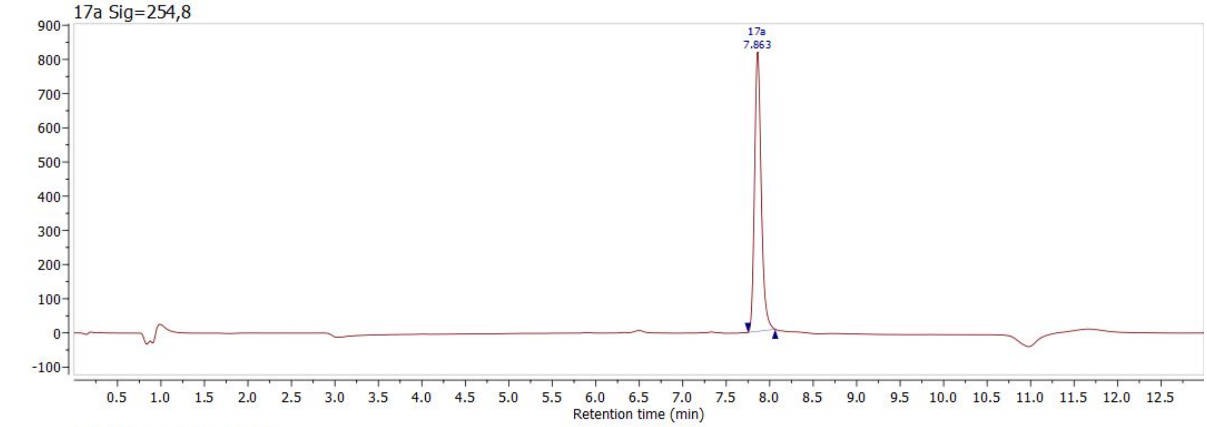

17a MS + spectrum 7.94

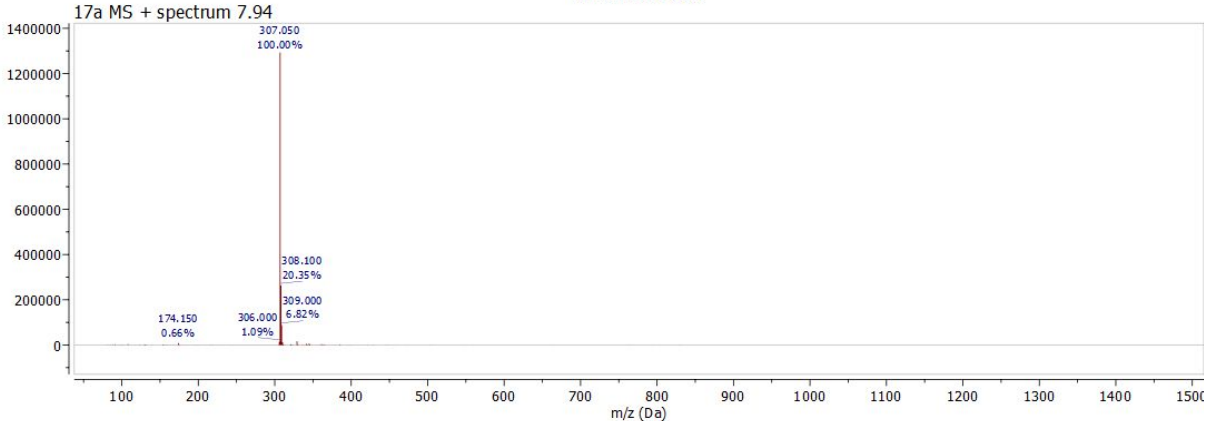

17b TIC

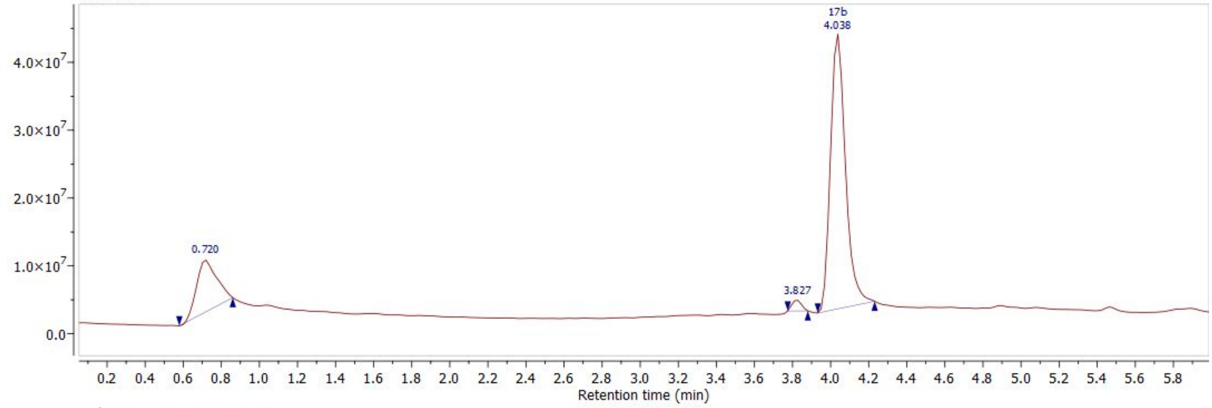

17b MS + spectrum 4.04

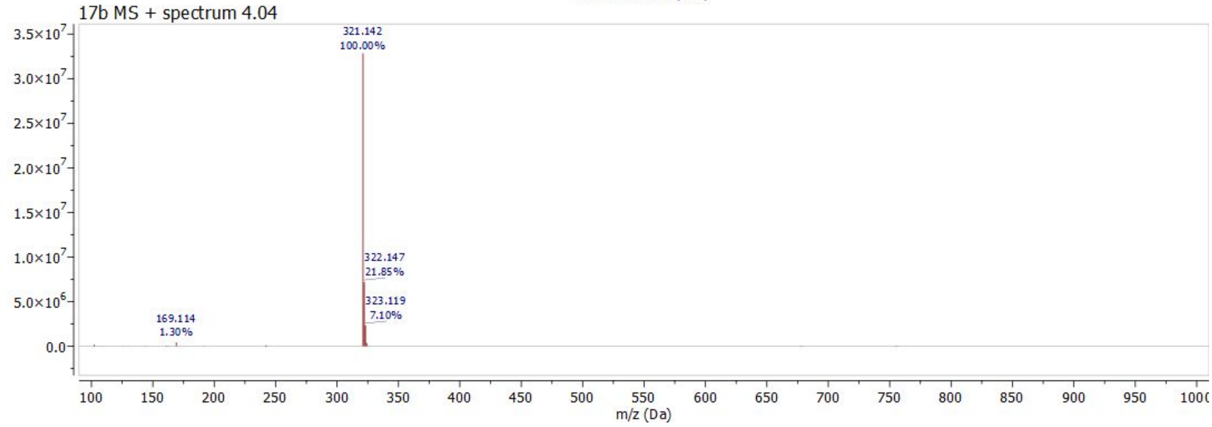

18a Sig=254,8

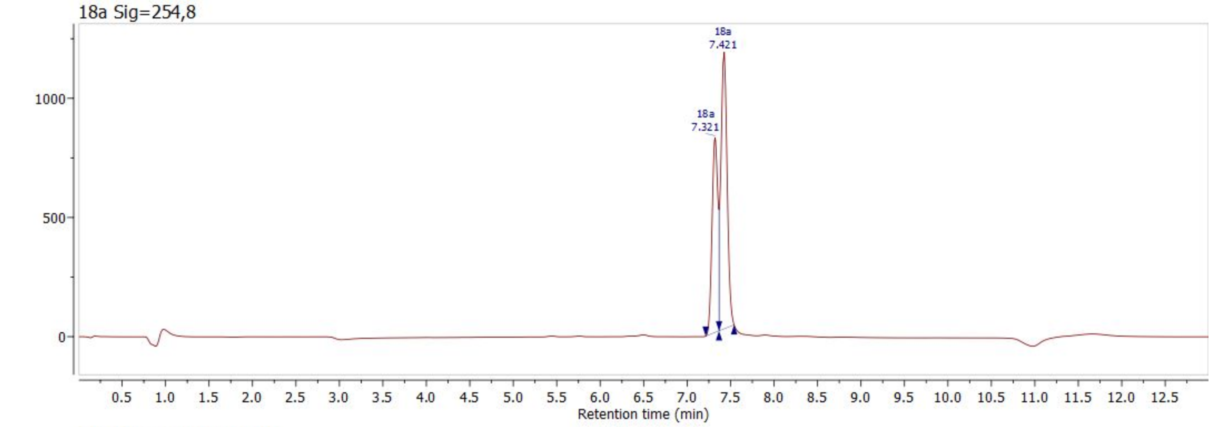

18a MS + spectrum 7.42

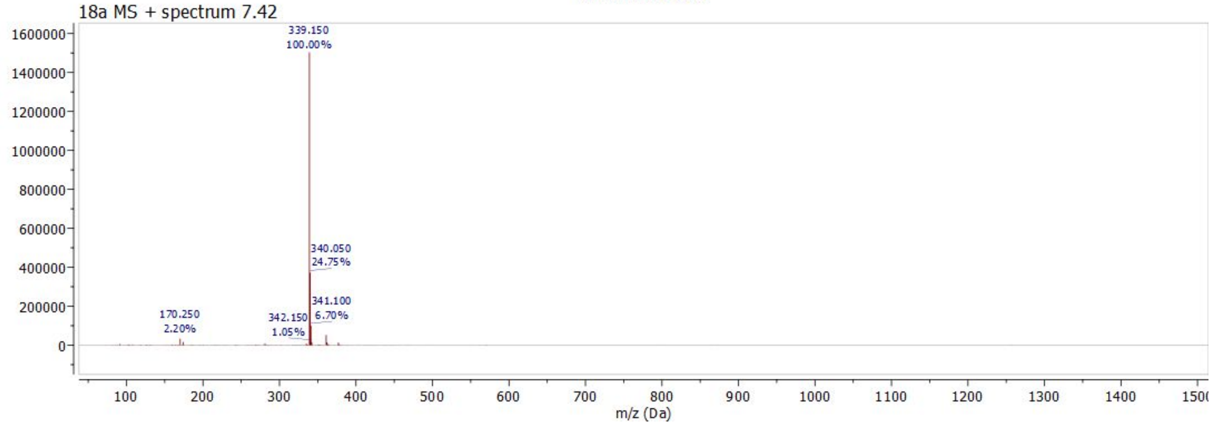

18b TIC

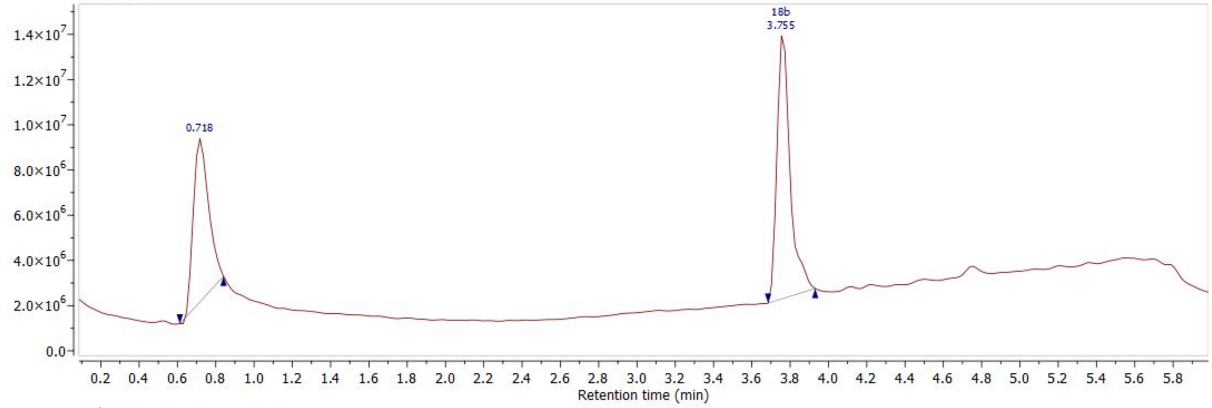

18b MS + spectrum 3.76

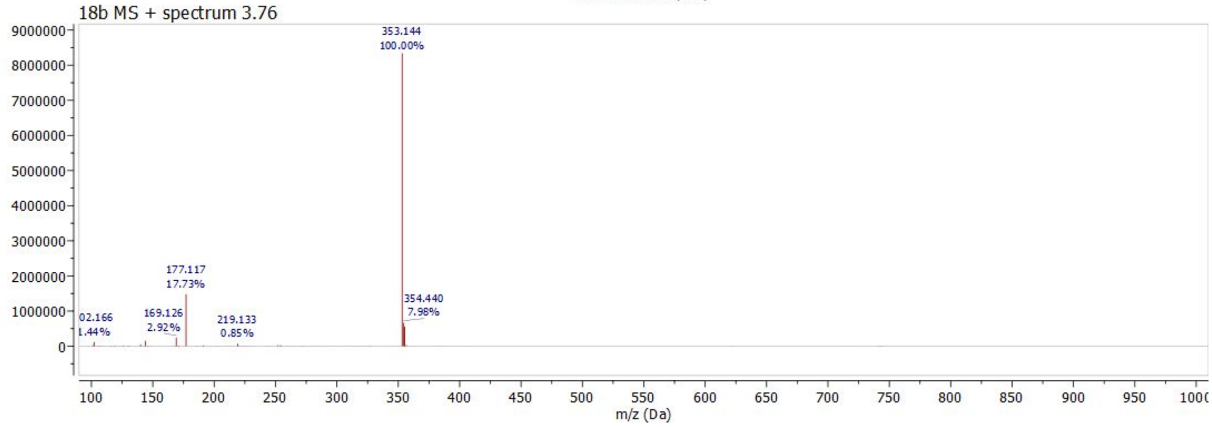

19a TIC

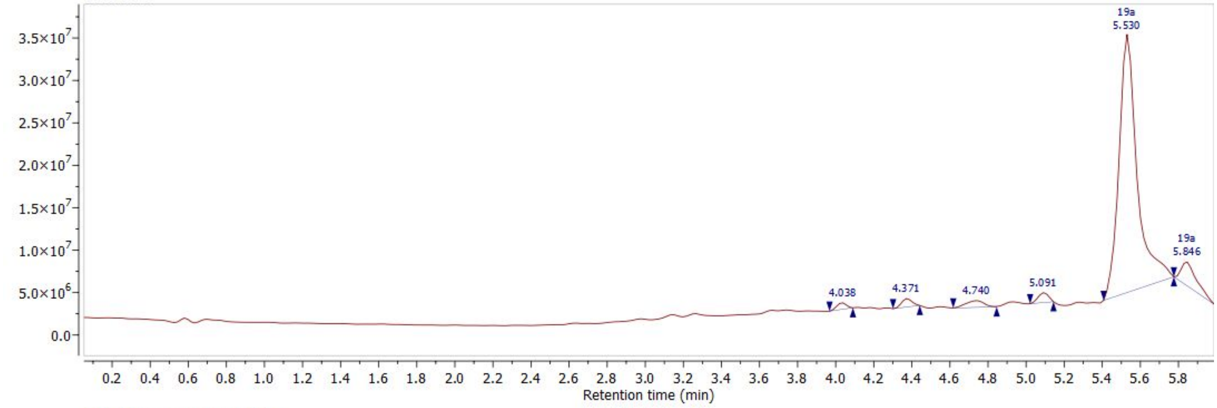

19a MS + spectrum 5.53

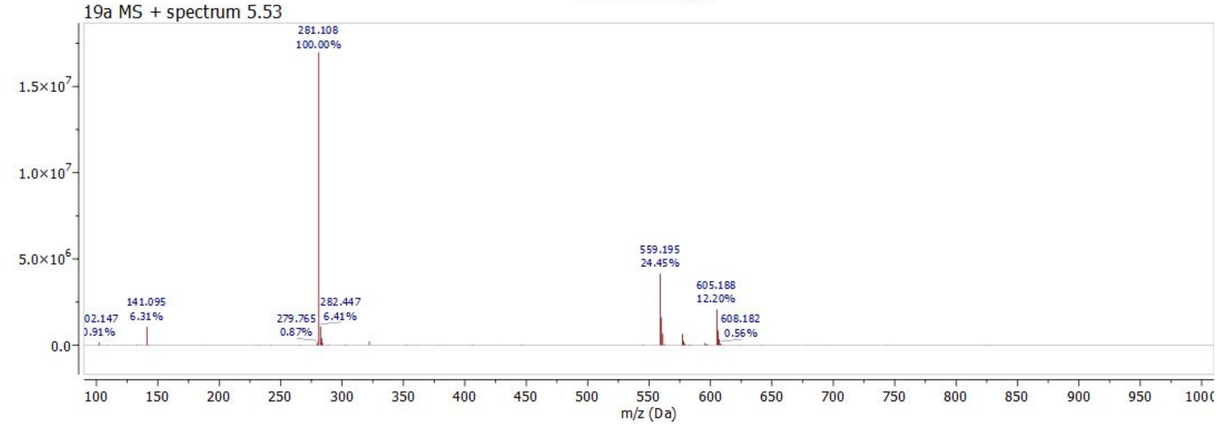

19b TIC

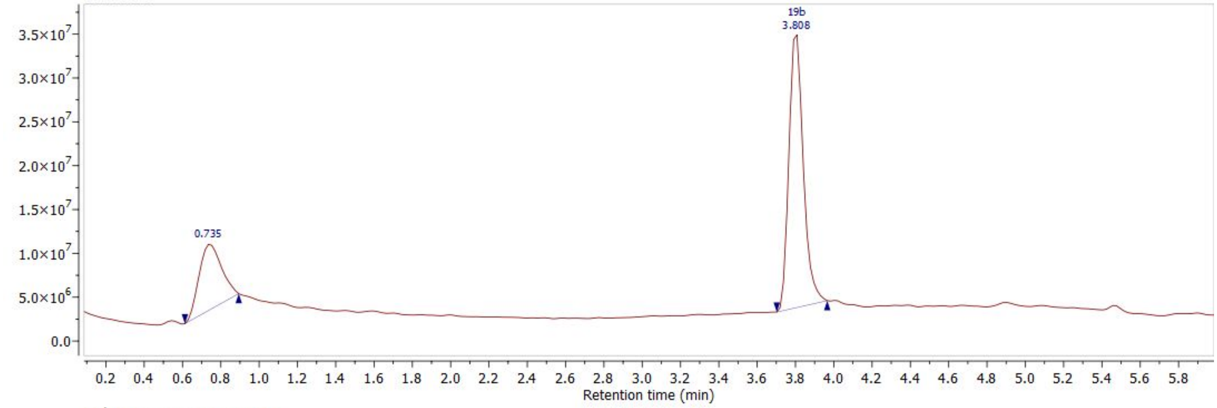

19b MS + spectrum 3.81

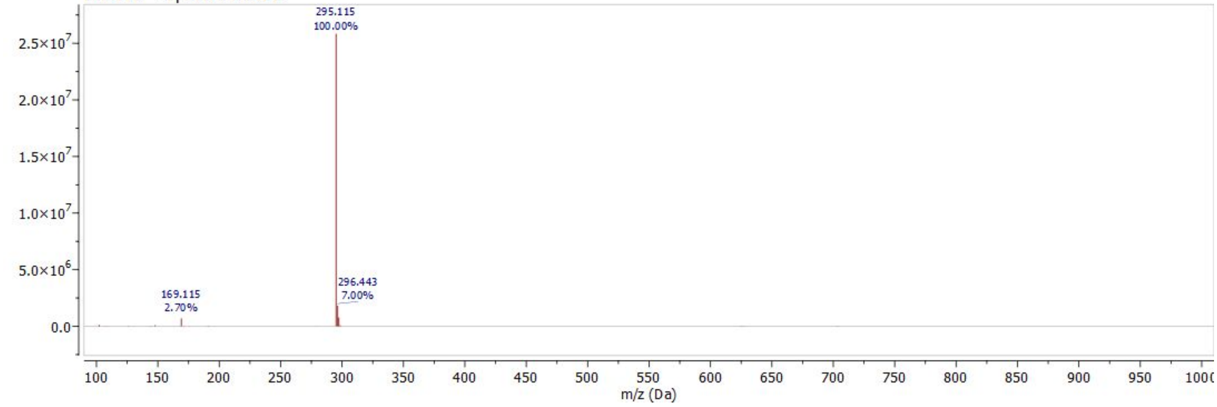

20 TIC

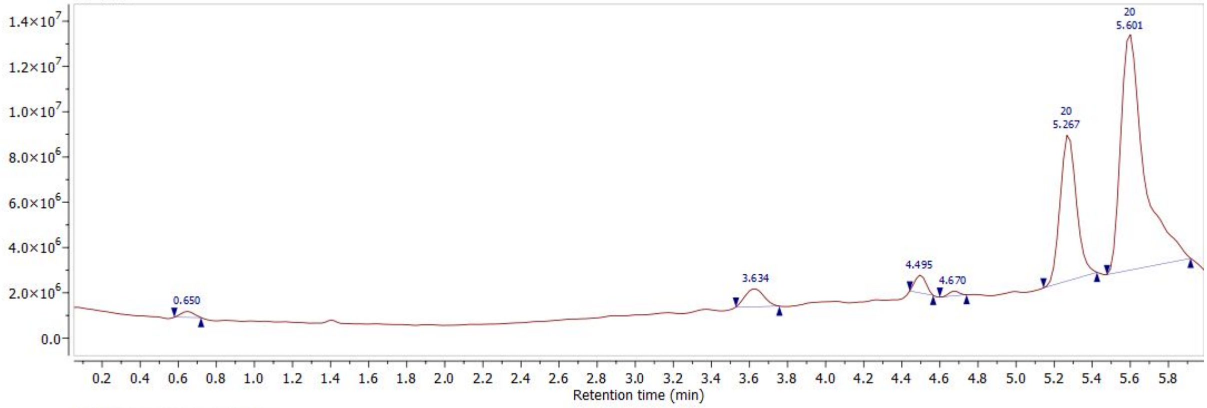

20 MS + spectrum 5.60

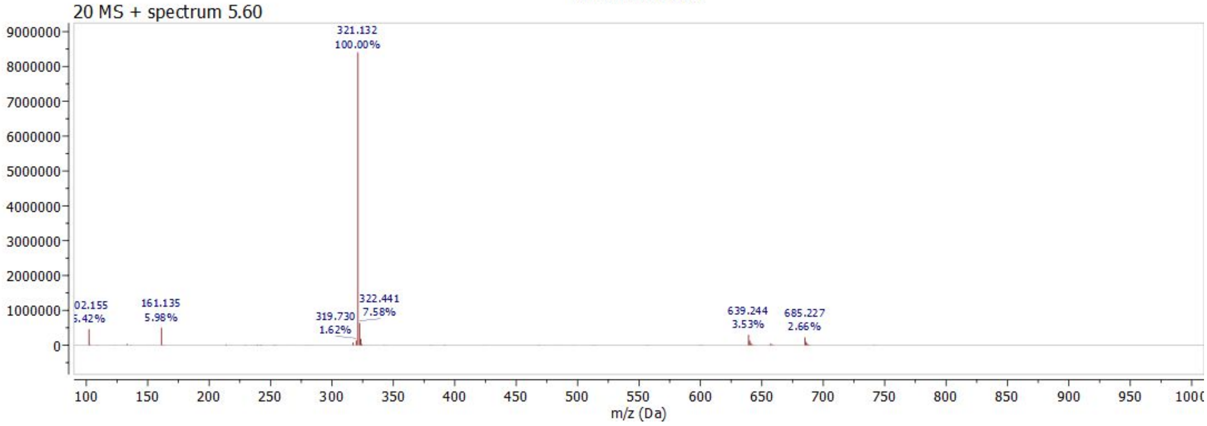

43 Sig=254,8

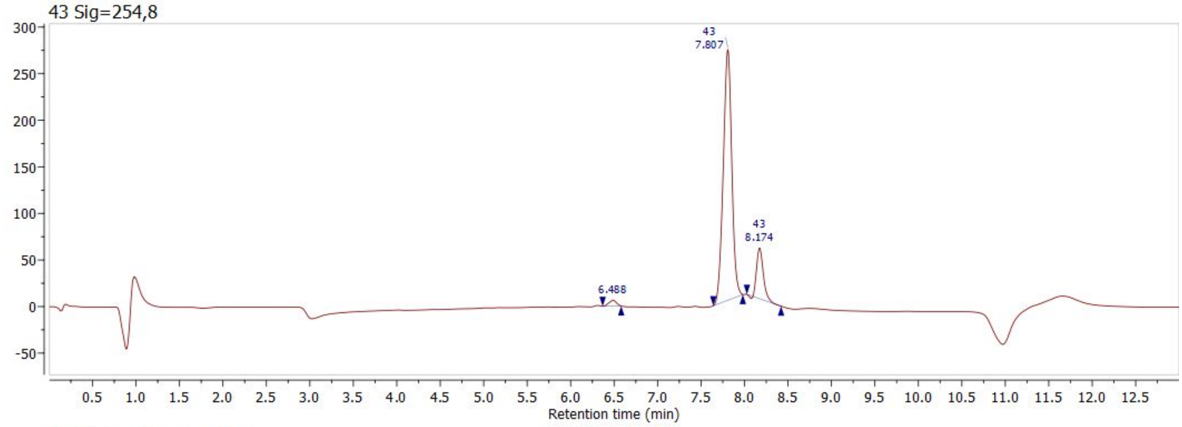

43 MS + spectrum 7.90

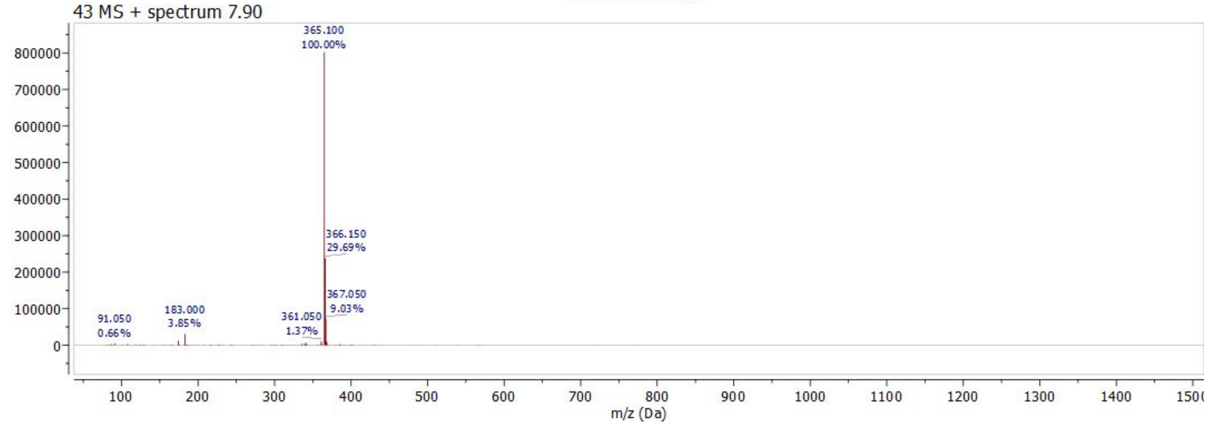

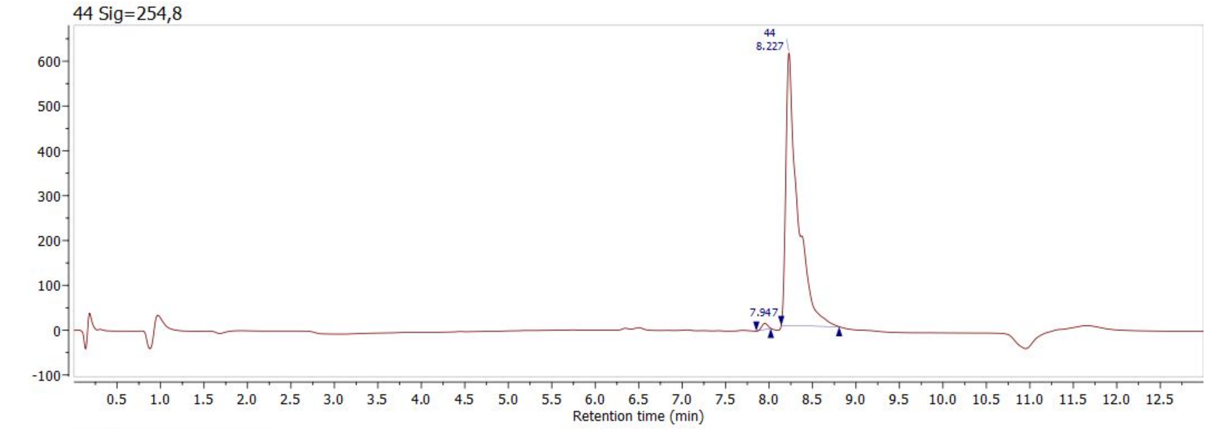
44 Sig=254,8
44
8.227
7.947
Retention time (min)

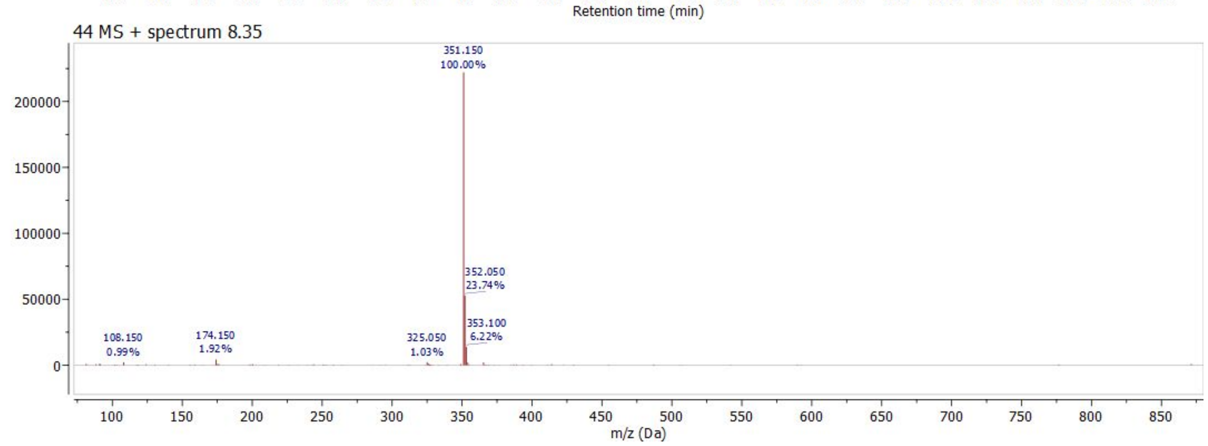
44 MS + spectrum 8.35
351.150
100.00%
352.050
23.74%
353.100
6.22%
325.050
1.03%
108.150
0.99%
174.150
1.92%
m/z (Da)

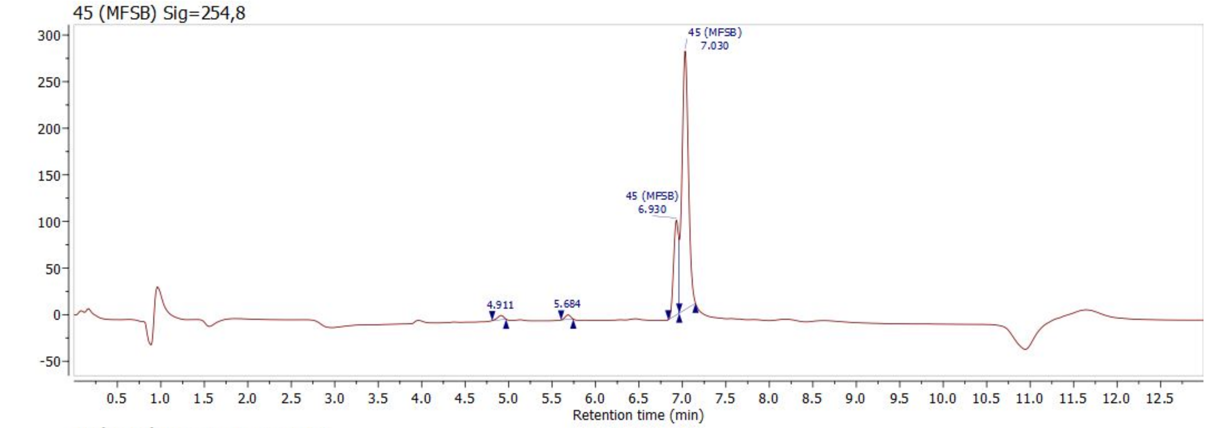
45 (MFSB) Sig=254,8
45 (MFSB)
7.030
45 (MFSB)
6.930
4.911
5.684
Retention time (min)

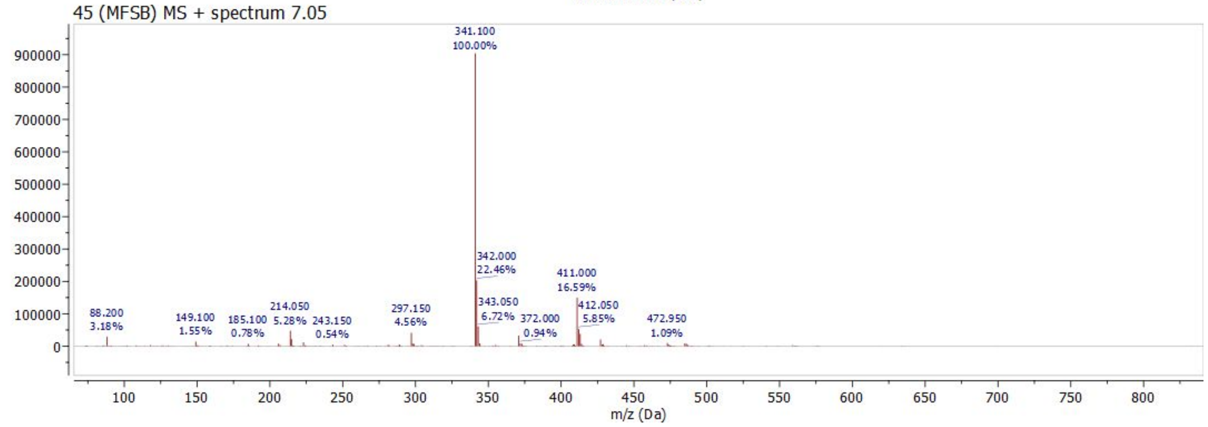
45 (MFSB) MS + spectrum 7.05
341.100
100.00%
342.000
22.46%
343.050
6.72%
411.000
16.59%
412.050
5.85%
372.000
0.94%
472.950
1.09%
88.200
3.18%
149.100
1.55%
185.100
0.78%
214.050
5.28%
243.150
0.54%
297.150
4.56%
m/z (Da)

5. HPLC-MS chromatograms of radiolabeling precursors 48 and 49

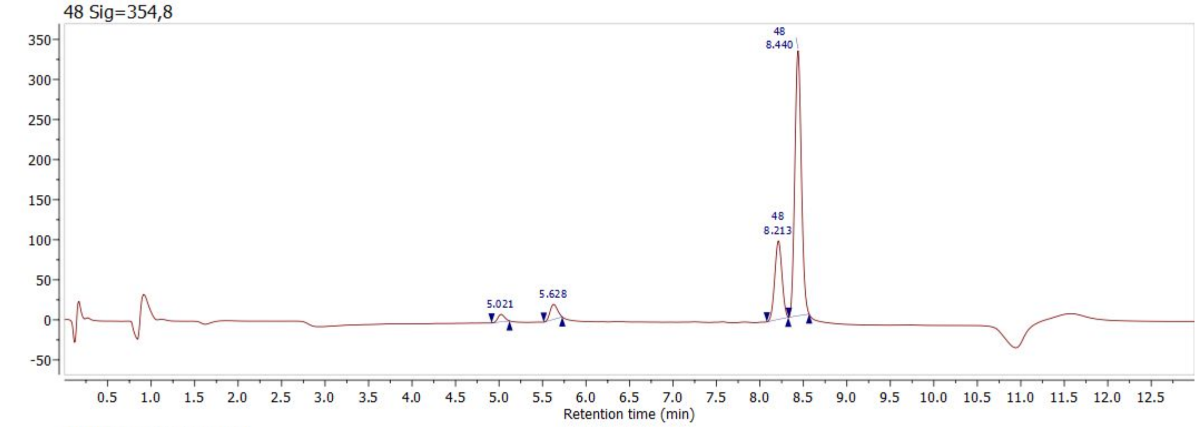

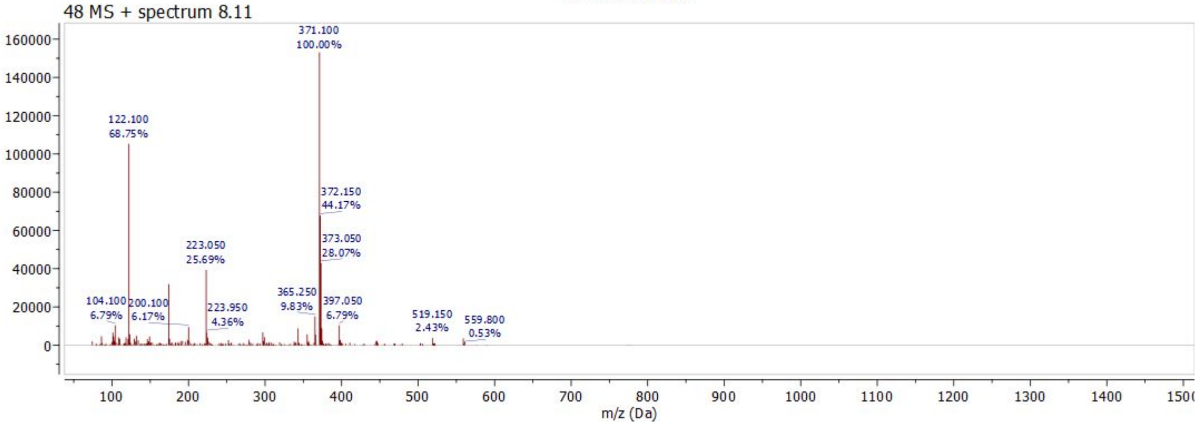

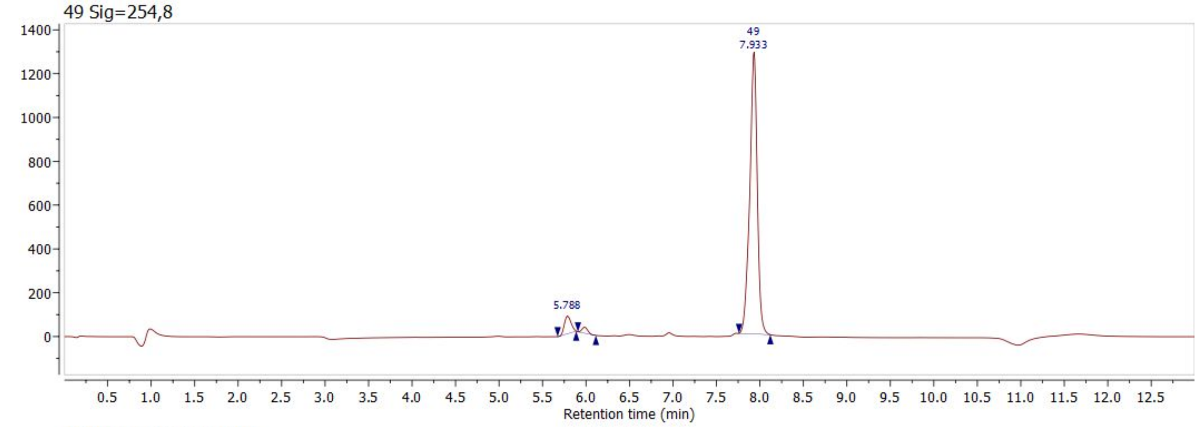

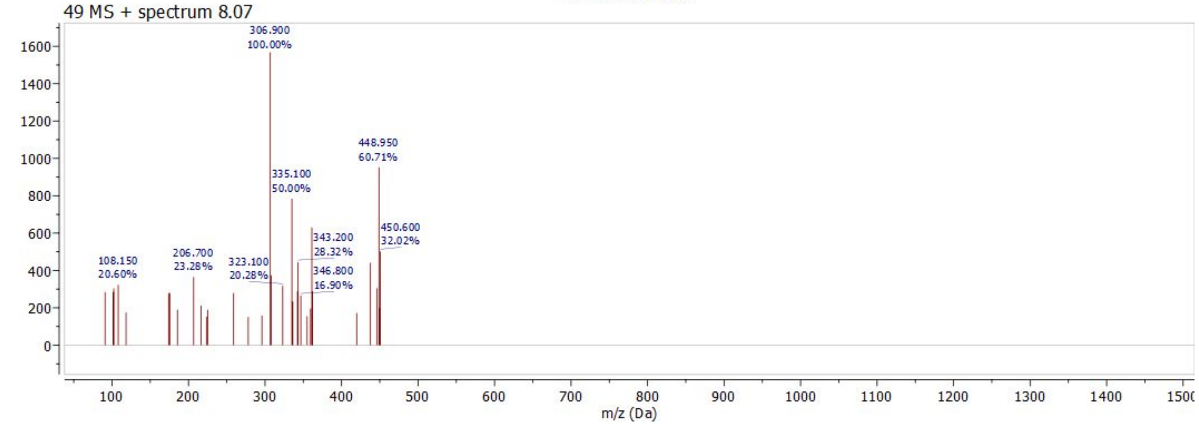

6. ^{1}H NMR spectra of the tested compounds

1a

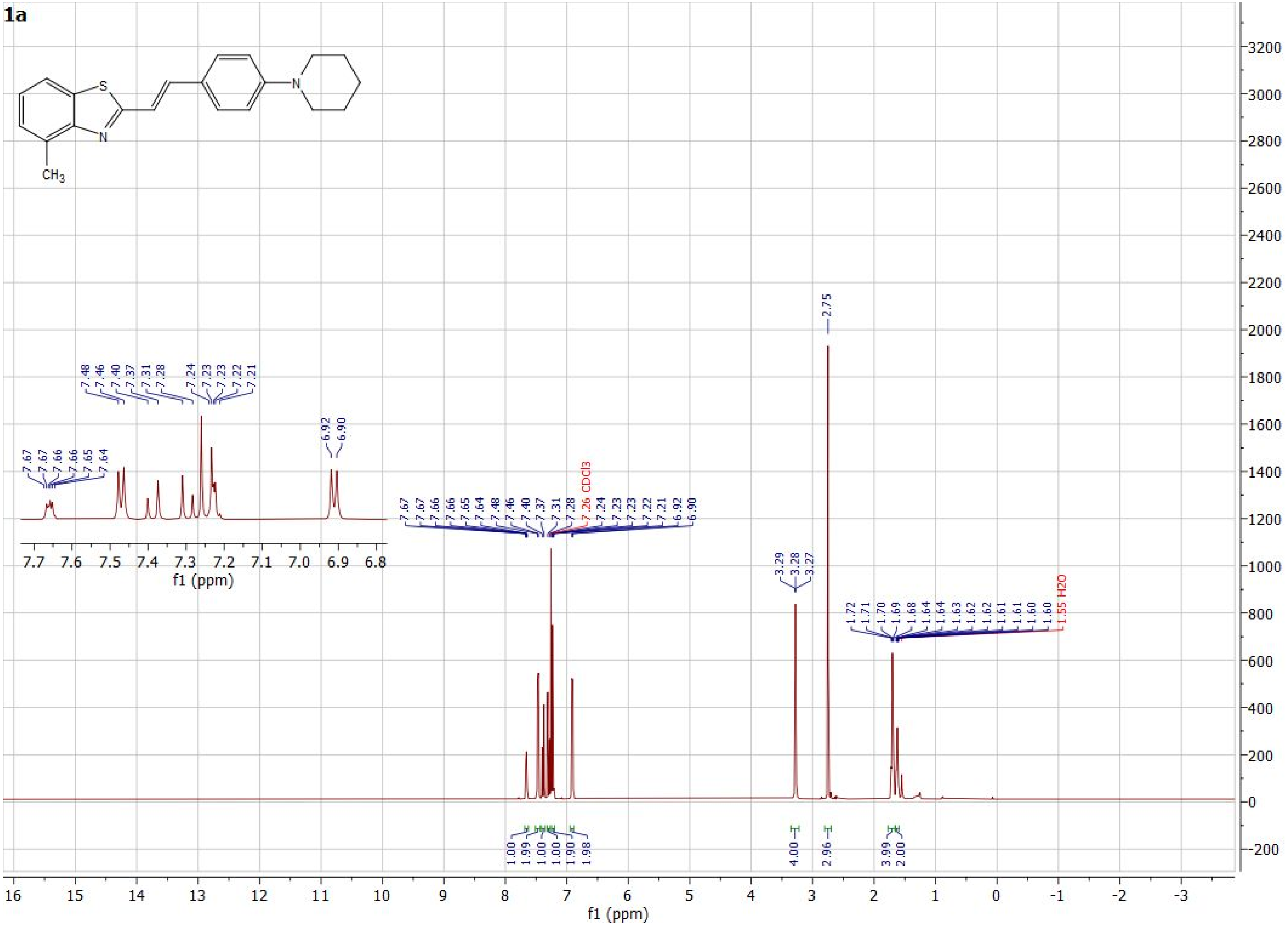

1b

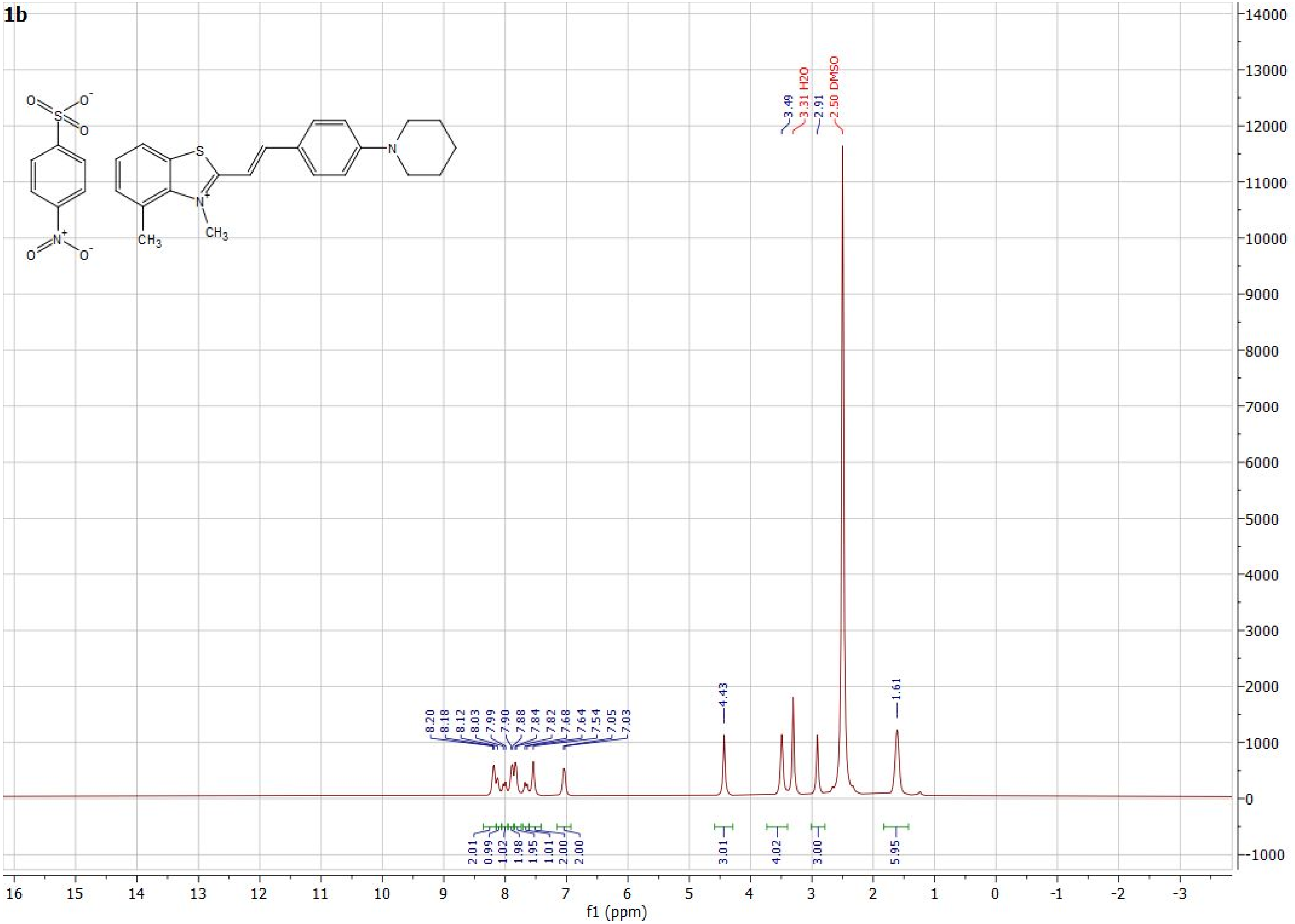

8.20
8.18
8.12
8.03
7.99
7.90
7.88
7.84
7.82
7.68
7.64
7.54
7.05
7.03
4.43
3.49
3.31 H2O
2.91
2.50 DMSO
1.61
2.01
0.99
1.02
1.98
1.95
1.01
2.00
2.00
3.01
4.02
3.00
5.95
f1 (ppm)

2a

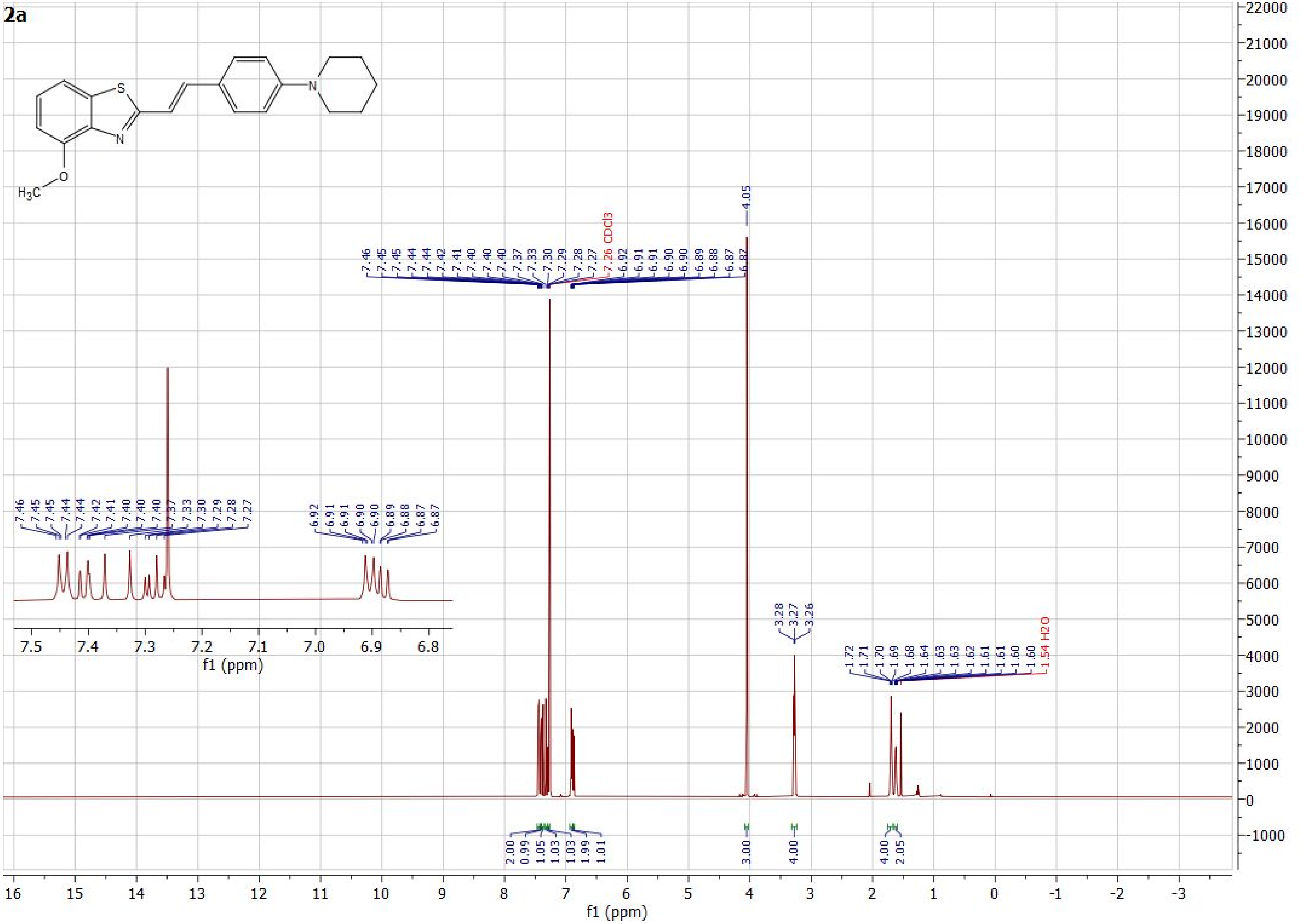

2b

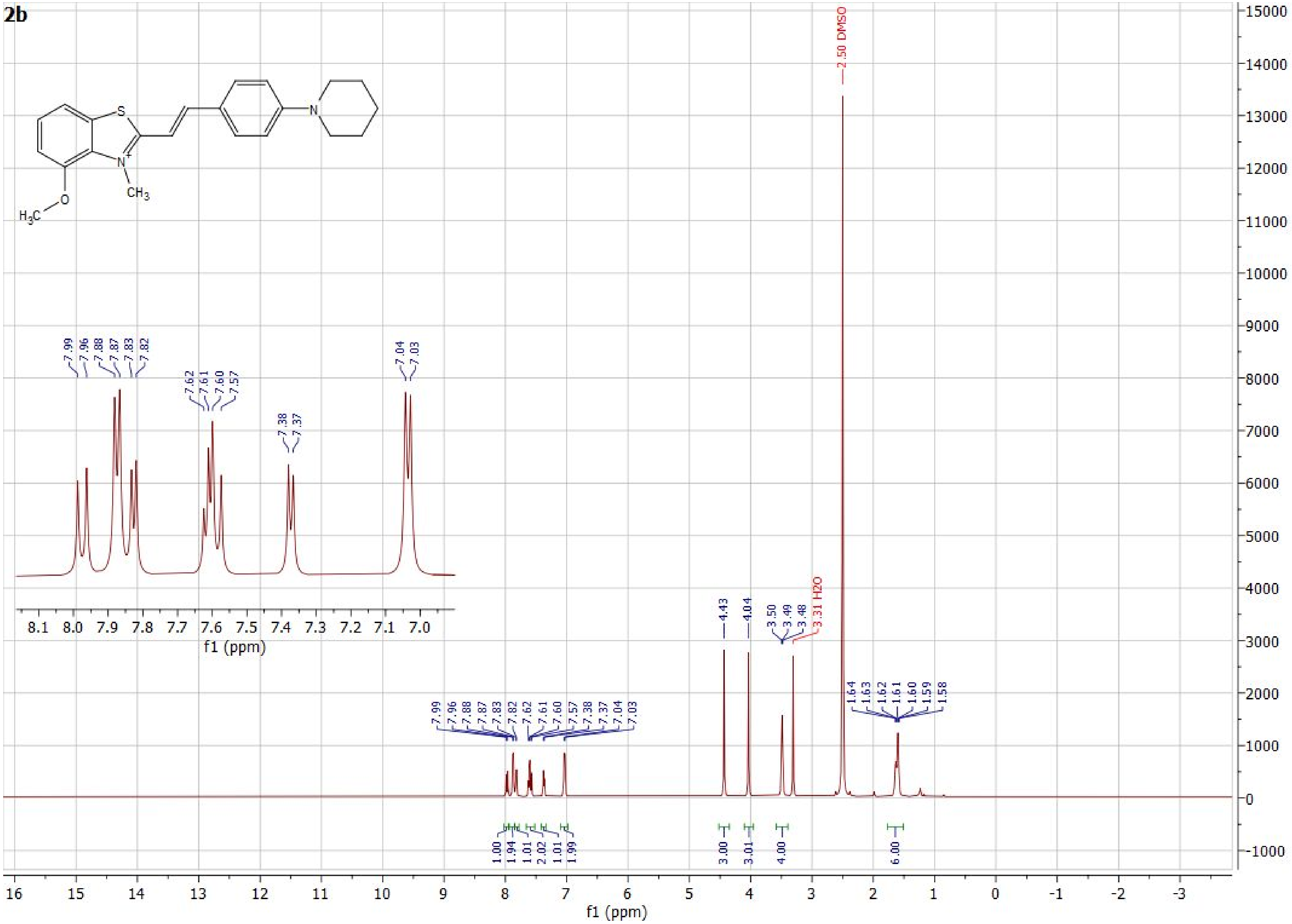
S
N+
CH3
O
H3C
N
7.99 7.96 7.88 7.87 7.83 7.82
7.62 7.61 7.60 7.57
7.38 7.37
7.04 7.03
8.1 8.0 7.9 7.8 7.7 7.6 7.5 7.4 7.3 7.2 7.1 7.0
f1 (ppm)
2.50 DMSO
4.43
4.04
3.50 3.49 3.48
3.31 H2O
1.64 1.63 1.62 1.61 1.60 1.59 1.58
1.00 1.94 1.01 2.02 1.01 1.99
3.00 3.01 4.00 6.00
16 15 14 13 12 11 10 9 8 7 6 5 4 3 2 1 0 -1 -2 -3
f1 (ppm)
15000 14000 13000 12000 11000 10000 9000 8000 7000 6000 5000 4000 3000 2000 1000 0 -1000

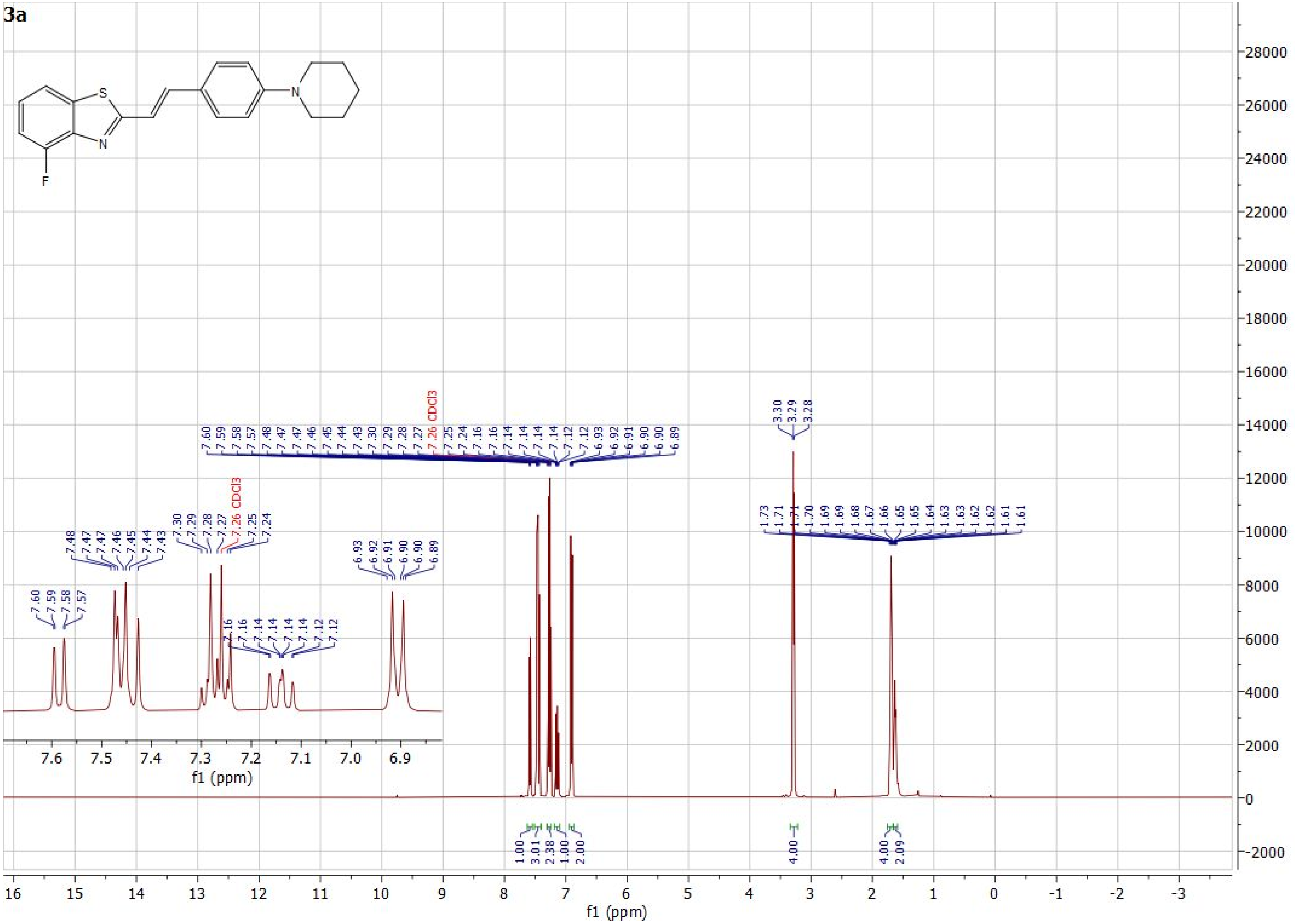
3a
S
N
N
F
f1 (ppm)
f1 (ppm)
CDCl3

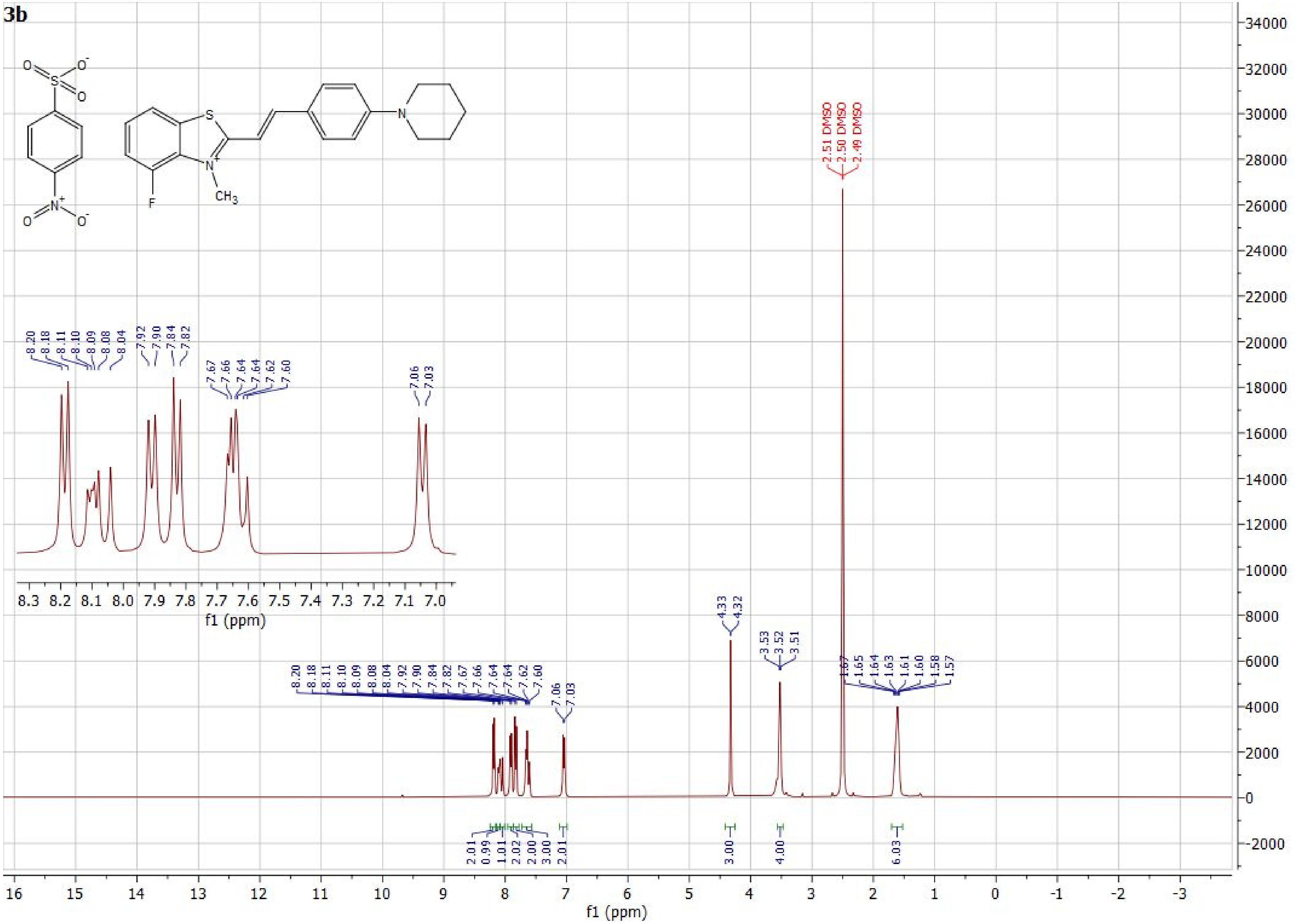
3b
f1 (ppm)
2.51 DMSO
2.50 DMSO
2.49 DMSO

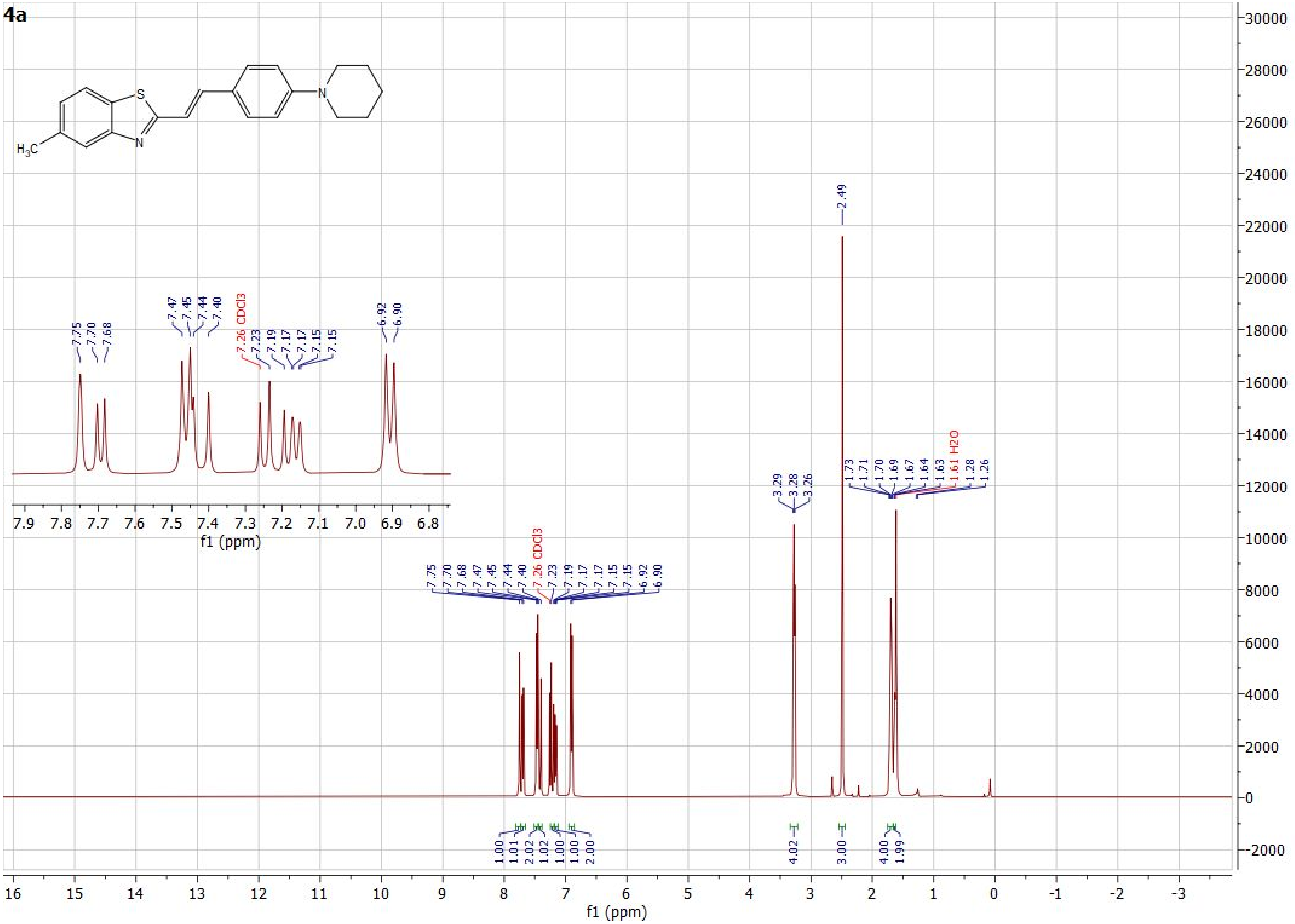
4a
H3C
S
N
N
7.75 7.70 7.68 7.47 7.45 7.44 7.40 7.26 CDCl3 7.23 7.19 7.17 7.17 7.15 7.15 6.92 6.90
3.29 3.28 3.26
2.49
1.73 1.71 1.70 1.69 1.67 1.64 1.63 1.61 H2O 1.28 1.26
1.00 1.01 2.02 1.02 1.00 1.00 2.00
4.02 3.00 4.00 1.99
f1 (ppm)

4b

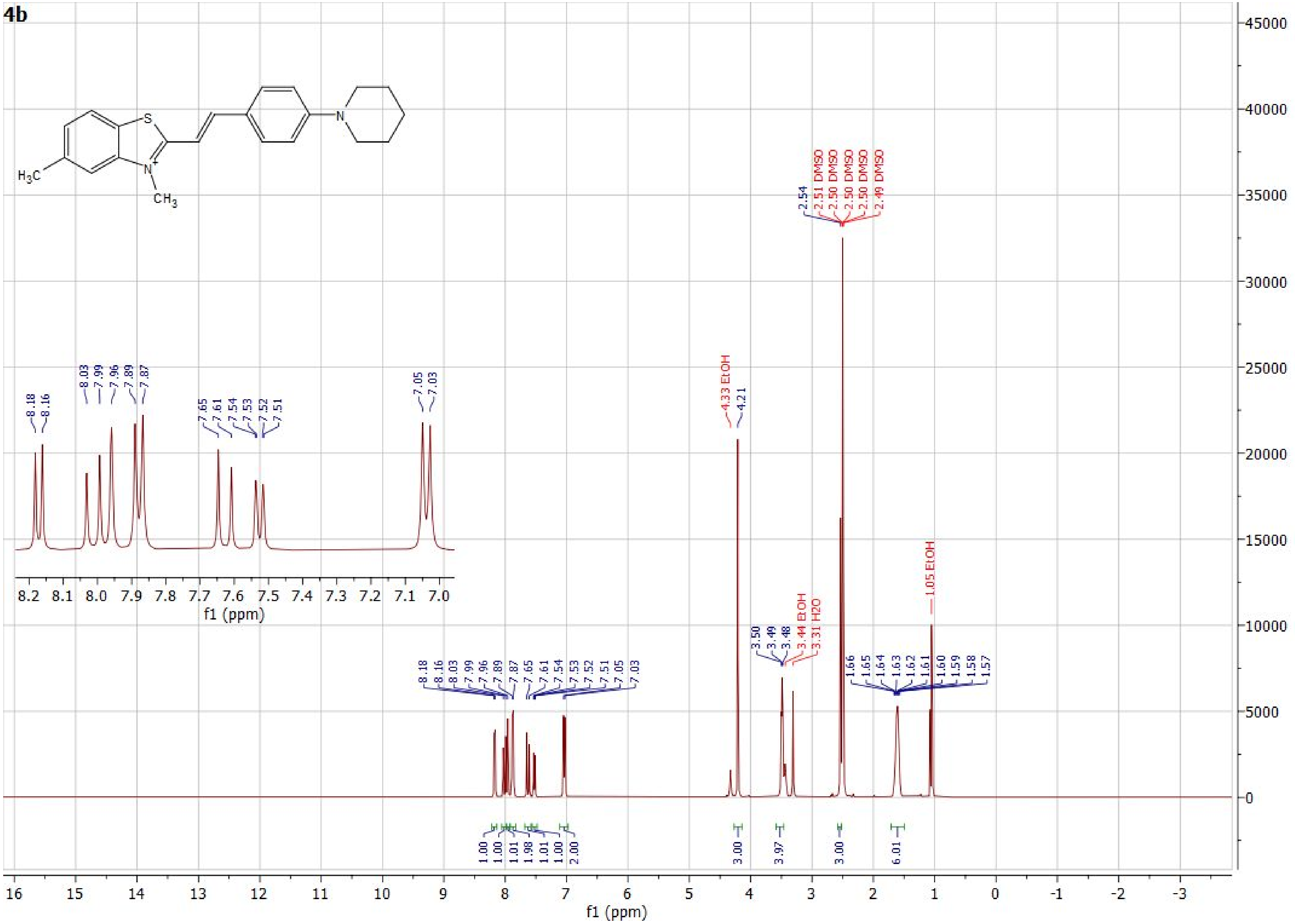

S
N
H3C
CH3
8.18
8.16
8.03
7.99
7.96
7.89
7.87
7.65
7.61
7.54
7.53
7.52
7.51
7.05
7.03
4.33 EtOH
4.21
3.50
3.49
3.48
3.44 EtOH
3.31 H2O
2.54
2.51 DMSO
2.50 DMSO
2.50 DMSO
2.50 DMSO
2.49 DMSO
1.66
1.65
1.64
1.63
1.62
1.61
1.60
1.59
1.58
1.57
1.05 EtOH
1.00
1.00
1.01
1.98
1.01
1.00
2.00
3.00
3.97
3.00
6.01
f1 (ppm)

5a

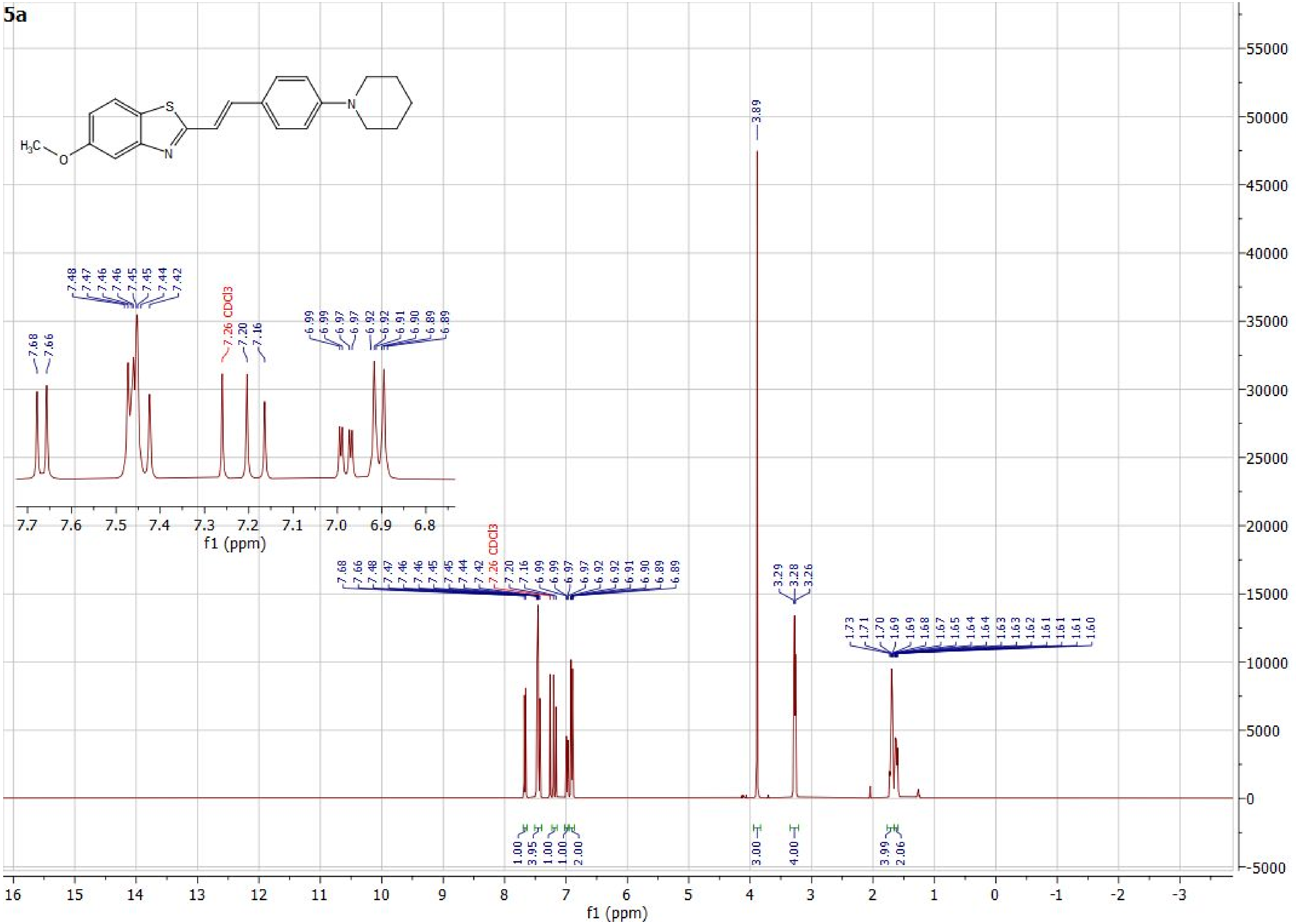

5b

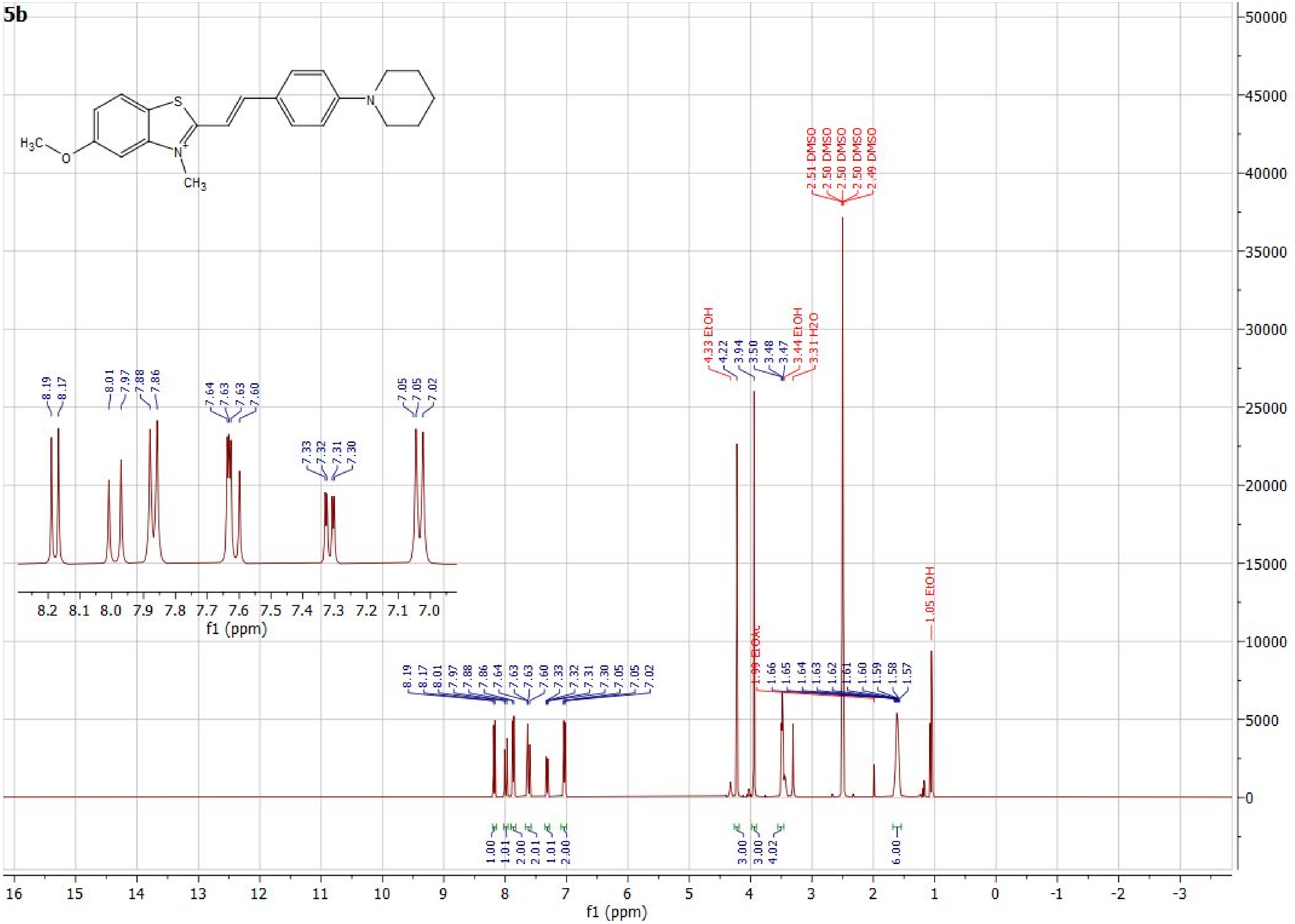

f1 (ppm)
2.51 DMSO
2.50 DMSO
2.50 DMSO
2.50 DMSO
2.49 DMSO
4.33 EtOH
3.44 EtOH
3.31 H2O
1.05 EtOH
1.99 EtOAc

6a

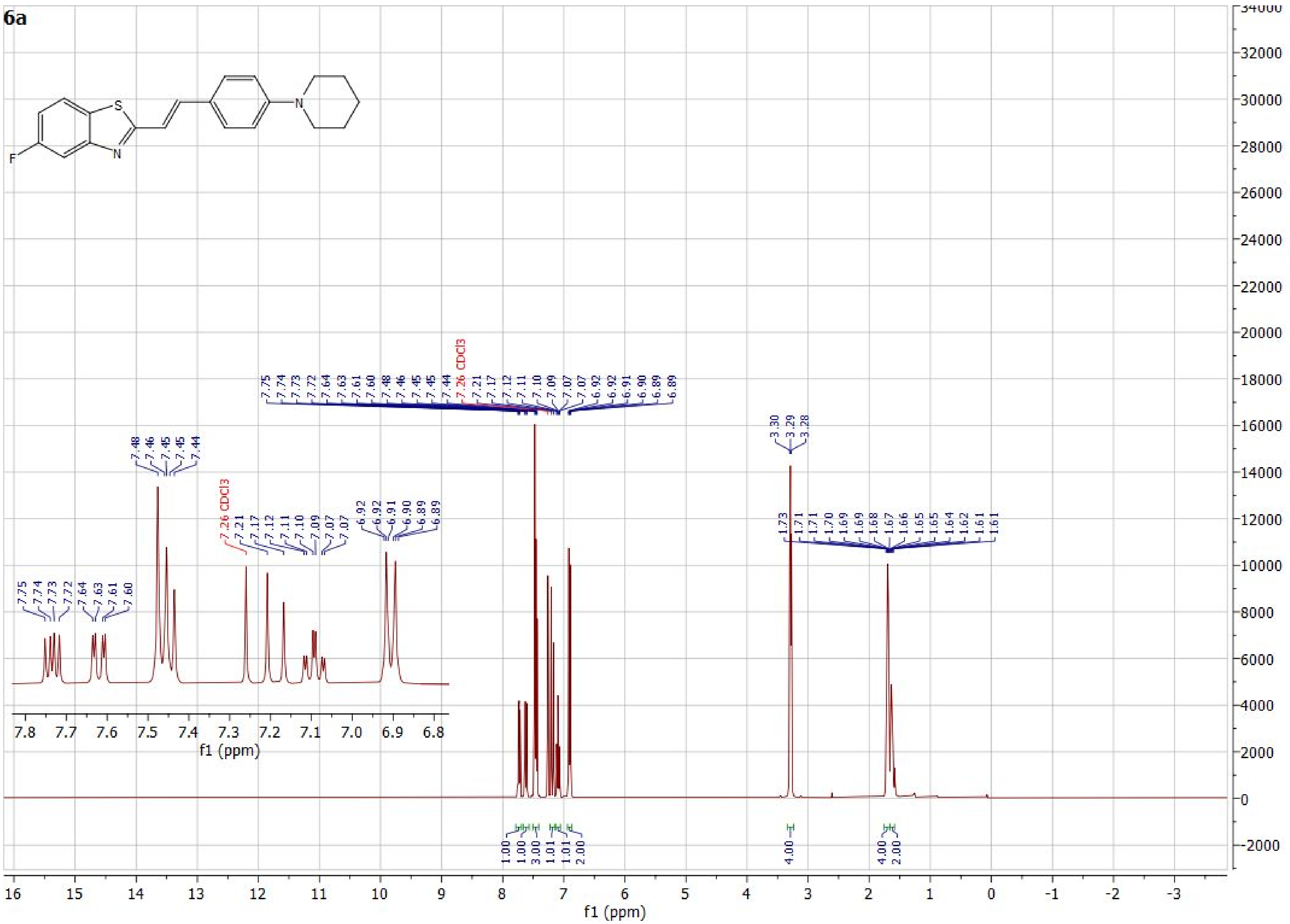

6b

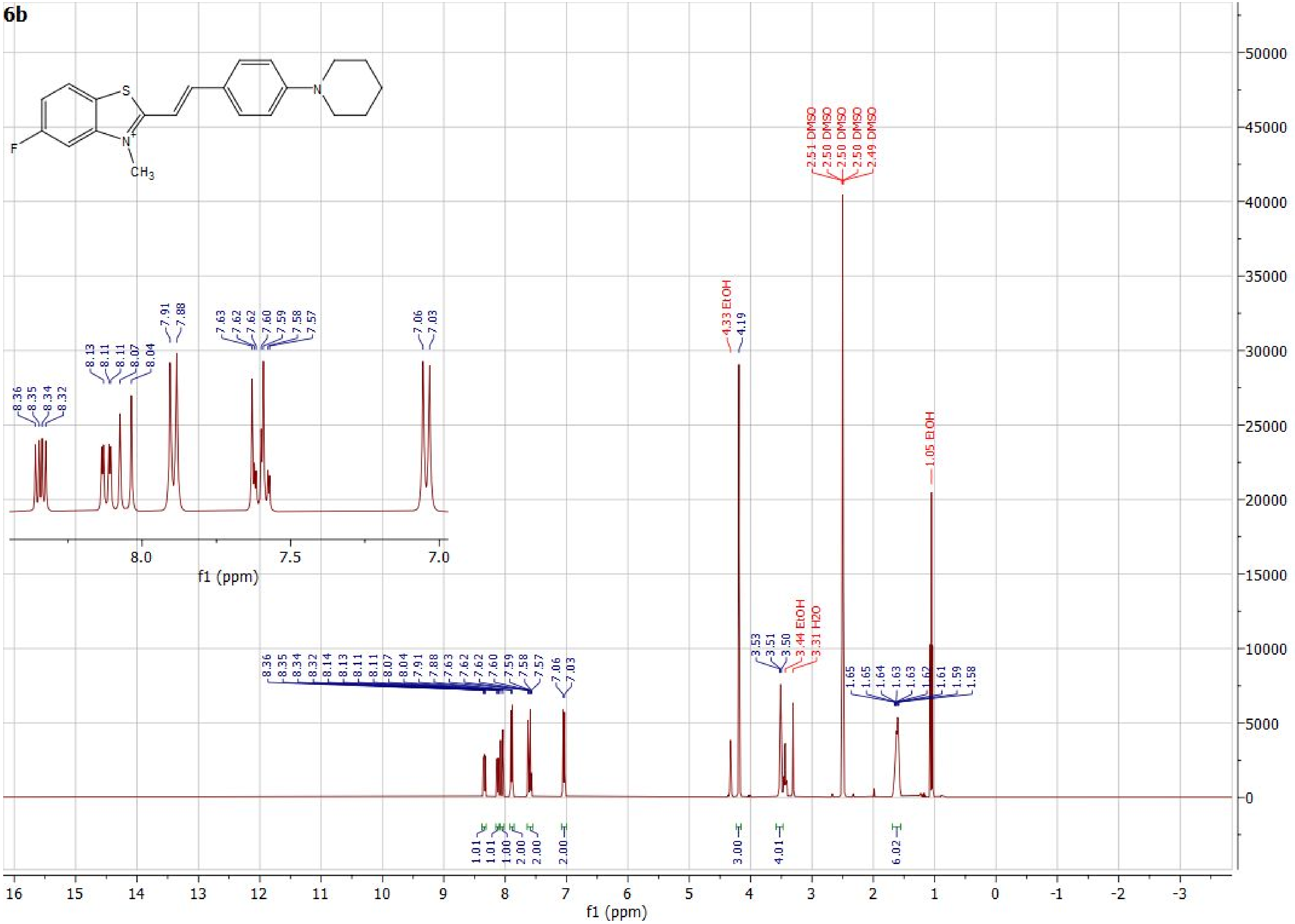

S
N
F
CH3
f1 (ppm)
4.33 EtOH
4.19
2.51 DMSO
2.50 DMSO
2.50 DMSO
2.50 DMSO
2.49 DMSO
3.44 EtOH
3.31 H2O
1.05 EtOH

7a

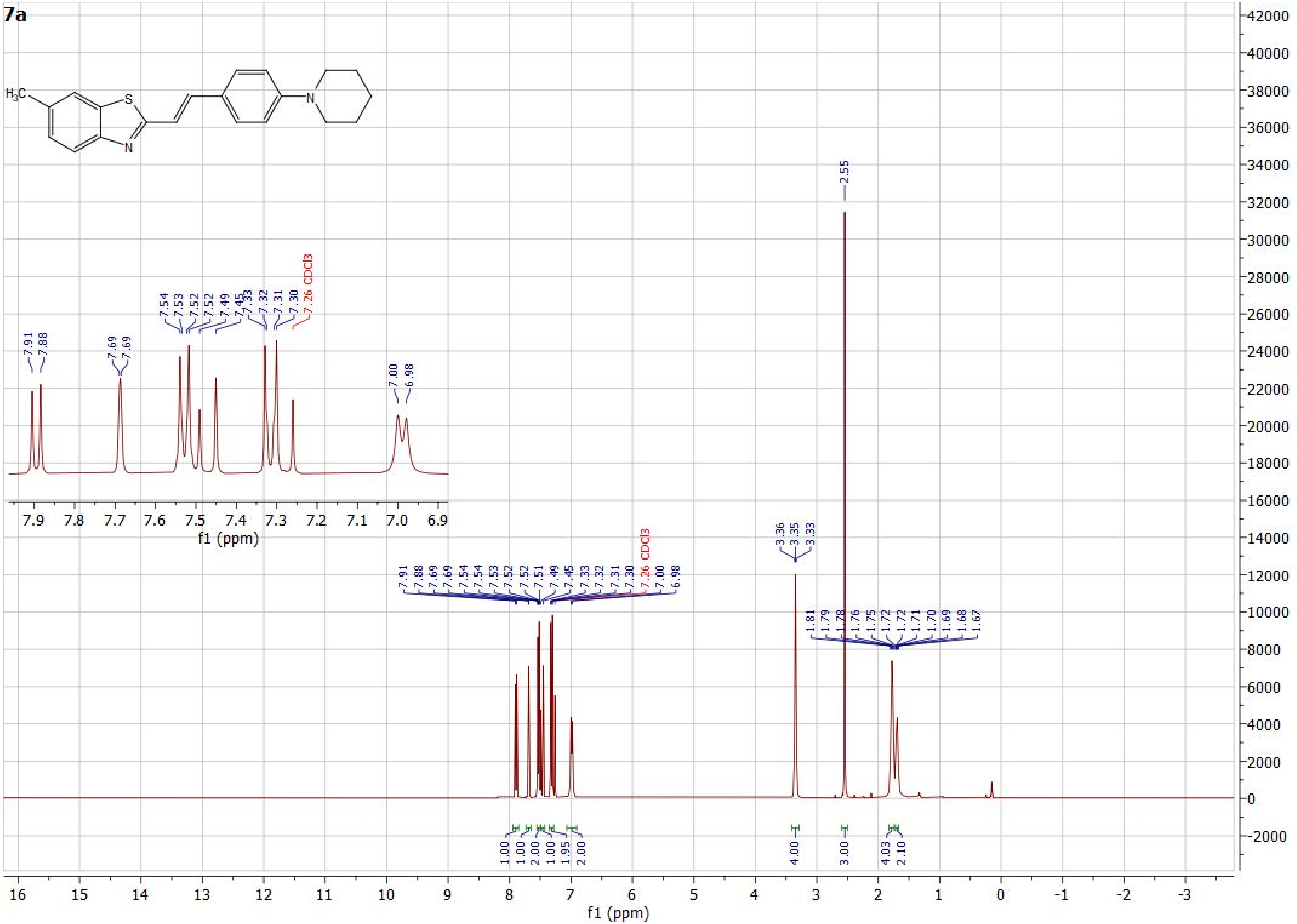

H3C
S
N
N
7.91
7.88
7.69
7.69
7.54
7.53
7.52
7.52
7.49
7.45
7.33
7.32
7.31
7.30
7.26 CDCl3
7.00
6.98
f1 (ppm)
3.36
3.35
3.33
2.55
1.81
1.79
1.78
1.76
1.75
1.72
1.72
1.71
1.70
1.69
1.68
1.67
1.00
1.00
2.00
1.00
1.95
2.00
4.00
3.00
4.03
2.10
f1 (ppm)

7b

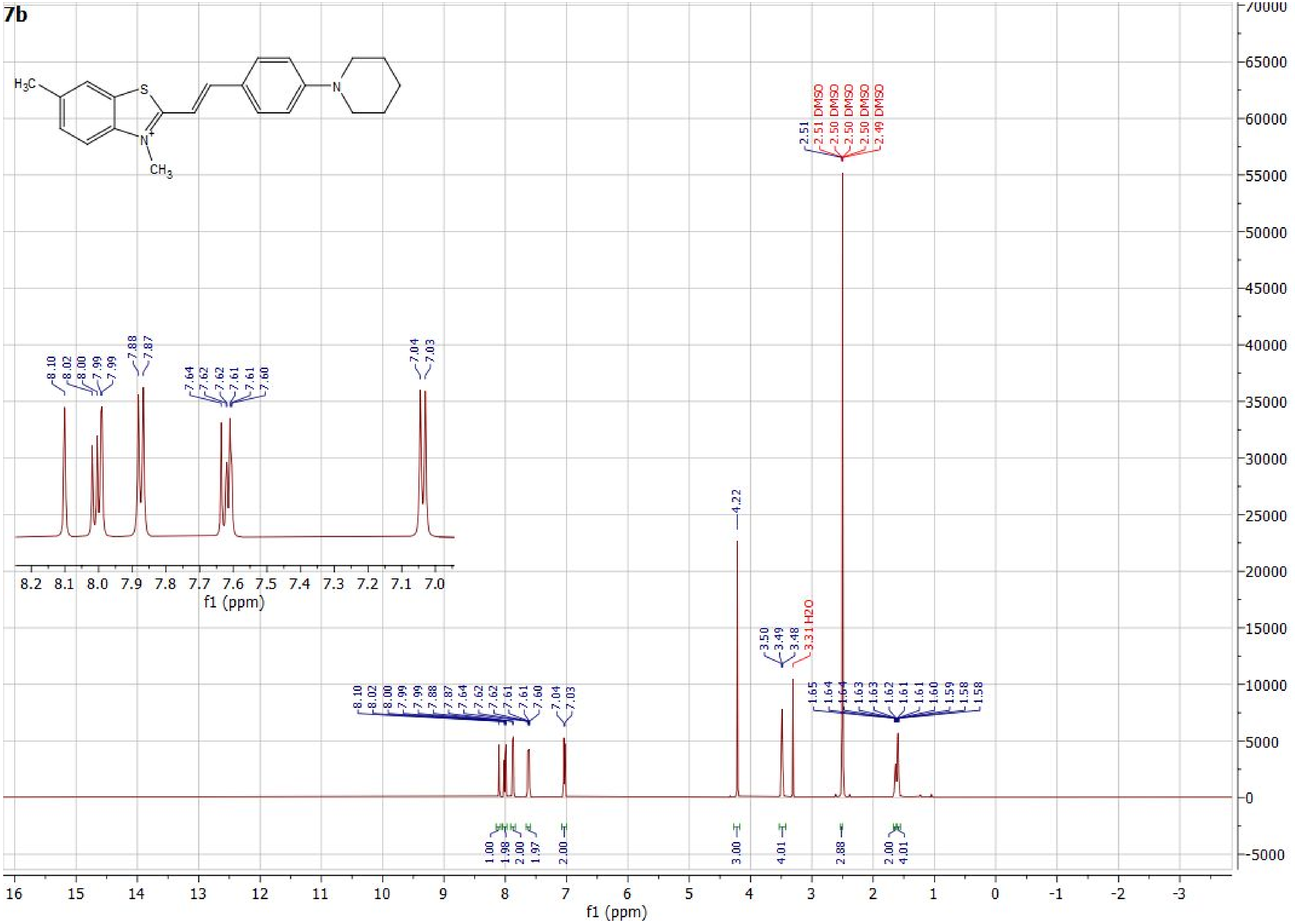

H3C
S
N+
CH3
N
2.51
2.51 DMSO
2.50 DMSO
2.50 DMSO
2.50 DMSO
2.49 DMSO
4.22
3.50
3.49
3.48
3.31 H2O
8.10 8.02 8.00 7.99 7.99 7.88 7.87 7.64 7.62 7.62 7.61 7.61 7.60 7.04 7.03
1.65 1.64 1.64 1.63 1.63 1.62 1.61 1.61 1.60 1.59 1.58 1.58
1.00 1.98 2.00 1.97 2.00 3.00 4.01 2.88 2.00 4.01
f1 (ppm)

8a

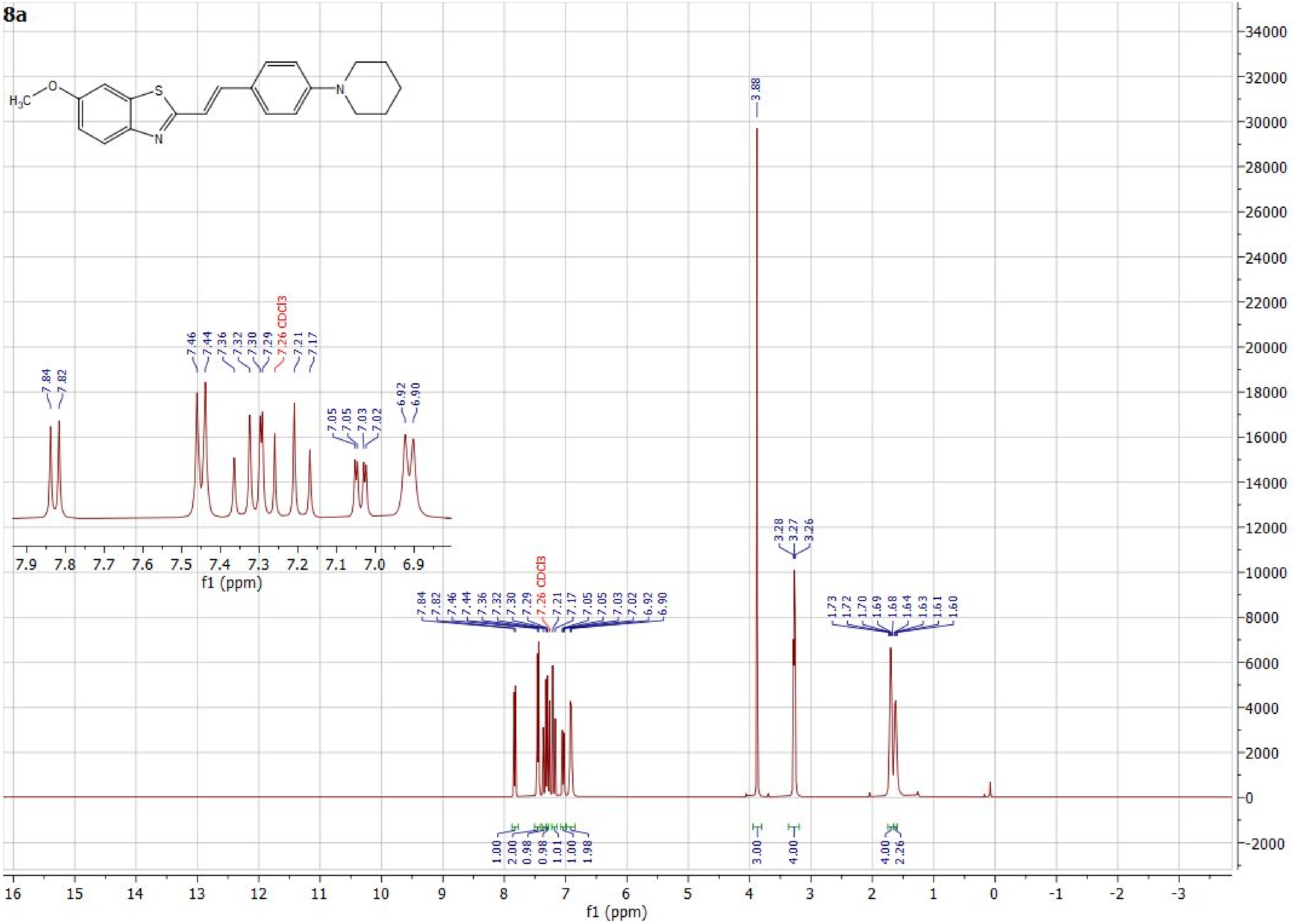
H3C
O
S
N
N
3.88
3.28
3.27
3.26
7.84
7.82
7.46
7.44
7.36
7.32
7.30
7.29
7.26 CDCl3
7.21
7.17
7.05
7.05
7.03
7.02
6.92
6.90
1.73
1.72
1.70
1.69
1.68
1.64
1.63
1.61
1.60
1.00
2.00
0.98
0.98
1.01
1.00
1.98
3.00
4.00
4.00
2.26
f1 (ppm)

8b

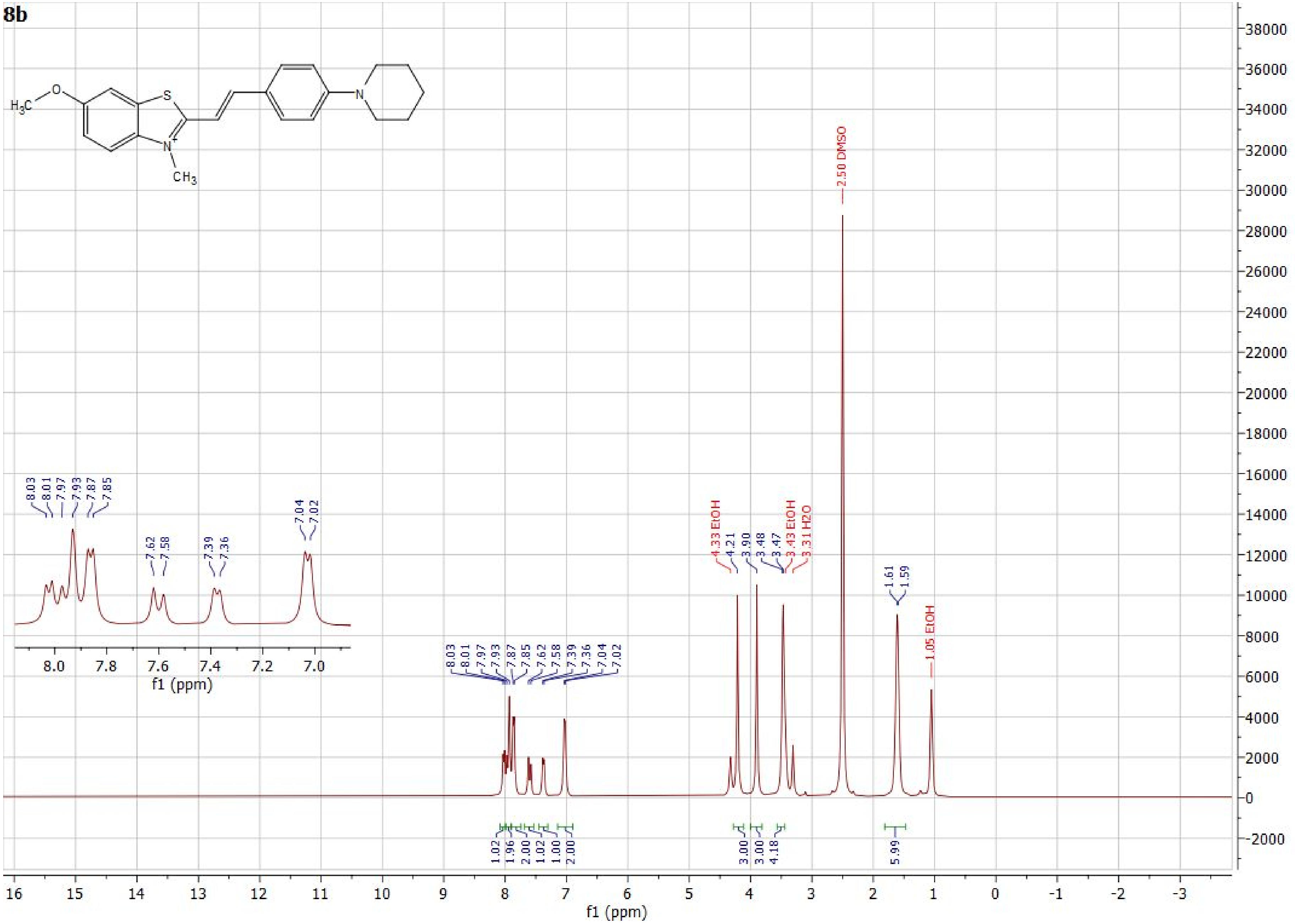

8.03
8.01
7.97
7.93
7.87
7.85
7.62
7.58
7.39
7.36
7.04
7.02
4.33 EtOH
4.21
3.90
3.48
3.47
3.43 EtOH
3.31 H2O
2.50 DMSO
1.61
1.59
1.05 EtOH
1.02
1.96
2.00
1.02
1.00
2.00
3.00
3.00
4.18
5.99
f1 (ppm)

9a (PFSB)

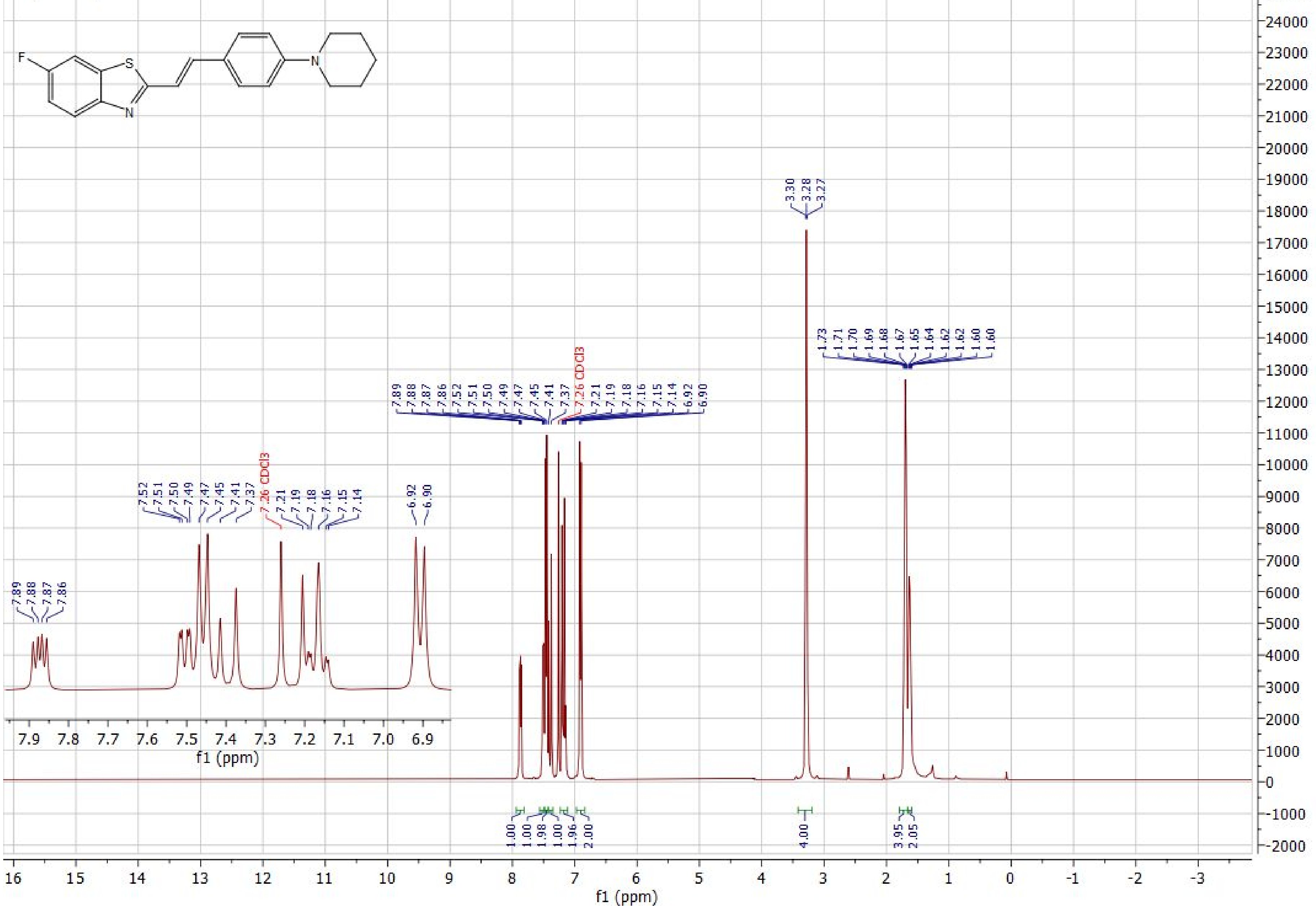

7.89
7.88
7.87
7.86
7.52
7.51
7.50
7.49
7.47
7.45
7.41
7.37
7.26 CDCl3
7.21
7.19
7.18
7.16
7.15
7.14
6.92
6.90
3.30
3.28
3.27
1.73
1.71
1.70
1.69
1.68
1.67
1.65
1.64
1.62
1.62
1.60
1.60
1.00
1.00
1.98
1.00
1.96
2.00
4.00
3.95
2.05
f1 (ppm)

9b

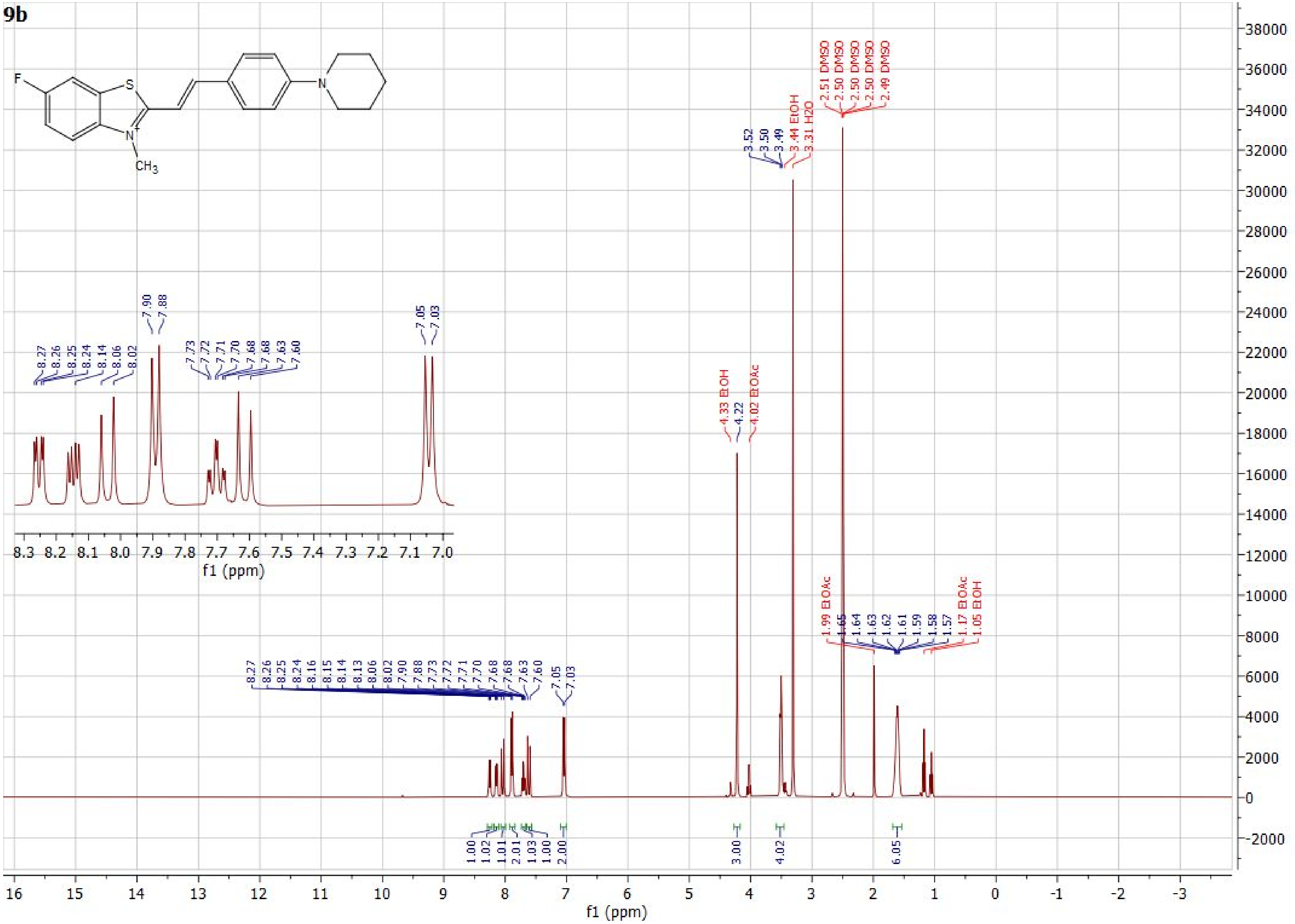

F
S
N+
CH3
N
8.27
8.26
8.25
8.24
8.14
8.06
8.02
7.90
7.88
7.73
7.72
7.71
7.70
7.68
7.68
7.63
7.60
7.05
7.03
f1 (ppm)
4.33 EtOH
4.22
4.02 EtOAc
3.52
3.50
3.49
3.44 EtOH
3.31 H2O
2.51 DMSO
2.50 DMSO
2.50 DMSO
2.50 DMSO
2.49 DMSO
1.99 EtOAc
1.65
1.64
1.63
1.62
1.61
1.59
1.58
1.57
1.17 EtOAc
1.05 EtOH
1.00
1.02
1.01
2.01
1.03
1.00
2.00
3.00
4.02
6.05
f1 (ppm)

10a

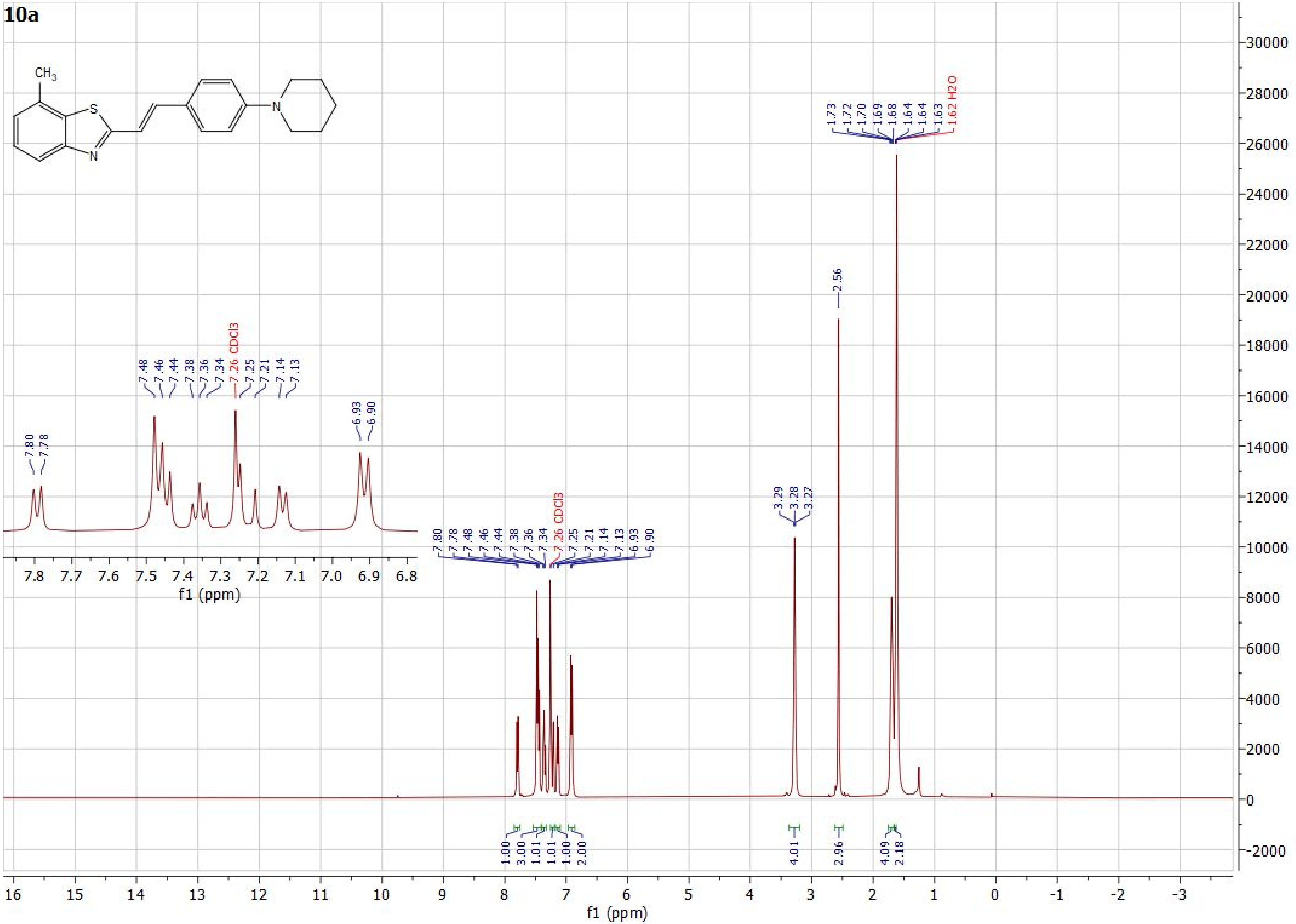
CH3
S
N
N
7.80
7.78
7.48
7.46
7.44
7.38
7.36
7.34
7.26 CDCl3
7.25
7.21
7.14
7.13
6.93
6.90
3.29
3.28
3.27
2.56
1.73
1.72
1.70
1.69
1.68
1.64
1.64
1.63
1.62 H2O
1.00
3.00
1.01
1.01
1.00
2.00
4.01
2.96
4.09
2.18
f1 (ppm)
f1 (ppm)

10b

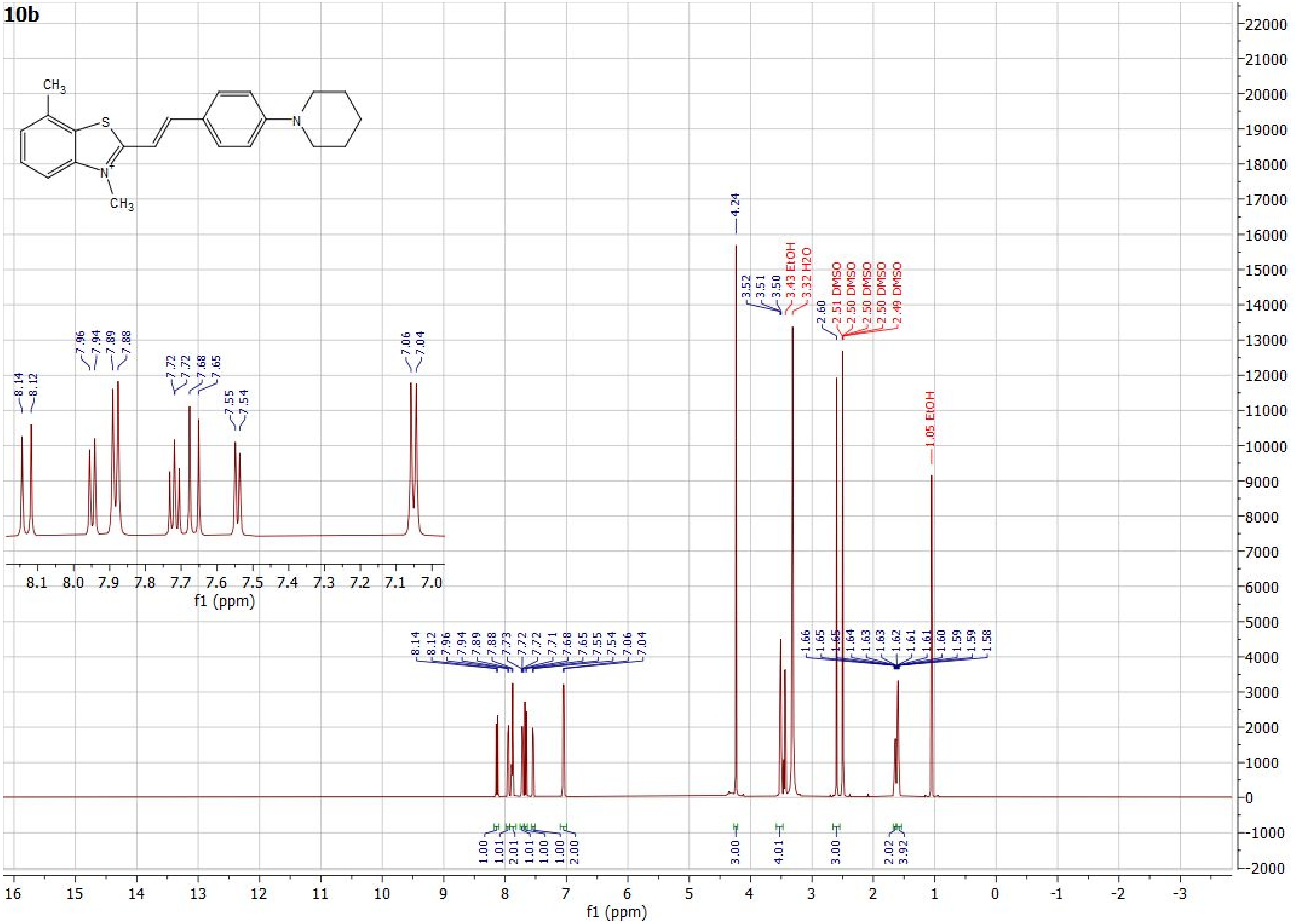

11a

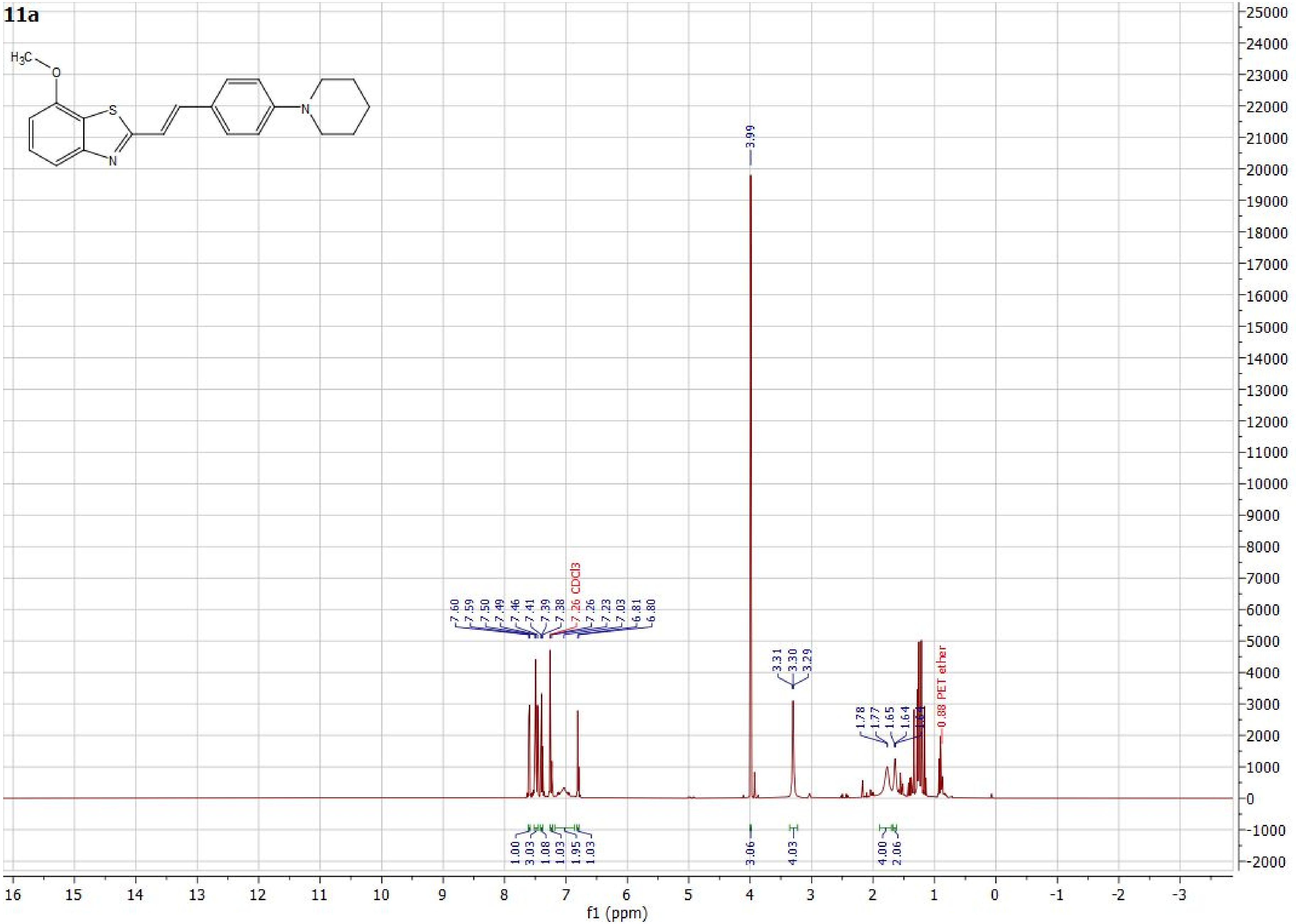

7.60
7.59
7.50
7.49
7.46
7.41
7.39
7.38
7.26 CDCl3
7.26
7.23
7.03
6.81
6.80
3.99
3.31
3.30
3.29
1.78
1.77
1.65
1.64
0.88 PET ether
1.00
3.03
1.08
1.03
1.95
1.03
3.06
4.03
4.00
2.06
f1 (ppm)

11b

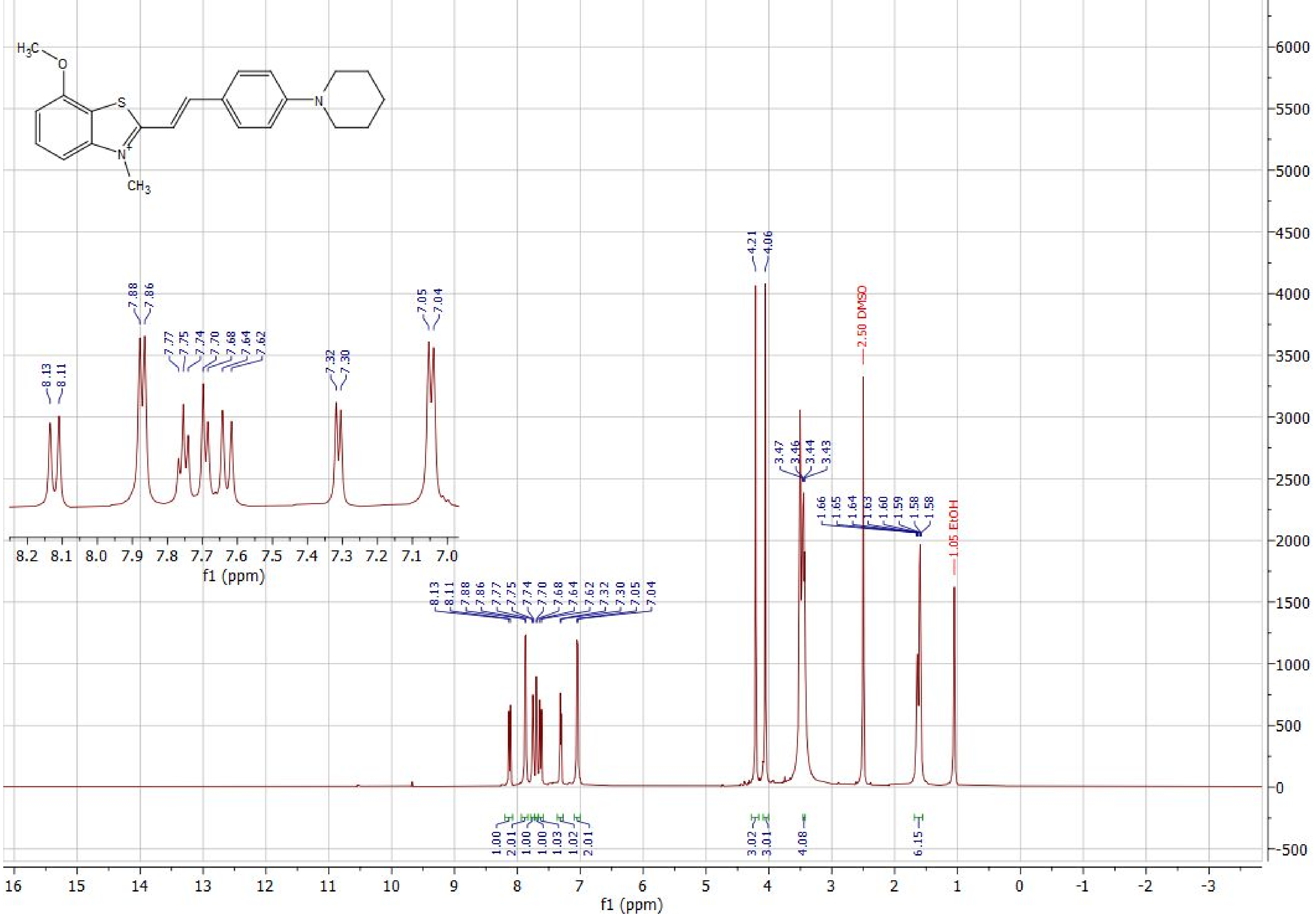

H_3C
O
S
N
N^+
CH_3
8.13
8.11
7.88
7.86
7.77
7.75
7.74
7.70
7.68
7.64
7.62
7.32
7.30
7.05
7.04
f1 (ppm)
4.21
4.06
3.47
3.46
3.44
3.43
2.50 DMSO
1.66
1.65
1.64
1.63
1.60
1.59
1.58
1.58
1.05 EtOH
1.00
2.01
1.00
1.00
1.03
1.02
2.01
3.02
3.01
4.08
6.15
f1 (ppm)

12a

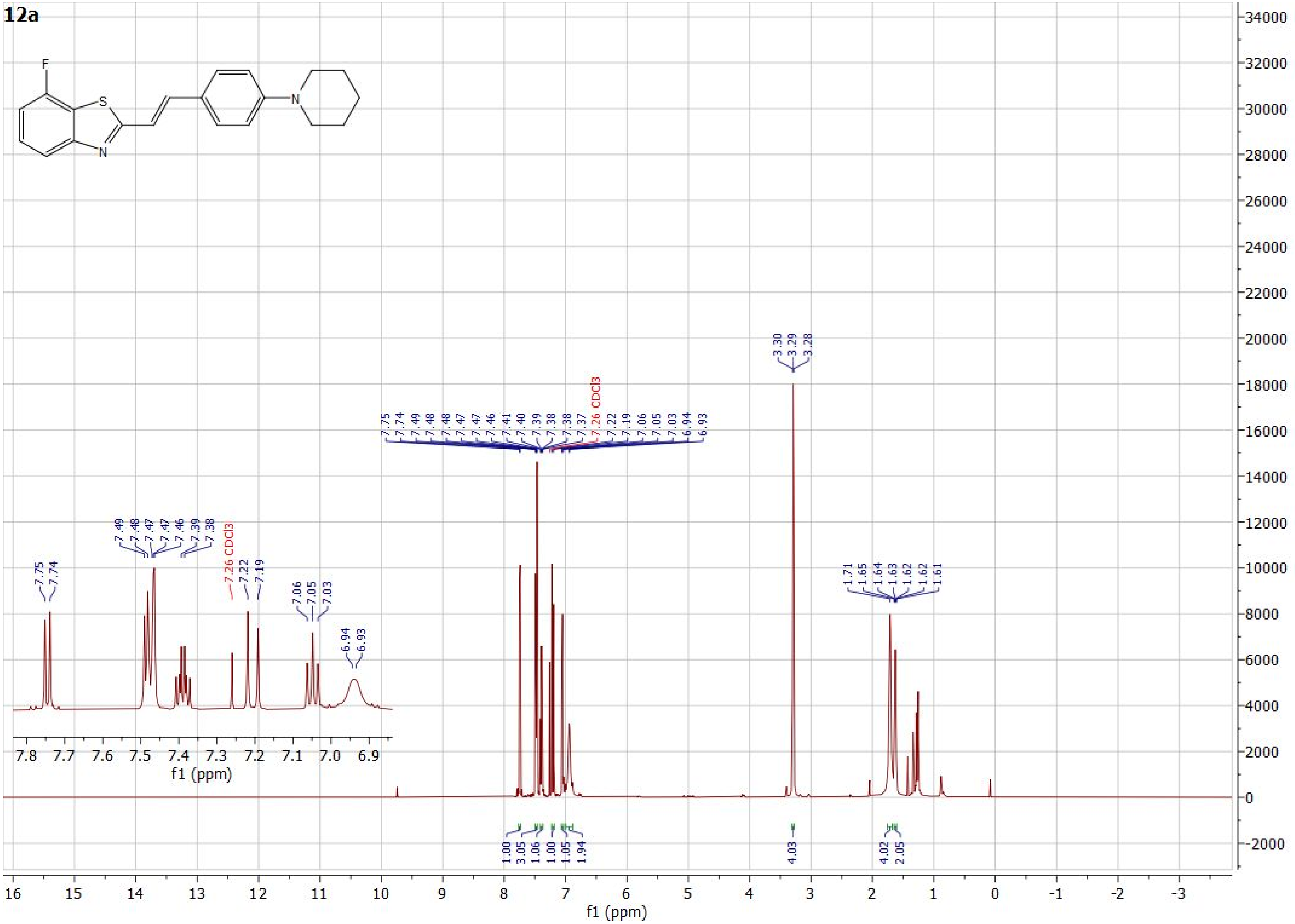

12b

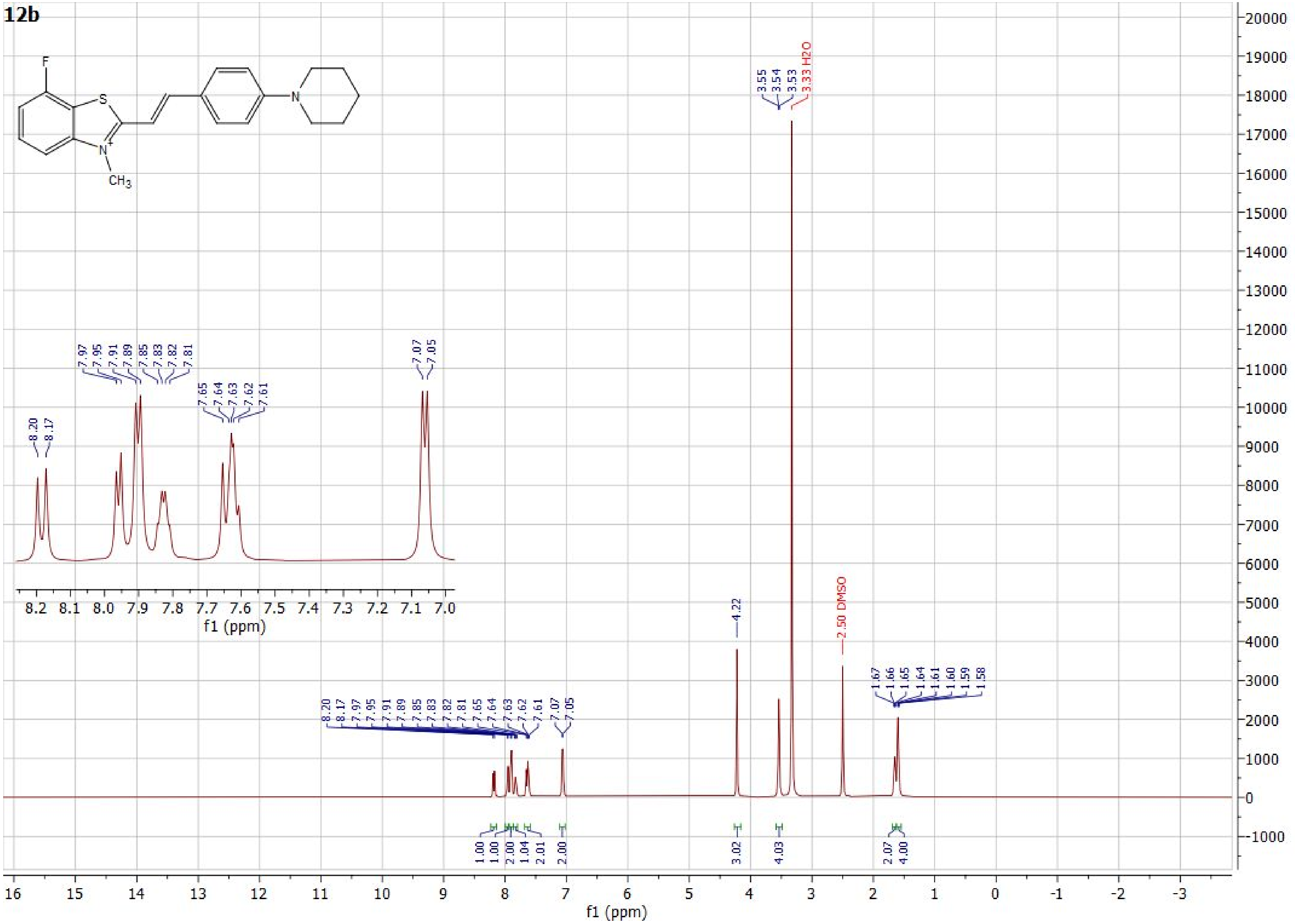

F
S
N+
CH3
N
8.20
8.17
7.97
7.95
7.91
7.89
7.85
7.83
7.82
7.81
7.65
7.64
7.63
7.62
7.61
7.07
7.05
4.22
3.55
3.54
3.53
3.33 H2O
2.50 DMSO
1.67
1.66
1.65
1.64
1.61
1.60
1.59
1.58
1.00
1.00
2.00
1.04
2.01
2.00
3.02
4.03
2.07
4.00
f1 (ppm)

13a

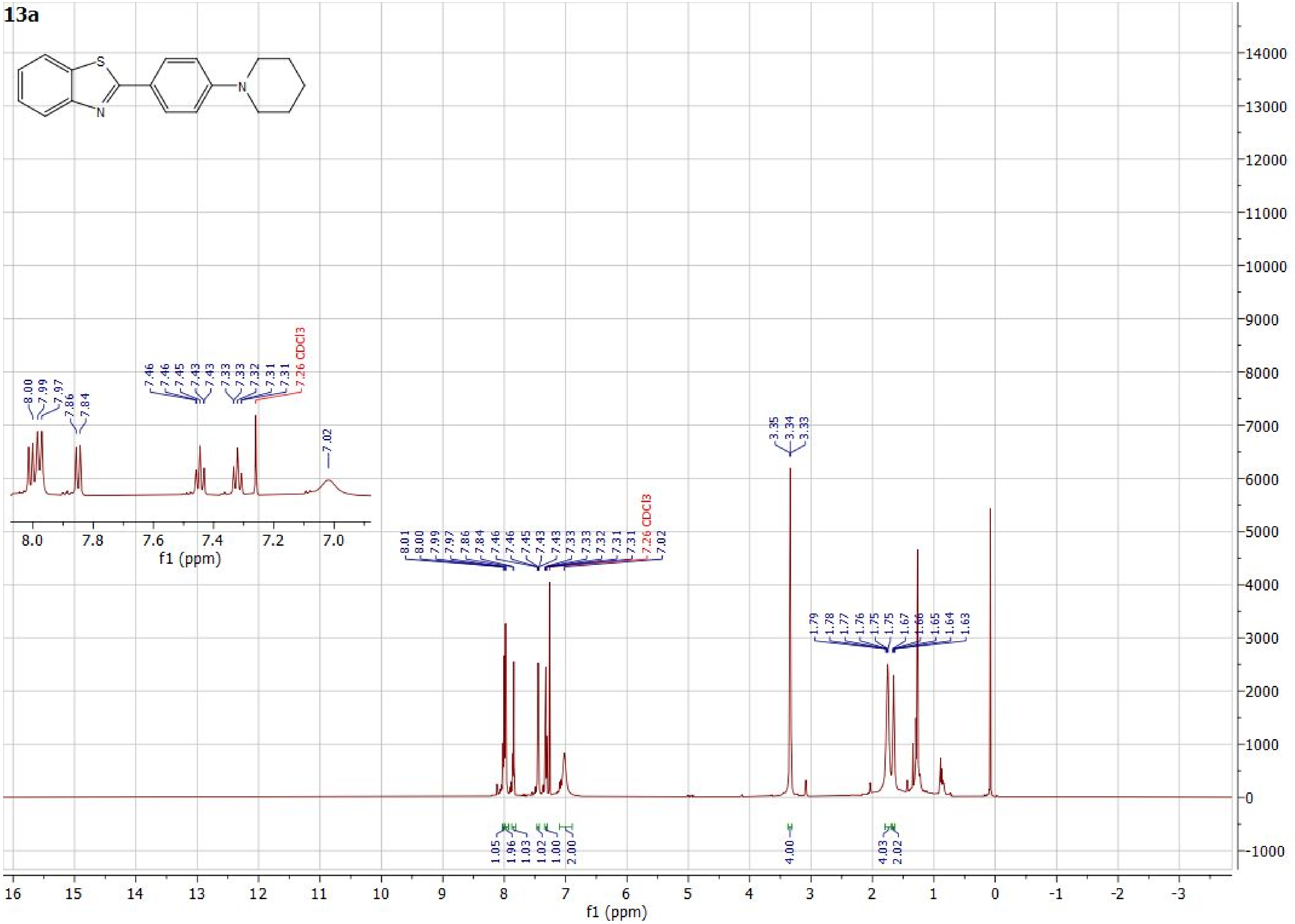

S
N
N
8.01
8.00
7.99
7.97
7.86
7.84
7.46
7.46
7.45
7.43
7.43
7.33
7.33
7.32
7.31
7.31
7.26 CDCl3
7.02
3.35
3.34
3.33
1.79
1.78
1.77
1.76
1.75
1.75
1.67
1.66
1.65
1.64
1.63
1.05
1.96
1.03
1.02
1.00
2.00
4.00
4.03
2.02
f1 (ppm)

13b

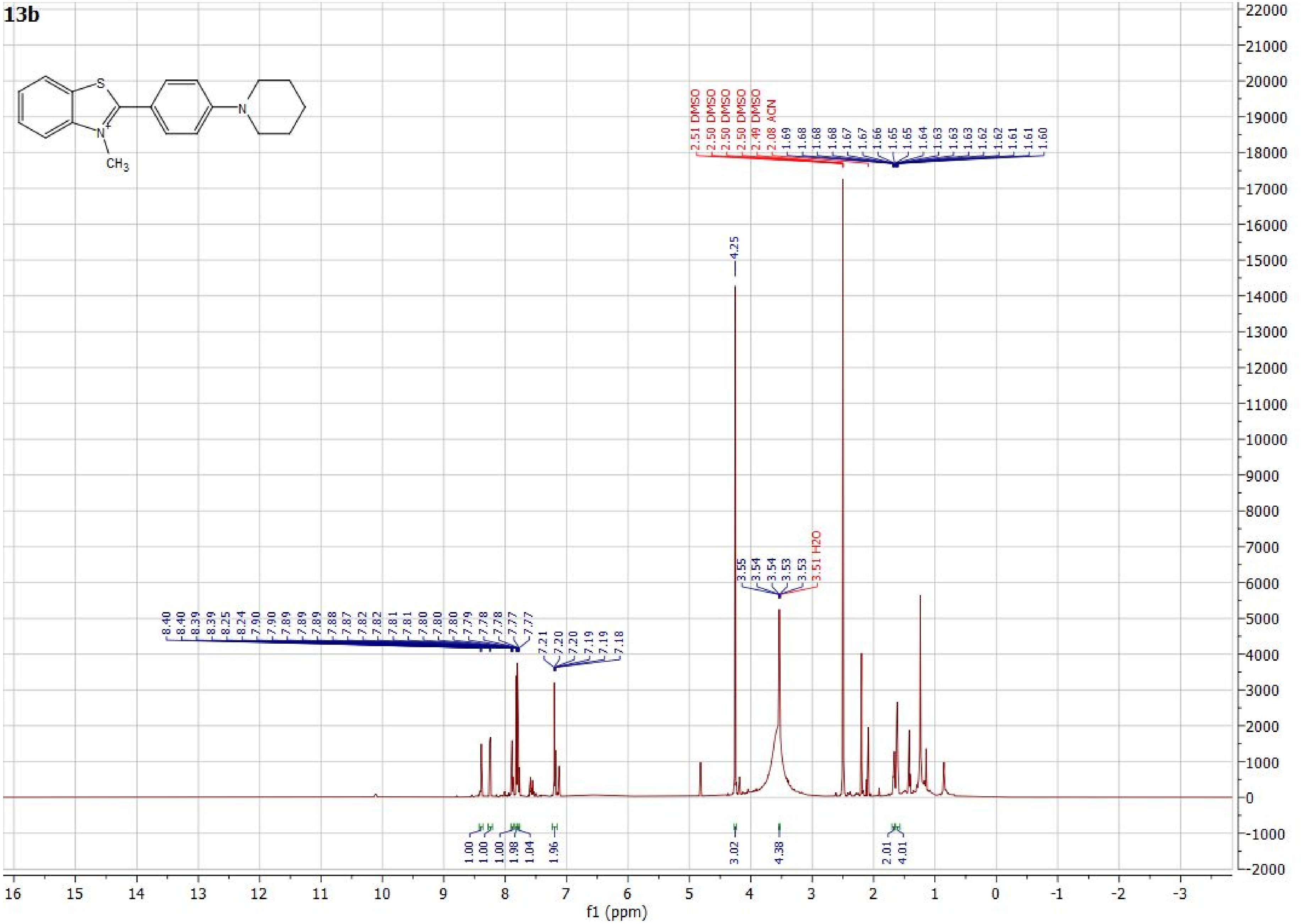

CH3
2.51 DMSO
2.50 DMSO
2.50 DMSO
2.50 DMSO
2.49 DMSO
2.08 ACN
4.25
3.51 H2O
f1 (ppm)

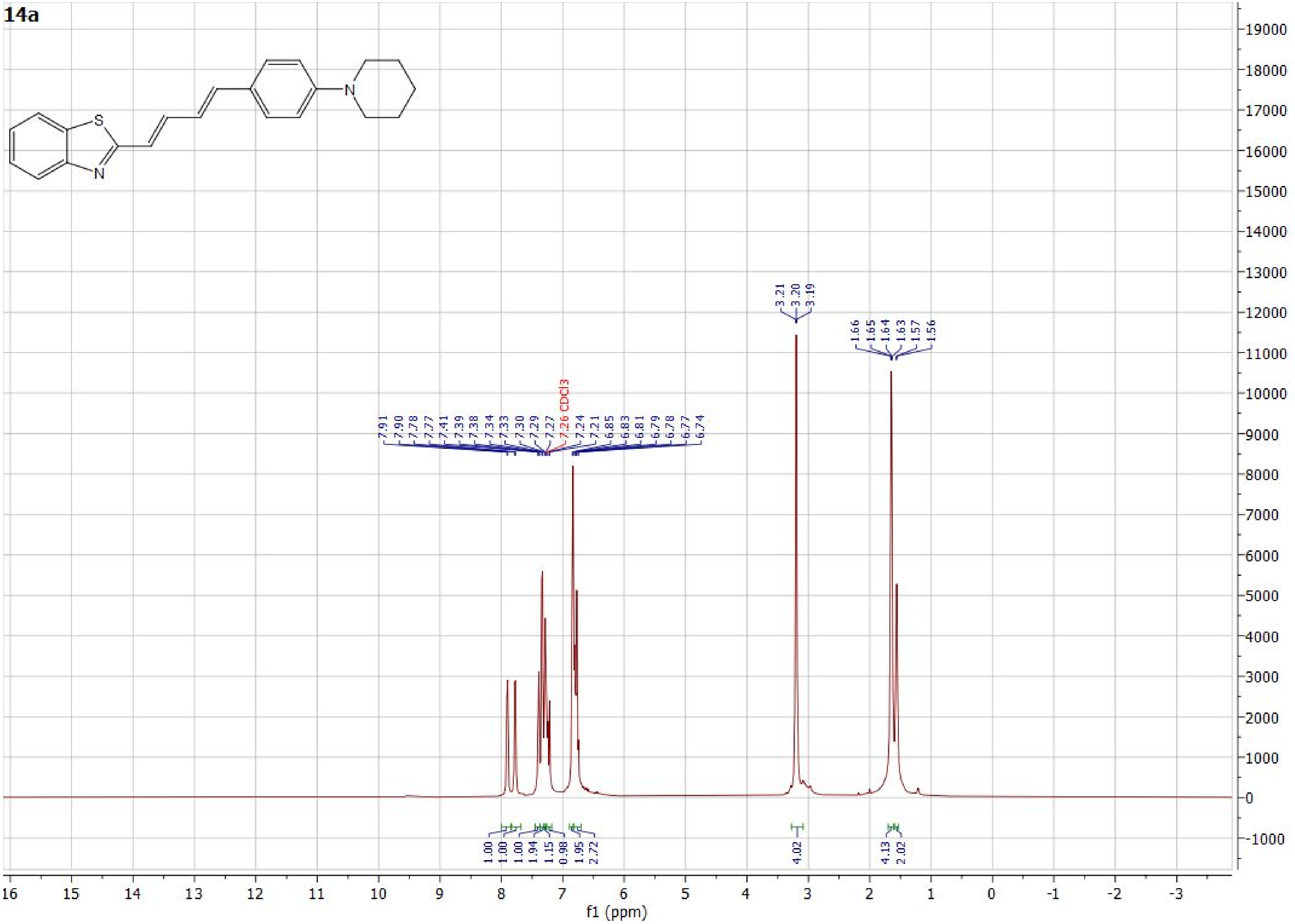
14a
S
N
N
7.91
7.90
7.78
7.77
7.41
7.39
7.38
7.34
7.33
7.30
7.29
7.27
7.26 CDCl3
7.24
7.21
6.85
6.83
6.81
6.79
6.78
6.77
6.74
3.21
3.20
3.19
1.66
1.65
1.64
1.63
1.57
1.56
1.00
1.00
1.00
1.94
1.15
0.98
1.95
2.72
4.02
4.13
2.02
f1 (ppm)

14b

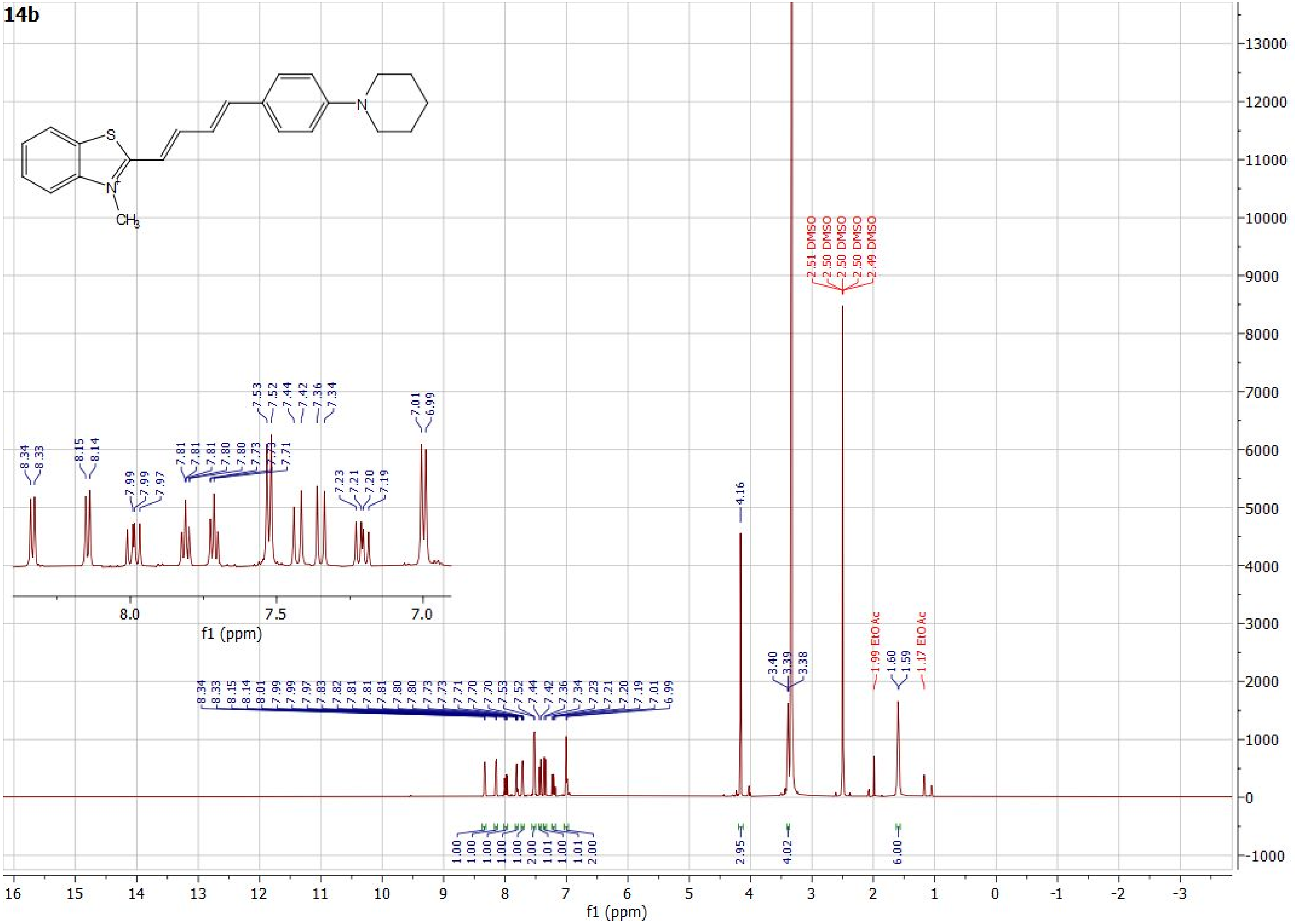

15a

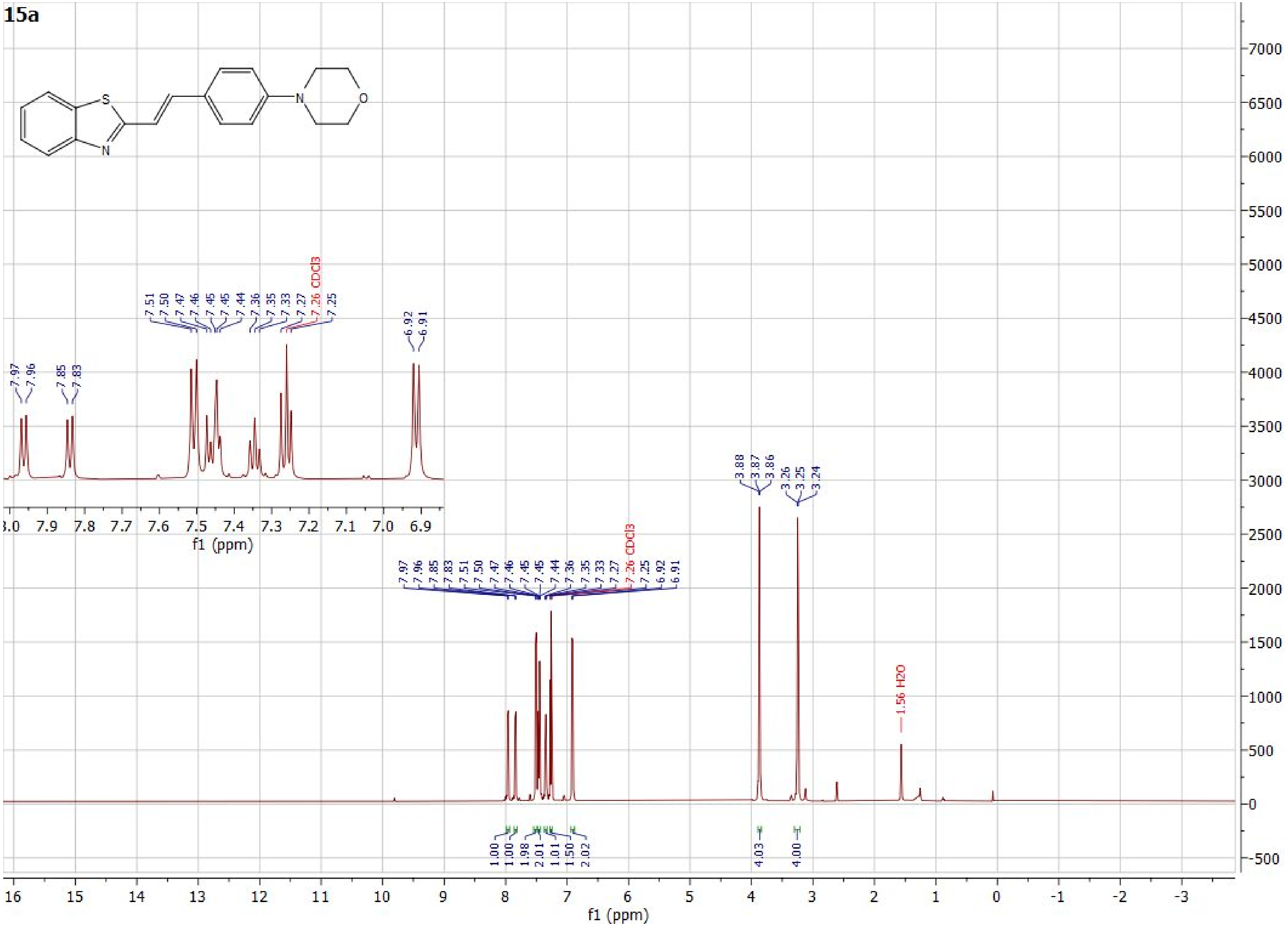

15b

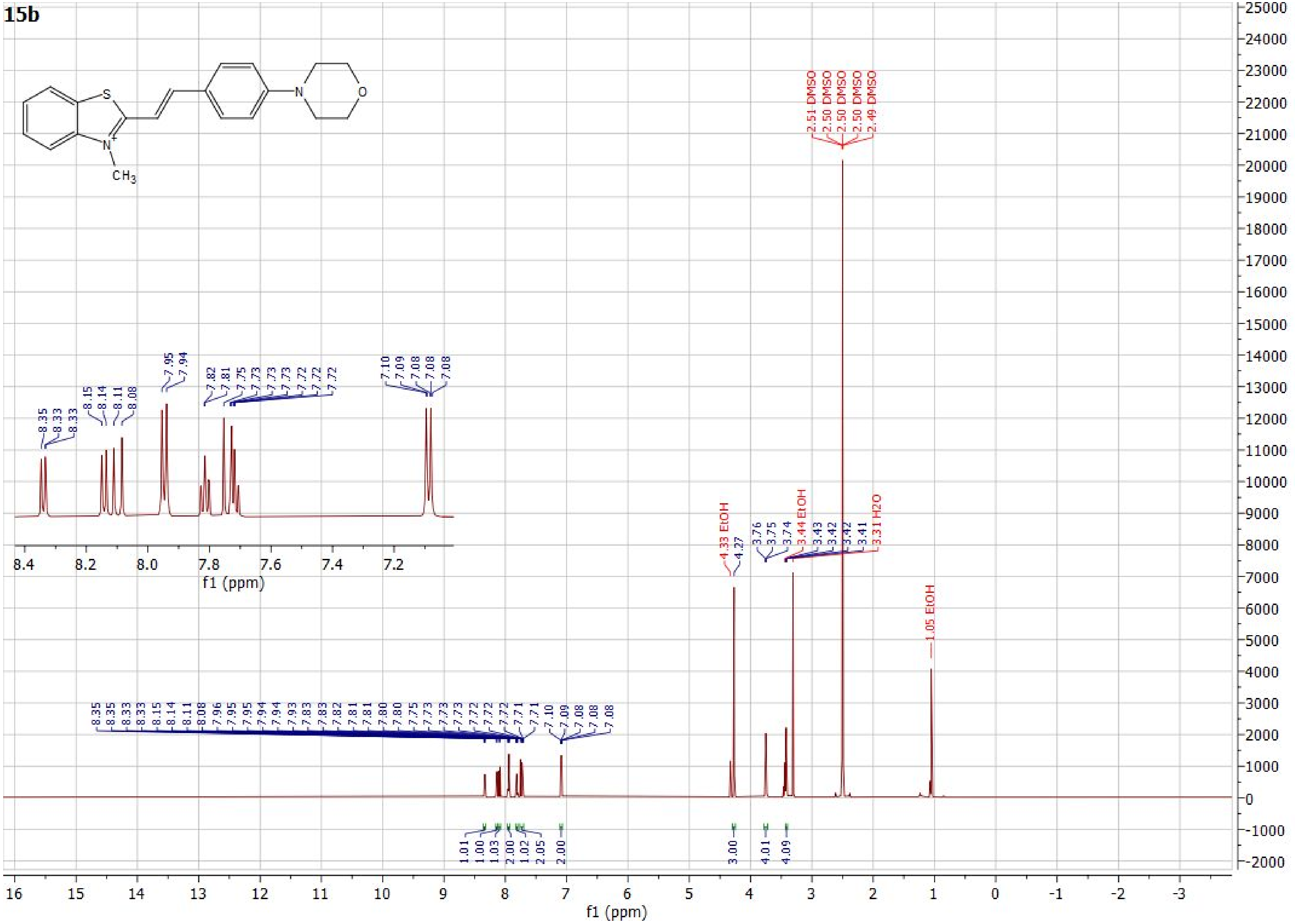
S
N+
CH3
N
O
8.35
8.33
8.33
8.15
8.14
8.11
8.08
7.95
7.94
7.82
7.81
7.75
7.73
7.73
7.72
7.72
7.72
7.10
7.09
7.08
7.08
7.08
f1 (ppm)
2.51 DMSO
2.50 DMSO
2.50 DMSO
2.50 DMSO
2.49 DMSO
4.33 EtOH
4.27
3.76
3.75
3.74
3.44 EtOH
3.43
3.42
3.42
3.41
3.31 H2O
1.05 EtOH
1.01
1.00
1.03
2.00
1.02
2.05
2.00
3.00
4.01
4.09
f1 (ppm)

16a

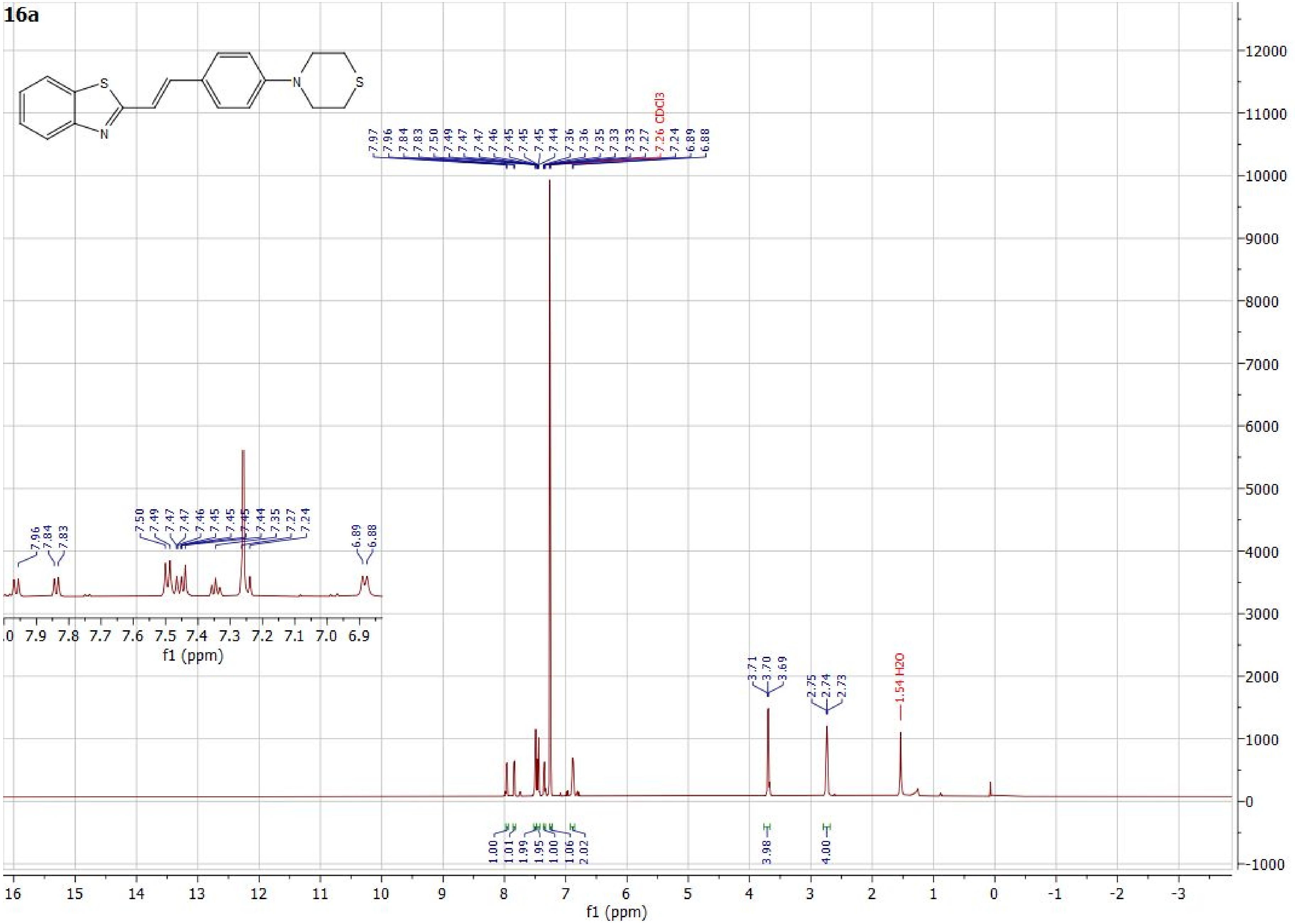

16b

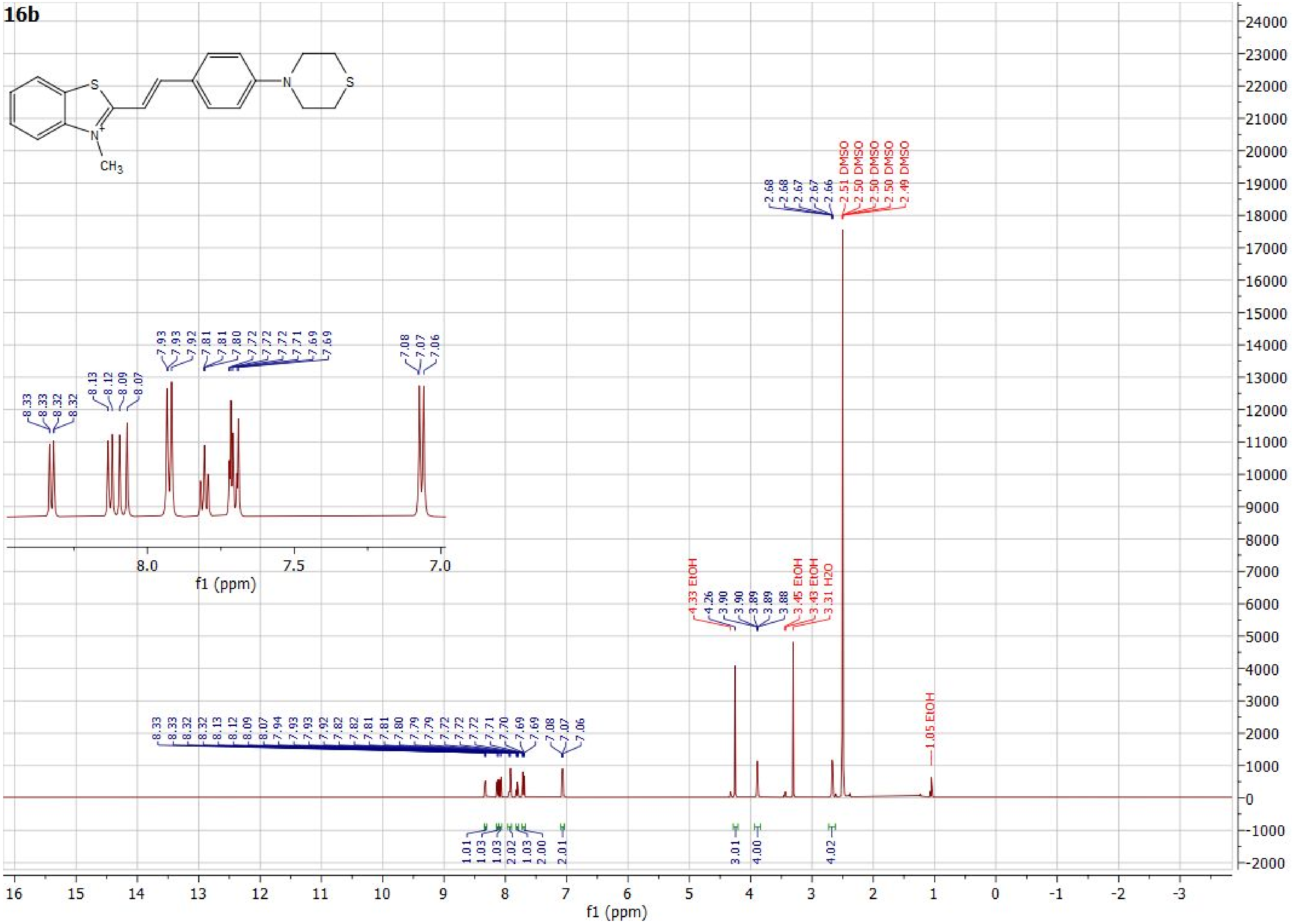

17a

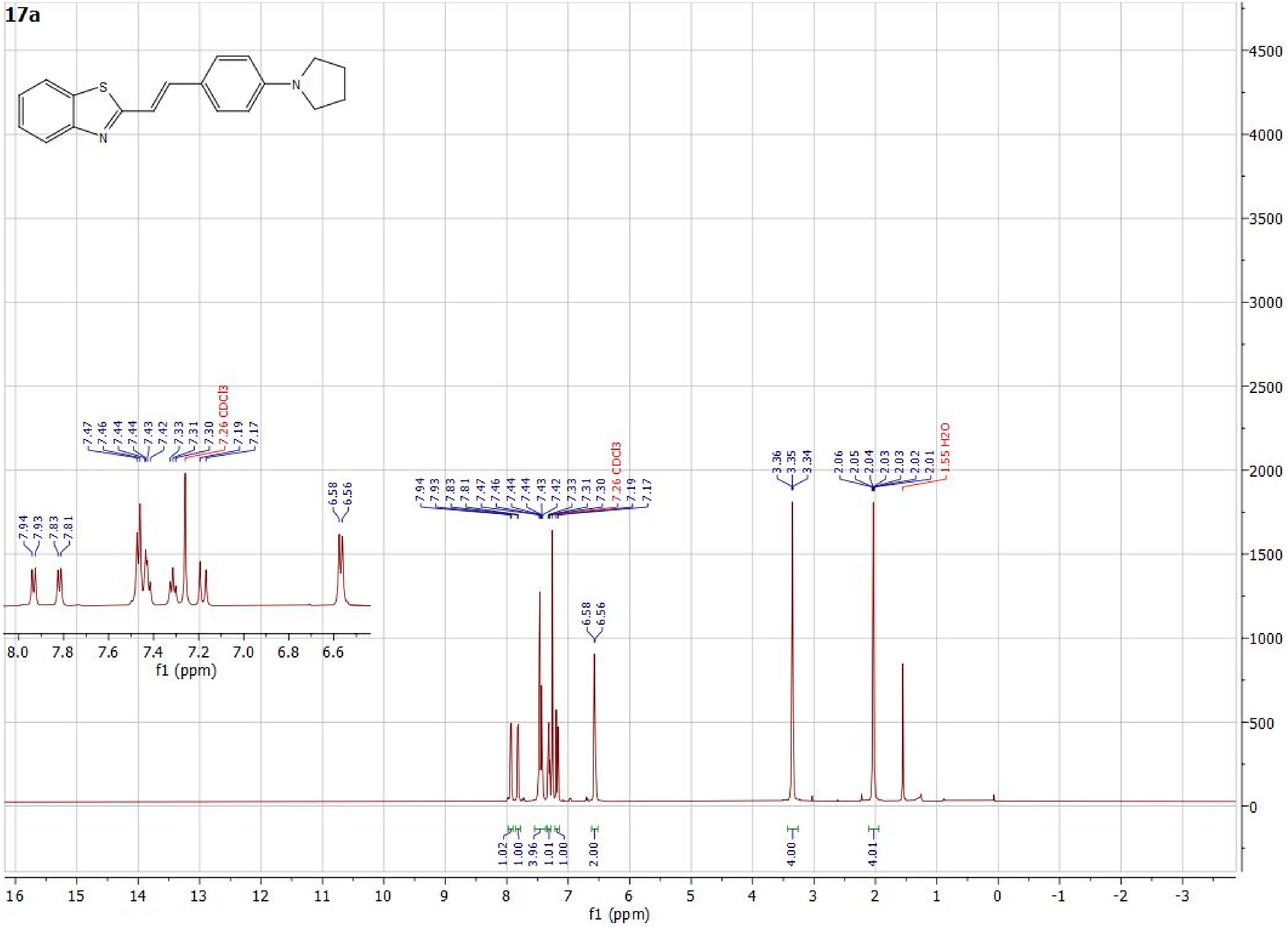

S
N
N
7.94
7.93
7.83
7.81
7.47
7.46
7.44
7.44
7.43
7.42
7.33
7.31
7.30
7.26 CDCl3
7.19
7.17
6.58
6.56
3.36
3.35
3.34
2.06
2.05
2.04
2.03
2.03
2.02
2.01
1.55 H2O
1.02
1.00
3.96
1.01
1.00
2.00
4.00
4.01
f1 (ppm)

17b

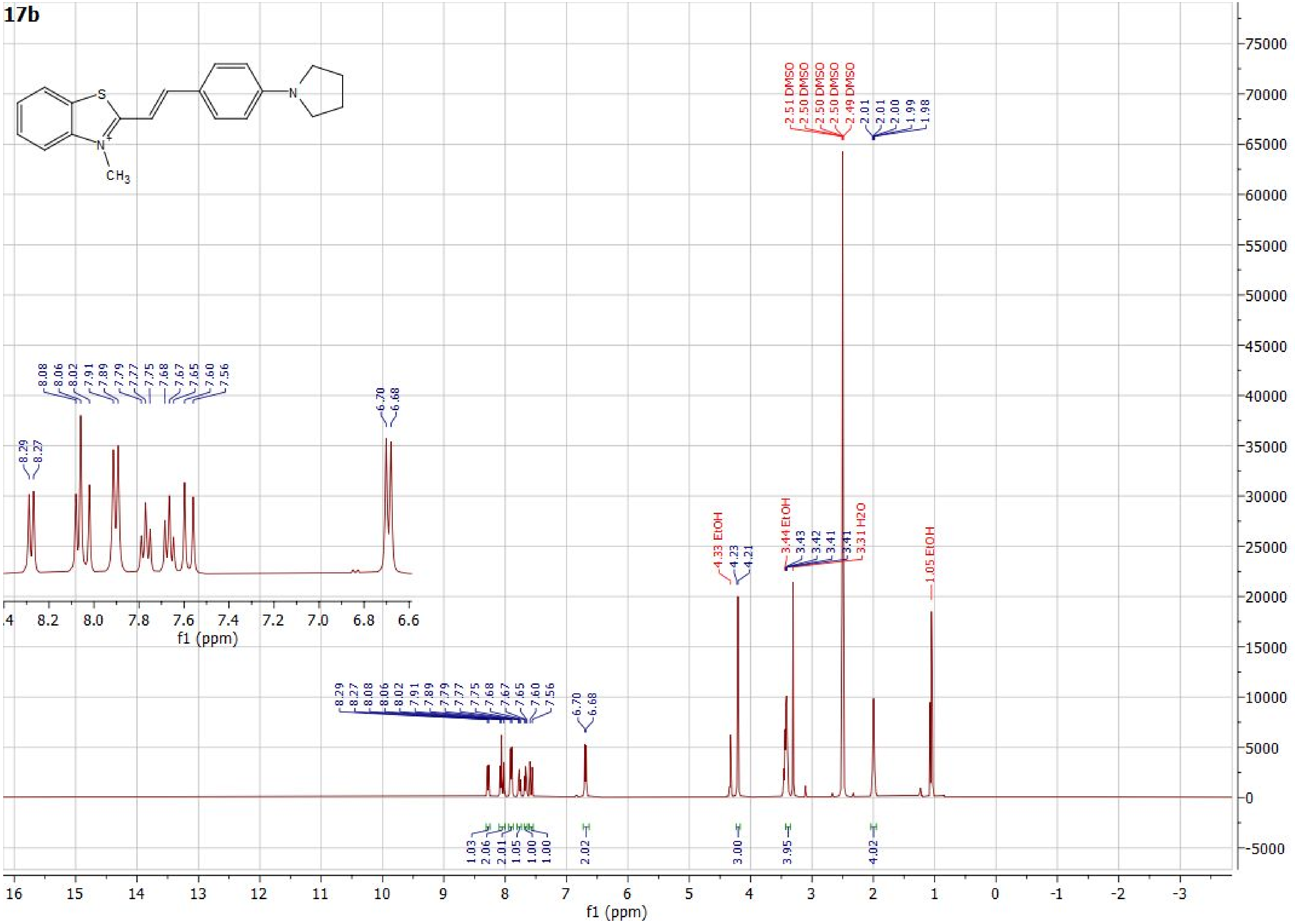

S
N+
CH3
N
2.51 DMSO
2.50 DMSO
2.50 DMSO
2.50 DMSO
2.49 DMSO
2.01
2.01
2.00
1.99
1.98
8.29
8.27
8.08
8.06
8.02
7.91
7.89
7.79
7.77
7.75
7.68
7.67
7.65
7.60
7.56
6.70
6.68
4.33 EtOH
4.23
4.21
3.44 EtOH
3.43
3.42
3.41
3.41
3.31 H2O
1.05 EtOH
1.03
2.06
2.01
1.05
1.00
1.00
2.02
3.00
3.95
4.02
f1 (ppm)
f1 (ppm)

18a

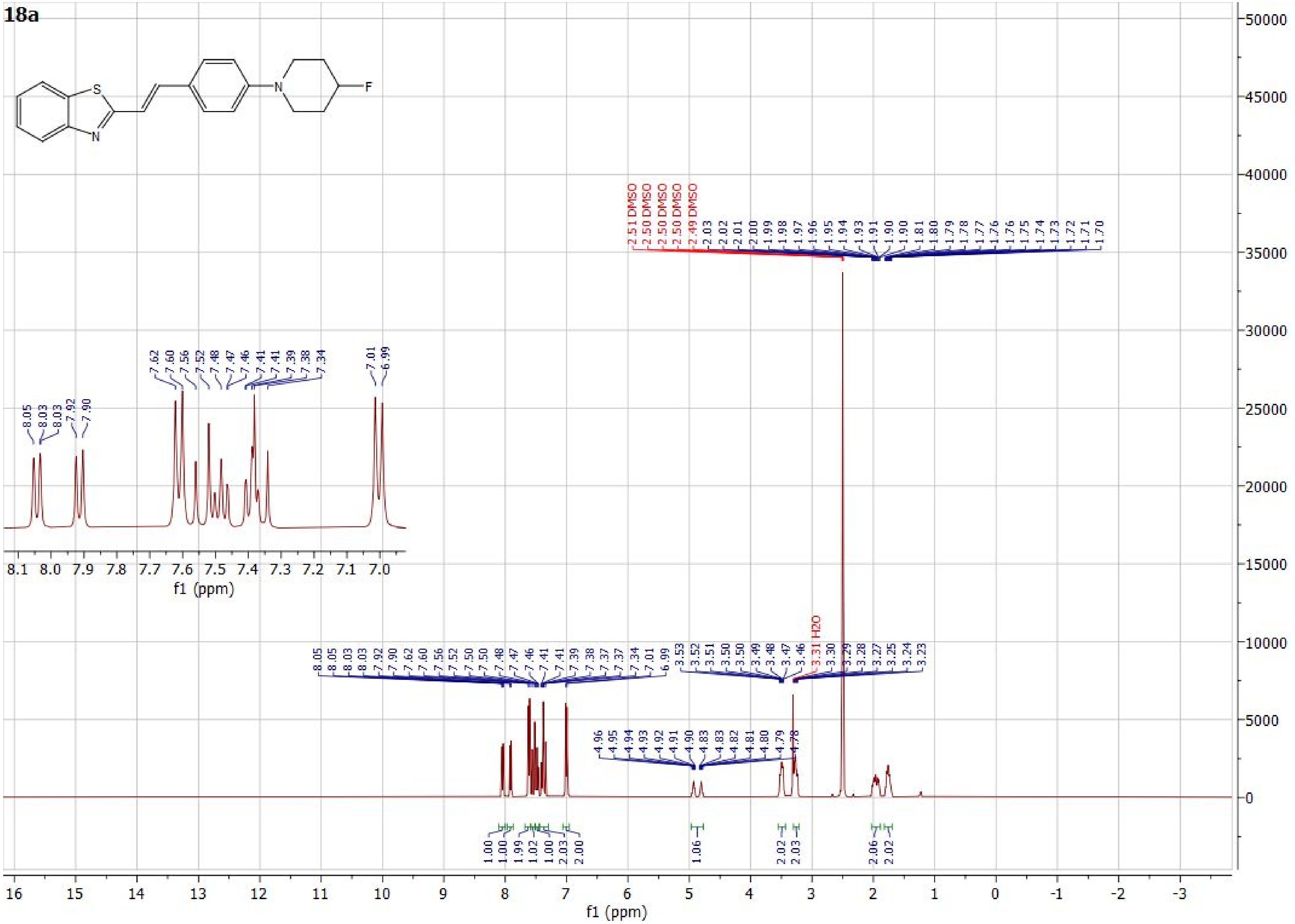

18b

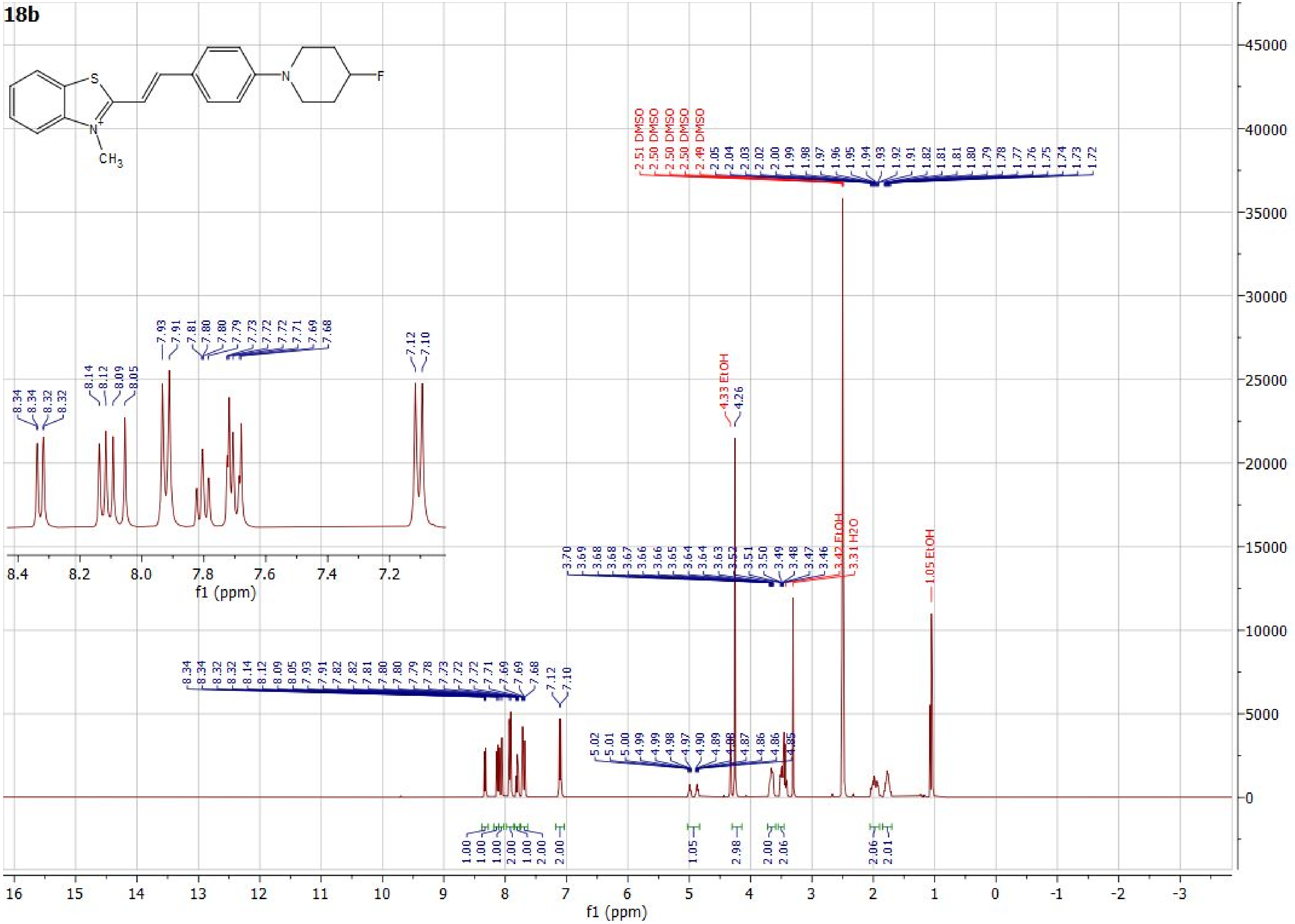

19a

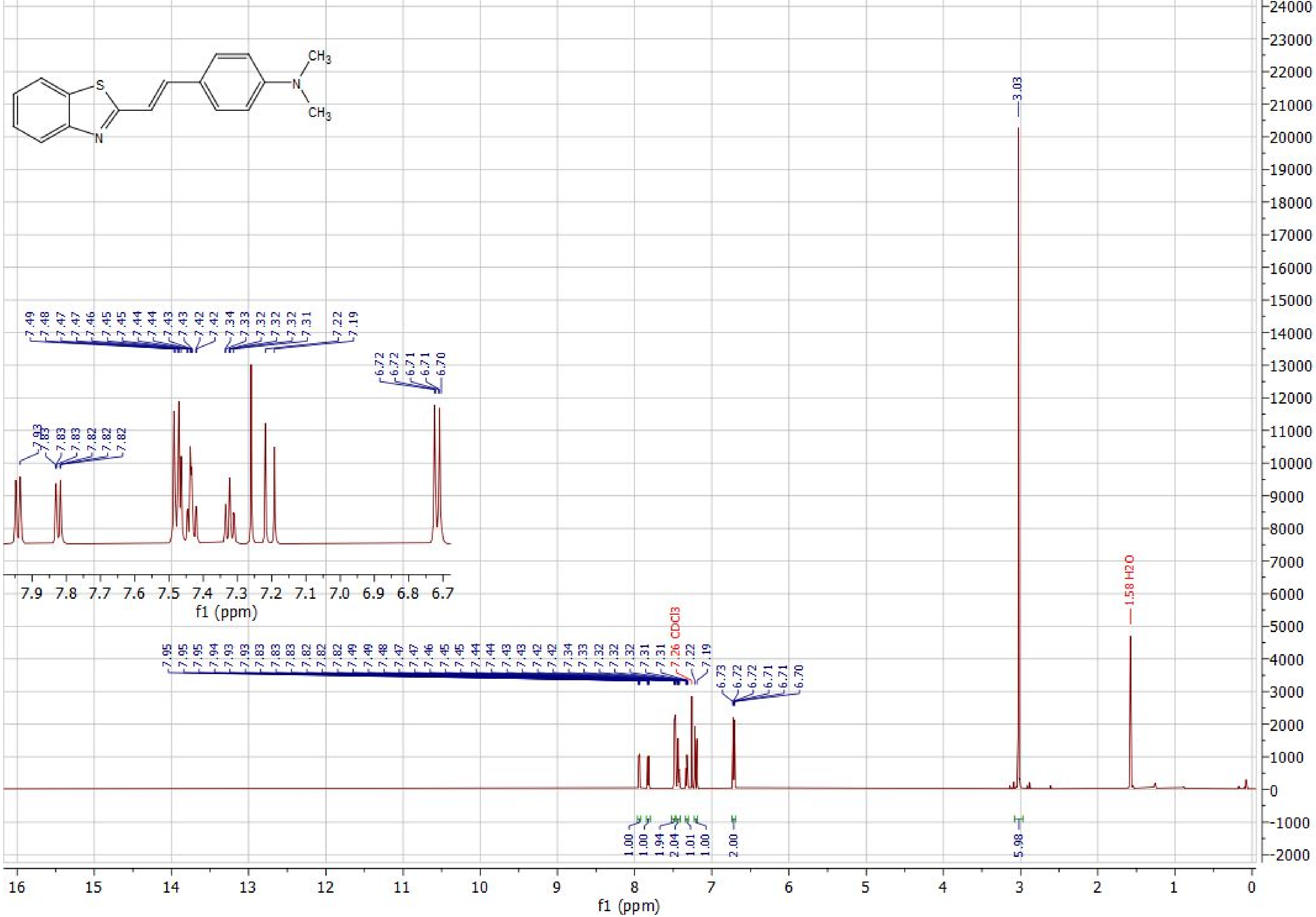

CH3
N
S
N
CH3
7.49 7.48 7.47 7.47 7.46 7.45 7.45 7.44 7.44 7.43 7.43 7.42 7.42 7.34 7.33 7.32 7.32 7.32 7.31 7.22 7.19
6.72 6.72 6.71 6.71 6.70
7.93 7.83 7.83 7.82 7.82 7.82
7.9 7.8 7.7 7.6 7.5 7.4 7.3 7.2 7.1 7.0 6.9 6.8 6.7
f1 (ppm)
3.03
1.58 H2O
7.26 CDCl3
7.95 7.95 7.95 7.94 7.93 7.93 7.83 7.83 7.83 7.82 7.82 7.82 7.49 7.49 7.48 7.47 7.47 7.46 7.45 7.45 7.44 7.44 7.43 7.43 7.42 7.42 7.34 7.33 7.32 7.32 7.32 7.31 7.31 7.22 7.19
6.73 6.72 6.72 6.71 6.71 6.70
1.00 1.00 1.94 2.04 1.01 1.00 2.00 5.98
24000 23000 22000 21000 20000 19000 18000 17000 16000 15000 14000 13000 12000 11000 10000 9000 8000 7000 6000 5000 4000 3000 2000 1000 0 -1000 -2000
16 15 14 13 12 11 10 9 8 7 6 5 4 3 2 1 0
f1 (ppm)

19b

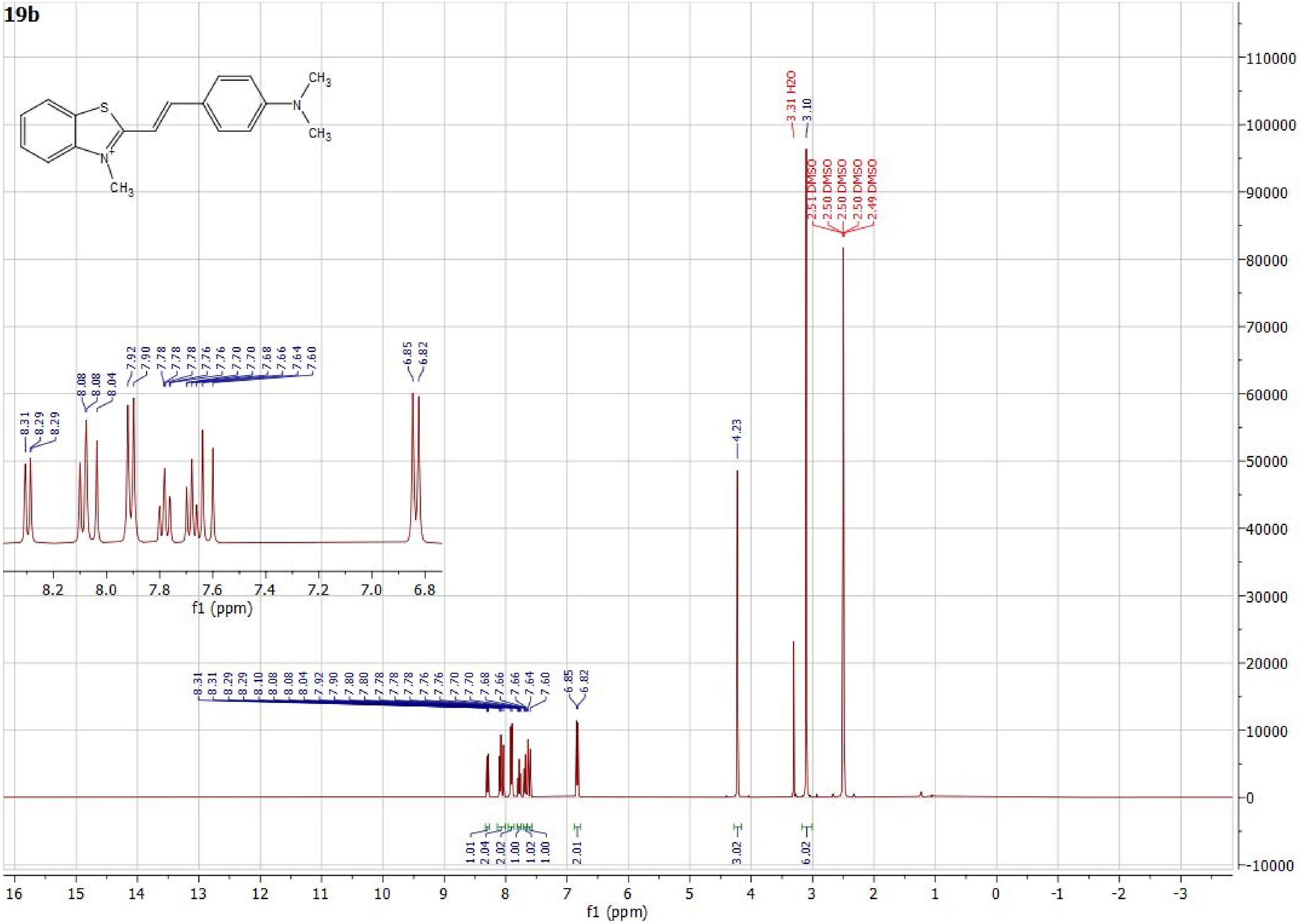

3.31 H2O
3.10
2.51 DMSO
2.50 DMSO
2.50 DMSO
2.50 DMSO
2.49 DMSO
4.23
6.85
6.82
f1 (ppm)

20

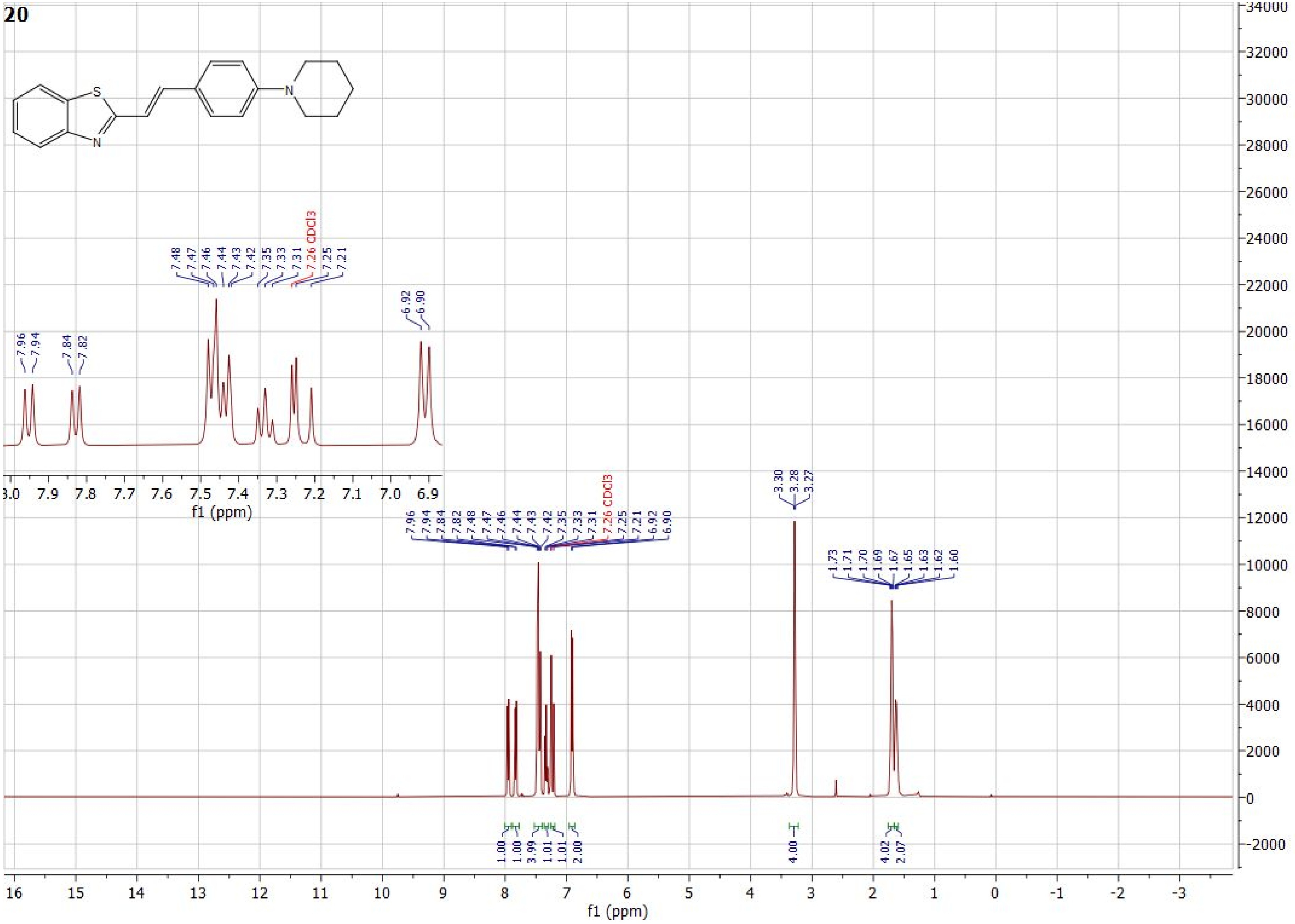
S
N
N
7.96
7.94
7.84
7.82
7.48
7.47
7.46
7.44
7.43
7.42
7.35
7.33
7.31
7.26 CDCl3
7.25
7.21
6.92
6.90
3.30
3.28
3.27
1.73
1.71
1.70
1.69
1.67
1.65
1.63
1.62
1.60
1.00
1.00
3.99
1.01
1.01
2.00
4.00
4.02
2.07
f1 (ppm)
f1 (ppm)

43

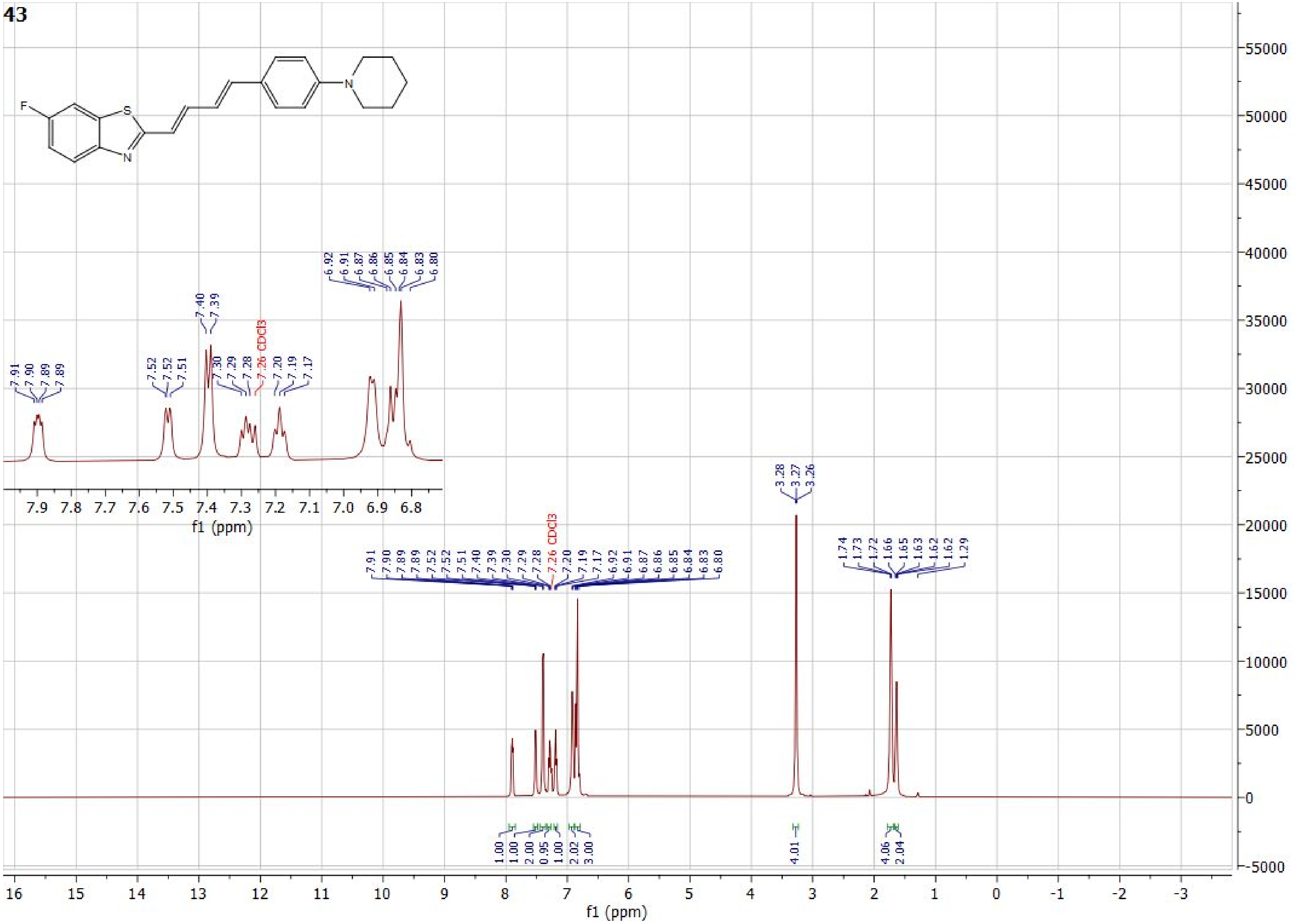

44

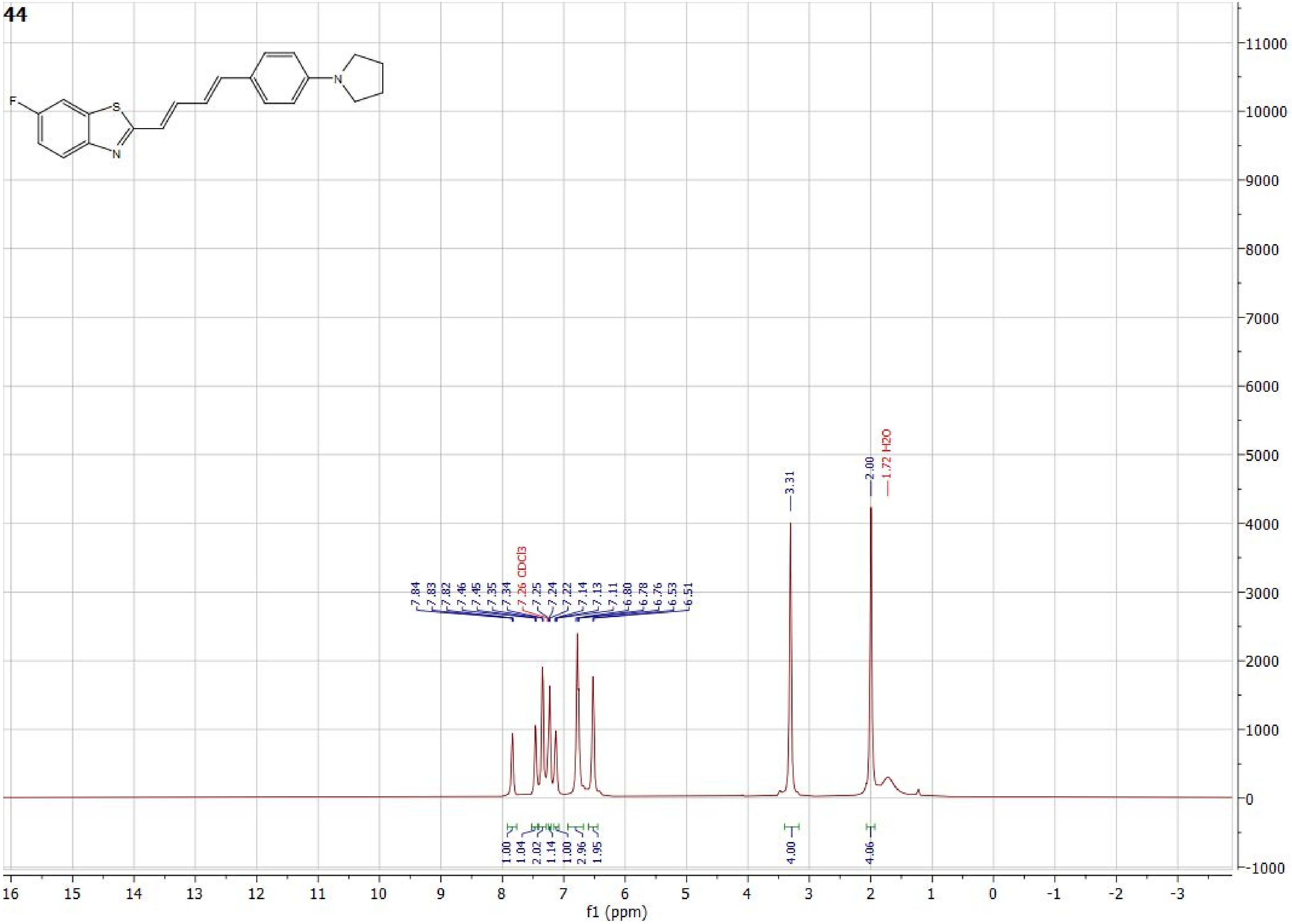

F
S
N
N
7.84
7.83
7.82
7.46
7.45
7.35
7.34
7.26 CDCl3
7.25
7.24
7.22
7.14
7.13
7.11
6.80
6.78
6.76
6.53
6.51
3.31
2.00
1.72 H2O
1.00
1.04
2.02
1.14
1.00
2.96
1.95
4.00
4.06
f1 (ppm)

45 (MFSB)

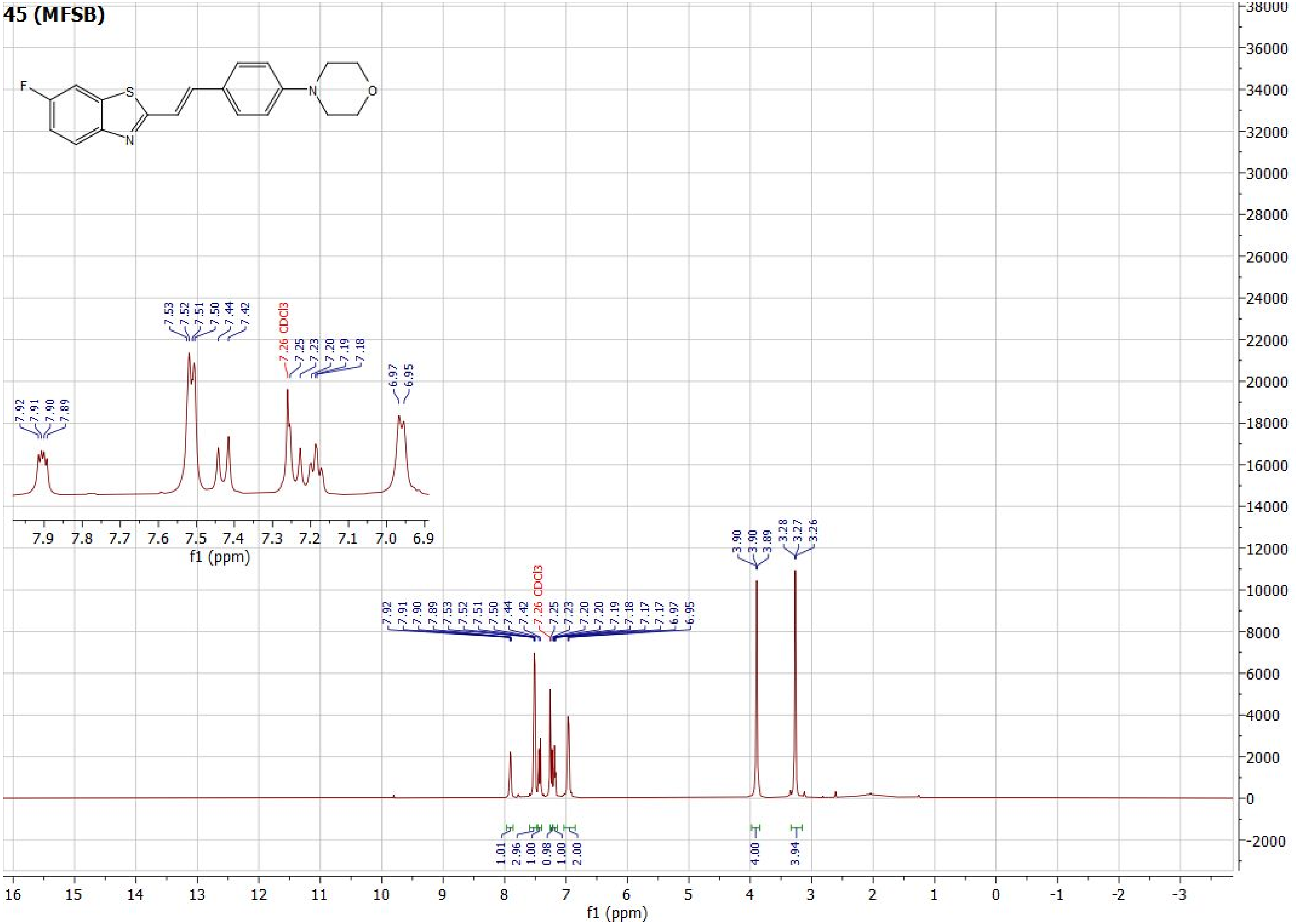

F
S
N
N
O
7.92
7.91
7.90
7.89
7.53
7.52
7.51
7.50
7.44
7.42
7.26 CDCl3
7.25
7.23
7.20
7.19
7.18
6.97
6.95
f1 (ppm)
3.90
3.90
3.89
3.28
3.27
3.26
1.01
2.96
1.00
0.98
1.00
2.00
4.00
3.94
f1 (ppm)

7. ^{1}H NMR spectra of radiolabeling precursors 48 and 49

48

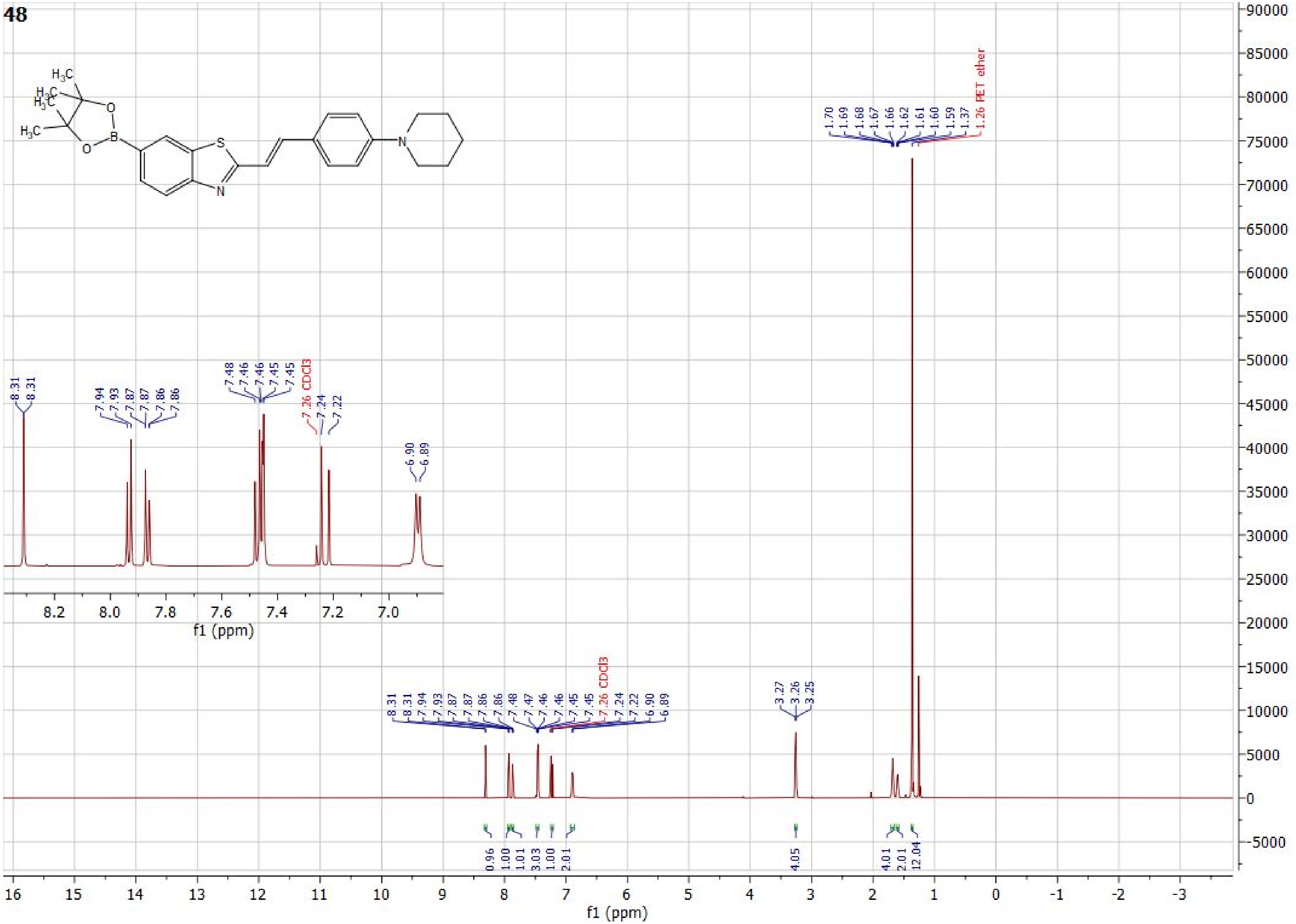

49

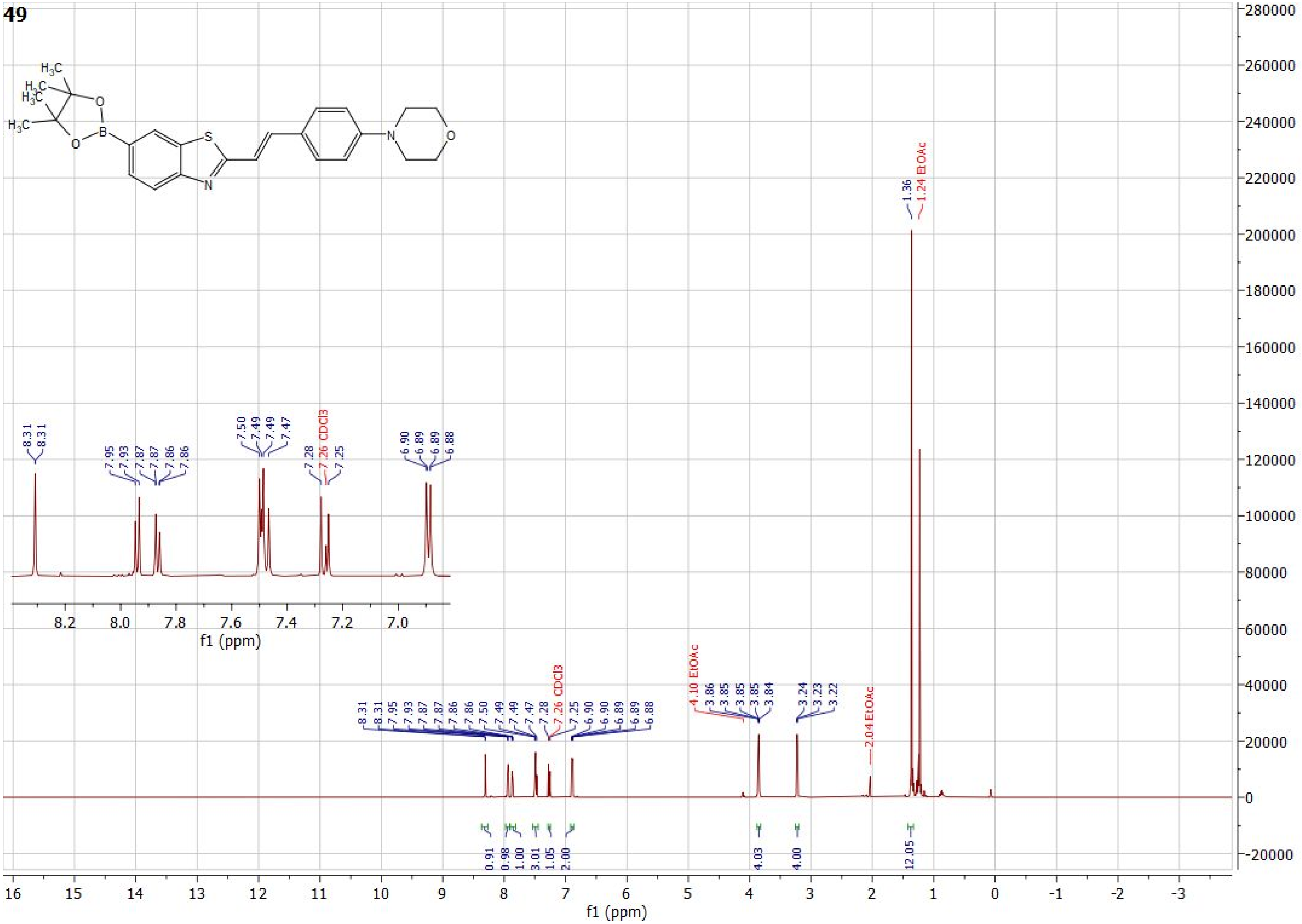

H3C
H3C
H3C
H3C
O
B
O
S
N
N
O
8.31
8.31
7.95
7.93
7.87
7.87
7.86
7.86
7.50
7.49
7.49
7.47
7.28
7.26 CDCl3
7.25
6.90
6.89
6.89
6.88
8.2 8.0 7.8 7.6 7.4 7.2 7.0
f1 (ppm)
4.10 EtOAc
3.86
3.85
3.85
3.84
3.24
3.23
3.22
2.04 EtOAc
1.36
1.24 EtOAc
0.91
0.98
1.00
3.01
1.05
2.00
4.03
4.00
12.05
16 15 14 13 12 11 10 9 8 7 6 5 4 3 2 1 0 -1 -2 -3
f1 (ppm)
280000 260000 240000 220000 200000 180000 160000 140000 120000 100000 80000 60000 40000 20000 0 -20000
